# Syndecan-1 Amplifies Ovalbumin-Induced Airway Remodeling by Strengthening TGFβ1/Smad3 Action

**DOI:** 10.3389/fimmu.2021.744477

**Published:** 2021-10-04

**Authors:** Dong Zhang, Xin-rui Qiao, Wen-Jing Cui, Jin-tao Zhang, Yun Pan, Xiao-fei Liu, Liang Dong

**Affiliations:** ^1^ Department of Respiratory, Shandong Qianfoshan Hospital, Cheeloo College of Medicine, Shandong University, Jinan, China; ^2^ Department of Respiratory, Shandong Institute of Respiratory Diseases, The First Affiliated Hospital of Shandong First Medical University, Shandong Provincial Qianfoshan Hospital, Shandong University, Jinan, China

**Keywords:** syndecan-1, ovalbumin, airway remodeling, TGFβ1/Smad3 signaling, asthma

## Abstract

Syndecan-1 (SDC-1) is a transmembrane proteoglycan of heparin sulfate that can regulate various cell signal transduction pathways in the airway epithelial cells and fibroblasts. Airway epithelial cells and human bronchial fibroblasts are crucial in airway remodeling. However, the importance of SDC-1 in the remodeling of asthmatic airways has not been confirmed yet. The present study was the first to uncover SDC-1 overexpression in the airways of humans and mice with chronic asthma. This study also validated that an increase in SDC-1 expression was correlated with TGFβ1/Smad3-mediated airway remodeling *in vivo* and *in vitro*. A small interfering RNA targeting SDC-1 (SDC-1 siRNA) and homo-SDC-1 in pcDNA3.1 (pc-SDC-1) was designed to assess the effects of SDC-1 on TGFβ1/Smad3-mediated collagen I expression in Beas-2B (airway epithelial cells) and HLF-1 (fibroblasts) cells. Downregulation of the SDC-1 expression by SDC-1 siRNA remarkably attenuated TGFβ1-induced p-Smad3 levels and collagen I expression in Beas-2B and HLF-1 cells. In addition, SDC-1 overexpression with pc-SDC-1 enhanced TGFβ1-induced p-Smad3 level and collagen I expression in Beas-2B and HLF-1 cells. Furthermore, the levels of p-Smad3 and collagen I induced by TGFβ1 were slightly increased after the addition of the recombinant human SDC-1 protein to Beas-2B and HLF-1 cells. These findings *in vitro* were also confirmed in a mouse model. A short hairpin RNA targeting SDC-1 (SDC-1 shRNA) to interfere with SDC-1 expression considerably reduced the levels of p-Smad3 and remodeling protein (α-SMA, collagen I) in the airways induced by ovalbumin (OVA). Similarly, OVA-induced p-Smad3 and remodeling protein levels in airways increased after mice inhalation with the recombinant mouse SDC-1 protein. These results suggested that SDC-1 of airway epithelial cells and fibroblasts plays a key role in the development of airway remodeling in OVA-induced chronic asthma.

## Introduction

Asthma is an inflammatory disease of the airways induced by allergens, gastroesophageal reflux, and genetic factors with complex pathogenesis (1). The normal structures and functions of bronchial epithelial cells and human fibroblasts are necessary to the standard functioning of lung tissues. Epithelial cells in the airways undergo interstitial transformation with disease progression, and fibroblasts are activated and differentiated into myofibroblasts (2). These cells produce numerous extracellular matrix proteins, including type I collagen and α-smooth muscle actin (α-SMA) (2). These changes in airway composition transform normal repairable injury responses to pathologic consequences, such as loss of epithelial integrity and excessive matrix deposition in interstitial, which, in turn, accelerate the structural remodeling of the airways (3).

Transforming growth factor β1 (TGFβ1), an important cytokine, mediates and regulates cellular transformation in various diseases (4). Large amounts of TGFβ1 are secreted after cell injury and other factors, which then participate in the regulation of various transcription factors and the synthesis of protein components of the extracellular matrix, thereby accelerating changes in cellular structures (5). Smad3, a member of the Smad protein family, is a TGFβ1 signaling downstream activator (6). TGFβ1/Smad3 signaling transduction plays a key regulatory role in the progression of liver fibrosis (7), pulmonary fibrosis (8), renal fibrosis (9), airway remodeling (10), cardiac structural remodeling (11), skin scar hyperplasia (12), and other diseases.

SDC-1 is a transmembrane heparin sulfate proteoglycan that carries various side chains, such as heparin sulfate (HS), chondroitin sulfate (CS), and hyaluronic acid (HA) (13). The SDC-1 core protein comprises extracellular, transmembrane, and intracellular regions (14). SDC-1 shedding has been widely studied in septic-related lung and kidney injury models (15). SDC-1 abscission is affected by many factors, such as inflammation and oxidative stress. In addition, Filla et al. (16) found that the cell surface proteoglycan SDC-1 mediates fibroblast growth factor-2 binding and activity. Hayashida et al. (17) reported that SDC-1 expression in epithelial cells can be induced by TGFβ1 through a PKA-dependent pathway. The special structural functions of SDC-1 in various physiological and pathological processes, such as cell proliferation (18), cell adhesion (19), and cell scarring (12), have been extensively studied. However, the exact role of SDC-1 in patients with asthma remains unclear.

The acute and chronic asthma models induced by OVA are respectively dominated by inflammation (20) and airway remodeling (21). SDC-1 has not been clearly described in OVA-induced acute or chronic asthma models. Therefore, the changes in SDC-1 in airway epithelium and alveolar lavage fluid (BALF) were further observed in OVA-induced acute or chronic asthma models. SDC-1 is hypothesized to be involved in airway remodeling by affecting TGFβ1/Smad3 signal transduction due to its role in regulating multiple cellular signal transduction pathways. Results suggested that SDC-1 is an important regulatory factor in the development of TGFβ1/Smad3 signal-mediated airway remodeling. Therefore, SDC-1 might be a novel therapeutic target in the pathologic form of airway remodeling in patients with asthma.

## Materials and Methods

### Reagents

Ovalbumin (OVA) and 4, 6-diamino-2-phenylindole dihydrochloride (DAPI) were purchased from Sigma-Aldrich (USA). The rabbit monoclonal antibodies SDC-1, p-Smad3, Smad3, and collagen I were prepared by Abcam (USA). Meanwhile, the rabbit monoclonal antibody GAPDH, HRP-conjugated goat anti-rabbit IgG, Alexa Fluor goat anti-rabbit, recombinant human TGFβ1, recombinant mouse SDC-1, and recombinant human SDC-1 were acquired from RD Biotechnology Ltd. Small interfering RNA targeting SDC-1 (SDC-1 siRNA), homo-SDC-1 in PCDNA3.1 (pc-SDC-1), and SDC-1 short hairpin RNA (SDC-1 shRNA) were synthesized by Gene Chem Co., Ltd. (Jinan, China).

### Human Subject

Human subjects were diagnosed with chronic asthma following the criteria of the 2019 Edition Global Asthma Initiative, and age-matched healthy subjects were enrolled at Qianfoshan Hospital (Jinan, China). The characteristics of patients with asthmatic and healthy subjects are shown in **Table 1**. This study was approved by the Human Research Ethics Review Committee of Qianfoshan Hospital of Shandong University.

**Table 1:** Characteristics and physiologies of asthmatic patients and the normal.

Characteristic	Normal	Asthmatic
Number	24	24
Age (years)	63.8 ± 6.4	60.7 ± 7.0
Gender (male/female)	12/12	11/13
FEV1 (% of predicted)	114.6 ± 8.5	42.3± 14.6
BDT	Negative	Positive
Past history of cough	Occasional	Always
Other diseases: COPD, lung tumors, coronary heart disease, rheumatic heart disease, allergic rhinitis, bronchitis	NO	NO

Data are depicted as means ± SD. FEV1, forced expiratory volume in one second; FEV1/FVC, the ratio of forced expiratory volume in the first second to forced vital capacity; BDT, bronchial dilation test.

### Cell Culture and Treatment

Beas-2B and HLF-1 cells (Cell Line Bank, Shanghai, China) were cultured in different DMEM containing 10% fetal bovine serum. The cells were seeded in 6- or 24-well culture plates under the following conditions: an incubator at 37°C, 95% air, 5% CO_2_, and a humid atmosphere. Serum-free synchronization was conducted for 24 h before the experiment and then used in all tests.

Beas-2B and HLF-1 cells were stimulated by 10 ng/mL TGFβ1 at different times (1, 2, 3, and 4 days), and changes in the cells were observed. The appropriate stimulating time point of TGFβ1 for Beas-2B or HLF-1 cells was selected by observing the protein levels of SDC-1, p-Smad3, Smad3, and collagen I.

The cell experiment was divided into four groups: 1) NC siRNA group: serum-free culture+siRNA negative control; 2) SDC-1 siRNA 1: cells cultured in serum-free culture and transfected with SDC-1 siRNA 1; 3) SDC-1 siRNA 2: cells cultured in serum-free culture and transfected with SDC-1 siRNA 2; 4) SDC-1 siRNA 3: cells cultured in serum-free culture and transfected with SDC-1 siRNA 3. Lipofectamine 3000 and NC siRNA or SDC-1 siRNA were added into the cell culture dishes for transfection according to the transfection manual. The SDC-1 siRNA and NC siRNA sequences were as follows: 1) SDC-1 siRNA 1: 5′-CCAAAUUGUGGCUACUAAUTT-3′; 2) SDC-1 siRNA 2: 5′-GCAGGACUUCACCUUUGAATT-3′; 3) SDC-1 siRNA 3: 5′-CCACCAAACAGGAGGAAUUTT-3′; 4) NC siRNA: 5′-UUCUCCGAACGUGUCACGUTT-3′. The effectiveness of SDC-1 siRNA in silencing SDC-1 protein expression was used for further studies.

Cells were then further divided into five groups: 1) Control group: cells cultured in serum-free culture without any stimulus factor; 2) NC siRNA group: serum-free culture+siRNA negative control; 3) SDC-1 siRNA: cells cultured in serum-free culture and transfected with SDC-1 siRNA. 4) NC siRNA+TGFβ1 group: cells cultured in serum-free culture with 10 ng/mL TGFβ1 and transfected with NC siRNA; 5) SDC-1 siRNA+TGFβ1: cells cultured in serum-free culture with 10 ng/mL TGFβ1 and transfected with SDC-1 siRNA. Lipofectamine 3000 and NC siRNA or SDC-1 siRNA were also transfected with cells according to the transfection manual.

The experiment was further divided into the following groups to observe the effects of pc-SDC-1: 1) Control group: cultured in serum-free culture without any stimulus factor; 2) pcDNA3.1 group: serum-free culture+pcDNA3.1 negative control; 3) pc-SDC-1 group: cells cultured in serum-free culture and transfected with pc-SDC-1; 4) TGFβ1 group: cells cultured in serum-free culture with 10 ng/mL TGFβ1; 5) pc-SDC-1+TGFβ1: cells cultured in serum-free culture with 10 ng/mL TGFβ1 and transfected with pc-SDC-1 for SDC-1 overexpression. The CDS region of the NM_002997 transcript of the SDC-1 gene was constructed into the pcDNA3.1 plasmid vector (**Figure 5A**), and pcSDC-1 was transferred into cells to generate SDC-1 overexpression according to the transfection manual. The effective of pc-SDC-1 in overexpressing SDC-1 protein was used for further studies.

Then, cells were divided into the following groups to investigate the effects of human recombinant SDC-1 protein: 1) Control group; 2) TGFβ1 group; 3) TGFβ1+recombinant SDC-1 (500 ng/mL); 4) TGFβ1+recombinant SDC-1 (1000 ng/mL); 5) TGFβ1+recombinant SDC-1 (2000 ng/mL). The cells in the TGFβ1+recombinant SDC-1 groups were c-ultured with 10 ng/mL TGFβ1 and the different dosages of human recombinant SDC-1 protein.

### Animals and Grouping

C57BL/6 female mice (18–20 g) were purchased from Pengyue Animal Breeding Co., Ltd. (Jinan, China). The mice were raised on an OVA-free diet without pathogens. The mice were then studied in accordance with the National Institutes of Health guidelines for the care and use of laboratory animals and approved by the Institutional Review Committee of Shandong University–Qianfoshan Hospital.

The mice were randomly divided into four groups to observe the dynamic changes in each index (n = 8/group): 1) Control-4 weeks group; 2) OVA-4 weeks group; 3) Control-8 weeks group; 4) OVA-8 weeks group. The sensitization, challenges, and atomization processes are shown in **Figure 2A**.

The Control-8 weeks and OVA-8 weeks groups were selected as the research object to further study SDC-1. The mice were randomly divided into four groups (n = 8/group): 1) Control group; 2) LV2NC group; 3) OVA group; 4) OVA+SDC-1 shRNA group. The specific process is outlined in **Figures 8A1, A2**. The LV2NC or SDC-1 shRNA sequences were as follows: 1) LV2NC: 5′-TTCTCCGAACGTGTCACACGT-3′; 2) SDC-1 shRNA: 5′-GAACAAGACTTCACCTTTGAA-3′.

Then, mice were divided into four groups to asses the effects of mouse recombinant SDC-1 protein on OVA-induced chronic asthma (n = 8/group): 1) Control group; 2) Recombinant SDC-1 group; 3) OVA group; 4) OVA+recombinant SDC-1 group. The mice in the OVA+recombinant SDC-1 group were inhaled with recombinant SDC-1 (2000 ng/mL) after preatment with OVA (3% g/mL). The specific process is outlined in **Figure 8A3**.

### Morphological Changes in the Airways

Lung tissues were fixed with 4% paraformaldehyde for 2 days, dehydrated with alcohol, embedded with paraffin, and finally cut into 5 μm thick paraffin sections. Airway inflammation was assessed by hematoxylin and eosin staining (HE). Collagen deposition was evaluated by Masson staining. The tissue sections were scored in accordance with the method described by Mohammadtursun et al. (22).

### Immunohistochemistry Assay

The 5 μm thick paraffin sections were antigenically repaired and endogenous peroxidase was removed, and then SDC-1 antibody was incubated at 4°C for 15 h. Subsequently, the lung tissue sections were washed with PBS solution and incubated with goat anti-rabbit antibody in the dark for 40 min. The lung tissue sections were washed with PBS solution and then incubated with DAB solution for 4 min. Finally, the tissue sections were dehydrated with alcohol, dewaxed until transparent with xylene, and photographed under a microscope. Protein expression was evaluated using Image J.

### Western Blot Analysis

The protein samples were added to 12% SDS polyacrylamide gel for electrophoresis separation, and then the proteins were transferred to a PVDF membrane, which was incubated with 5% skim milk for 90 min at room temperature. The primary antibodies (SDC-1, p-Smad3, Smad3, collagen I, α-SMA, and GAPDH) were incubated at 4°C for 15 h after washing with TBST solution. The PVDF membrane was washed with TBST and then incubated with HRP-conjugated goat anti-rabbit IgG for 1.5 h. The PVDF membrane was washed three times with TBST and then visualized using an enhanced chemiluminescence reagent.

### Immunofluorescence

The lung tissues were repaired with citric acid antigen and washed with PBS and then incubated with the antibodies of SDC-1, TGFβ1, p-Smad3, collagen I, and α-SMA at 4°C for 15 h. The lung tissues were washed with PBS and then stained with Alexa Fluor goat anti-rabbit and DAPI. Finally, the lung tissues were observed and photographed under a fluorescence microscope (Olympus, Japan).

The Beas-2B or HLF-1 cells were seeded on soul plates in 24-well plates. The cells were fixed with 4% paraformaldehyde after different pretreatments and treatments and incubated with the antibody of SDC-1 overnight at 4°C. The soul plates were washed with PBS and then stained with Alexa Fluor goat anti-rabbit and DAPI. Finally, the cell images were photographed under a fluorescence microscope (Olympus, Japan).

### Measurement Inflammatory Cytokine and SDC-1 Level in BALF

The mice were anesthetized with 4% chloral hydrate and intubated. BALF was obtained *via* multiple intratracheal injections of PBS (a total of 1.0 mL PBS). BALF was centrifuged at 3500 rpm at 4°C; for 15 min, and the supernatant was collected. Levels of SDC-1 (Zcibio Biotechnology, China), IL-4 (70-EK204/2, Multisciences), IL-5 (70-EK205, Multisciences), and IL-13 (70-EK213, Multisciences) in BALF were detected using an ELISA kit.

### Statistical Analysis

All data were expressed as the mean ± standard deviation, and differences among groups were analyzed by one-way ANOVA and SNK test. p < 0.05 was considered statistically significant. All statistical analyses were performed using SPSS19.0.

## Results

### SDC-1 Is Overexpressed in the Bronchial Epithelium of Human Chronic Asthma

**Figure 1:**
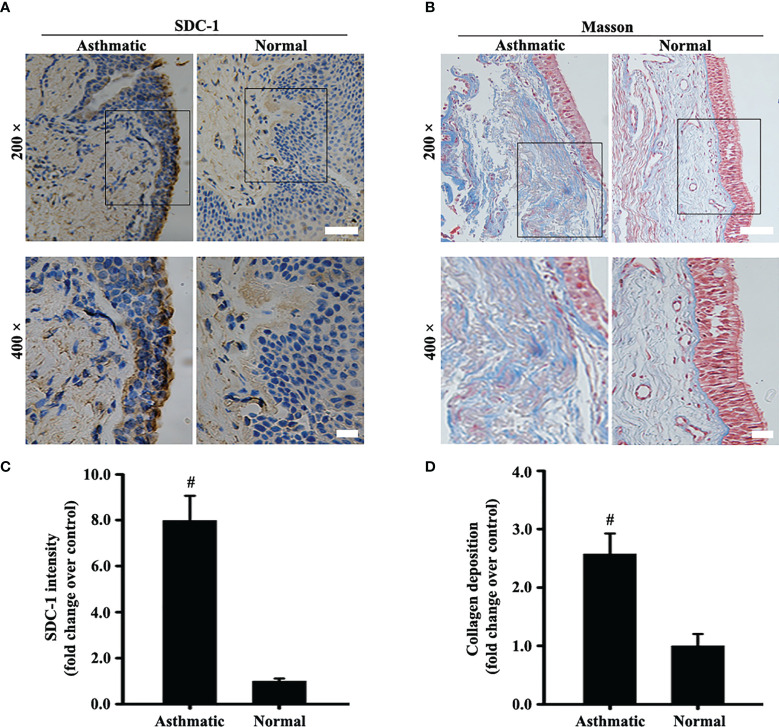
SDC-1 levels in the asthmatic and normal human bronchial samples were evaluated by immunohistochemical staining **(A)**. Collagen deposition in the asthmatic and normal human bronchial samples was evaluated by Masson staining **(B)**. **(C)** SDC-1 intensity analysis of **(A). (D)** Collagen intensity analysis of **(B)** Data are shown as the means ± SD of three independent experiments. ^#^p < 0.05 *versus* the control group.

The protein level of SDC-1 in the human subjects with chronic asthma was detected by immunohistochemistry. Results showed that SDC-1 was overexpressed in the lung airways of the asthmatic subjects, and the degree of overexpression in the tracheal epithelium was considerably higher than that around the trachea (**Figures 1A, C** **)**. In addition, Masson staining indicated that substantial amounts of collagen were deposited around the bronchial duct in the subjects with chronic asthma, suggesting the possible involvement of SDC-1 in airway remodeling (**Figures 1B, D** **)**.

### Syndecan-1 Is Upregulated in the Bronchial Epithelium of OVA-Induced Chronic Asthma

**Figure 2:**
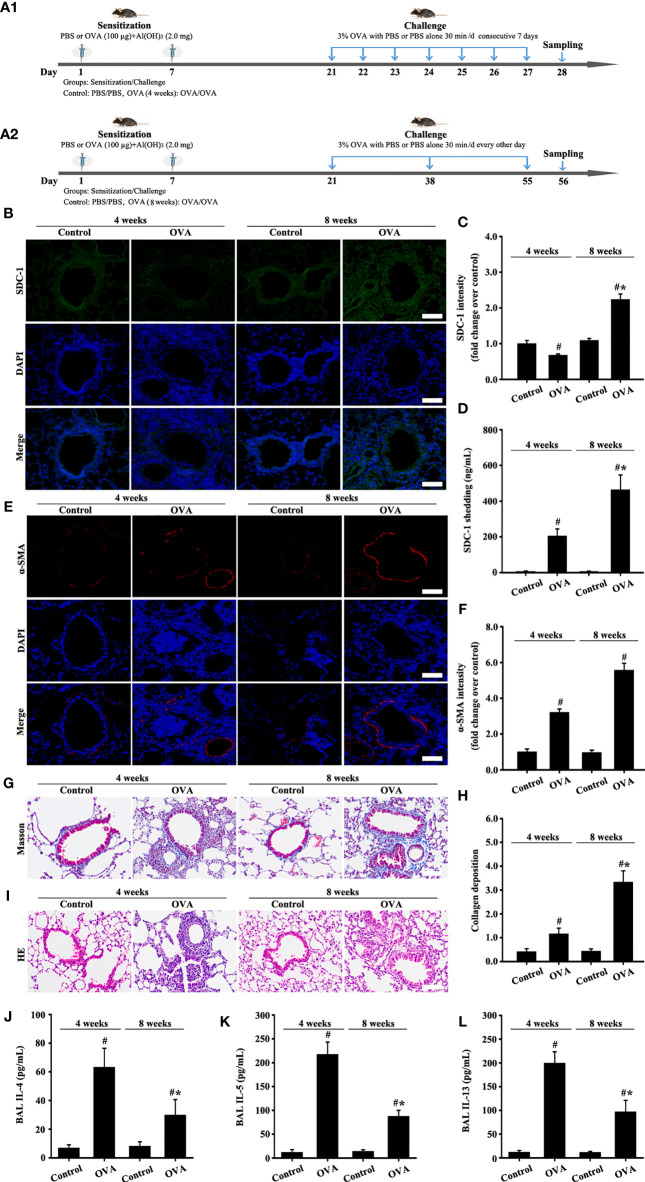
Animal experimental schedule is shown in **(A)**. SDC-1 **(B)** and α-SMA **(E)** levels were detected by immunofluorescence. Collagen deposition was evaluated by Masson staining **(G)**. Histopathologic changes were detected by HE staining **(I)**. Levels of SDC-1 shedding **(D)**, IL-4 **(J)**, IL-5 **(K)**, and IL-13 **(L)** in BALF were measured by ELISA. **(C, F)** immunofluorescence intensity analysis of **(B, E). (H)** collagen deposition intensity analysis of **(G)** Data are shown as the means ± SD of three independent experiments. ^#^p < 0.05 *versus* the control group. ^#^*p < 0.05 *versus* the OVA (4 weeks) or control group.

Changes in SDC-1 expression in the airways of mice with acute and chronic asthma were detected by immunofluorescence. Results showed that SDC-1 expression slightly decreased in the tracheal epithelium and periductal of OVA-induced acute asthma compared with normal mice (**Figures 2B, C** **)**. However, SDC-1 expression remarkably increased in the tracheal epithelium and periductal of OVA-induced chronic asthma (**Figures 2B, C** **)**. In addition, SDC-1 levels in the BALF were higher in chronic asthma than in acute asthma (**Figure 2D**)

The results of masson staining and α-SMA indicated that airway remodeling induced by OVA-induced was considerably higher in chronic asthma than in acute asthma (**Figures 2E-H**).

HE staining and ELISA detection results of inflammatory factors (IL-4, IL-5, and IL-13) showed that the inflammatory response was substantially higher in OVA-induced acute asthma (OVA-4 weeks group) than that in chronic asthma (OVA-8 weeks group) (**Figures 2I-L**). These findings suggested that SDC-1 was overexpressed in human subjects with chronic asthma and mice with OVA-induced chronic asthma.

### TGFβ1/Smad3 and Collagen I Are Upregulated in the Bronchial Epithelium of OVA-Induced Acute and Chronic Asthma

**Figure 3:**
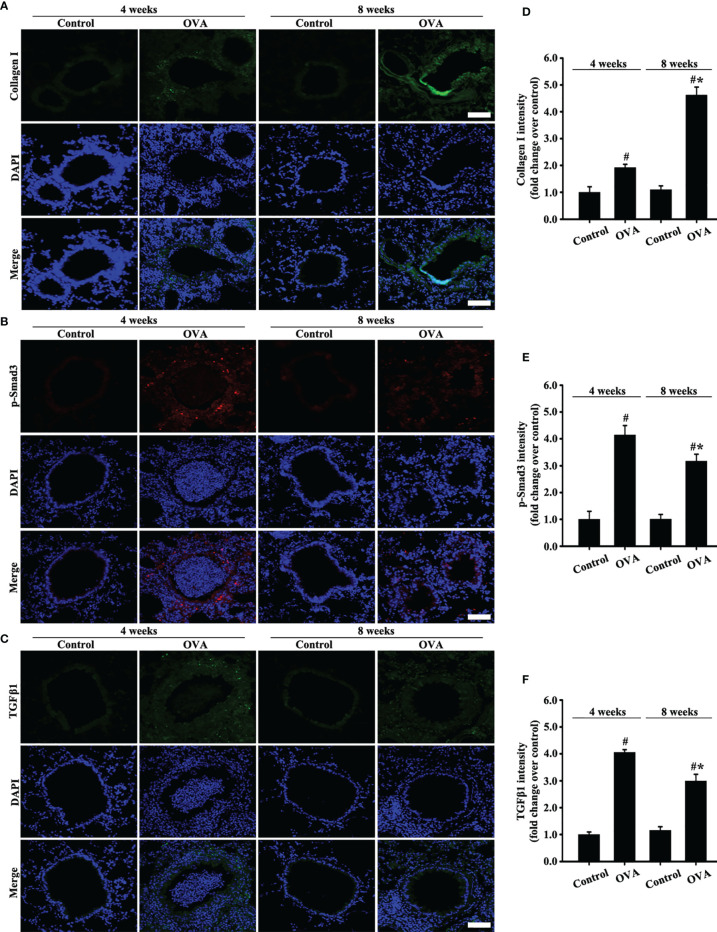
Animal experimental schedule is shown in **Figures 2A**. Collagen I **(A)**, p-Smad3 **(B)**, and TGFβ1 **(C)** were detected by immunofluorescence. **(D–F)** Immunofluorescence intensity analysis of **(A–C)**, respectively. Data are shown as the means ± SD of three independent experiments. ^#^p < 0.05 *versus* the control group. ^#^*p < 0.05 *versus* the OVA (4 weeks) or control group.

Moreover, collagen I expression in the airways of mice with OVA-induced acute asthma was higher than that in normal mice (**Figures 3A, D** **)**. Meanwhile, collagen I expression in the airways of mice with chronic asthma induced by OVA sharply increased compared with that in the acute asthma group (**Figures 3A, D** **)**.

TGFβ1/Smad3 signaling plays an important role in airway remodeling. Accordingly, changes in the expression of TGFβ1/Smad3 and collagen I in the airways with acute and chronic asthma were detected *via* immunofluorescence. Results showed that TGFβ1 and p-Smad3 expression substantially increased in the airways of mice with acute asthma induced by OVA compared with normal mice (**Figures 3B, C, E, F**). However, TGFβ1 and p-Smad3 expression slightly decreased in the OVA-induced chronic asthma compared with the acute asthma group.

### SDC-1 siRNA Deletion of SDC-1 Suppresses Collagen I Expression by TGFβ1/Smad Signaling in Beas-2B Cells

**Figure 4:**
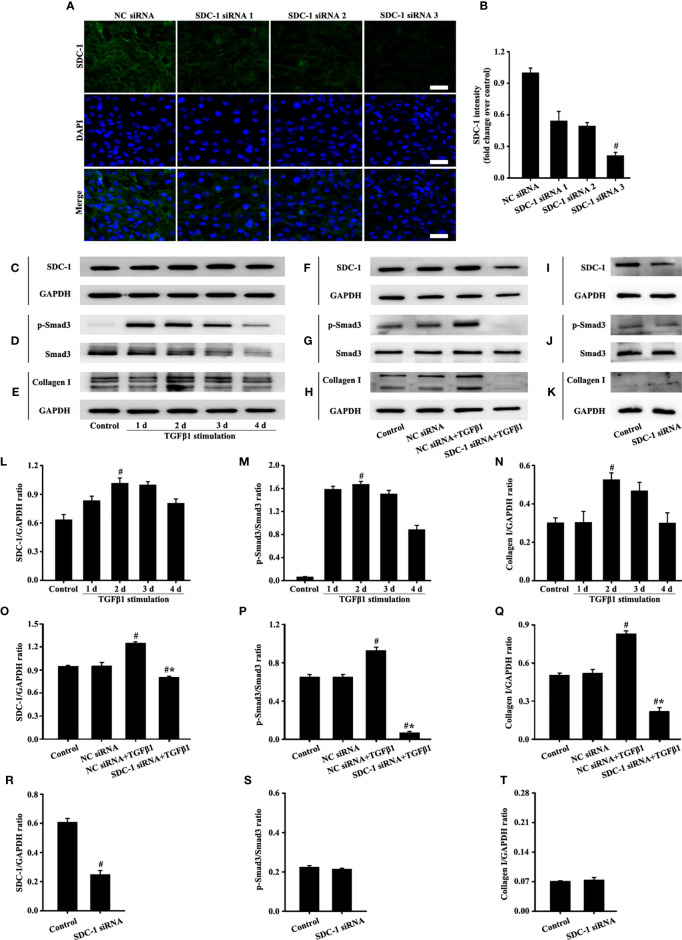
Effects of SDC-1 siRNA on collagen I and p-Smad3 in Beas-2B cells were observed. Deletion effects of SDC-1 siRNA 1, SDC-1 siRNA 2, and SDC-1 siRNA 3 on SDC-1 expression **(A)** were detected by immunofluorescence. Changes in SDC-1 **(C)**, p-Smad3 **(D)**, and collagen I **(E)** under TGFβ1 stimulation at different periods were detected by Western blot. Effects of SDC-1 siRNA 3 on SDC-1 **(F**, **I)**, p-Smad3 **(G**, **J)**, and collagen I **(H**, **K)** were detected by Western blot. **(B)** SDC-1 immunofluorescence intensity analysis of **(A)**. **(L–T)** Protein intensity analysis of **(C**-**K)**, respectively. Data are shown as the means ± SD of three independent experiments. ^#^p < 0.05 *versus* the control group. ^#^*p < 0.05 *versus* the NC siRNA+TGFβ1 group.

The siRNA targeting SDC-1 was used to knock out SDC-1 expression to evaluate the role of SDC-1 in Beas-2B cells. Immunofluorescence revealed that three kinds of SDC-1 siRNA downregulated SDC-1 expression, and the most remarkable downregulation of SDC-1 expression was observed after SDC-1 siRNA 3 interference (**Figures 4A, B** **)**. Therefore, SDC-1 siRNA 3 was selected for downstream studies to target the knockout of SDC-1 expression.

TGFβ1/Smad3 signaling plays an important role in collagen deposition. Therefore, changes in the expression of SDC-1, p-Smad3, and collagen I over time under TGFβ1 stimulation were detected by Western blot analysis. Results showed that SDC-1, p-Smad3, and collagen I expressions substantially increased after 2 days of TGFβ1 stimulation (**Figures 4C-E, L-N**). Therefore, TGFβ1 stimulation for 2 days was selected in the downstream study.

Interestingly, Western blot analysis revealed that TGFβ1 stimulation substantially induced the levels of SDC-1, p-Smad3 and collagen I in Beas-2B cells compared with those in the control group (**Figures 4F-H, O-Q**). However, SDC-1 downregulation by SDC-1 siRNA strongly inhibited the levels of p-Smad3 and collagen I induced by TGFβ1 stimulation in Beas-2B cells (**Figures 4F-H, O-Q**). In addition, SDC-1 intervention alone had no significant difference in p-Smad3 and Collagen I compared with those in the control group (**Figures 4I-K, R-T**).

These results suggested that SDC-1 siRNA might improve collagen deposition by inhibiting TGFβ1/Smad3 signaling.

### SDC-1 Overexpression Aggravates Collagen I Expression by TGFβ1/Smad Signaling in Beas-2B Cells

**Figure 5:**
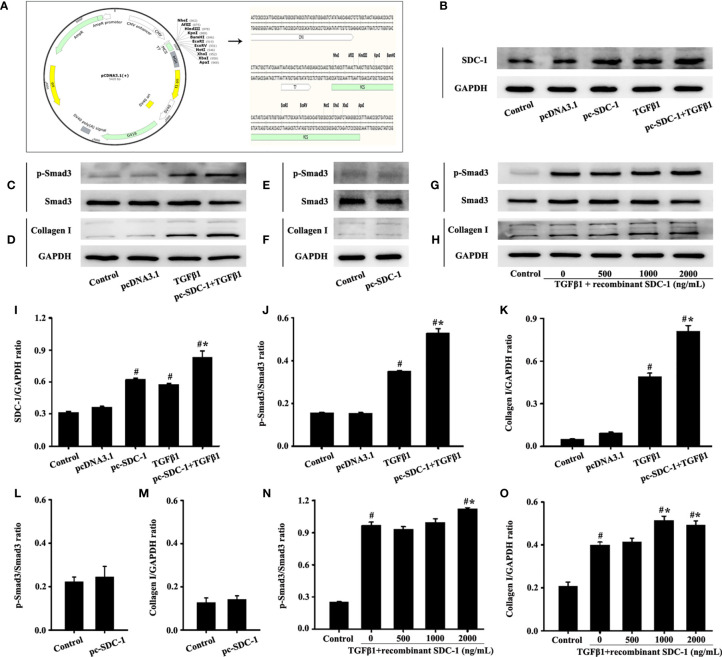
Effects of SDC-1 overexpression by pc-SDC-1 or addition of the recombinant human SDC-1 protein on the levels of collagen I and p-Smad3 in Beas-2B cells were observed. Plasmid vector atlas of SDC-1 overexpression **(A)**. Effect of pc-SDC-1 overexpression on TGFβ1-stimulated SDC-1 expression **(B)** was detected by Western blot. Effect of SDC-1 overexpression on the levels of p-Smad3 **(C, E)** and collagen I **(D, F)** were detected. Effects of the addition of different concentrations of the recombinant human SDC-1 protein on TGFβ1-stimulated p-Smad3 **(G)** and collagen I **(H)** were detected by Western blot. **(I–O)** Protein intensity analysis of **(B–H)**, respectively. Data are shown as the means ± SD of three independent experiments. ^#^p < 0.05 *versus* the control group. ^#^*p < 0.05 *versus* the TGFβ1 group.

A plasmid vector was constructed to overexpress SDC-1 and further explore the role of SDC-1 in p-Smad3 and collagen I expressions. Interestingly, the pc-SDC-1 considerably increased TGFβ1-induced SDC-1 expression compared with the TGFβ1 group (**Figures 5B, I** **)**. The pc-SDC-1 upregulation of SDC-1 expression also notably enhanced the level of TGFβ1-induced p-Smad3 and collagen I expressions in Beas-2B cells (**Figures 5C, D, J, K**). In addition, SDC-1 overexpression alone had no significant difference in p-Smad3 and Collagen I compared with those in the control group (**Figures 5E, F, L, M**).

The effects of exogenous recombinant human SDC-1 addition on p-Smad3 and collagen I expressions were also investigated. Results demonstrated that high concentrations of recombinant human SDC-1 slightly increased the levels of p-Smad3 and collagen I induced by TGFβ1 compared with those in the TGFβ1 group (**Figures 5G, H, N, O**).

These results suggested that SDC-1 overexpression and SDC-1 detachment might aggravate collagen deposition by enhancing TGFβ1/Smad3 signaling.

### SDC-1 siRNA Deletion of SDC-1 Suppresses Collagen I Expression by TGFβ1/Smad Signaling in HLF-1 Cells

Fibroblasts play an important role in airway remodeling. SDC-1 expression in fibroblasts was obtained through database retrieval (www.proteinatlas.org). Therefore, the effects of SDC-1 on TGFβ1/Smad3 signal in fibroblasts were further explored.

**Figure 6:**
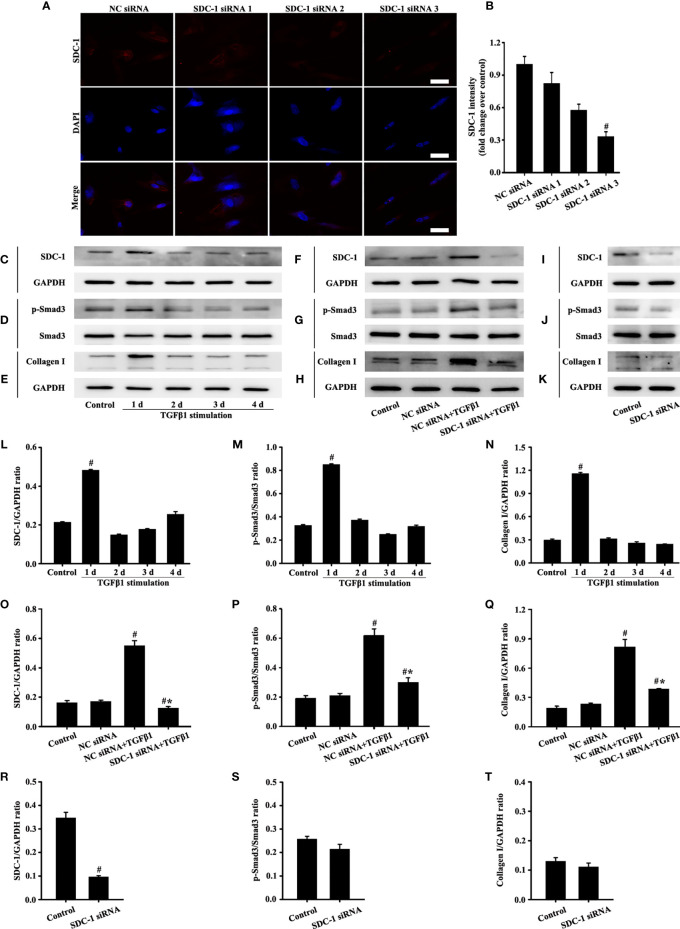
Effects of SDC-1 siRNA on HLF-1 cells were detected. Deletion effects of SDC-1 siRNA 1, 2, or 3 on SDC-1 expression **(A)** were observed by immunofluorescence. Changes in levels of SDC-1 **(C)**, p-Smad3 **(D)**, and collagen I **(E)** under TGFβ1 stimulation were detected by Western blot. Effect of SDC-1 siRNA 3 on the levels of SDC-1 **(F**, **I)**, p-Smad3 **(G**, **J)**, and collagen I **(H**, **K)** were detected by Western blot. **(B)** SDC-1 immunofluorescence intensity analysis of **(A). (L–T)** Protein intensity analysis of **(C–K)**, respectively. Data are shown as the means ± SD of three independent experiments. ^#^p < 0.05 *versus* the control group. ^#^*p < 0.05 *versus* the NC siRNA+ TGFβ1 group.

The role of three SDC-1 siRNAs in HLF-1 cells was reevaluated to analyze the effects of SDC-1 on HLF-1 cells. Immunofluorescence results confirmed that SDC-1 expression was most notably downregulated after SDC-1 siRNA 3 interference (**Figures 6A, B** **)**. Therefore, SDC-1 siRNA 3 was also selected for the downstream study to target the knockout of SDC-1 expression in HLF-1 cells.

Western blot analysis revealed that SDC-1, p-Smad3 and collagen I substantially increased after 1 day of TGFβ1 stimulation (**Figures 6C-E, L-N**). Therefore, TGFβ1 stimulation for 1 day was selected in the downstream study of HLF-1 cells.

Western blot analysis revealed that SDC-1 downregulation by SDC-1 siRNA strongly inhibited the levels of TGFβ1-induced p-Smad3 and collagen I in HLF-1 cells compared with those in the TGFβ1-treated group (**Figures 6F-H, O-Q**). While downregulation SDC-1 alone had no significant difference in p-Smad3 and Collagen I compared with those in the control group (**Figures 6I-K, R-T**).

### SDC-1 Overexpression Aggravates Collagen I Deposition by TGFβ1/Smad Signaling in HLF-1 cells

**Figure 7:**
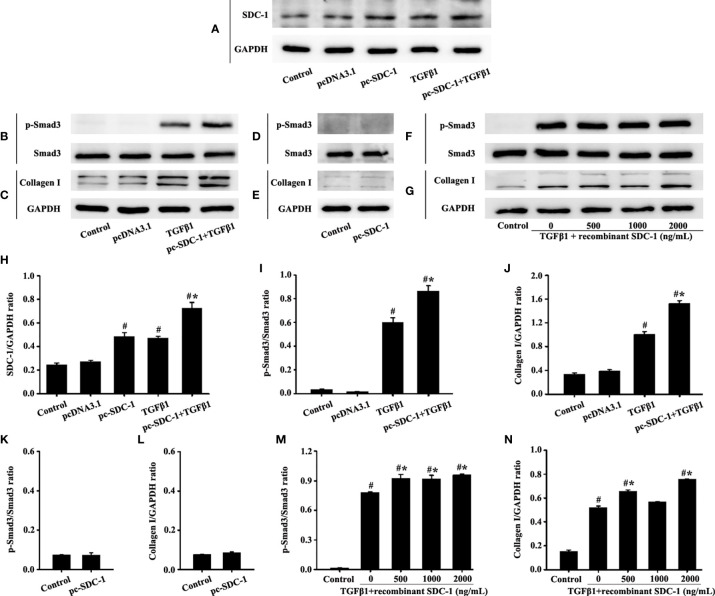
Effects of SDC-1 overexpression by pc-SDC-1 or the addition of the recombinant human SDC-1 protein on the collagen I and p-Smad3 in HLF-1 cells were observed. Effects of pc-SDC-1 overexpression on TGFβ1-stimulated SDC-1 expression **(A)** were detected. Effects of pc-SDC-1 on the levels of p-Smad3 **(B**, **D)** and collagen I **(C**, **E)** were detected by Western blot. Effects of the recombinant human SDC-1 protein addition on TGFβ1-stimulated p-Smad3 **(F)** and collagen I **(G)** expression were detected by Western blot. **(H**-**N)** Protein intensity analysis of **(A**-**G)**, respectively. Data are shown as the means ± SD of three independent experiments. ^#^p < 0.05 *versus* the control group. ^#^*p < 0.05 *versus* the TGFβ1 group.

Interestingly, the pc-SDC-1 substantially increased TGFβ1-induced SDC-1 expression in the pc-SDC-1+TGFβ1 group compared with that in the TGFβ1 group (**Figures 7A, H** **)**. The pc-SDC-1 also notably enhanced the level of TGFβ1-induced p-Smad3 and collagen I in the pc-SDC-1+TGFβ1 group compared with that in the TGFβ1 group (**Figures 7B, C, I, J**). However, SDC-1 overexpression by pc-SDC-1 alone had no significant difference in p-Smad3 and Collagen I compared with those in the control group (**Figures 7D, E, K, L**).

p-Smad3 and collagen I expression in the TGFβ1+recombinant human SDC-1 group increased compared with that in the TGFβ1 group (**Figures 7F, G, M, N**). These results indicated that SDC-1 overexpression might transfuse signaling to fibroblasts to enhance collagen expression.

### SDC-1 Overexpression Accelerates Airway Remodeling-Induced by OVA in Chronic Asthma

The similarities in SDC-1 overexpression in mice and humans with chronic asthma suggested that airway remodeling had a conserved effect across species. Therefore, SDC-1 deficient mice with lentivirus were constructed to determine the functional role of SDC-1 in airway remodeling.

**Figure 8:**
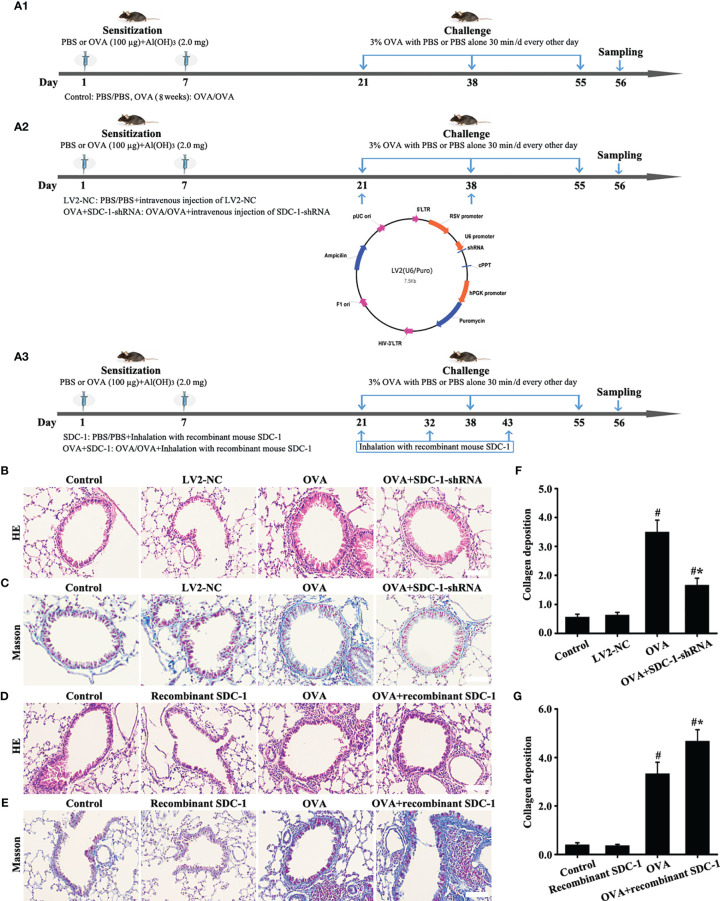
Animal experimental intervention schedule is shown in **(A)**. Effects of SDC-1 deletion by SDC-1 shRNA or inhalation with the recombinant mouse SDC-1 protein on collagen deposition **(C**, **E)** and pathological changes **(B**, **D)** were observed. **(F, G)** Collagen intensity analysis of **(C, E)**, respectively. Data are shown as the means ± SD of three independent experiments. ^#^p < 0.05 *versus* the control group. ^#^*p < 0.05 *versus* the OVA group.

**Figure 9:**
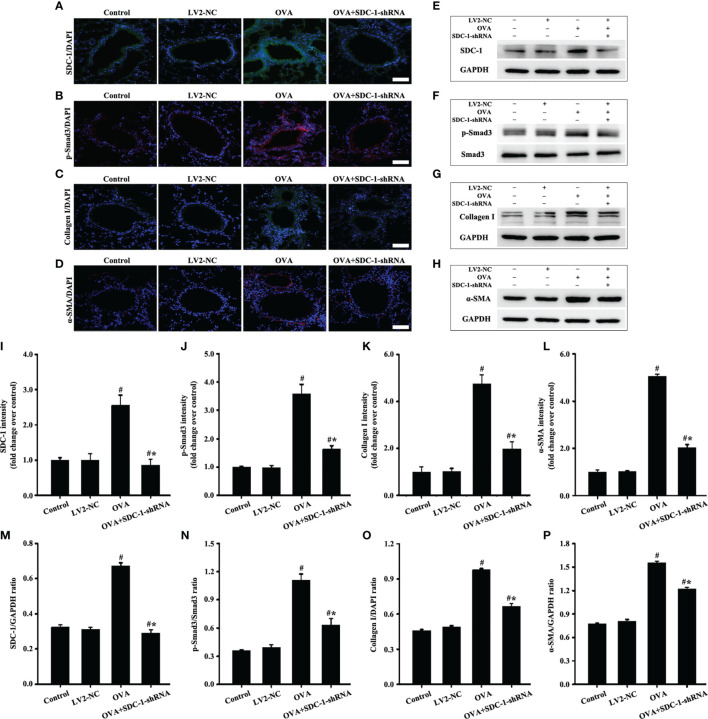
Animal experimental intervention schedule is shown in **Figures 8A1, A3**. Effects of SDC-1 deletion by SDC-1 shRNA on the levels of SDC-1 **(A**, **E)**, p-Smad3 **(B**, **F)**, collagen I **(C**, **G)**, and α-SMA **(D**, **H)** in mice were observed by immunofluorescence and Western blot. **(I–L)** Immunofluorescence intensity analysis of **(A–D)**, respectively. **(M–P)** Gray intensity analysis of **(E–H)**, respectively. Data are shown as the means ± SD of three independent experiments. ^#^p < 0.05 *versus* the control group. ^#^*p < 0.05 *versus* the OVA group.

No difference in the airway of control and LV2NC mice was found (**Figures 8B, C, F**). The mice with OVA-induced chronic asthma showed higher levels of inflammation and collagen deposition (**Figures 8B–G**) as well as SDC-1, p-Smad3, collagen I and α-SMA expression than the control group (**Figures 9A–P**). However, SDC-1 downregulation by SDC-1 shRNA in the OVA+SDC-1 shRNA group remarkably alleviated OVA-induced p-Smad3, collagen deposition, and collagen I and α-SMA expression compared with that in the OVA group (**Figures 8C, F** and **9A–P**).

These findings collectively clarify the mechanism of SDC-1 overexpression in airway remodeling in mice and humans.

### Inhalation With Recombinant Mouse SDC-1 Protein Accelerates Airway Remodeling-Induced by OVA in Chronic Asthma

ELISA detected the presence of shedding SDC-1 in the BALF, and the concentration of SDC-1 in mice with chronic asthma was higher than that in mice with acute asthma (**Figure 2D**). Thus, the mice were induced to inhale the recombinant mouse SDC-1 protein to investigate the effects of shedding SDC-1 on airway remodeling.

**Figure 10:**
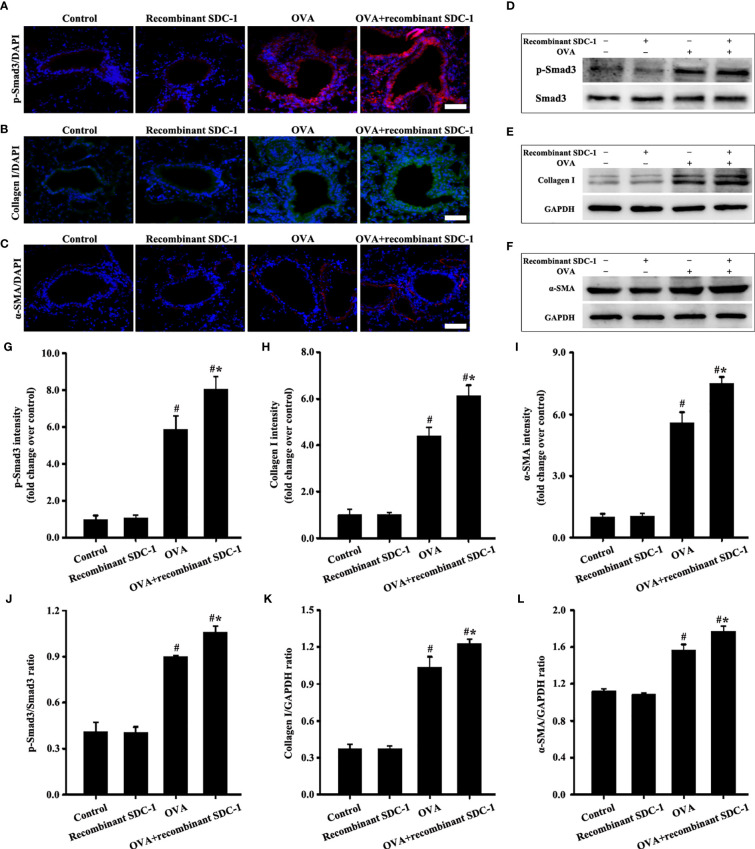
Animal experimental intervention schedule is shown in **Figures 8A1, A3**. Effects of inhalation with the recombinant mouse SDC-1 protein on the levels of p-Smad3 **(A**, **D)**, collagen I **(B**, **E)**, and α-SMA **(C**, **F)** in mice were observed. **(G–I)** Immunofluorescence intensity analysis of **A–C)**, respectively. **(J–L)** Gray intensity analysis of **(D–F)**, respectively. Data are shown as the means ± SD of three independent experiments. ^#^p < 0.05 *versus* the control group. ^#^*p < 0.05 *versus* the OVA group.

No difference was found in pathological form, Smad3 phosphorylation, collagen deposition, and α-SMA and collagen I expression in the airways of the control and inhalation with the recombinant mouse SDC-1 protein mice (**Figures 8D, E, G**, **10A–L**). Smad3 phosphorylation and collagen I and α-SMA expression induced by OVA increased compared with those in the control mice (**Figures 10A-L**). However, inhalation with the recombinant mouse SDC-1 protein in the OVA+SDC-1 group increased collagen deposition and the expression levels of p-Smad3, α-SMA, and collagen I compared with those in the OVA group (**Figures 8E, G**, and **10A-L**). These findings were consistent with the results of *in vitro* experiments.

These results suggested that shedding SDC-1 might strengthen TGFβ1/Smad3 signaling to participate in the airway remodeling of mice and humans.

## Discussion

**Figure 11:**
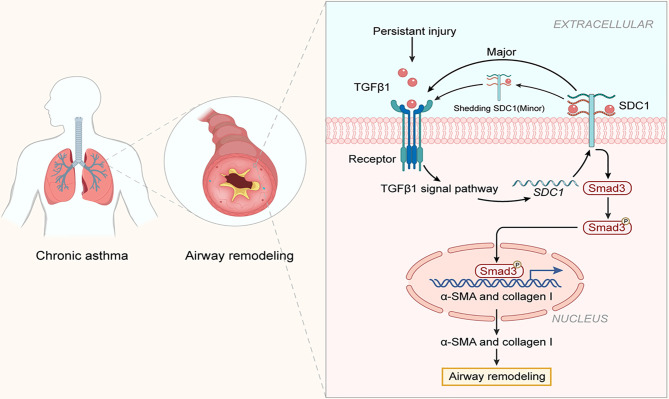
Structure of SDC-1 overexpression with aggravated airway remodeling by strengthening TGFβ1/Smad3 signaling in OVA-induced chronic asthma.

Airway remodeling is an important feature in the development of chronic asthma. Blocking or slowing down airway remodeling has become a hot topic in the research on asthma (23, 24). The present study was the first to demonstrate the involvement of SDC-1 in airway remodeling in chronic asthma *in vivo* and *in vitro*. The primary finding of this study was that SDC-1 may mediate TGFβ1/Smad3 signaling to participate in airway remodeling (**Figure 11**).

SDC-1, a major component of glycocalyx, is an important signaling transmembrane protein molecule. SDC-1 has an extracellular glycosaminoglycan chain comprising HS, HA, and CS (13, 15). First, SDC-1 protrudes from extracellular structural features and functions as a physical barrier, capturing signal molecules and directing them to receptors on the membrane (14). Second, the transmembrane structure and cytoplasmic region of SDC-1 can transmit extracellular signals into cells and then mediate a series of intracellular reactions (25). The previous study confirmed the role of SDC-1 as a barrier against permeability in the LPS-induced ARDS model (15). Parimon et al. (26) confirmed that SDC-1 promotes lung fibrosis by regulating epithelial reprogramming through extracellular vesicles. The present results showed that SDC-1 and collagen deposition synchronously increased in humans and mice with chronic asthma, whereas that after SDC-1 shRNA intervention in mice or SDC-1 siRNA intervention in cells synchronously decreased to reduce SDC-1 level. To a certain extent, this study also supported the idea that SDC-1 enhances intracellular signal transduction through the recruitment of its external domain amplification ligand.

The present study also found an interesting phenomenon that SDC-1 expression in bronchial epithelial and periductal cells decreased in humans and mice with acute asthma stage, whereas that in humans and mice with chronic asthma stage substantially increased. These results led to speculation that SDC-1 has different mechanisms of action in various disease stages. The inflammatory damage caused by inflammatory responses can lead to a certain increase in SDC-1 shedding (27, 28). Xu et al. (29) reported that the SDC-1 ectodomains of the airway epithelium are closely related to the inflammatory response of acute asthma. Changes in inflammatory cytokines, such as IL-4, IL-5, and IL-13 in BALF, were further detected in the present study. ELISA results showed that the levels of IL-4, IL-5, and IL-13 sharply increased in mice with acute asthma compared with normal mice. However, the levels of IL-4, IL-5, and IL-13 substantially decreased in mice with chronic asthma compared with those in mice with acute asthma. These results indicated the possible relation of shedding of the SDC-1 ectodomains of the airway epithelium in mice with acute asthma to acute inflammatory response. By contrast, the persistence of chronic inflammatory response had minimal effect on SDC-1 expression in chronic asthma.

The SDC-1 ectodomains of epithelial cells have anti-inflammatory, antipermeability, and other barrier effects. However, the shedding of the SDC-1 exodomain has different roles in various models. Li et al. (30) found that the abdication of the SDC-1 exogenous domain regulates bleomycin-induced intrapulmonary transport of neutrophils in acute lung injury. Xu et al. (29) uncovered that the shedding of the SDC-1 exodomain has anti-inflammatory properties in acute asthma. Different degrees of SDC-1 exodomain shedding in acute and chronic asthma were observed in the present study, and inhalation with SDC-1 promoted collagen deposition in OVA-induced chronic asthma models. These findings suggested that SDC-1 shedding has different regulatory functions in various disease models.

TGFβ1 is a kind of factor with multiple effects on the different disease models, such as pro-airway remodeling, as well as anti-inflammatory or pro-inflammatory effects (31, 32). Hogan et al. (33) reported that the TGFβ1 pathway is required for NF-κB signaling in mouse keratinocytes. However, TGFβ1 plays an anti-inflammatory, pro-inflammatory, and pro-airway remodeling role in the pathogenesis of asthma at different stages (34–36). TGFβ1 was substantially higher in mice with acute asthma than those with chronic asthma in the present study. These results led to speculation regarding the possible role of TGFβ1 levels in the production of IL-4, IL-5, and IL-13. In addition, TGFβ1 stimulates epithelial or fibroblast transcription and suppresses collagenase transcription, thus allowing TGFβ1/Smad3 signaling to play a key role in collagen deposition (37, 38). TGFβ1/Smad3 is crucial in promoting airway remodeling in chronic asthma, and this phenomenon was also verified in this study. Therefore, these results strongly suggested that SDC-1 overexpression played an important role in collagen I expression in the epithelial cells or fibroblasts through TGFβ1/Smad3 signaling.

Overall, the present study was the first to demonstrate that the high expression of SDC-1 in the trachea of patients with chronic asthma is closely related to airway remodeling, and SDC-1 may regulate collagen deposition in epithelial cells or fibroblasts through the TGFβ1/Smad signaling pathway. This study provides a theoretical basis for the treatment of asthma in the future.

## Data Availability Statement

The original contributions presented in the study are included in the article/**Supplementary Material**. Further inquiries can be directed to the corresponding author.

## Ethics Statement

The studies involving human participants were reviewed and approved by Human Research Ethics Review Committee of Qianfoshan Hospital. Written informed consent for participation was not required for this study in accordance with the national legislation and the institutional requirements. The animal study was reviewed and approved by Institutional Review Committee of Shandong University–Qianfoshan Hospital. Written informed consent was obtained from the individual(s) for the publication of any potentially identifiable images or data included in this article.

## Author Contributions

LD conceived the idea and proofread the manuscrift. DZ conceived the idea, conducted the study, drafted the figures, and wrote the original draft. J-TZ, W-JC, and X-RQ interpreted the data and drafted the figures. YP and X-FL proofread the manuscript. DZ contributed mainly to the article. All authors contributed to the article and approved the submitted version.

## Funding

This work was supported by the National Natural Science Foundation of China (81770029).

## Conflict of Interest

The authors declare that the research was conducted in the absence of any commercial or financial relationships that could be construed as a potential conflict of interest.

## Publisher’s Note

All claims expressed in this article are solely those of the authors and do not necessarily represent those of their affiliated organizations, or those of the publisher, the editors and the reviewers. Any product that may be evaluated in this article, or claim that may be made by its manufacturer, is not guaranteed or endorsed by the publisher.
