# Supplementary figures and images for: Syndecan-1 Amplifies Ovalbumin-Induced Airway Remodeling by Strengthening TGFβ1/Smad3 Action (part 1 of 2)
Source: Front Immunol. 2021 Oct 4;12:744477. doi: 10.3389/fimmu.2021.744477 (PMC8521046; doi:10.3389/fimmu.2021.744477)

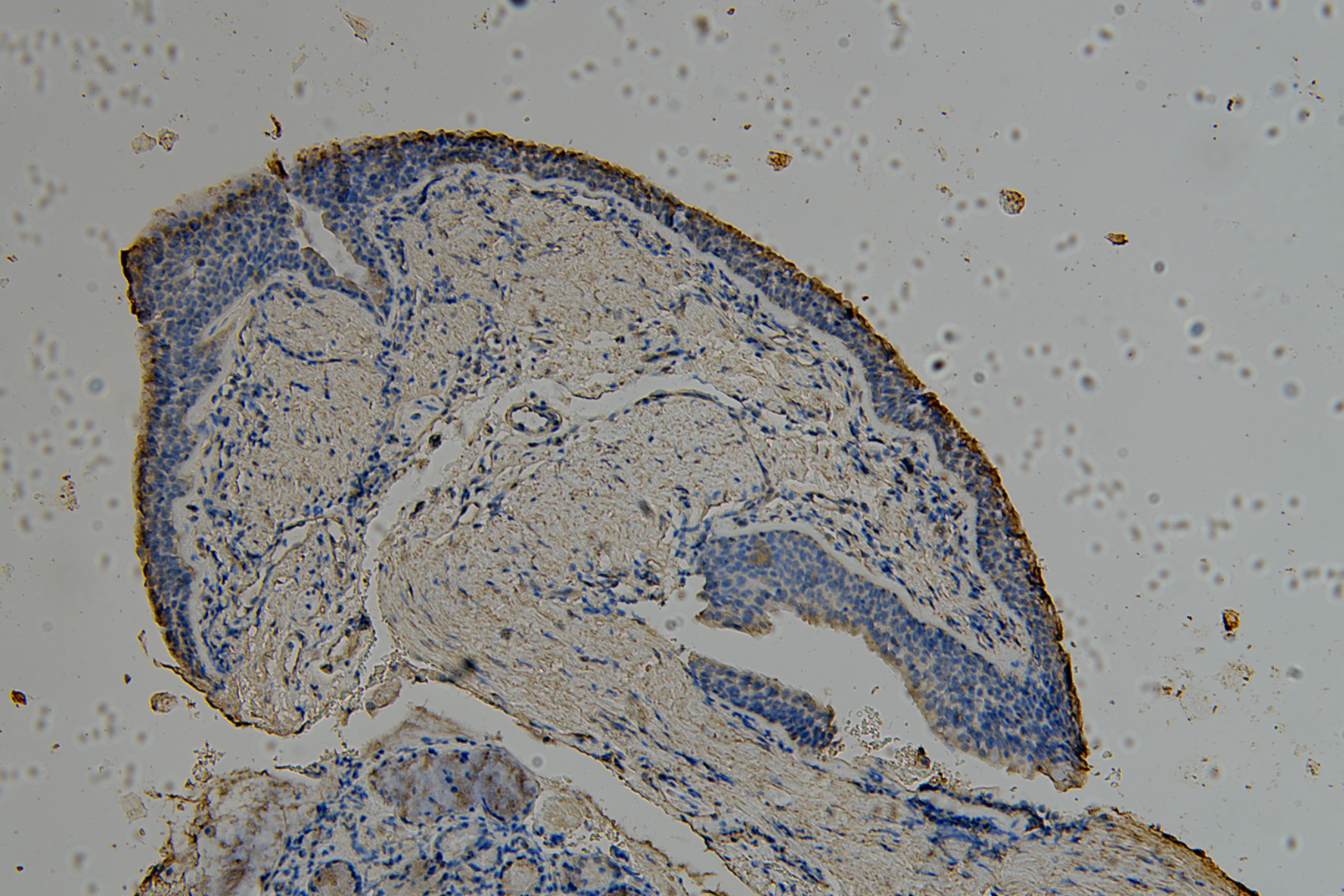

Supplement: Supplementary file 1 [file DataSheet_1.zip › Figure 1 raw datas/A. SDC-1/Asthmatic SDC-1 100X.tif]

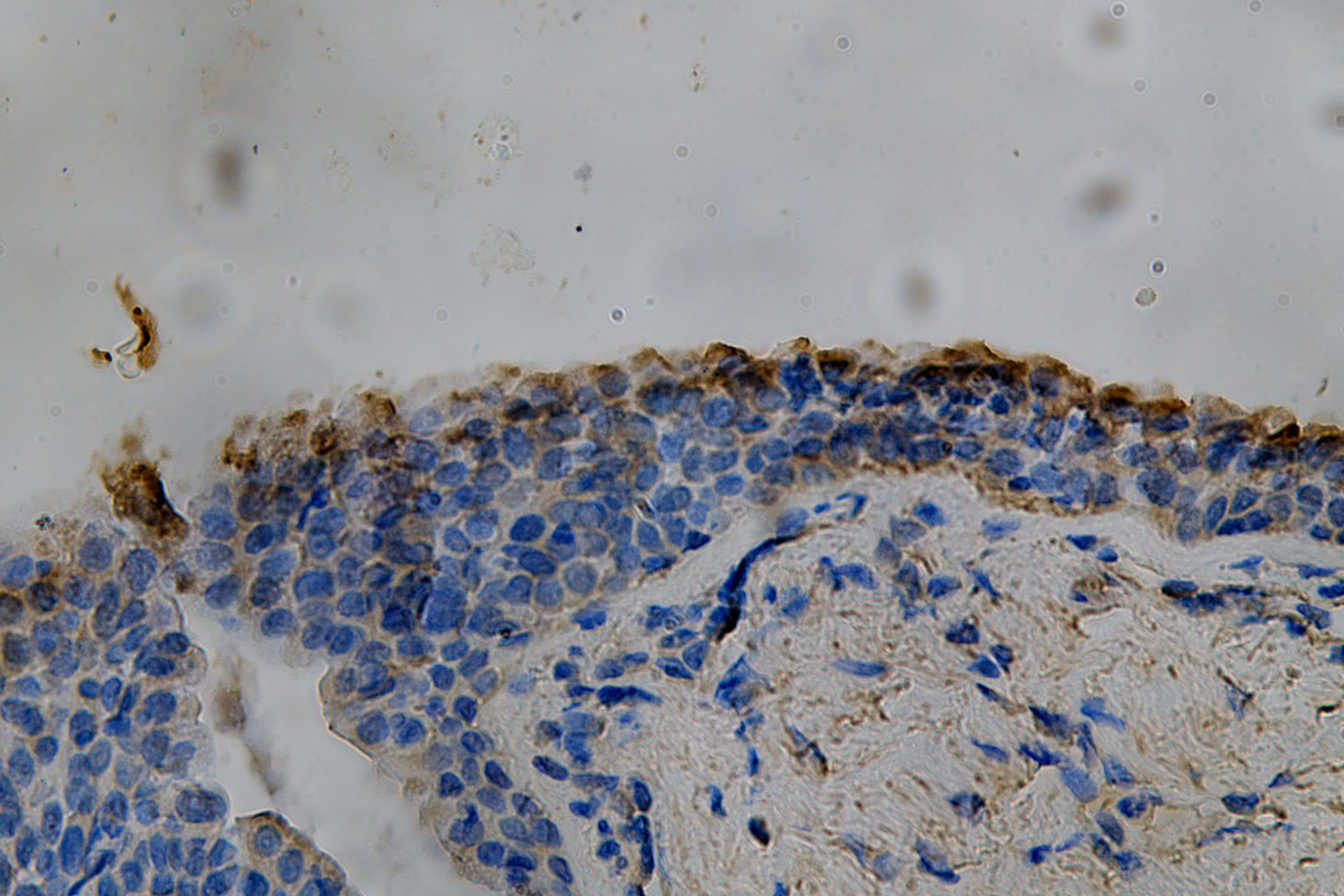

Supplement: Supplementary file 1 [file DataSheet_1.zip › Figure 1 raw datas/A. SDC-1/Asthmatic SDC-1 400X.tif]

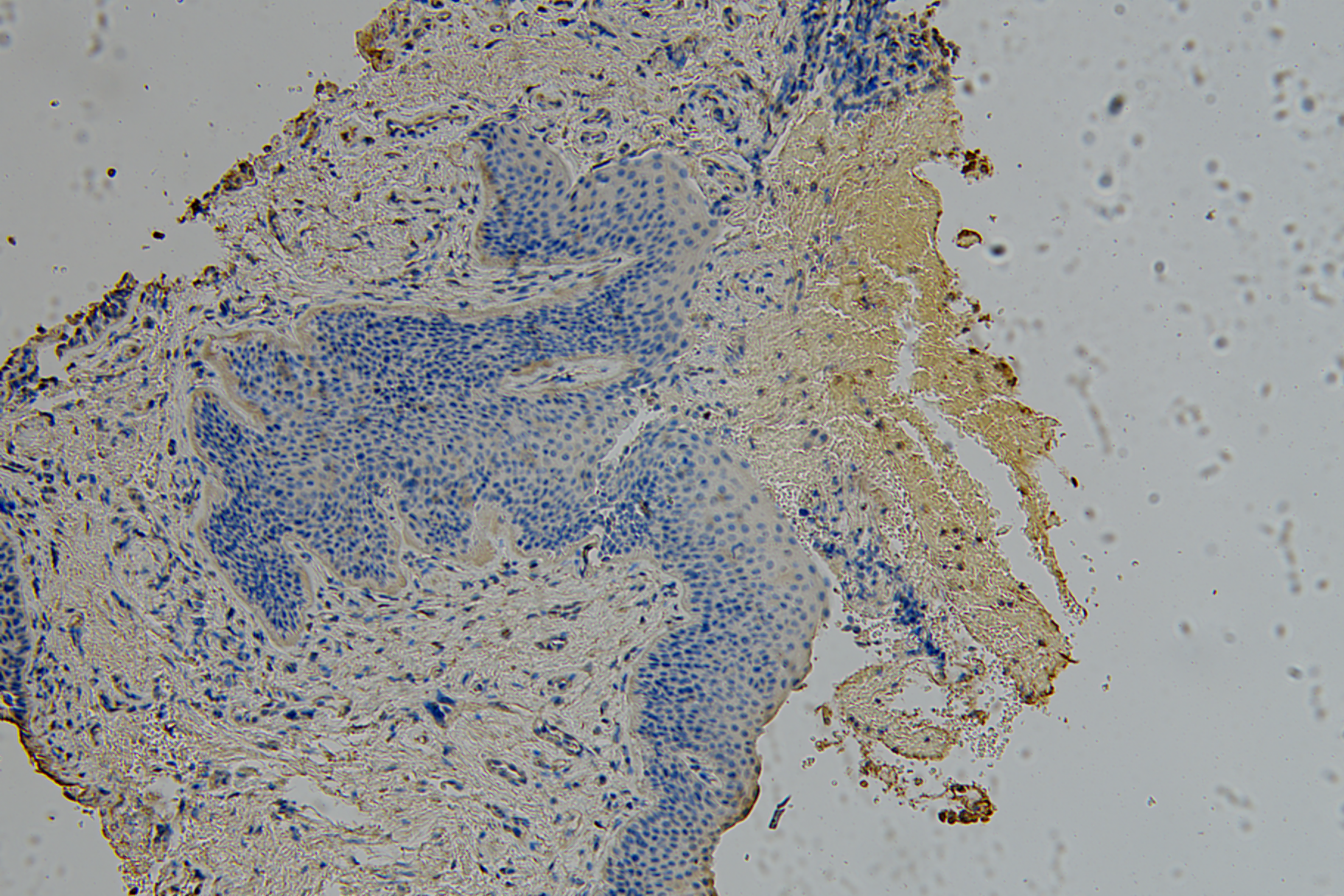

Supplement: Supplementary file 1 [file DataSheet_1.zip › Figure 1 raw datas/A. SDC-1/Normal SDC-1 100X.tif]

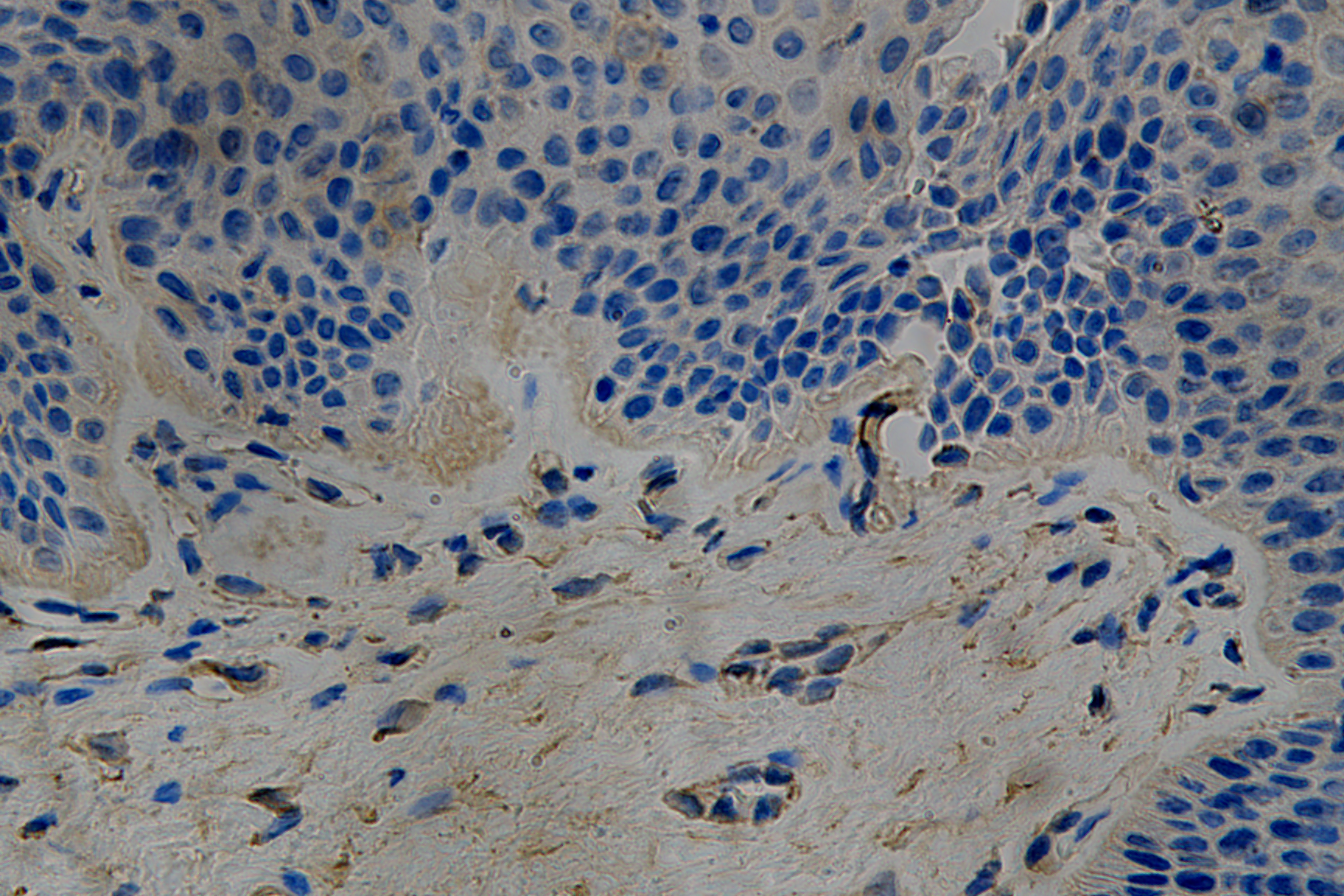

Supplement: Supplementary file 1 [file DataSheet_1.zip › Figure 1 raw datas/A. SDC-1/Normal SDC-1 400X.tif]

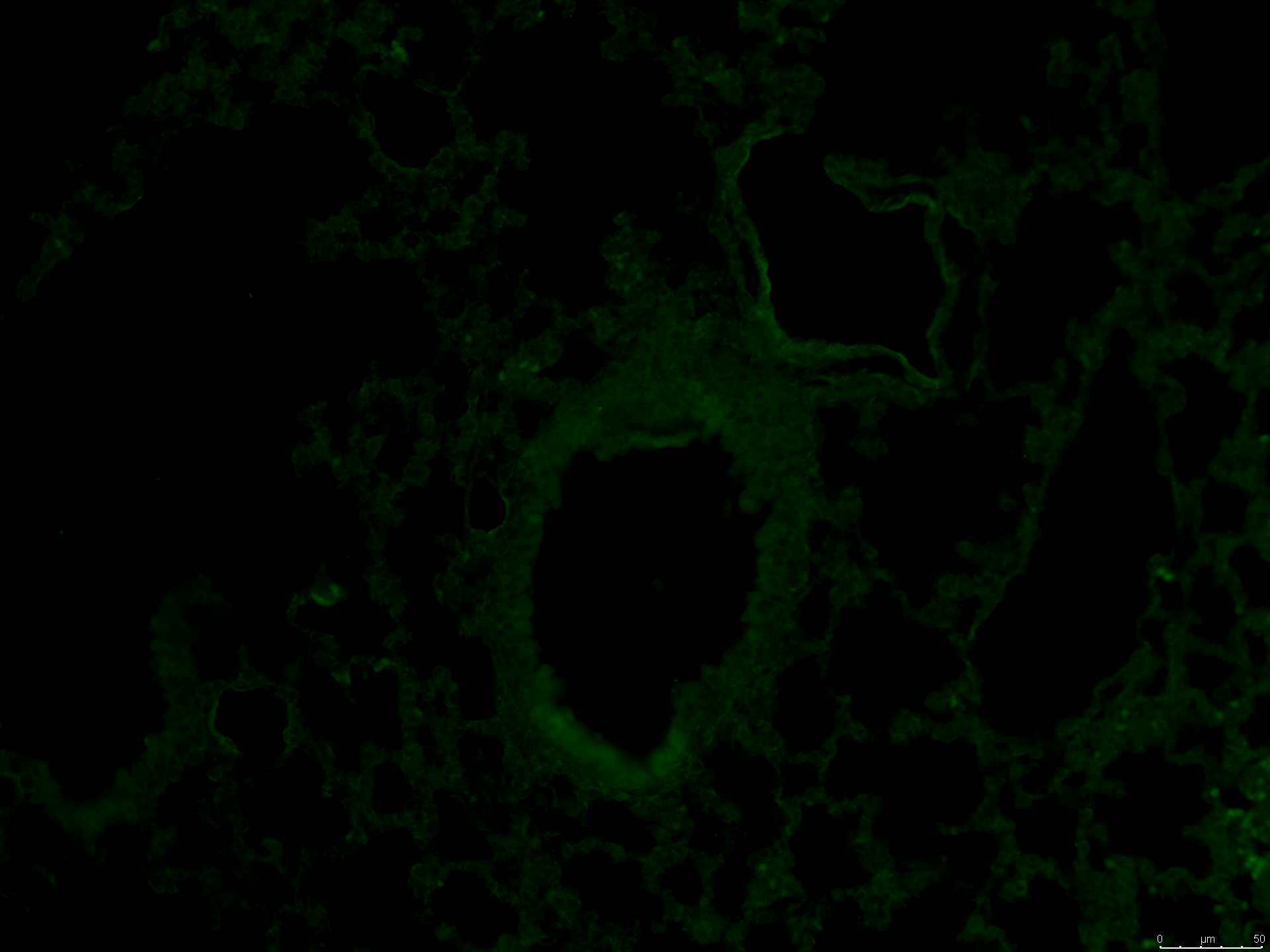

Supplement: Supplementary file 2 [file DataSheet_2.zip › Figure 2 raw datas/B. SDC-1/Control-4weeks 1.tif]

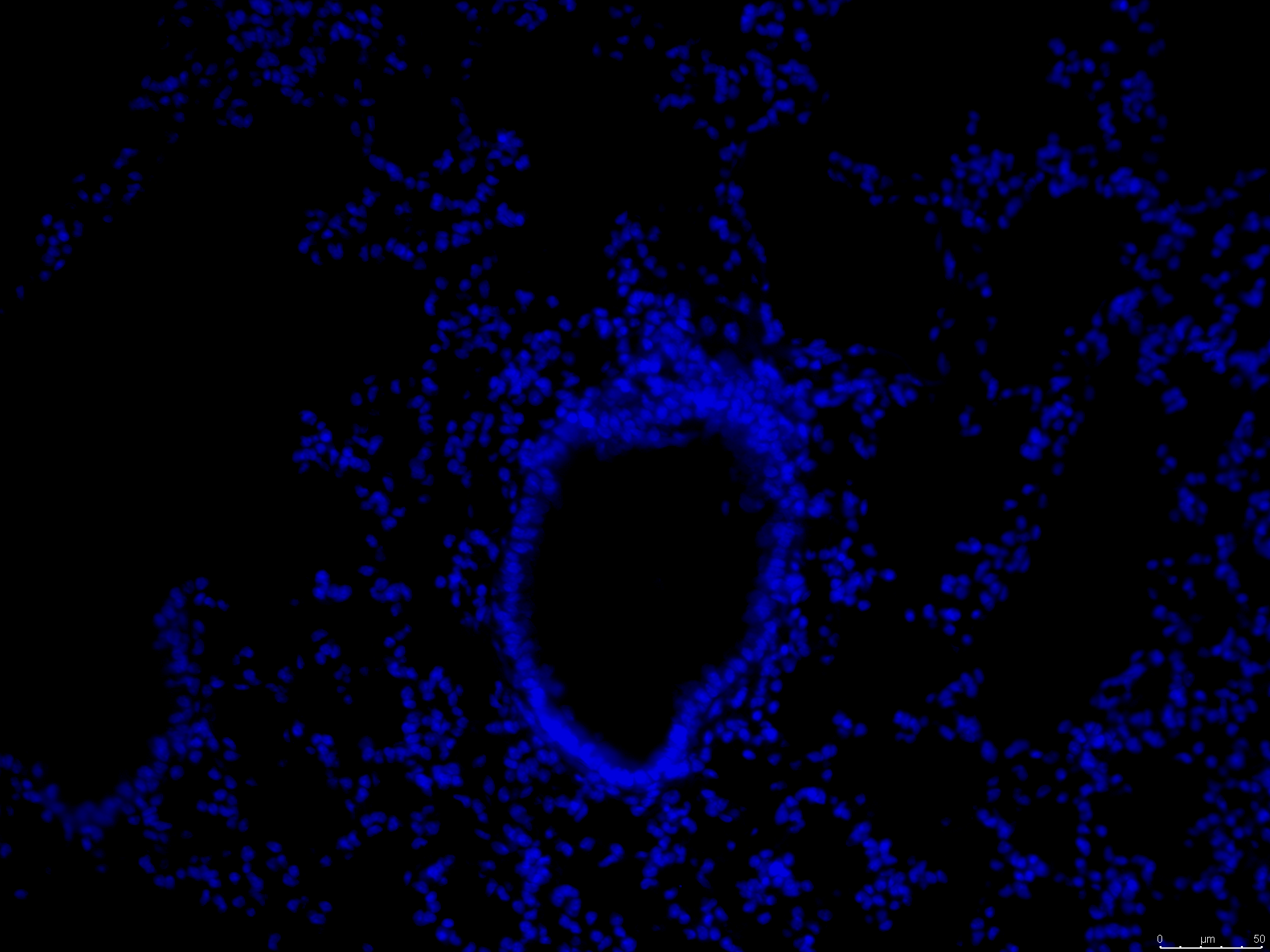

Supplement: Supplementary file 2 [file DataSheet_2.zip › Figure 2 raw datas/B. SDC-1/Control-4weeks 2.tif]

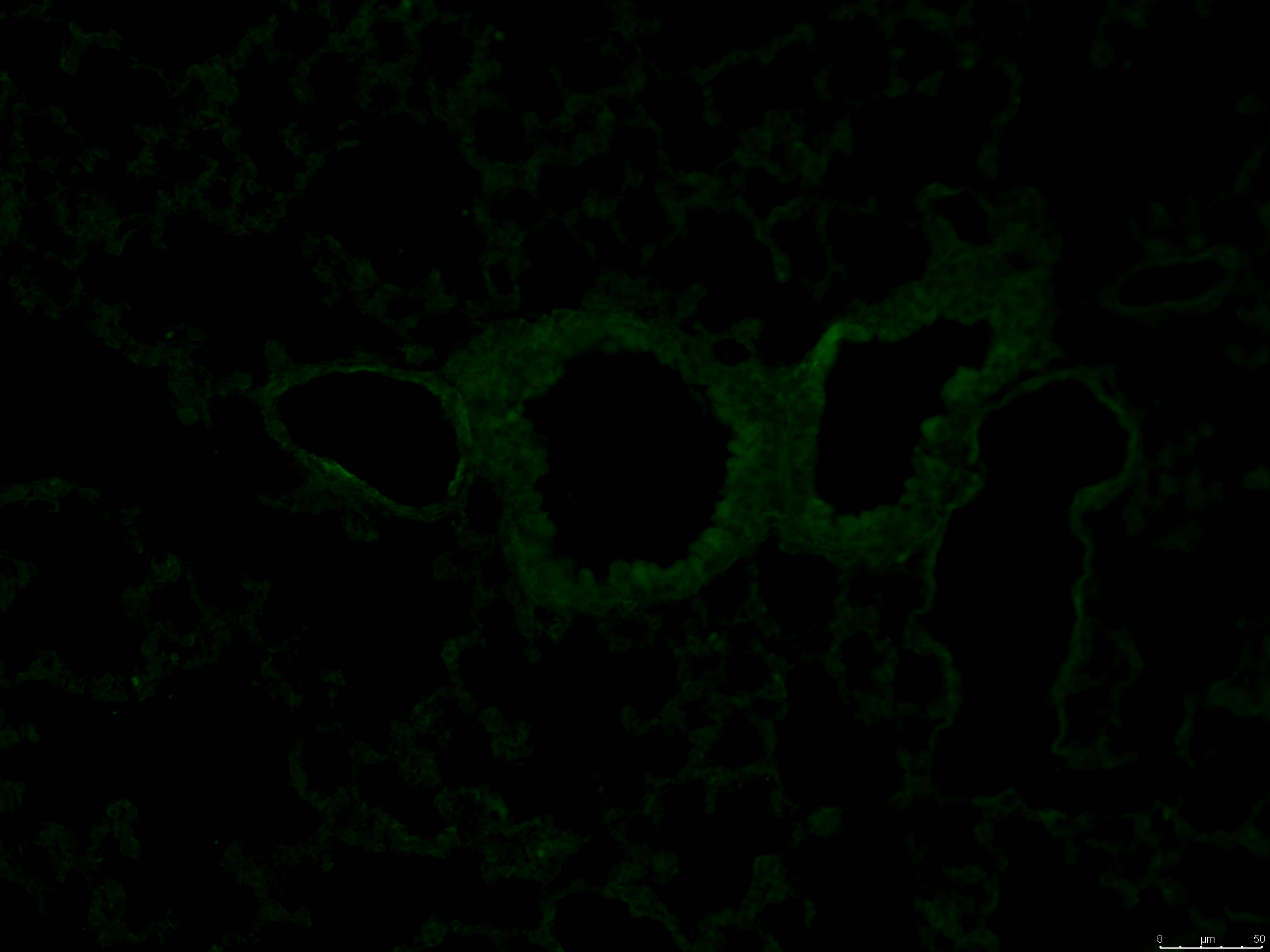

Supplement: Supplementary file 2 [file DataSheet_2.zip › Figure 2 raw datas/B. SDC-1/Control-8weeks 1.tif]

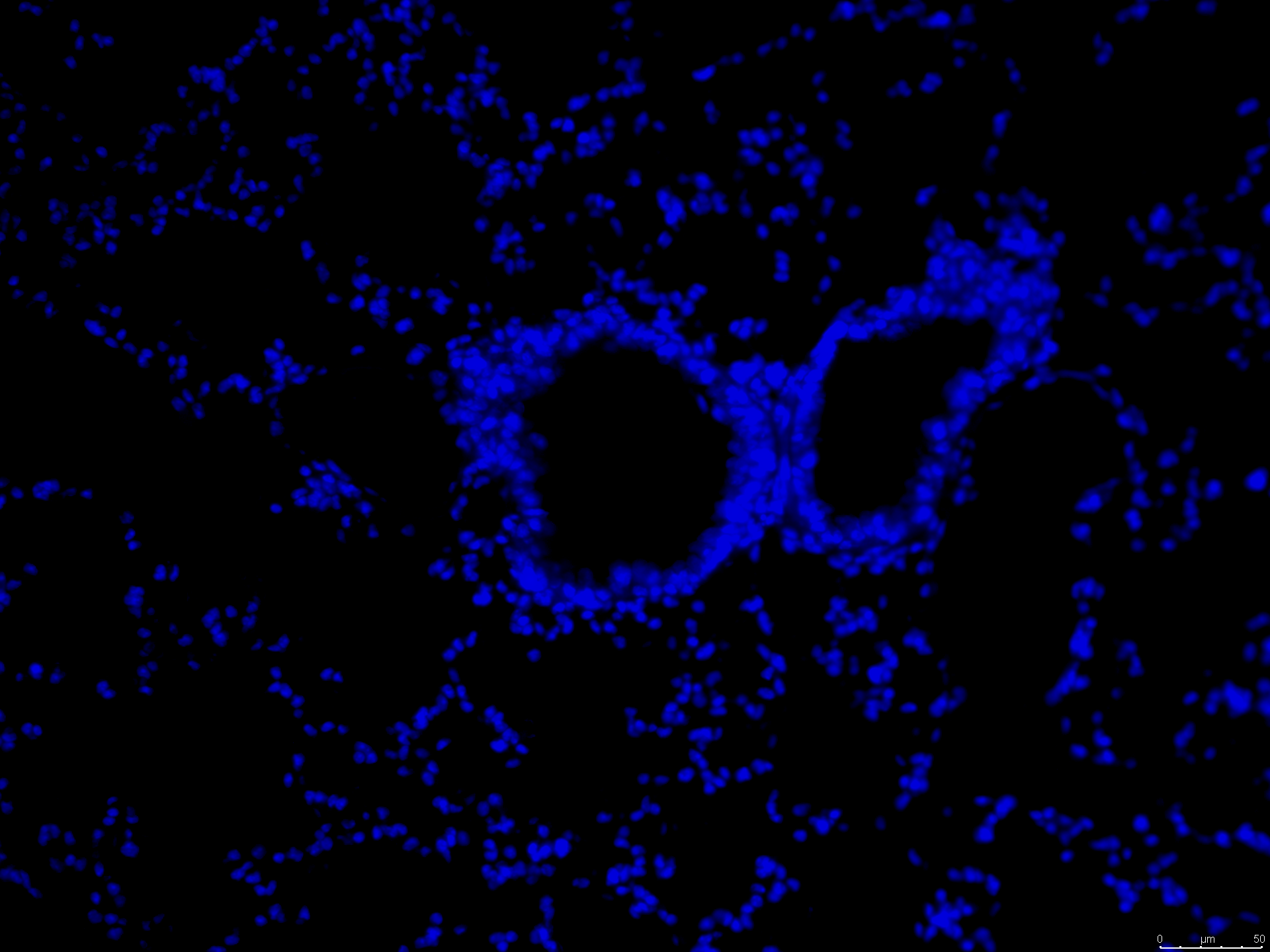

Supplement: Supplementary file 2 [file DataSheet_2.zip › Figure 2 raw datas/B. SDC-1/Control-8weeks 2.tif]

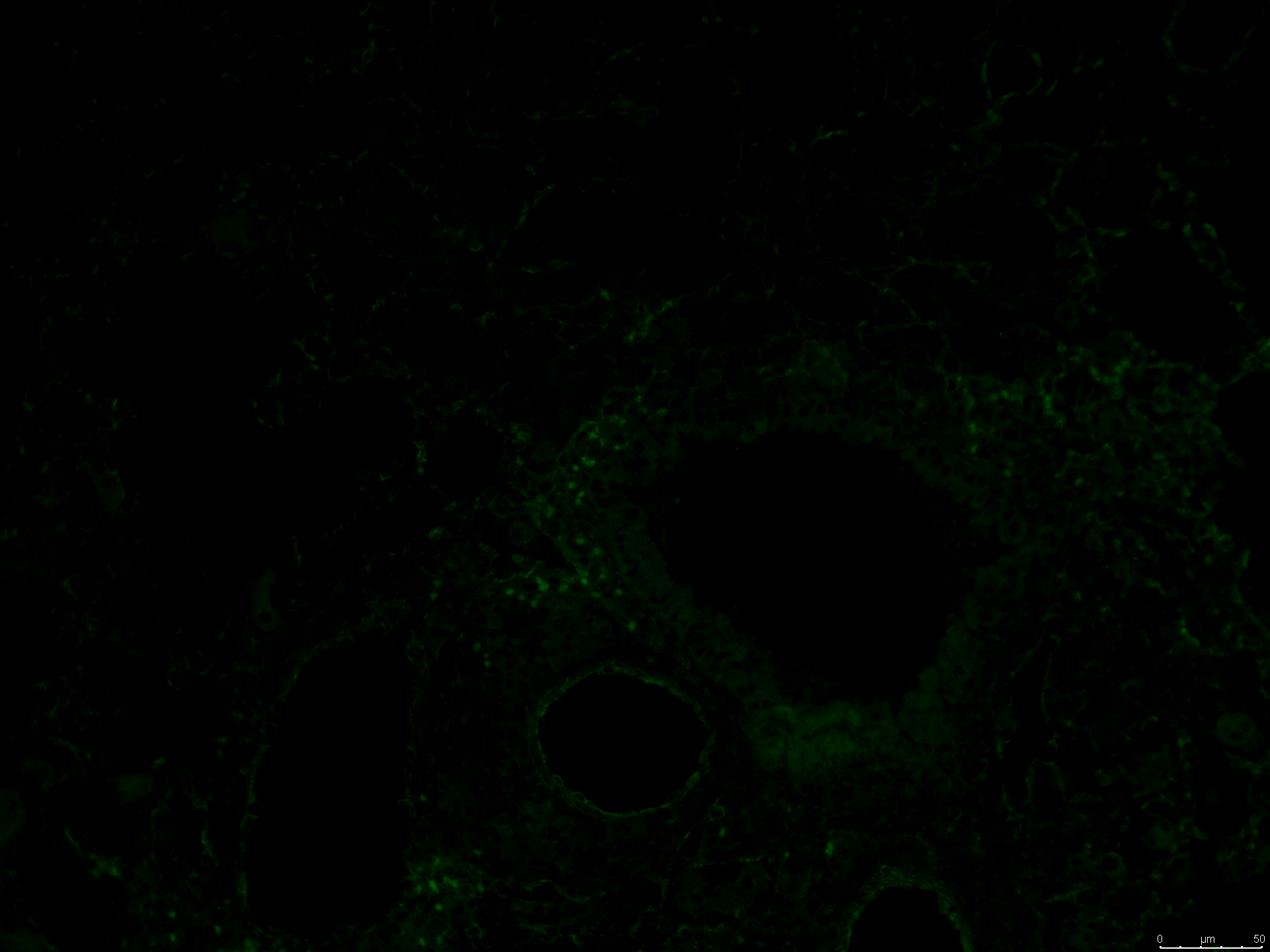

Supplement: Supplementary file 2 [file DataSheet_2.zip › Figure 2 raw datas/B. SDC-1/OVA-4weeks 1.tif]

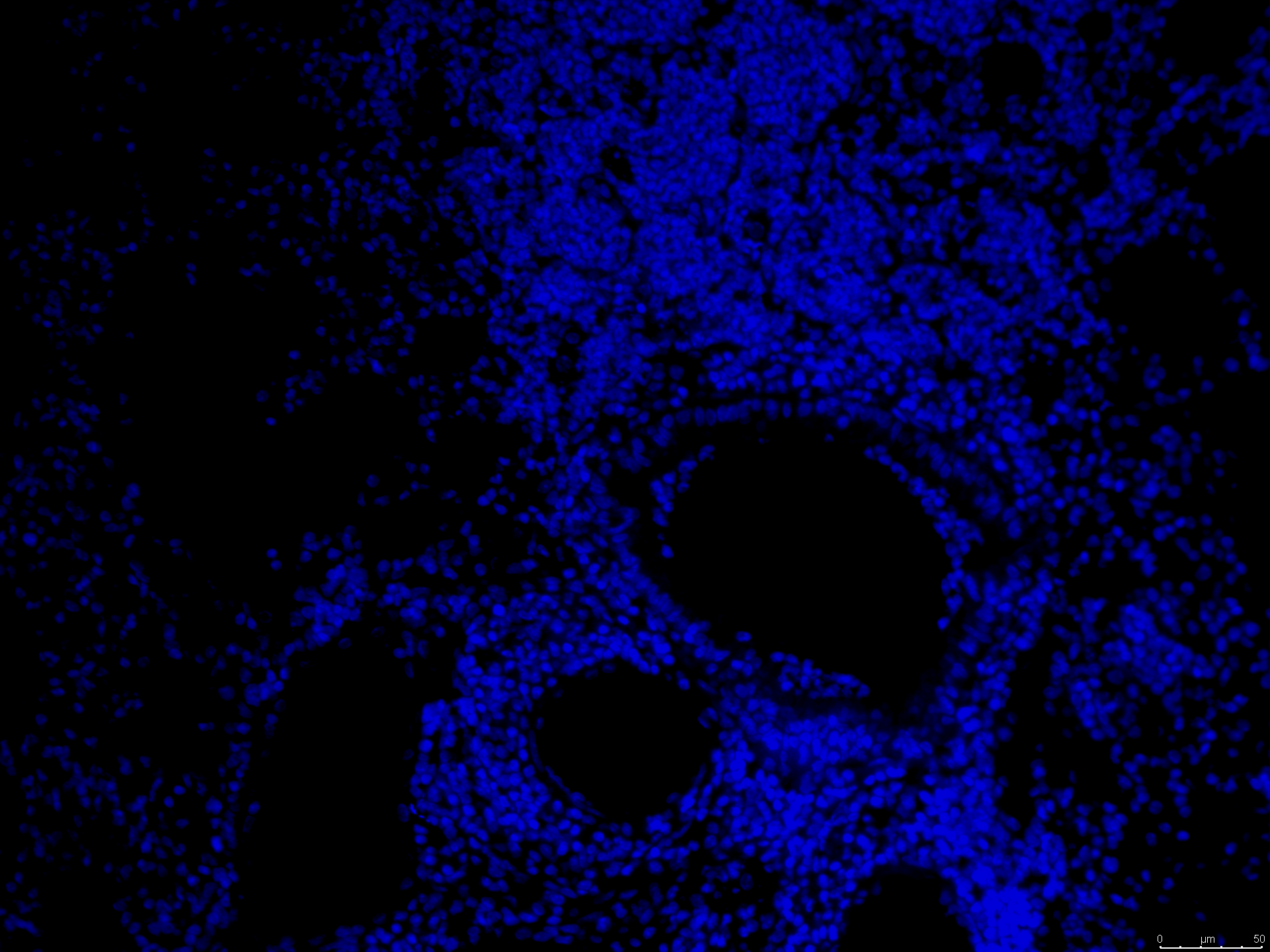

Supplement: Supplementary file 2 [file DataSheet_2.zip › Figure 2 raw datas/B. SDC-1/OVA-4weeks 2.tif]

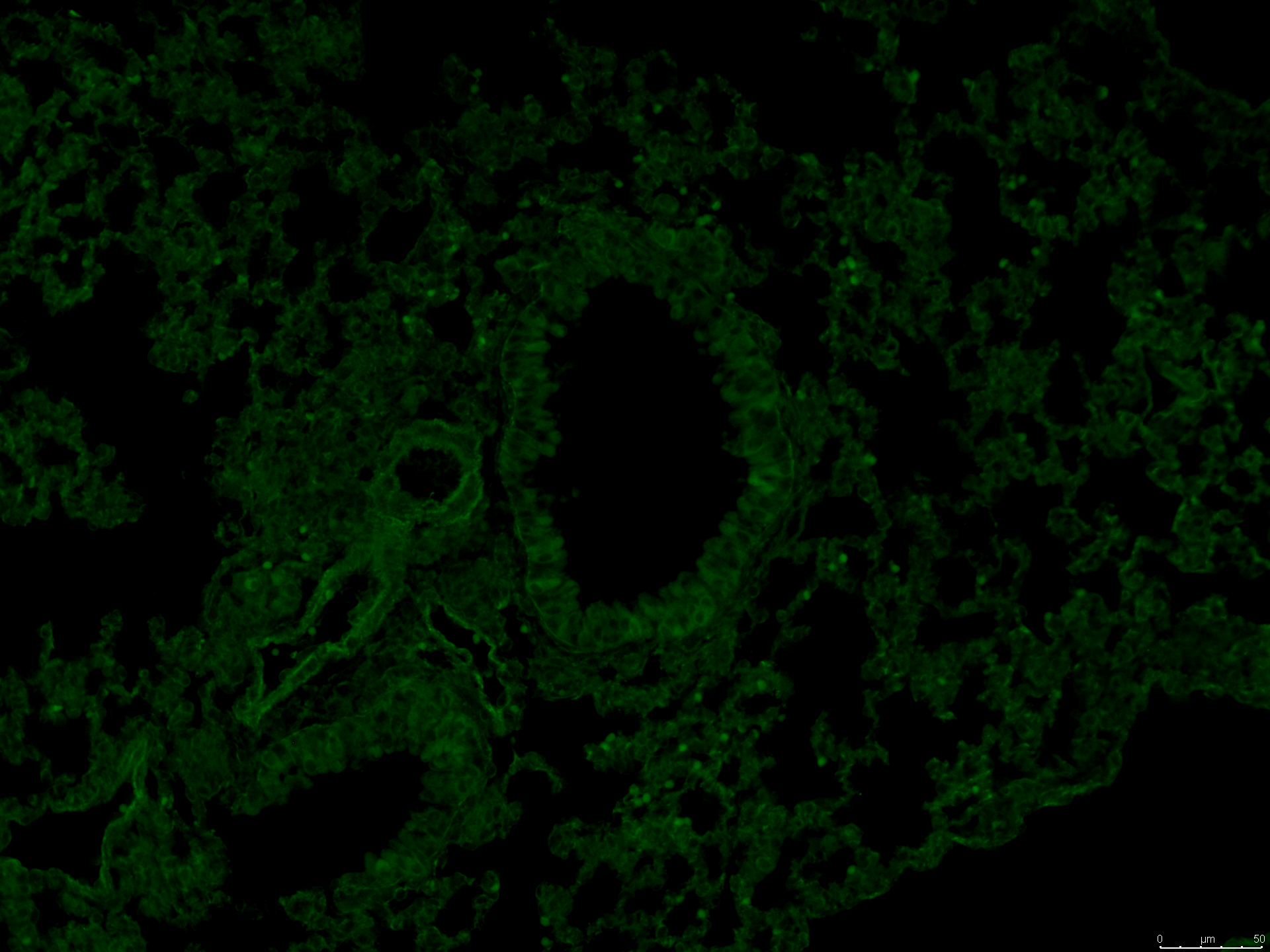

Supplement: Supplementary file 2 [file DataSheet_2.zip › Figure 2 raw datas/B. SDC-1/OVA-8weeks 1.tif]

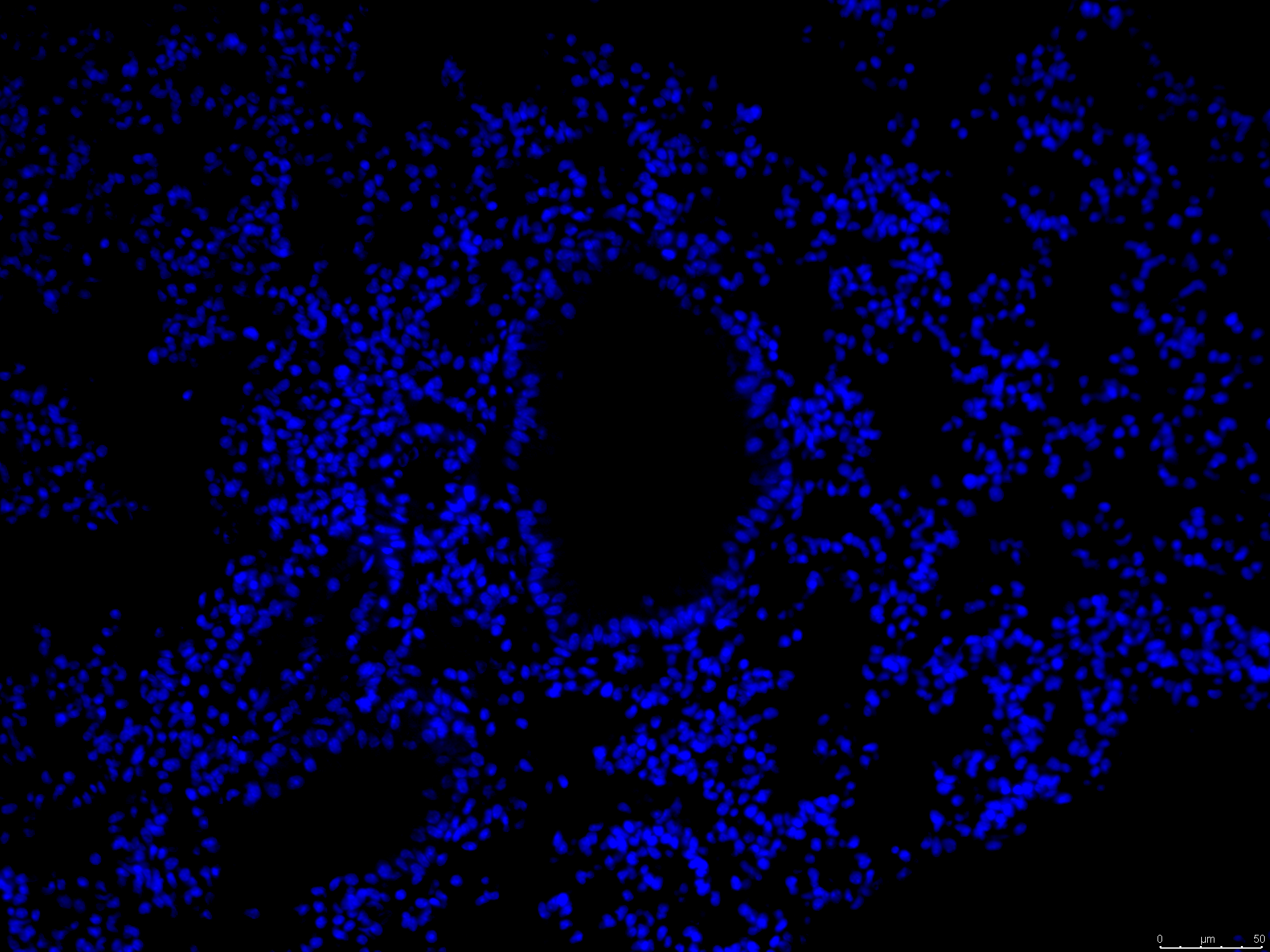

Supplement: Supplementary file 2 [file DataSheet_2.zip › Figure 2 raw datas/B. SDC-1/OVA-8weeks 2.tif]

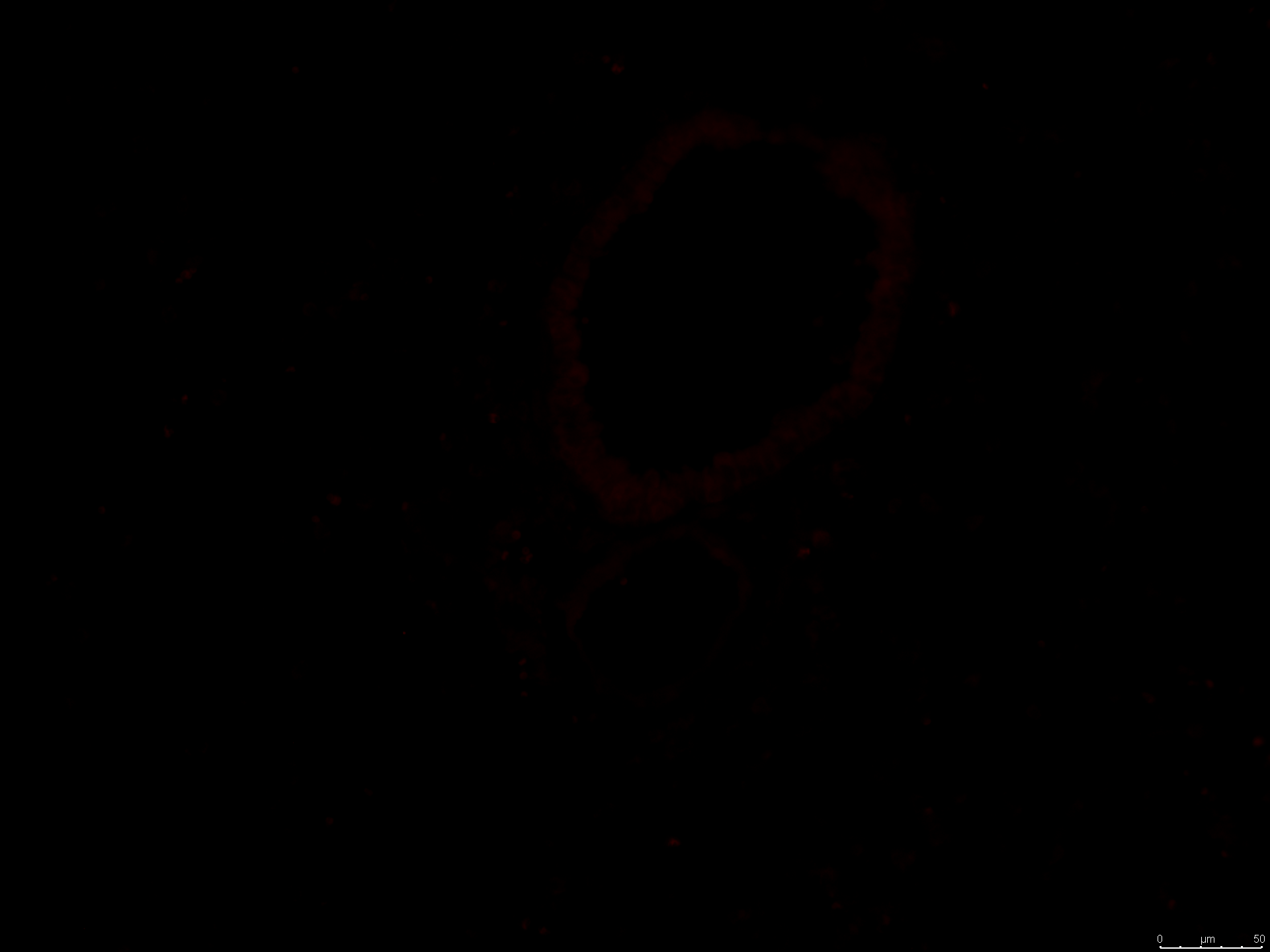

Supplement: Supplementary file 3 [file DataSheet_3.zip › Figure 3 raw datas/B. p-smad3/Control 4weeks 1.tif]

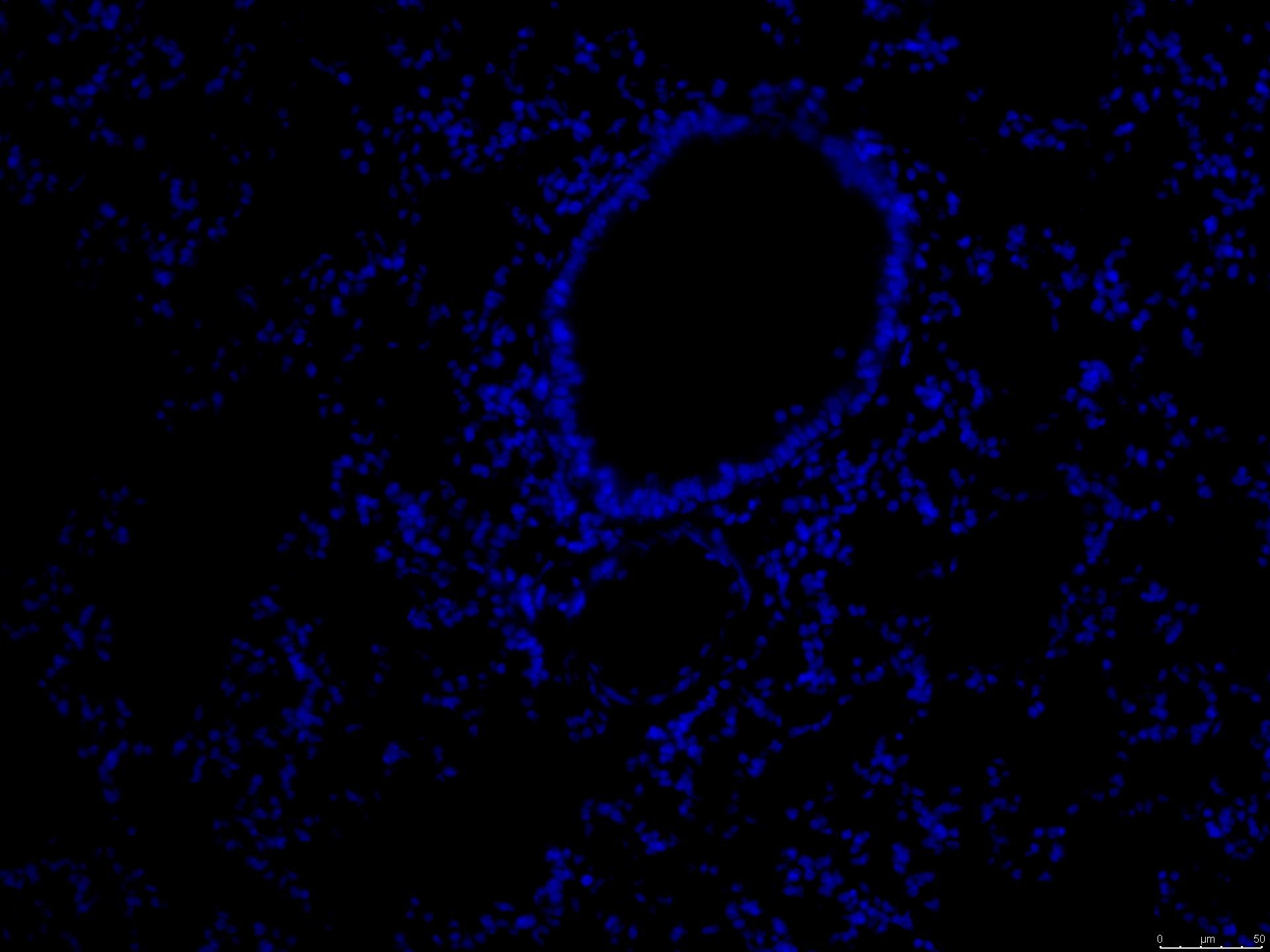

Supplement: Supplementary file 3 [file DataSheet_3.zip › Figure 3 raw datas/B. p-smad3/Control 4weeks 2.tif]

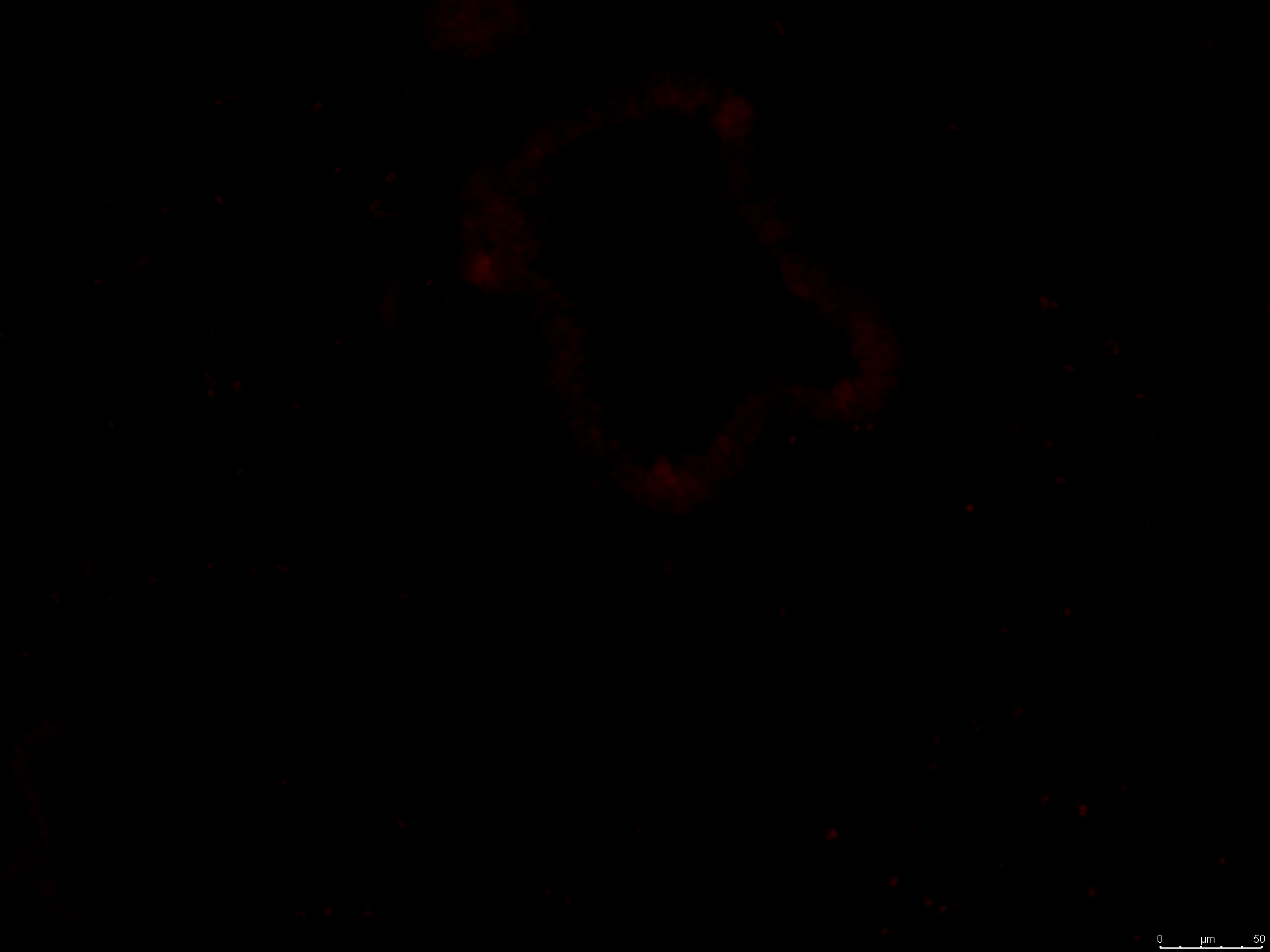

Supplement: Supplementary file 3 [file DataSheet_3.zip › Figure 3 raw datas/B. p-smad3/Control 8weeks 1.tif]

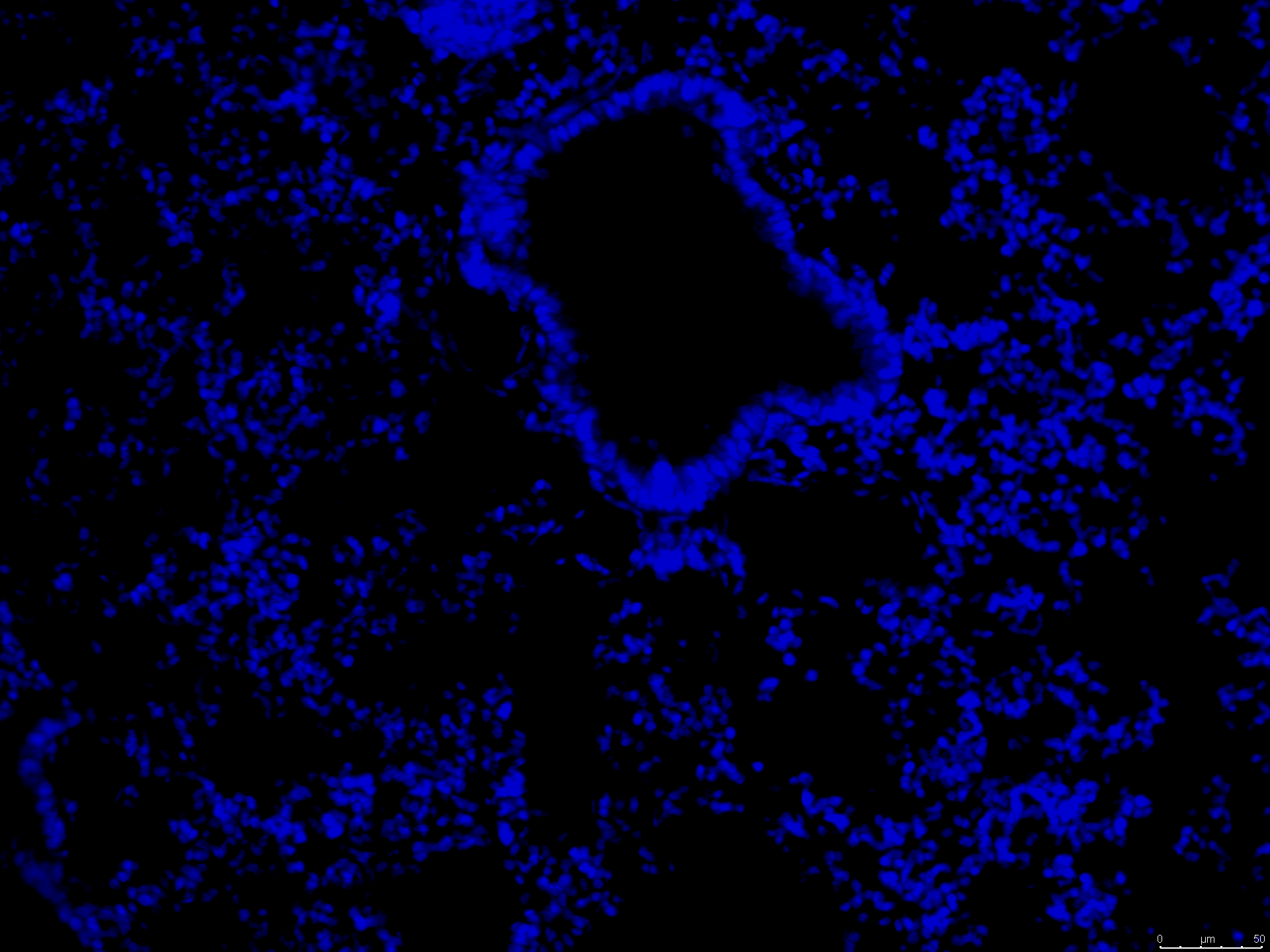

Supplement: Supplementary file 3 [file DataSheet_3.zip › Figure 3 raw datas/B. p-smad3/Control 8weeks 2.tif]

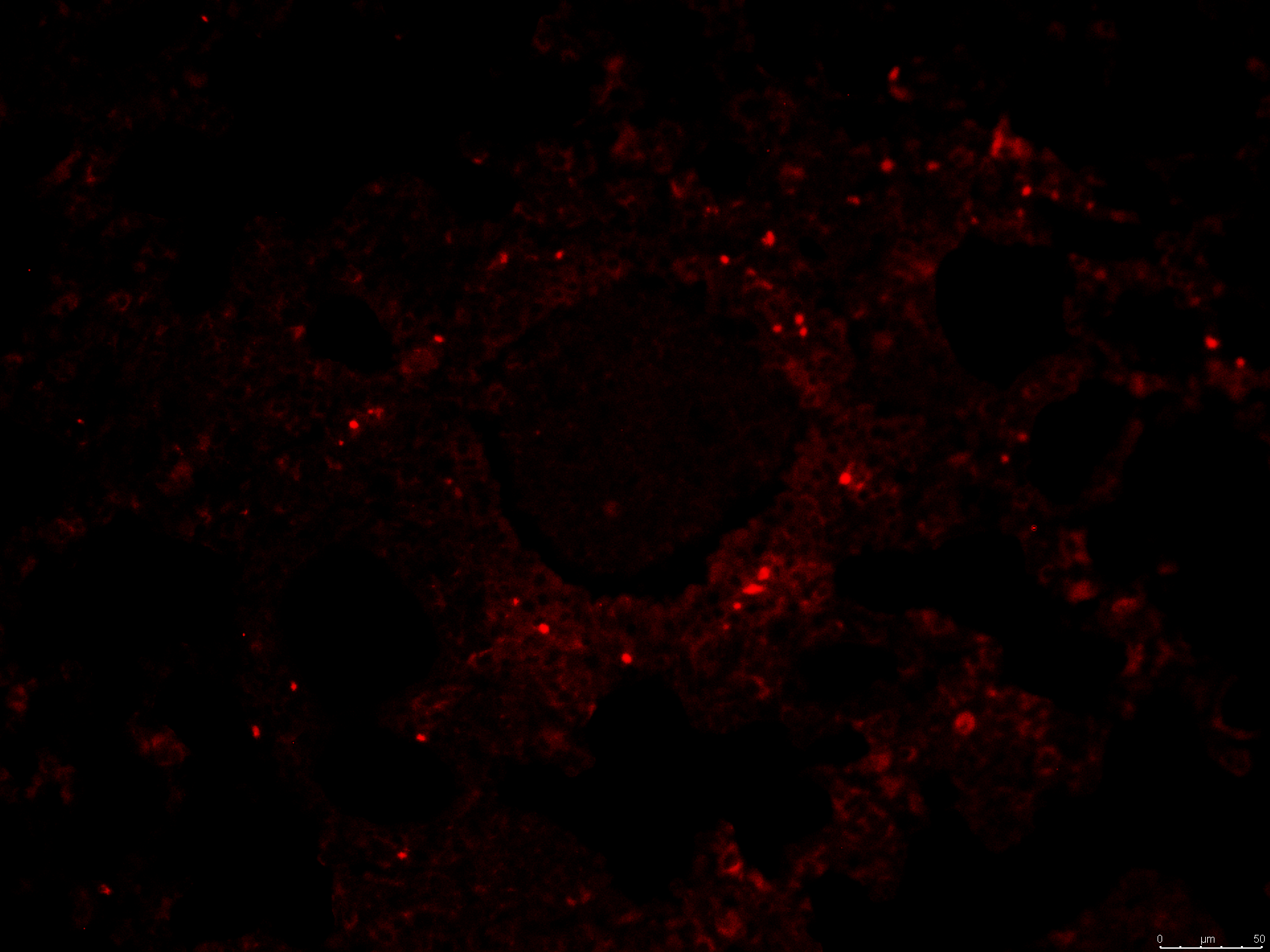

Supplement: Supplementary file 3 [file DataSheet_3.zip › Figure 3 raw datas/B. p-smad3/OVA 4weeks 1.tif]

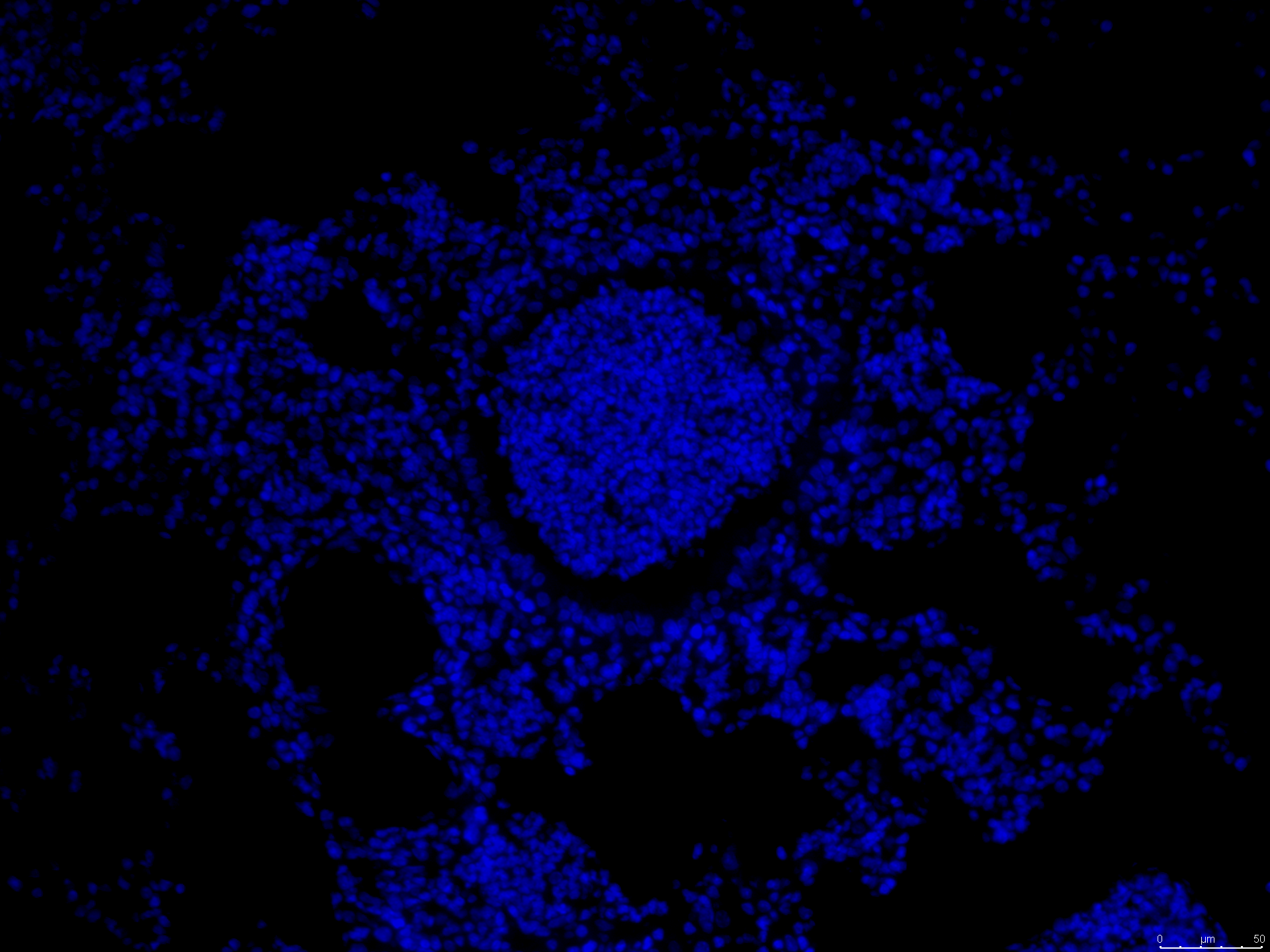

Supplement: Supplementary file 3 [file DataSheet_3.zip › Figure 3 raw datas/B. p-smad3/OVA 4weeks 2.tif]

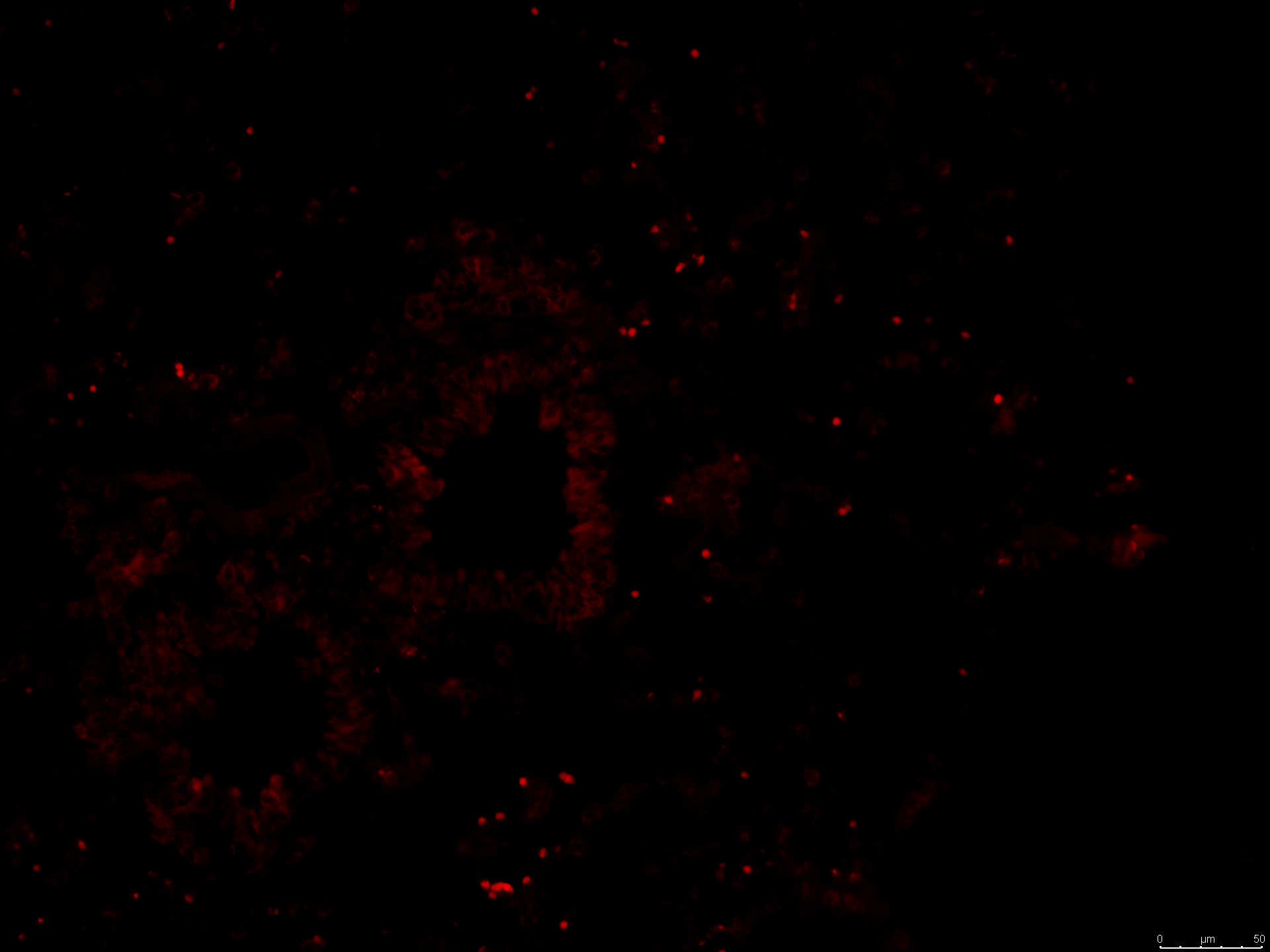

Supplement: Supplementary file 3 [file DataSheet_3.zip › Figure 3 raw datas/B. p-smad3/OVA 8weeks 1.tif]

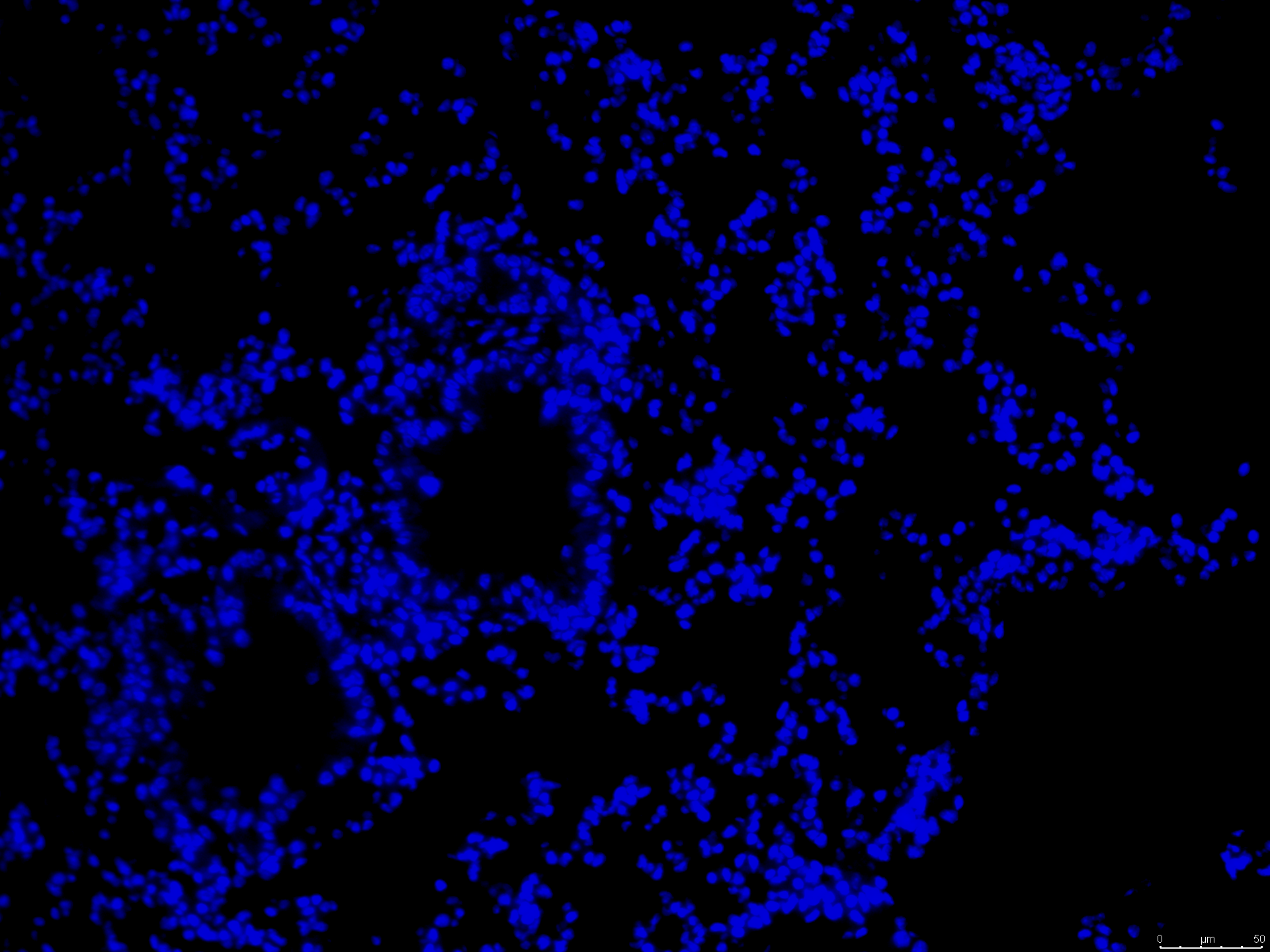

Supplement: Supplementary file 3 [file DataSheet_3.zip › Figure 3 raw datas/B. p-smad3/OVA 8weeks 2.tif]

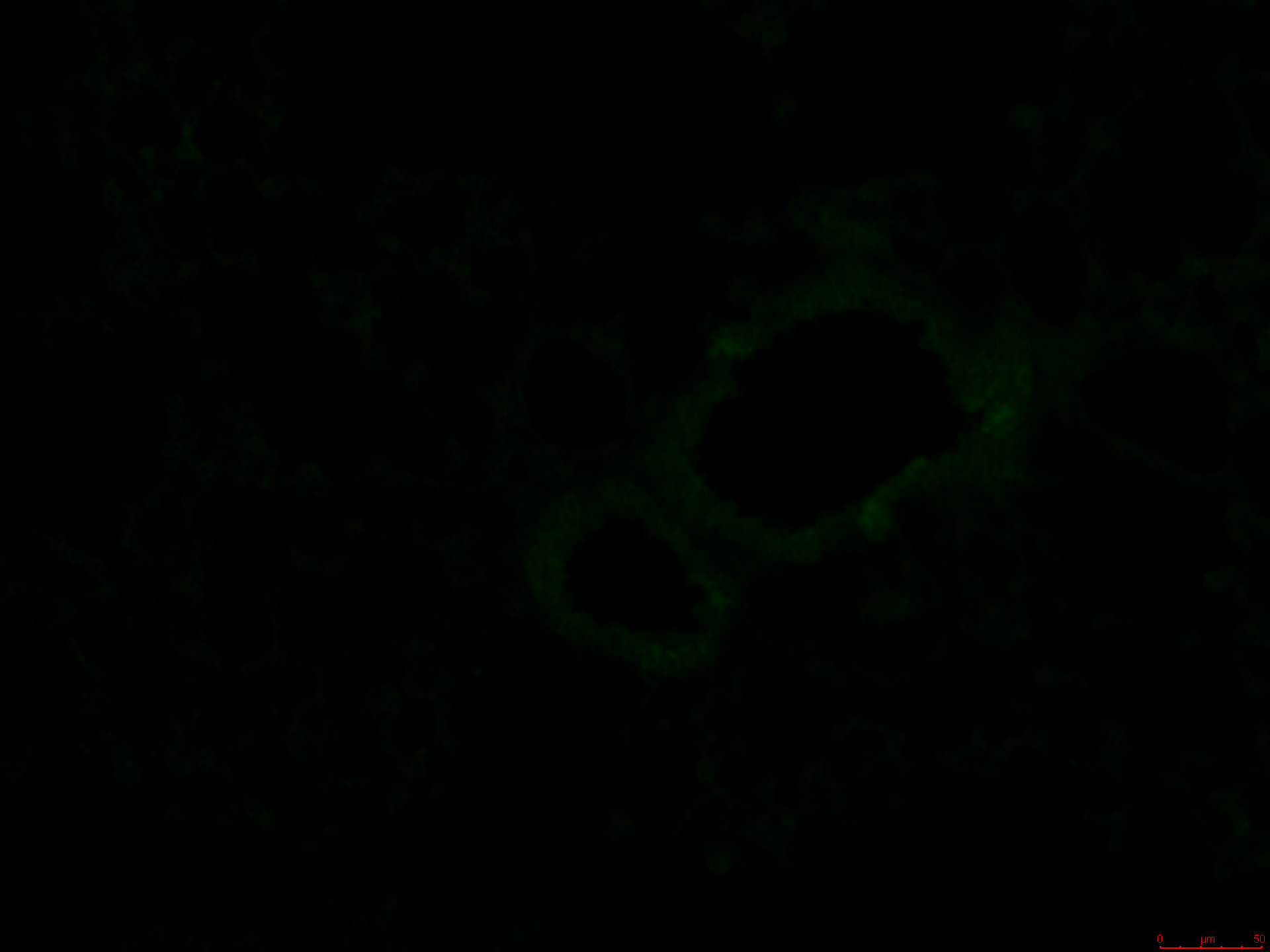

Supplement: Supplementary file 3 [file DataSheet_3.zip › Figure 3 raw datas/C. collagen I/Control 4weeks 1.tif]

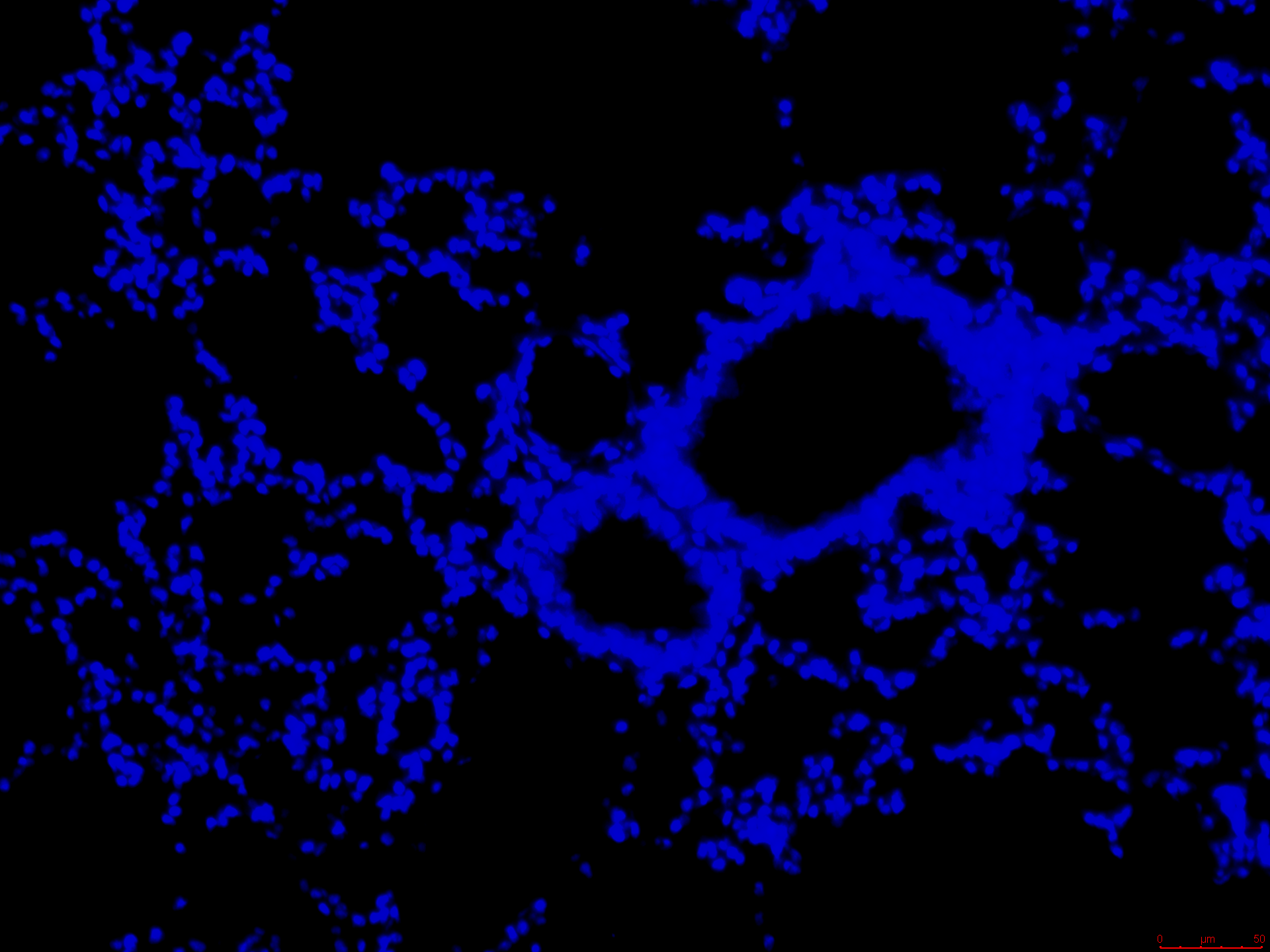

Supplement: Supplementary file 3 [file DataSheet_3.zip › Figure 3 raw datas/C. collagen I/Control 4weeks 2.tif]

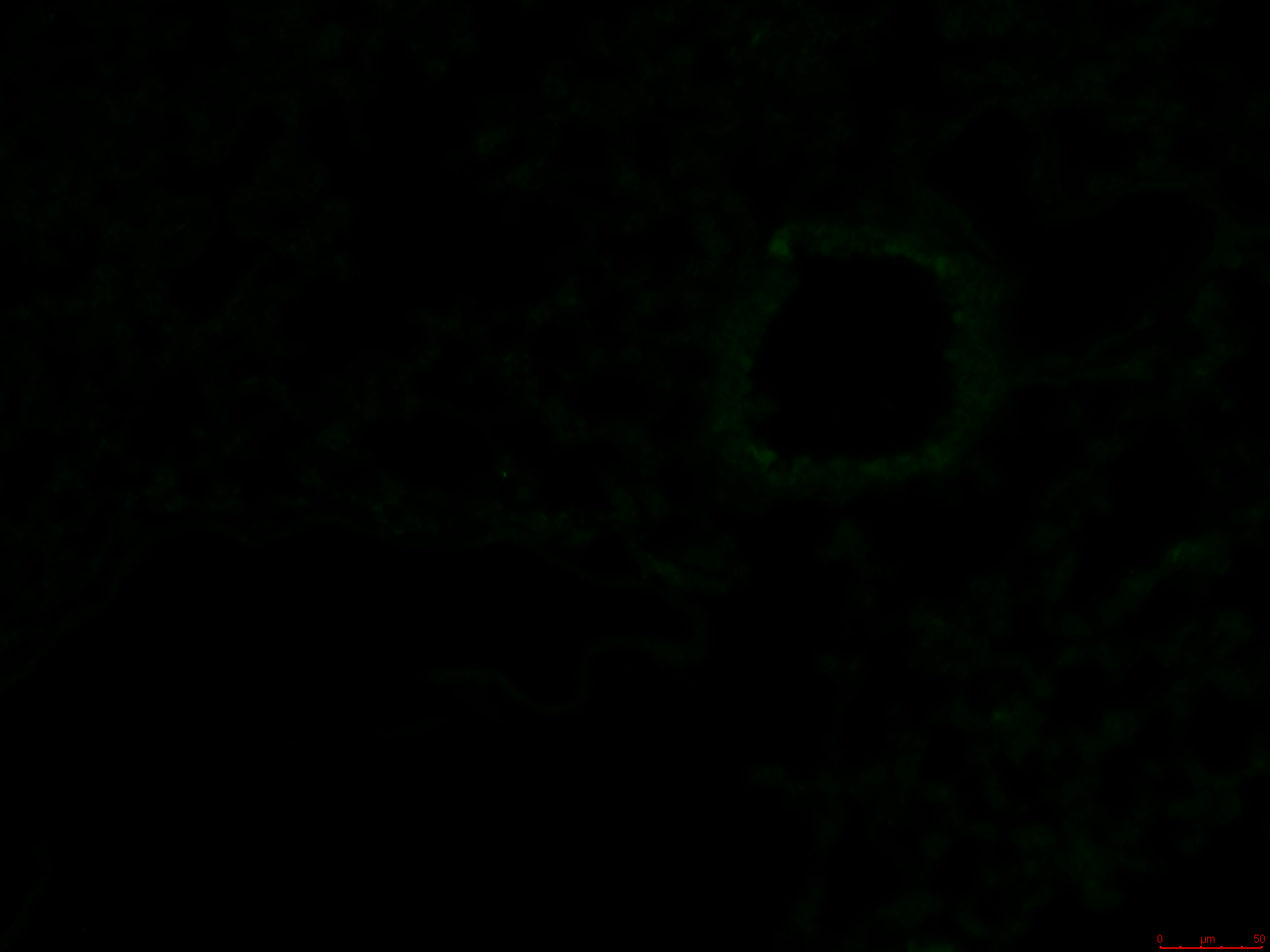

Supplement: Supplementary file 3 [file DataSheet_3.zip › Figure 3 raw datas/C. collagen I/Control 8weeks 1.tif]

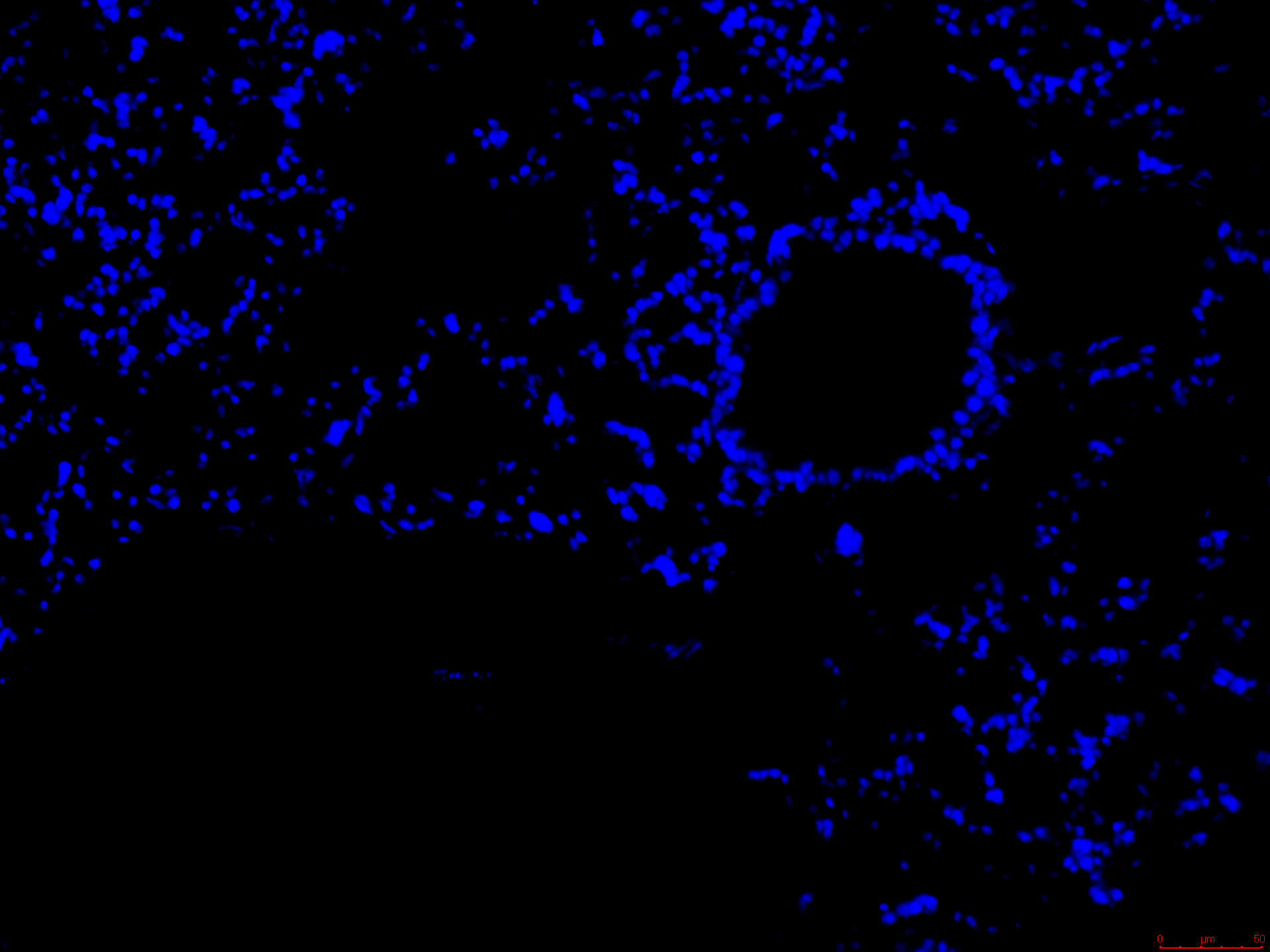

Supplement: Supplementary file 3 [file DataSheet_3.zip › Figure 3 raw datas/C. collagen I/Control 8weeks 2.tif]

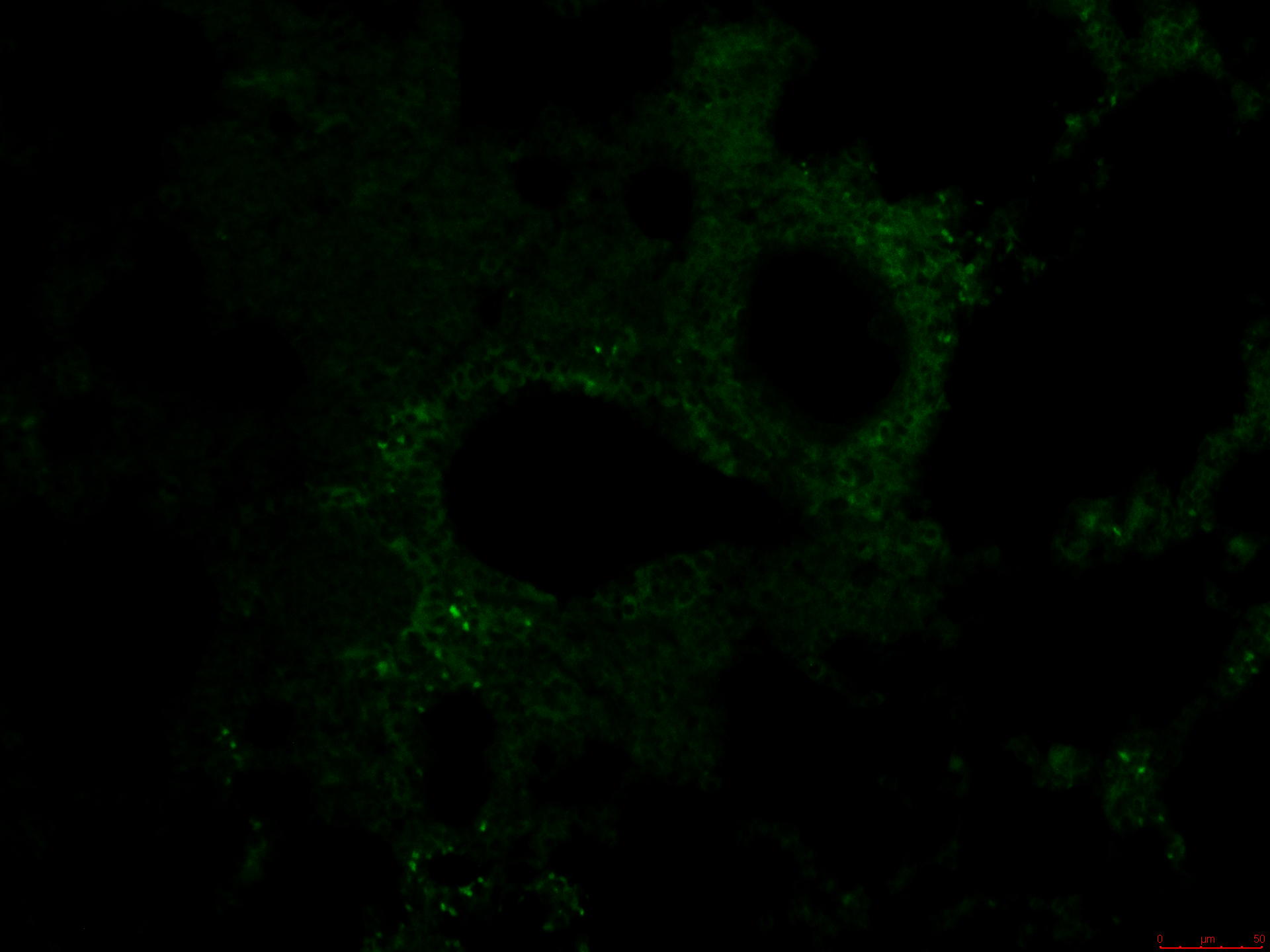

Supplement: Supplementary file 3 [file DataSheet_3.zip › Figure 3 raw datas/C. collagen I/OVA 4weeks 1.tif]

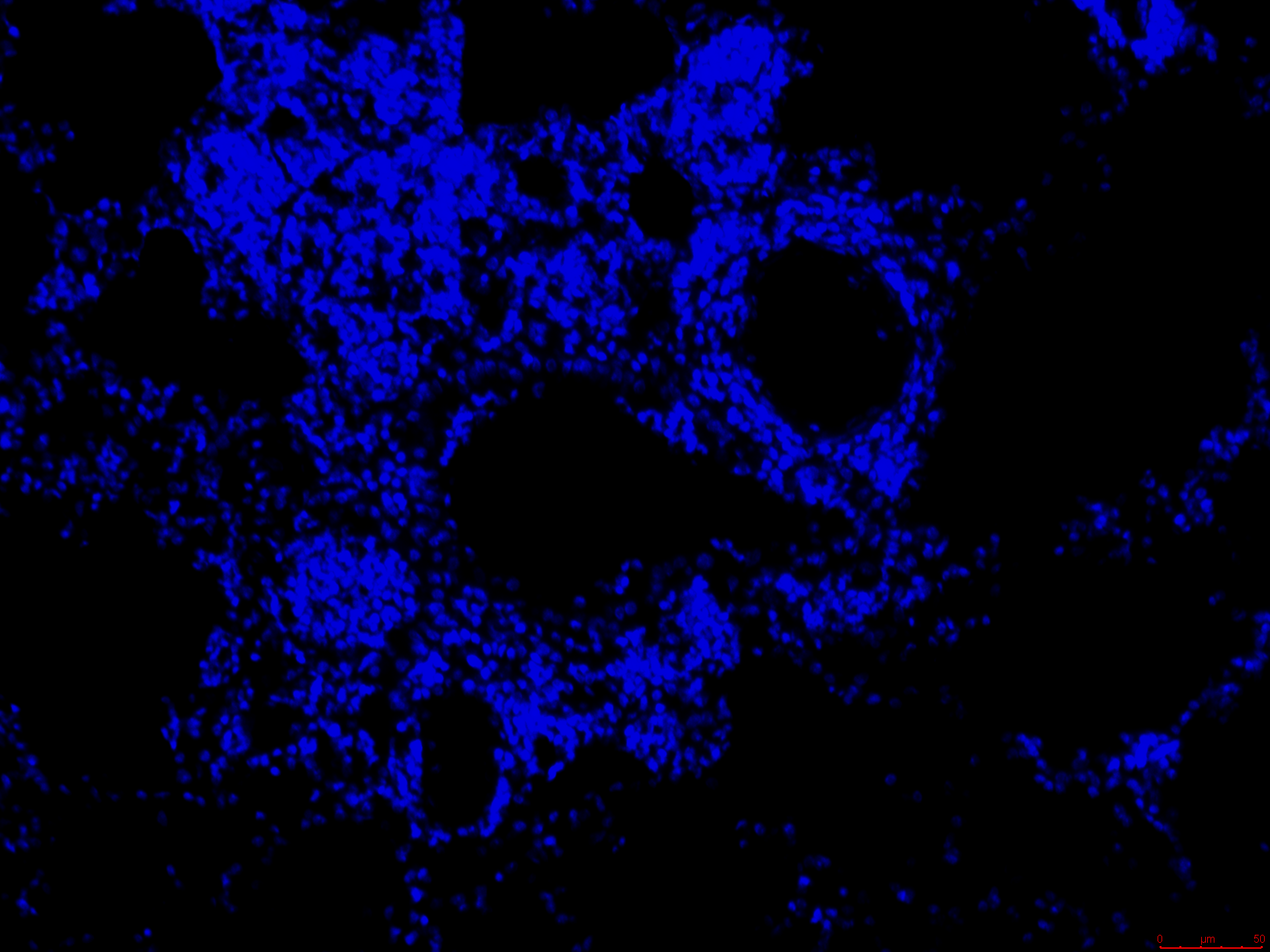

Supplement: Supplementary file 3 [file DataSheet_3.zip › Figure 3 raw datas/C. collagen I/OVA 4weeks 2.tif]

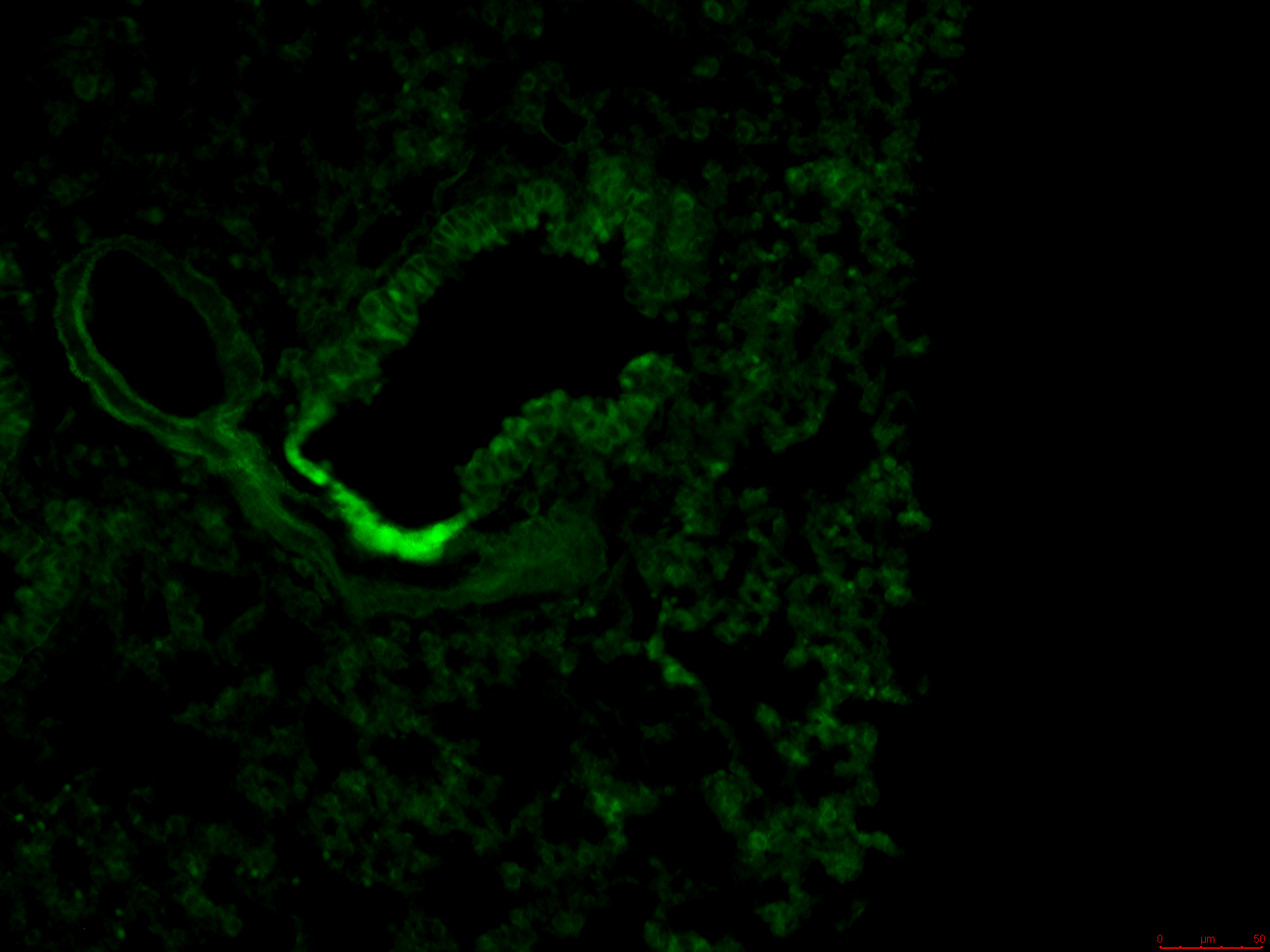

Supplement: Supplementary file 3 [file DataSheet_3.zip › Figure 3 raw datas/C. collagen I/OVA 8weeks 1.tif]

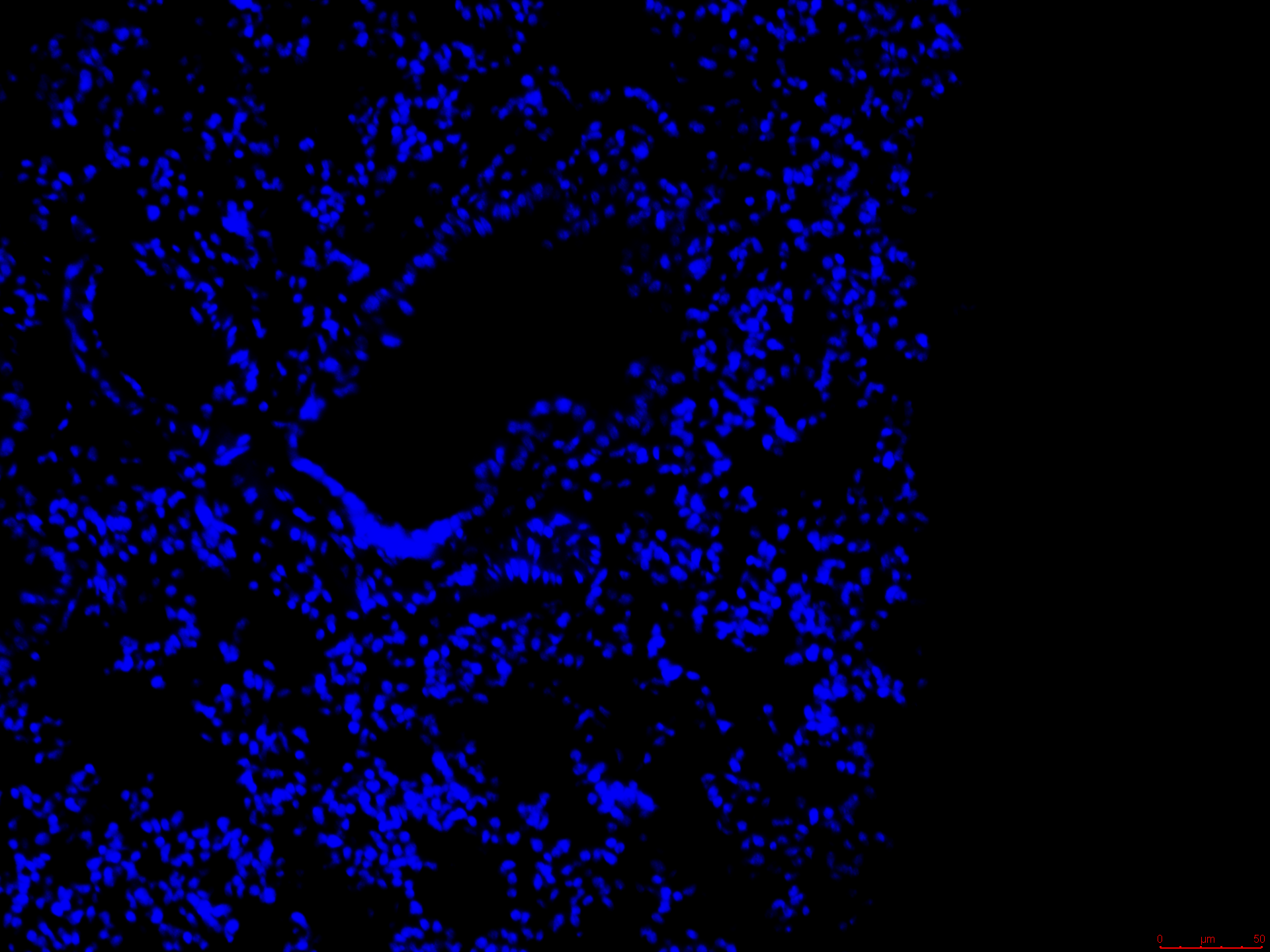

Supplement: Supplementary file 3 [file DataSheet_3.zip › Figure 3 raw datas/C. collagen I/OVA 8weeks 2.tif]

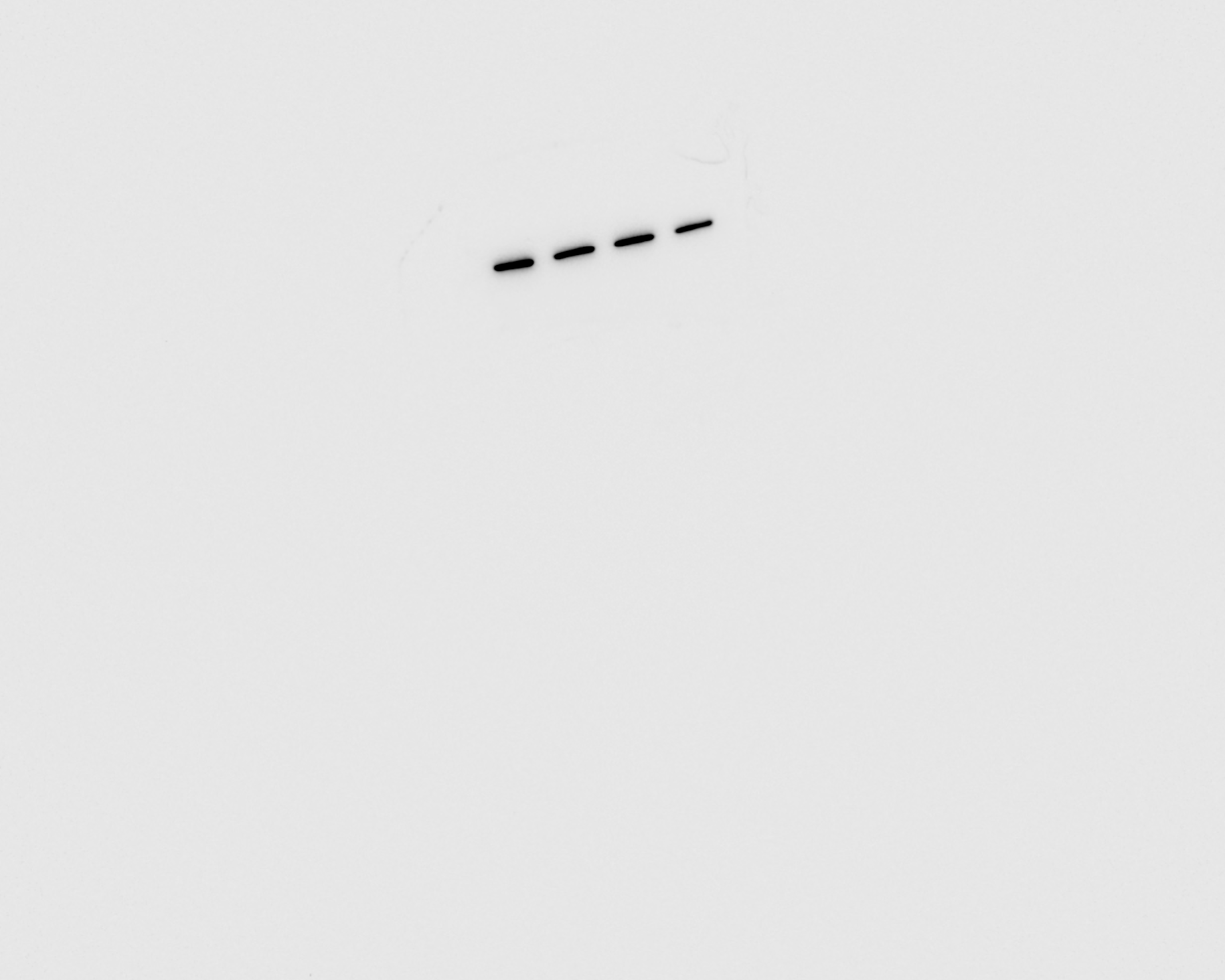

Supplement: Supplementary file 4 [file DataSheet_4.zip › Figure 4 raw datas/D E F/D. GAPDH.tif]

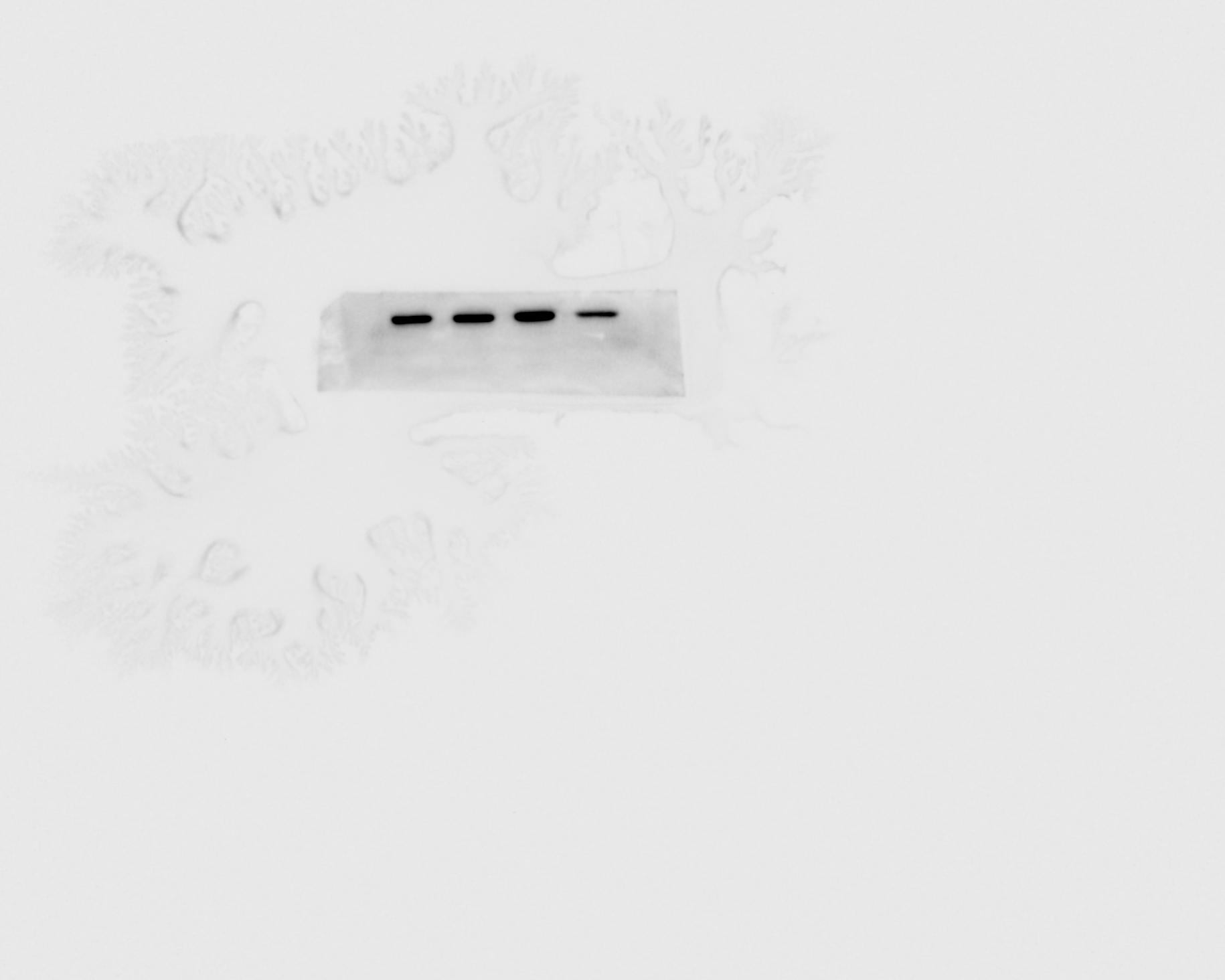

Supplement: Supplementary file 4 [file DataSheet_4.zip › Figure 4 raw datas/D E F/D. SDC-1.tif]

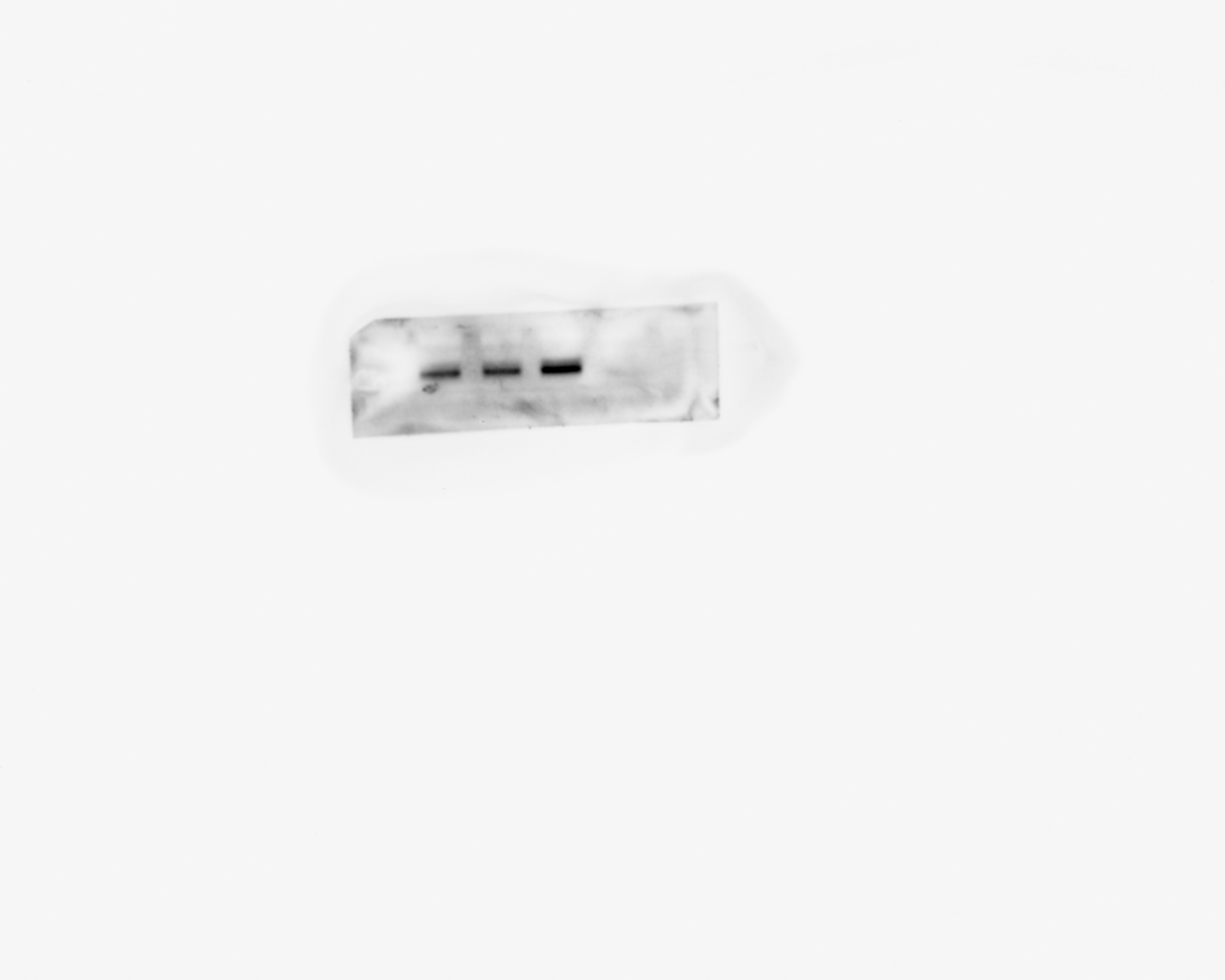

Supplement: Supplementary file 4 [file DataSheet_4.zip › Figure 4 raw datas/D E F/E. p-smad3.tif]

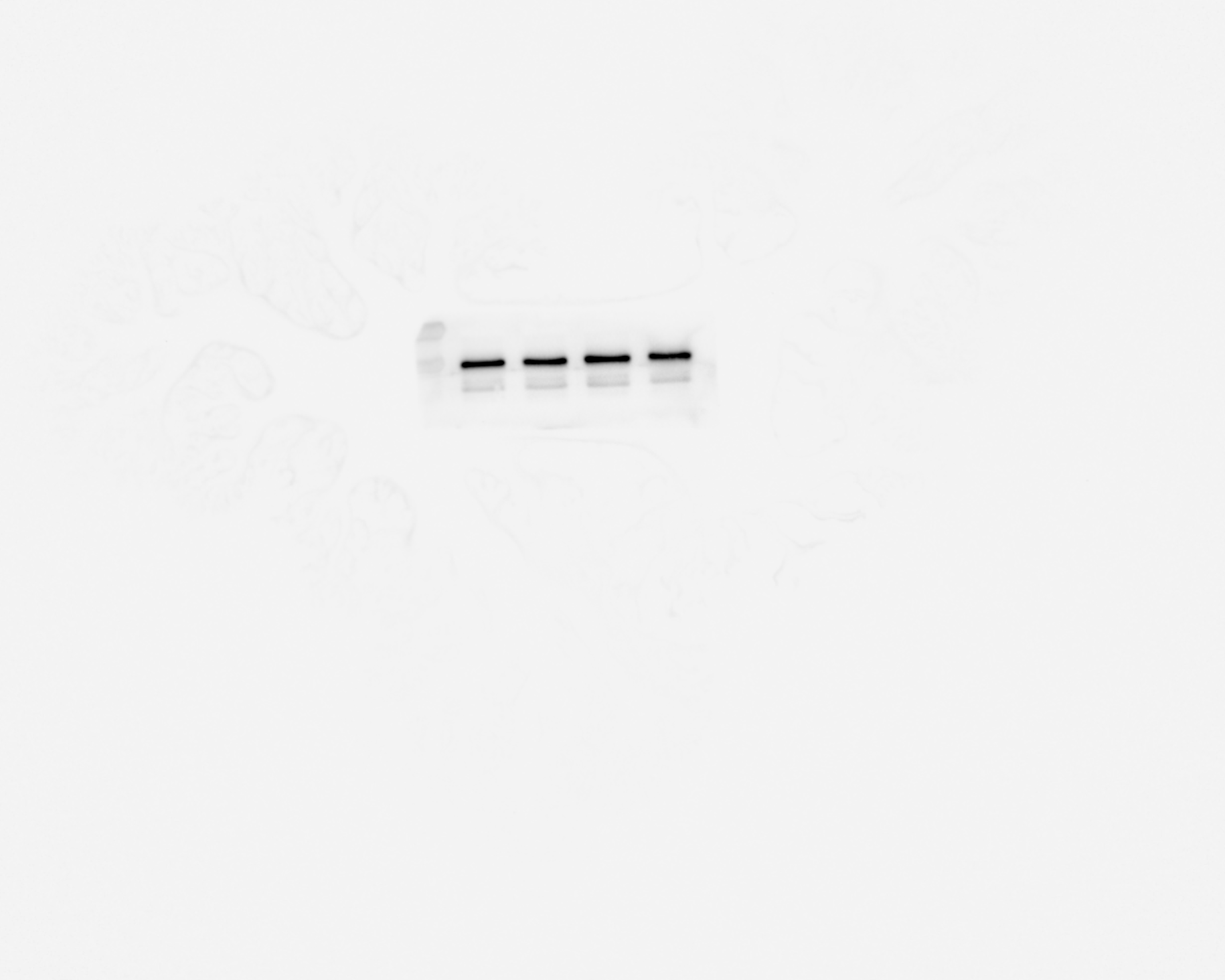

Supplement: Supplementary file 4 [file DataSheet_4.zip › Figure 4 raw datas/D E F/E. sma3.tif]

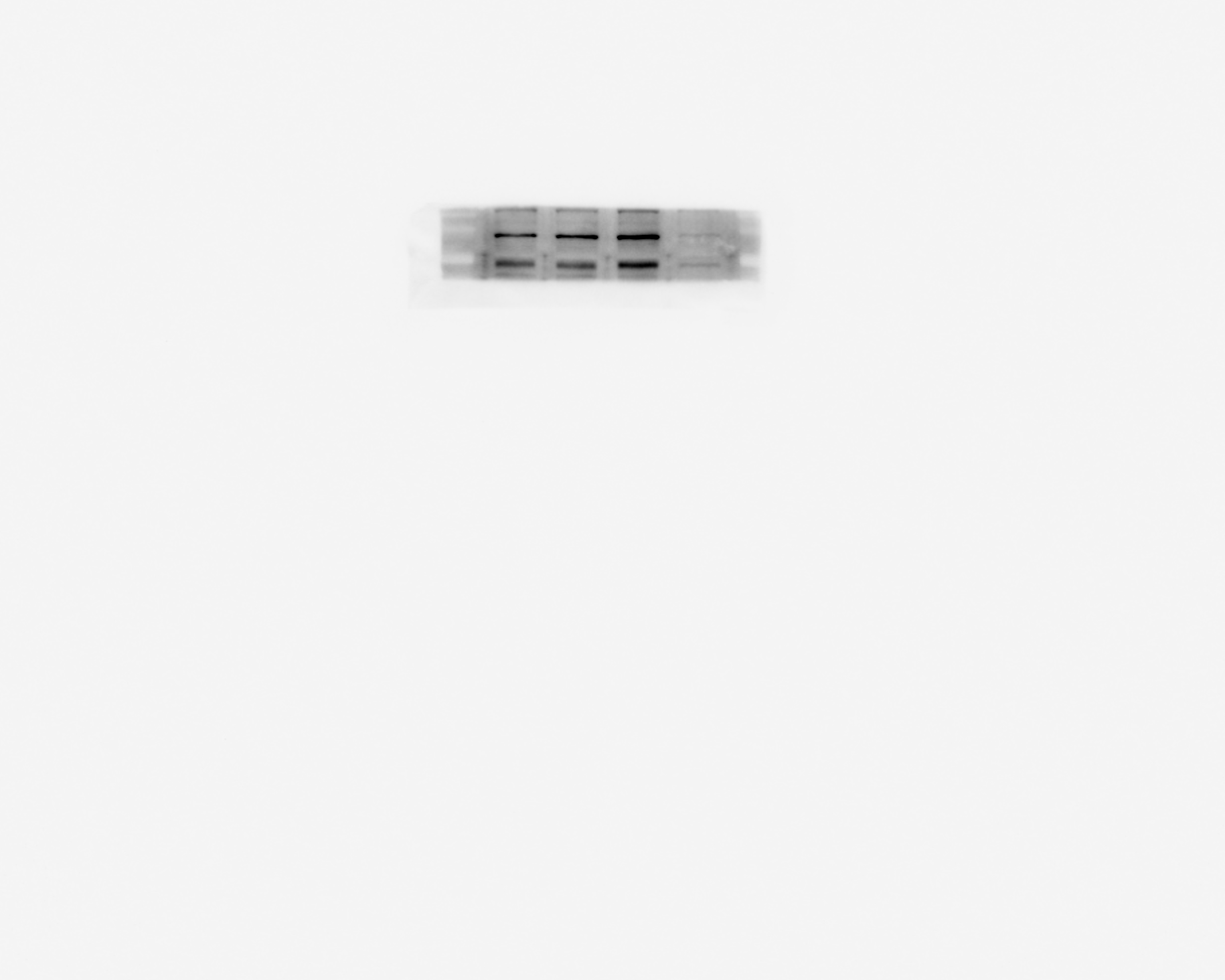

Supplement: Supplementary file 4 [file DataSheet_4.zip › Figure 4 raw datas/D E F/F. Collagen I.tif]

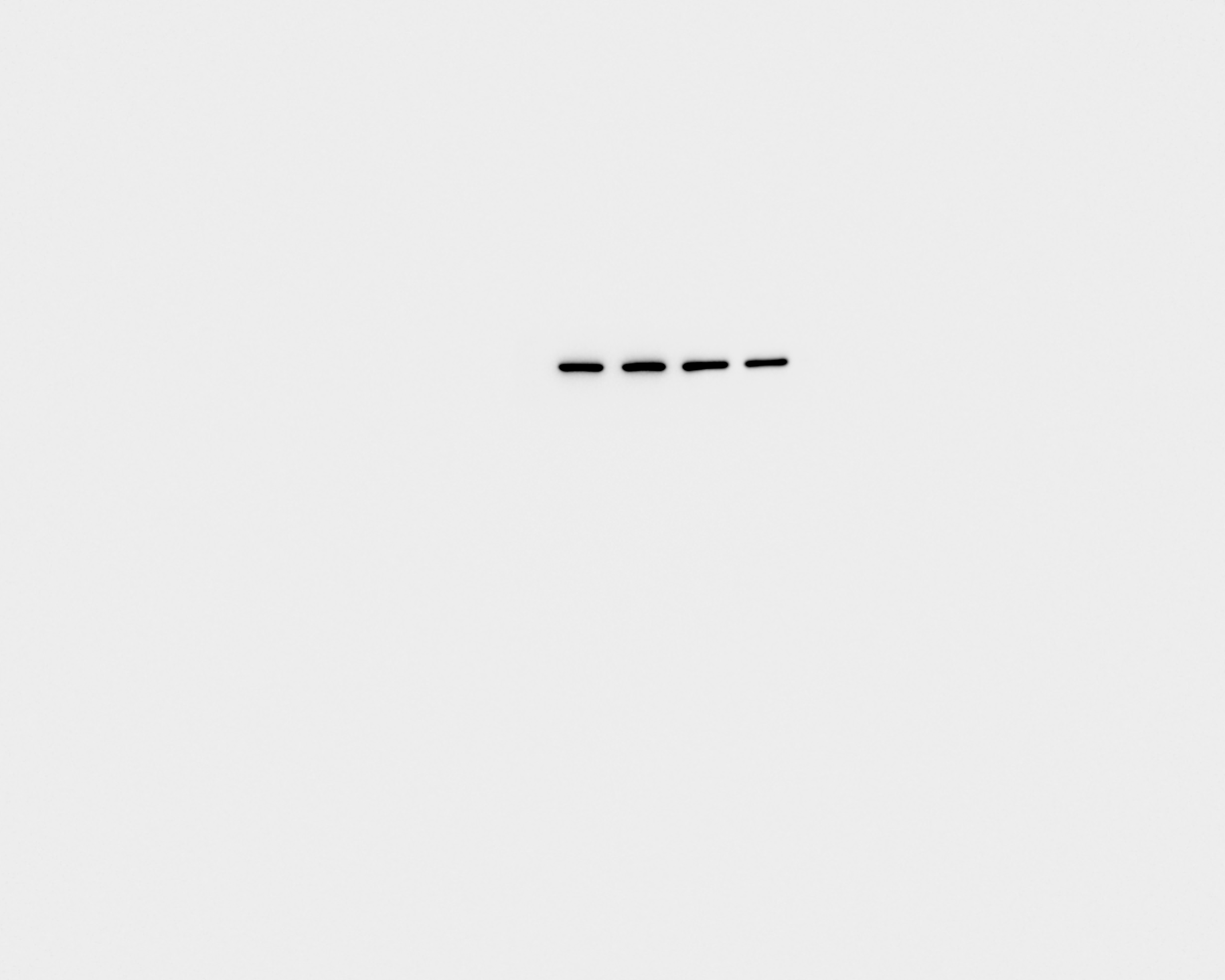

Supplement: Supplementary file 4 [file DataSheet_4.zip › Figure 4 raw datas/D E F/F. GAPDH.tif]

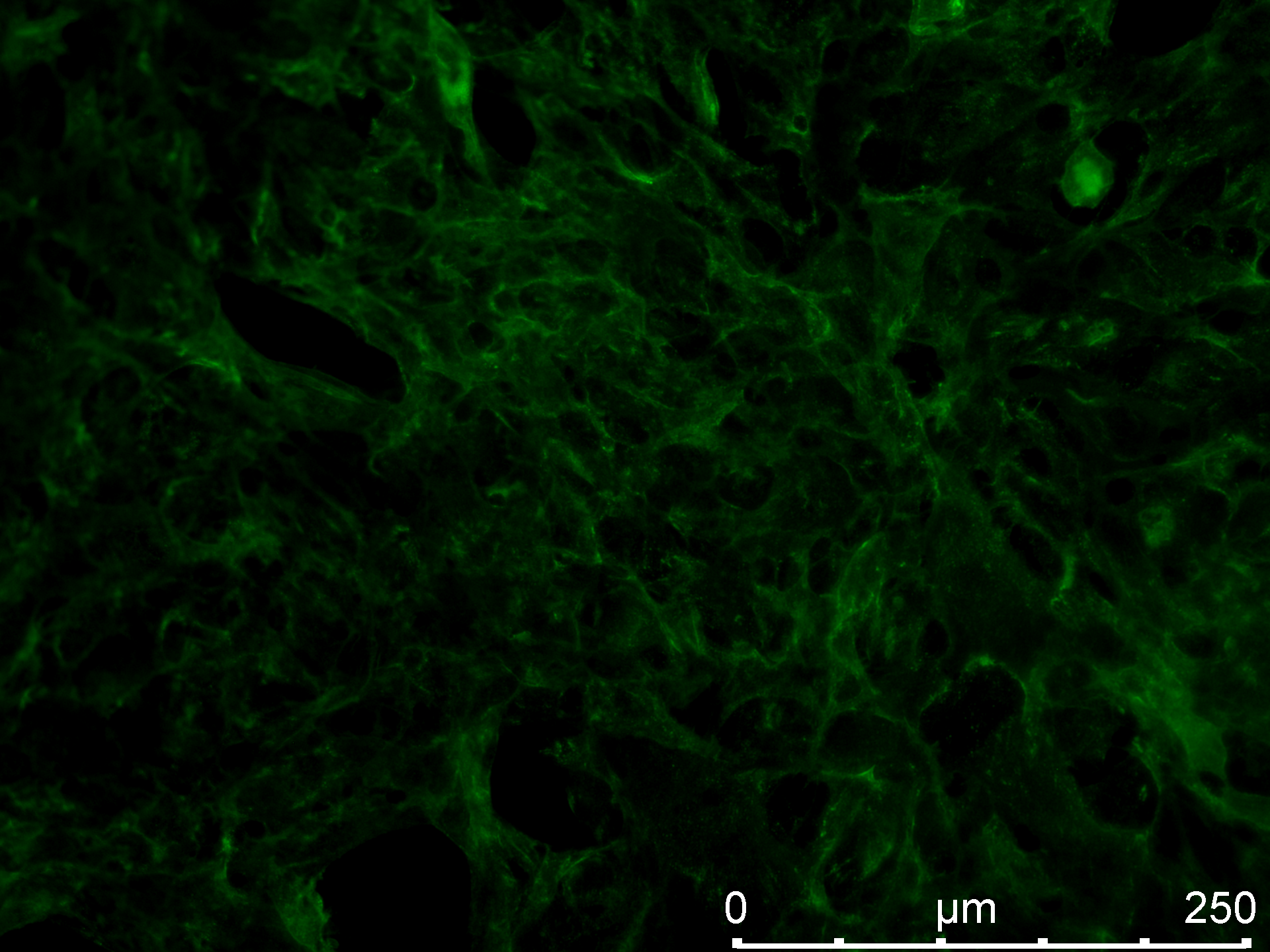

Supplement: Supplementary file 4 [file DataSheet_4.zip › Figure 4 raw datas/M/NC-siRNA 1.tif]

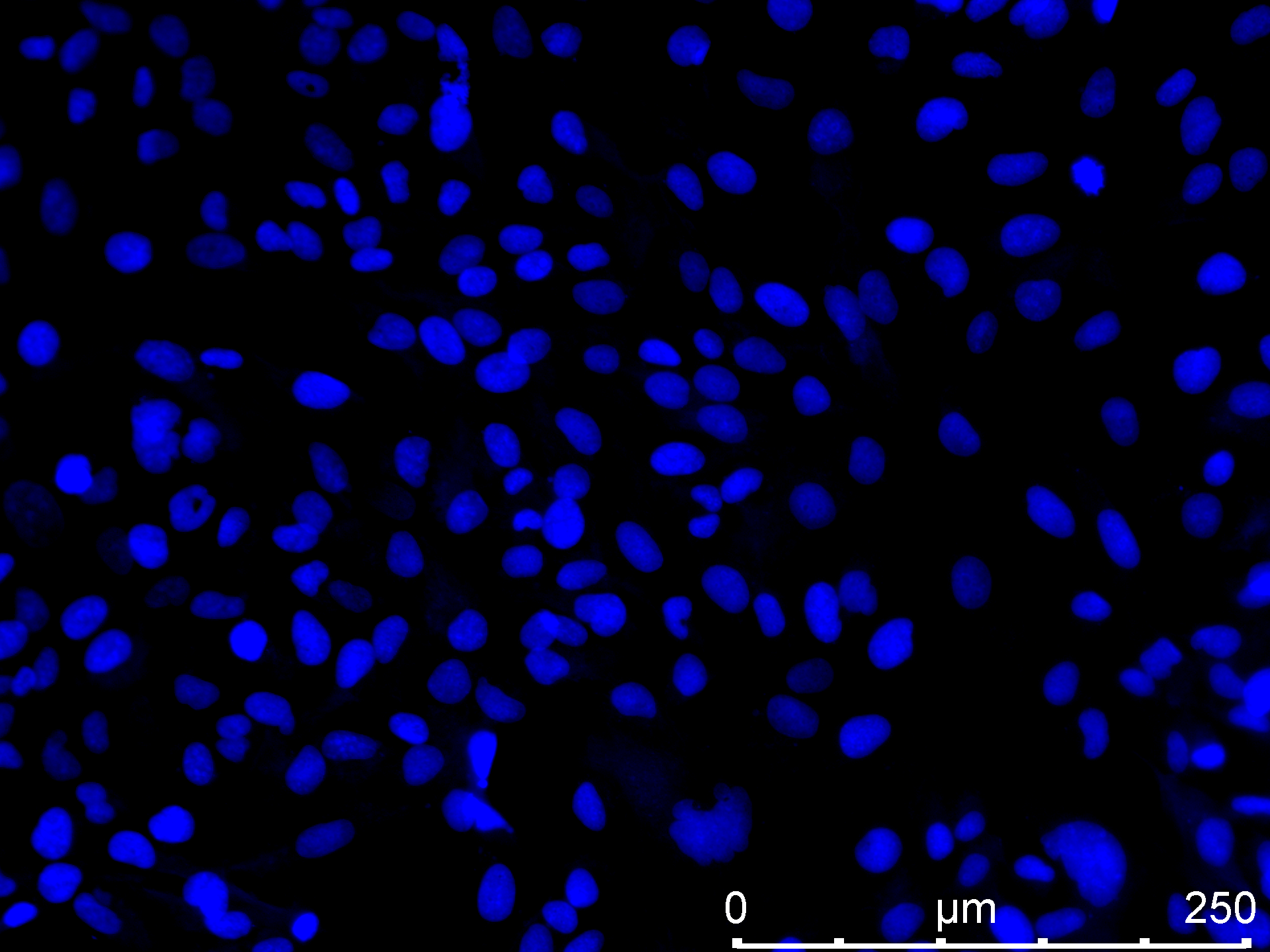

Supplement: Supplementary file 4 [file DataSheet_4.zip › Figure 4 raw datas/M/NC-siRNA 2.tif]

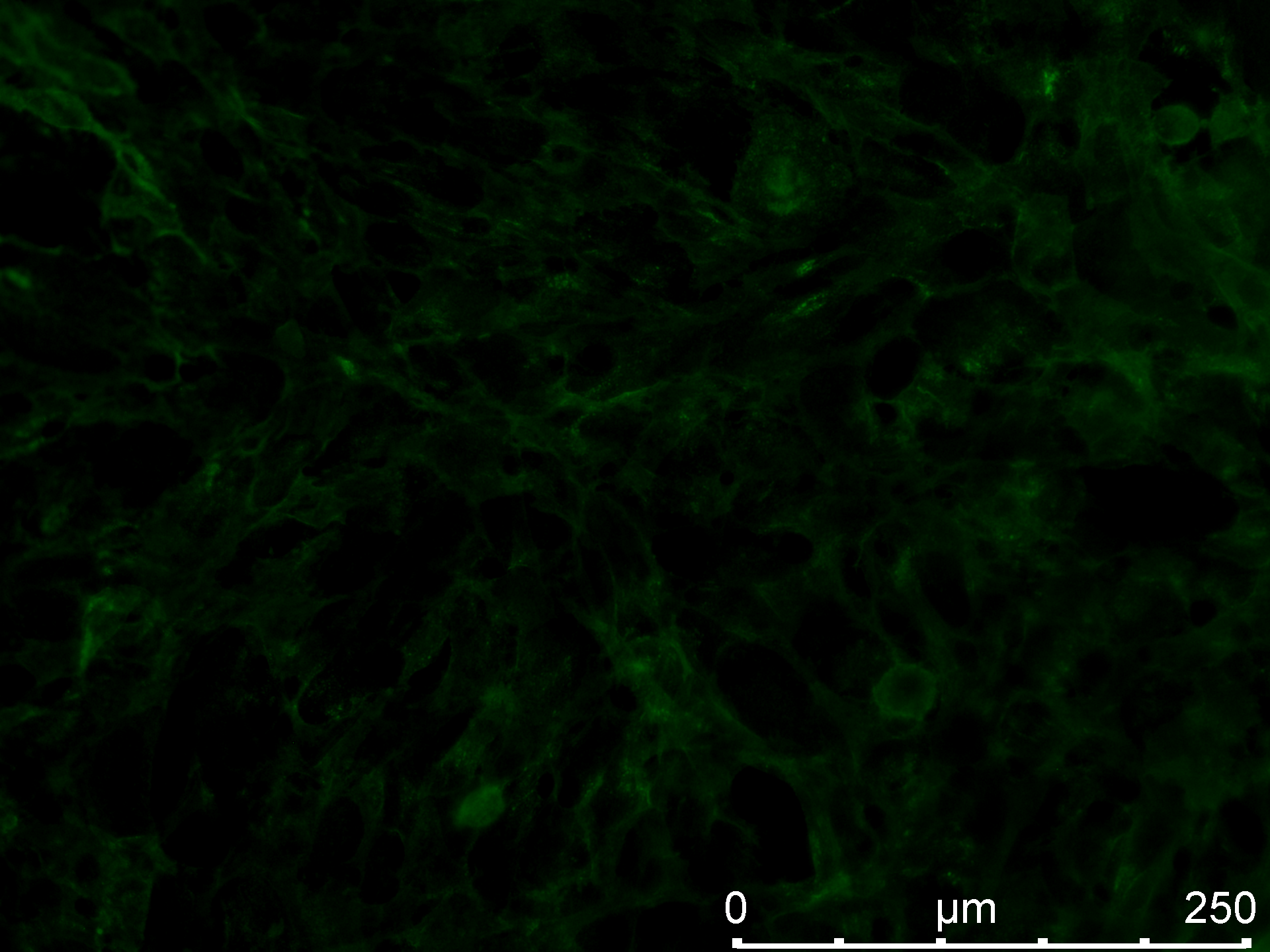

Supplement: Supplementary file 4 [file DataSheet_4.zip › Figure 4 raw datas/M/SDC-1 siRNA 11.tif]

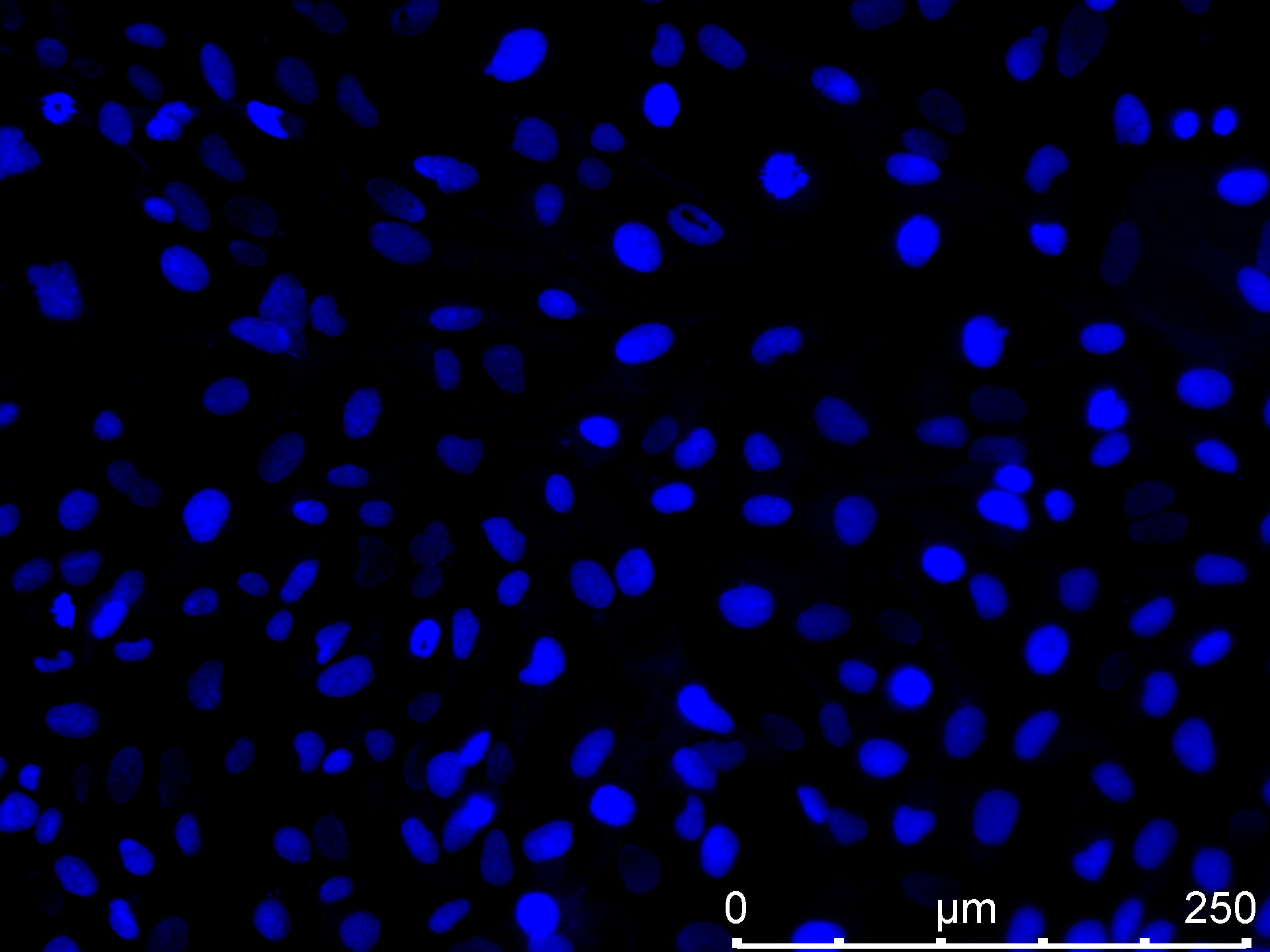

Supplement: Supplementary file 4 [file DataSheet_4.zip › Figure 4 raw datas/M/SDC-1 siRNA 12.tif]

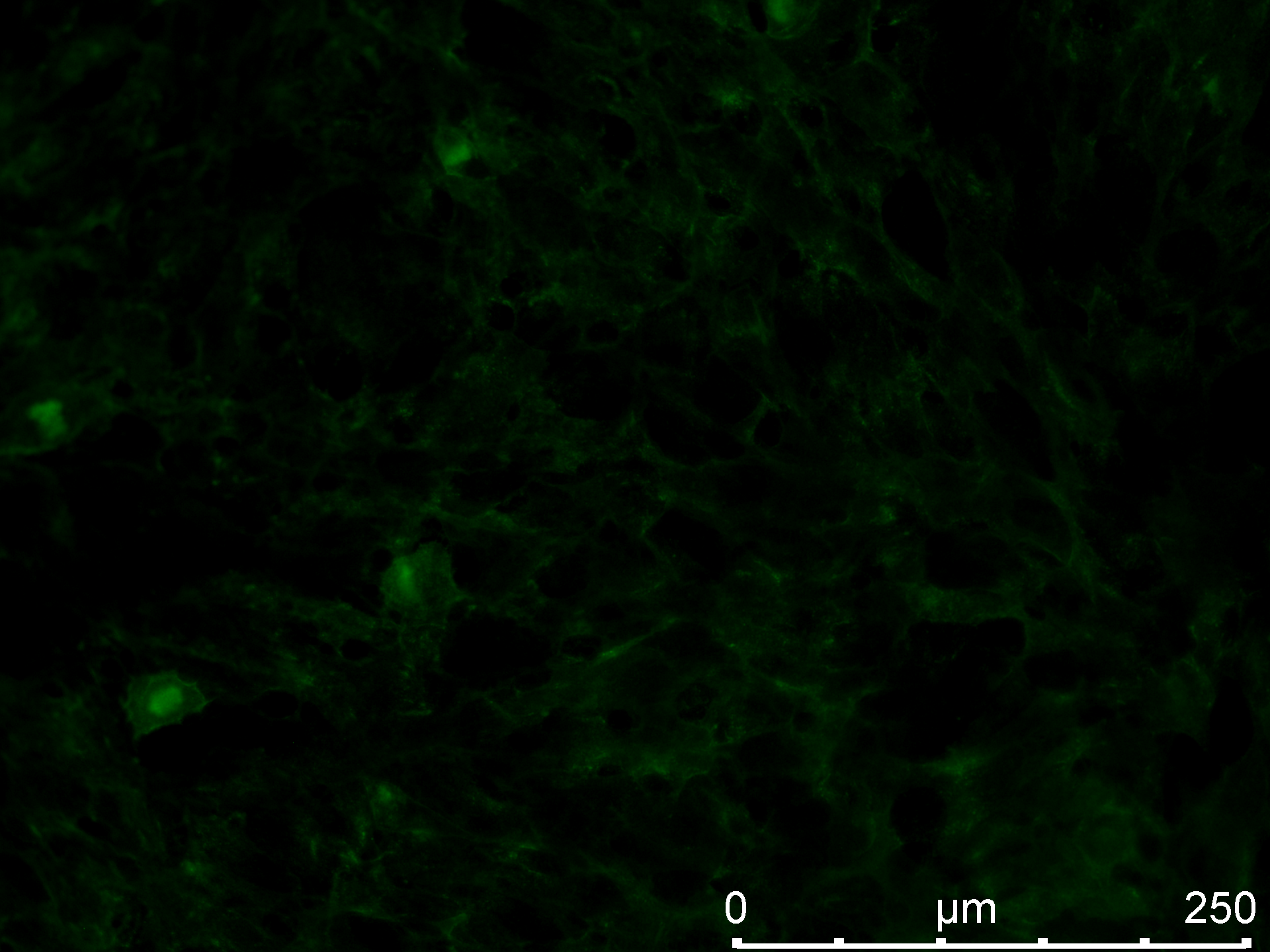

Supplement: Supplementary file 4 [file DataSheet_4.zip › Figure 4 raw datas/M/SDC-1 siRNA 21.tif]

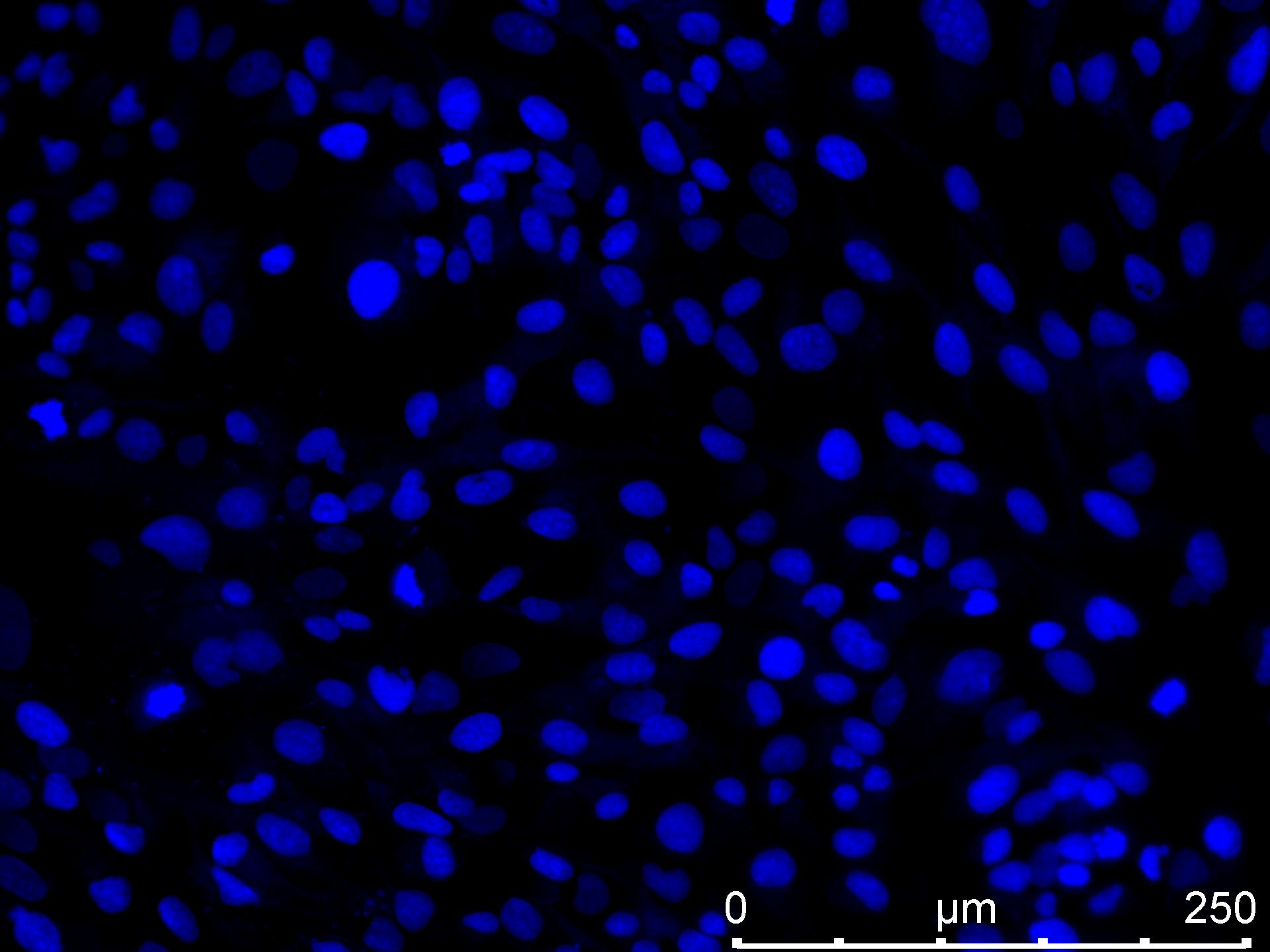

Supplement: Supplementary file 4 [file DataSheet_4.zip › Figure 4 raw datas/M/SDC-1 siRNA 22.tif]

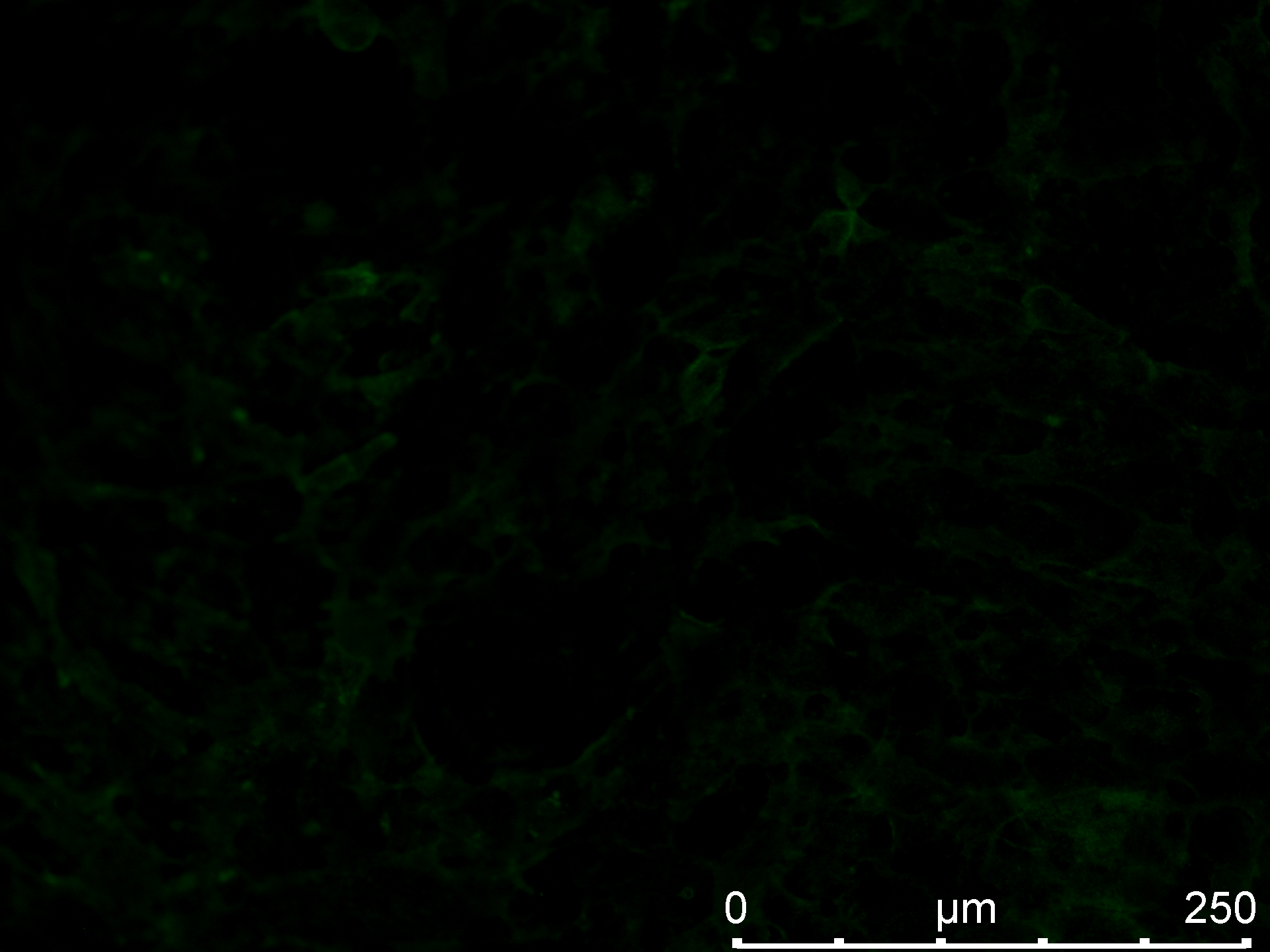

Supplement: Supplementary file 4 [file DataSheet_4.zip › Figure 4 raw datas/M/SDC-1 siRNA 31.tif]

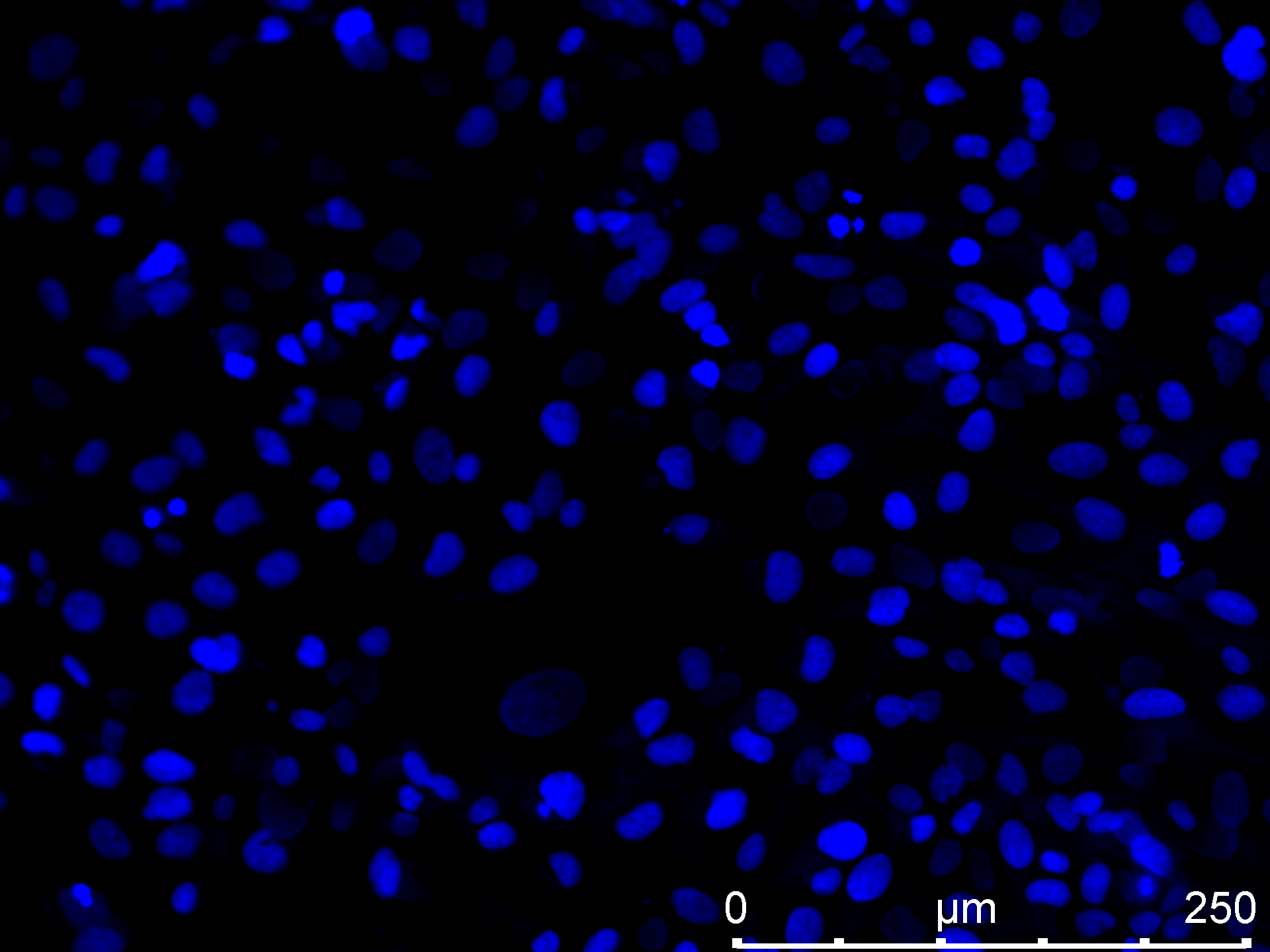

Supplement: Supplementary file 4 [file DataSheet_4.zip › Figure 4 raw datas/M/SDC-1 siRNA 32.tif]

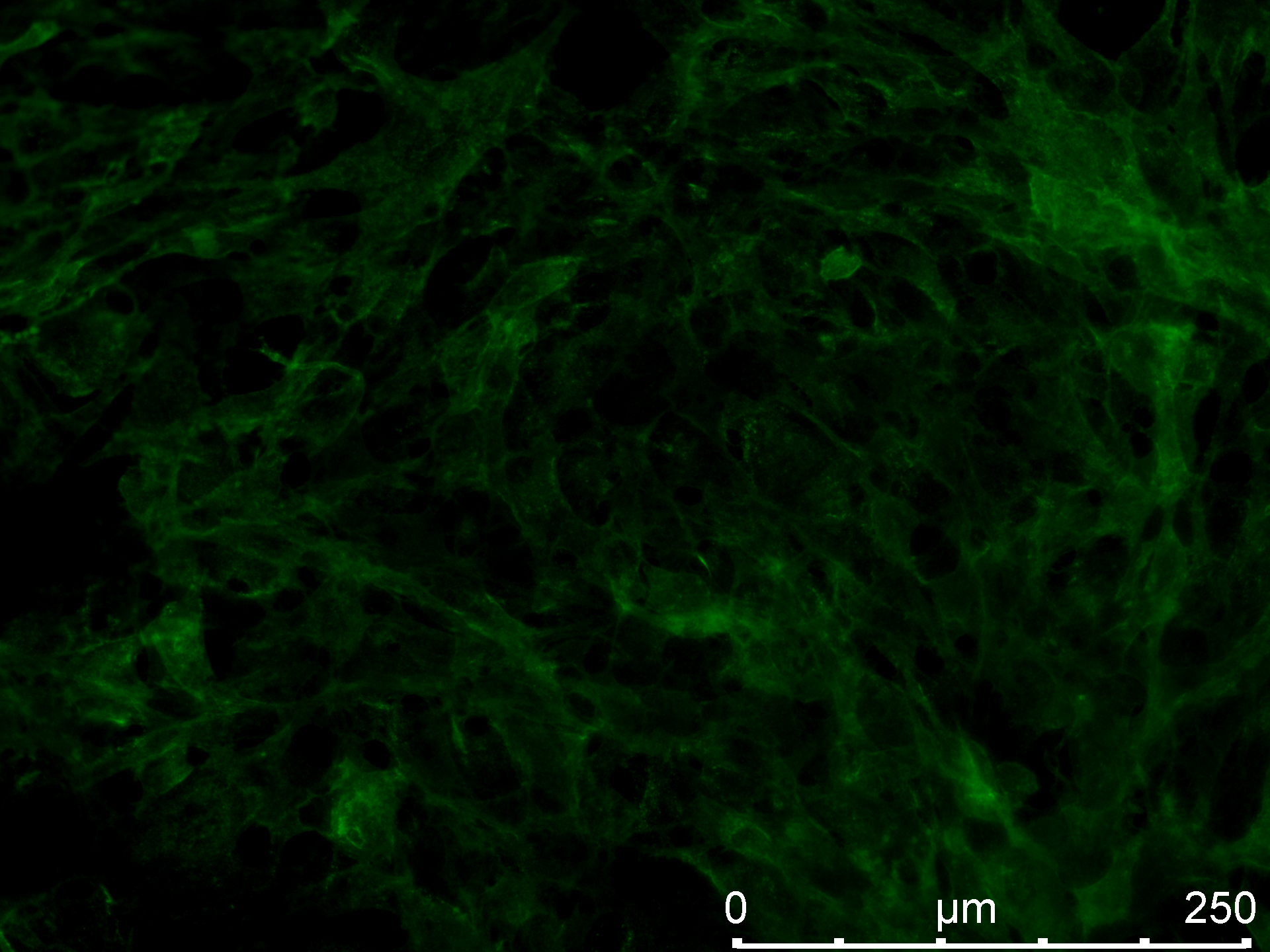

Supplement: Supplementary file 5 [file DataSheet_5.zip › Figure 5 raw datas/J/Control 1.tif]

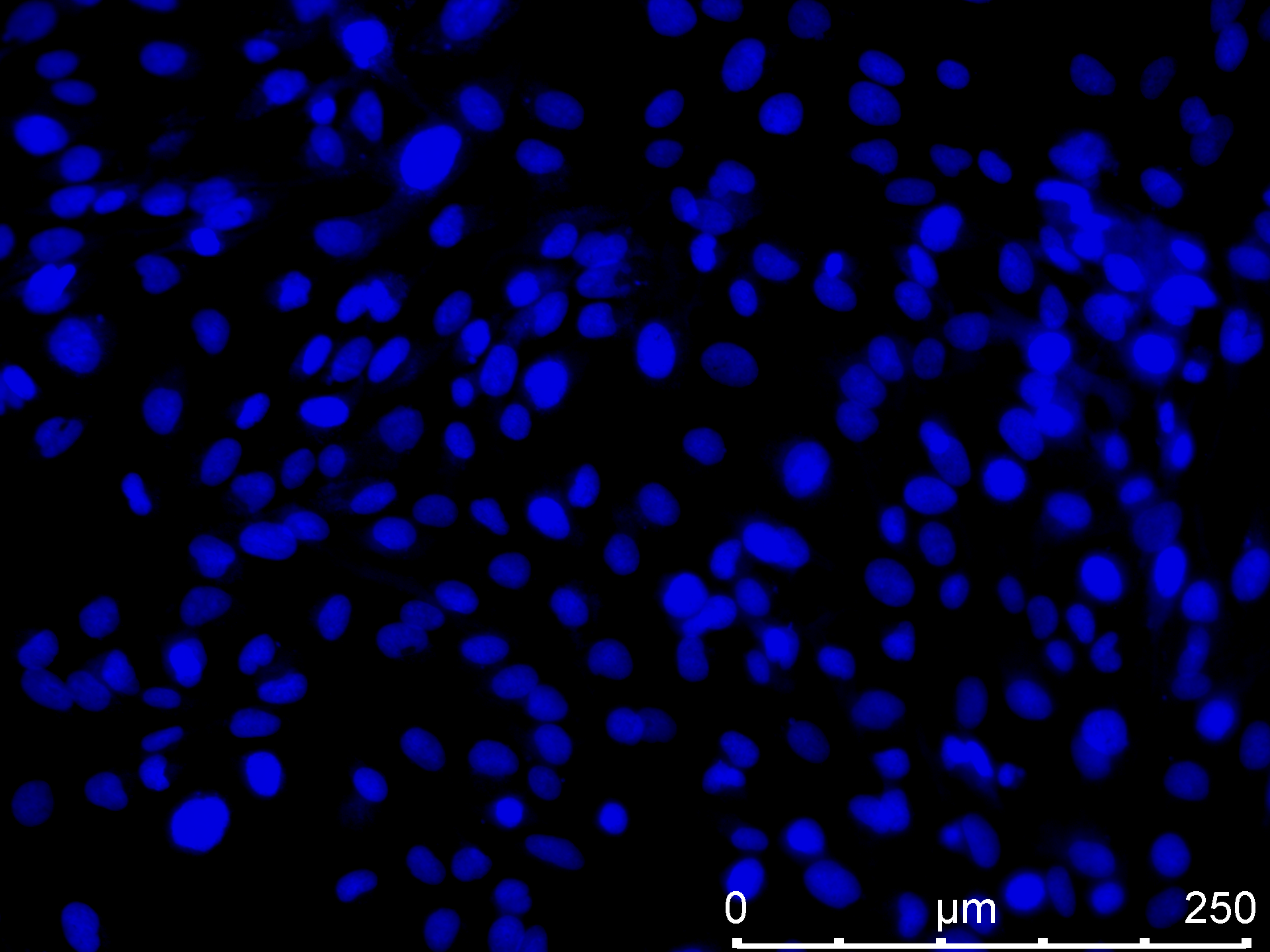

Supplement: Supplementary file 5 [file DataSheet_5.zip › Figure 5 raw datas/J/Control 2.tif]

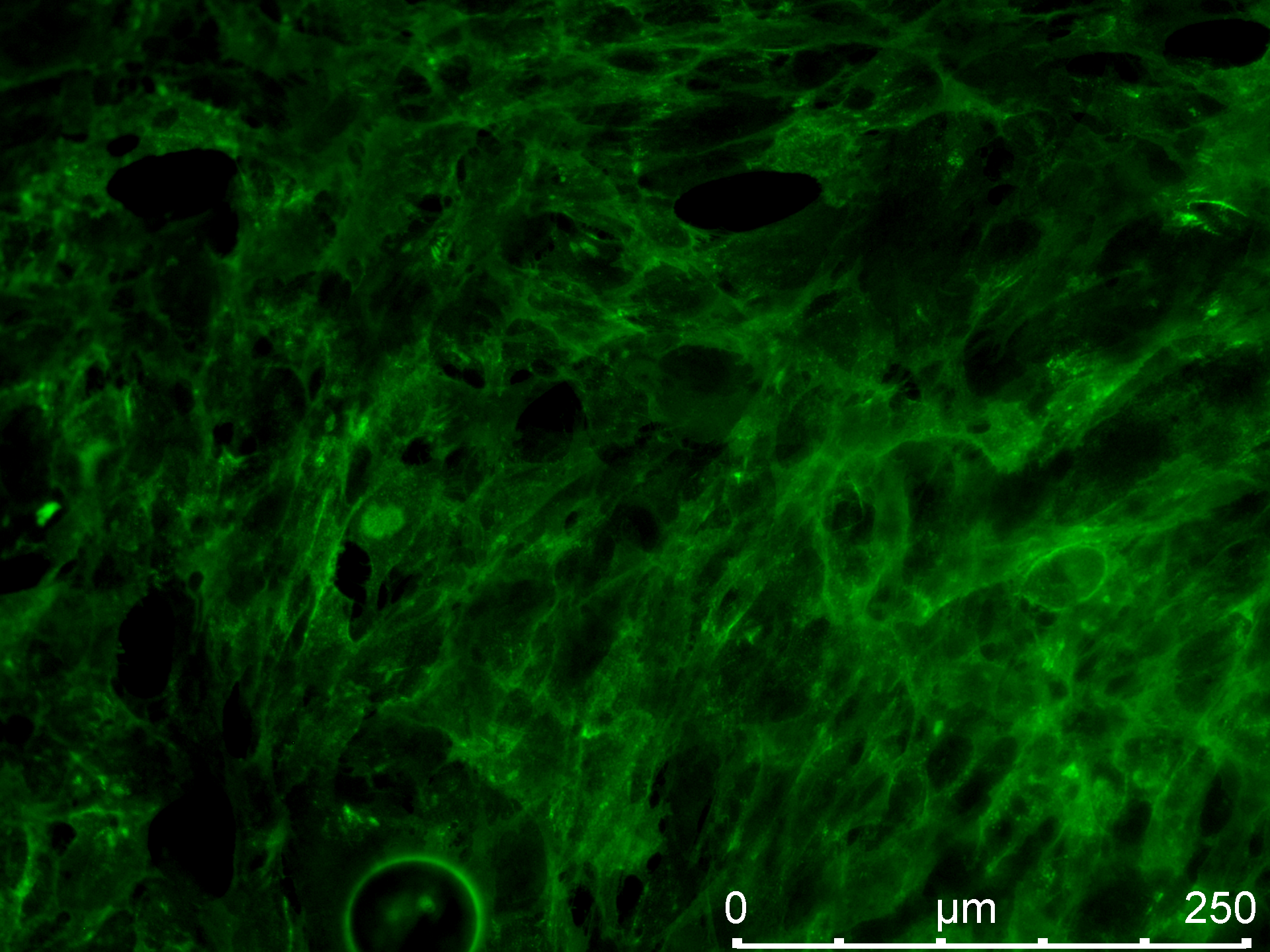

Supplement: Supplementary file 5 [file DataSheet_5.zip › Figure 5 raw datas/J/TGFB1 1.tif]

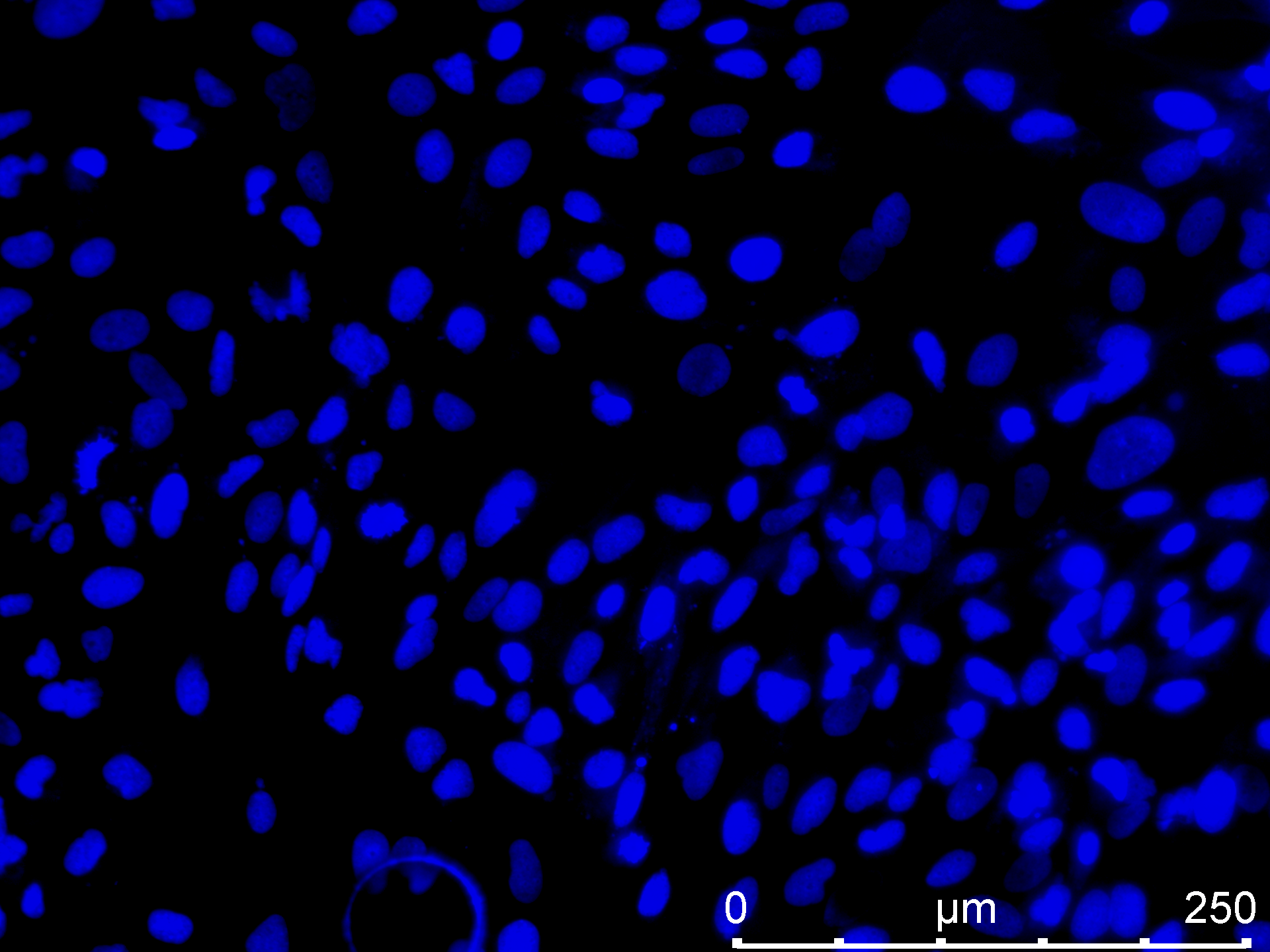

Supplement: Supplementary file 5 [file DataSheet_5.zip › Figure 5 raw datas/J/TGFB1 2.tif]

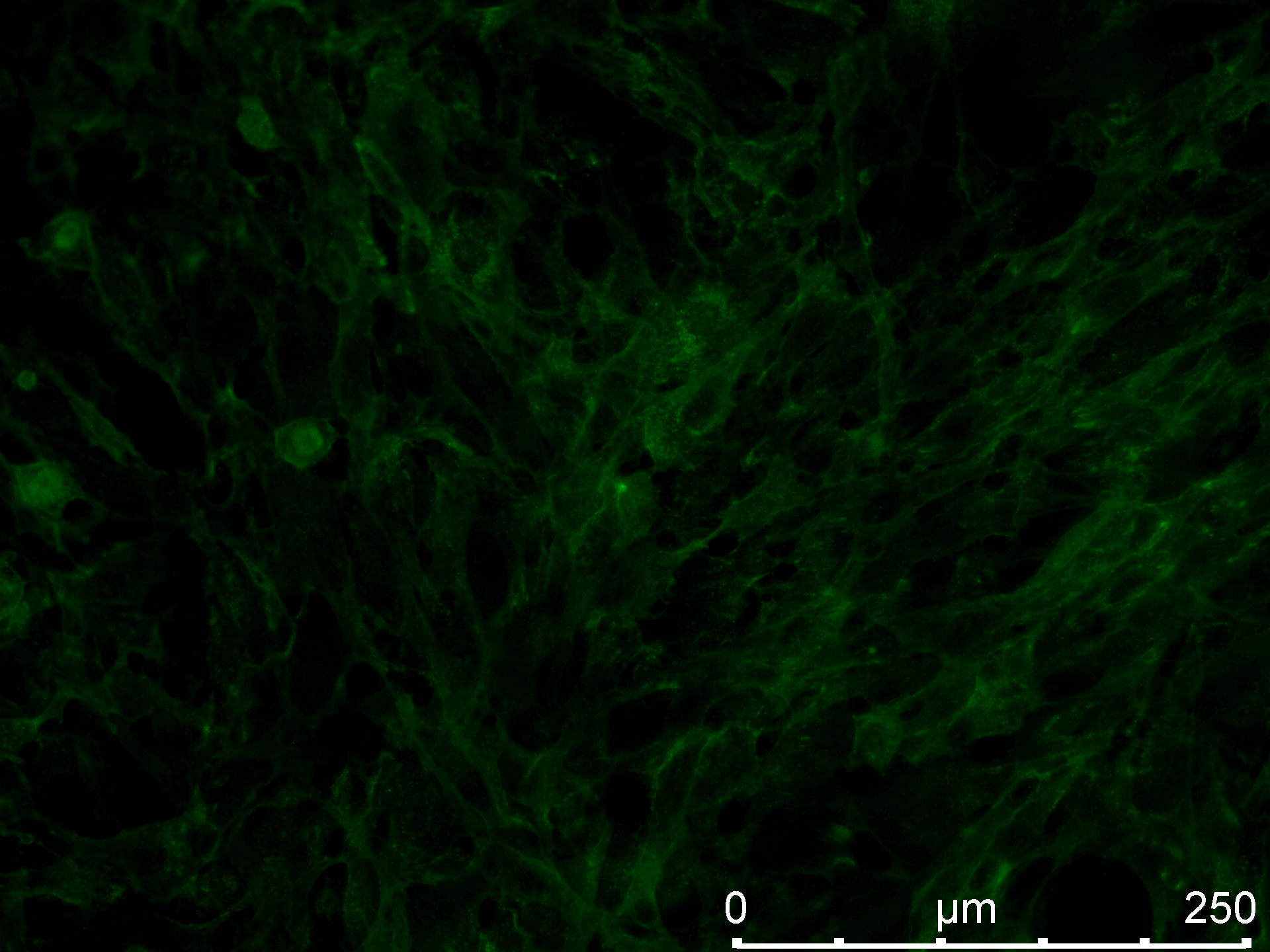

Supplement: Supplementary file 5 [file DataSheet_5.zip › Figure 5 raw datas/J/pc DNA3.1 1.tif]

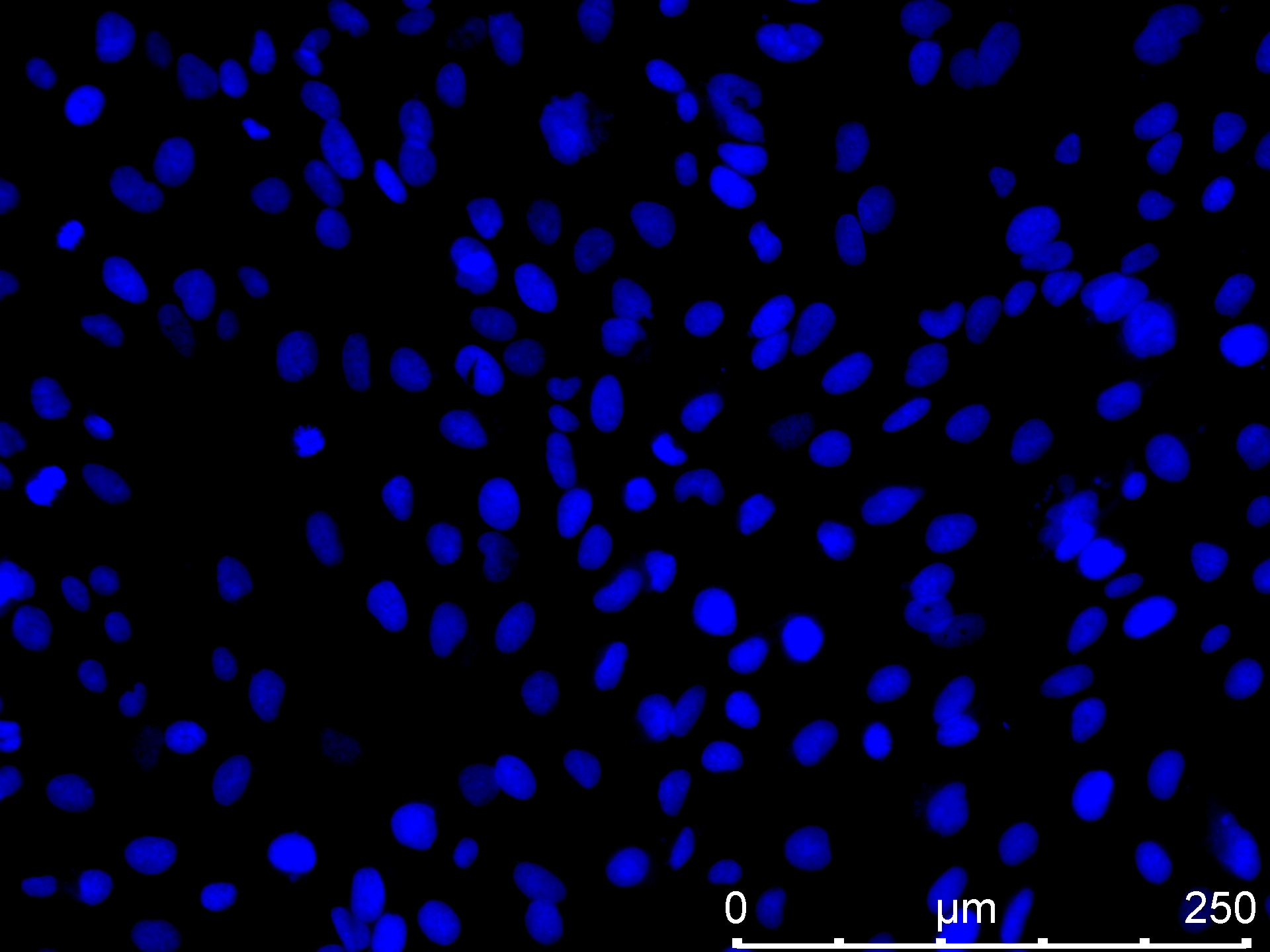

Supplement: Supplementary file 5 [file DataSheet_5.zip › Figure 5 raw datas/J/pc DNA3.1 2.tif]

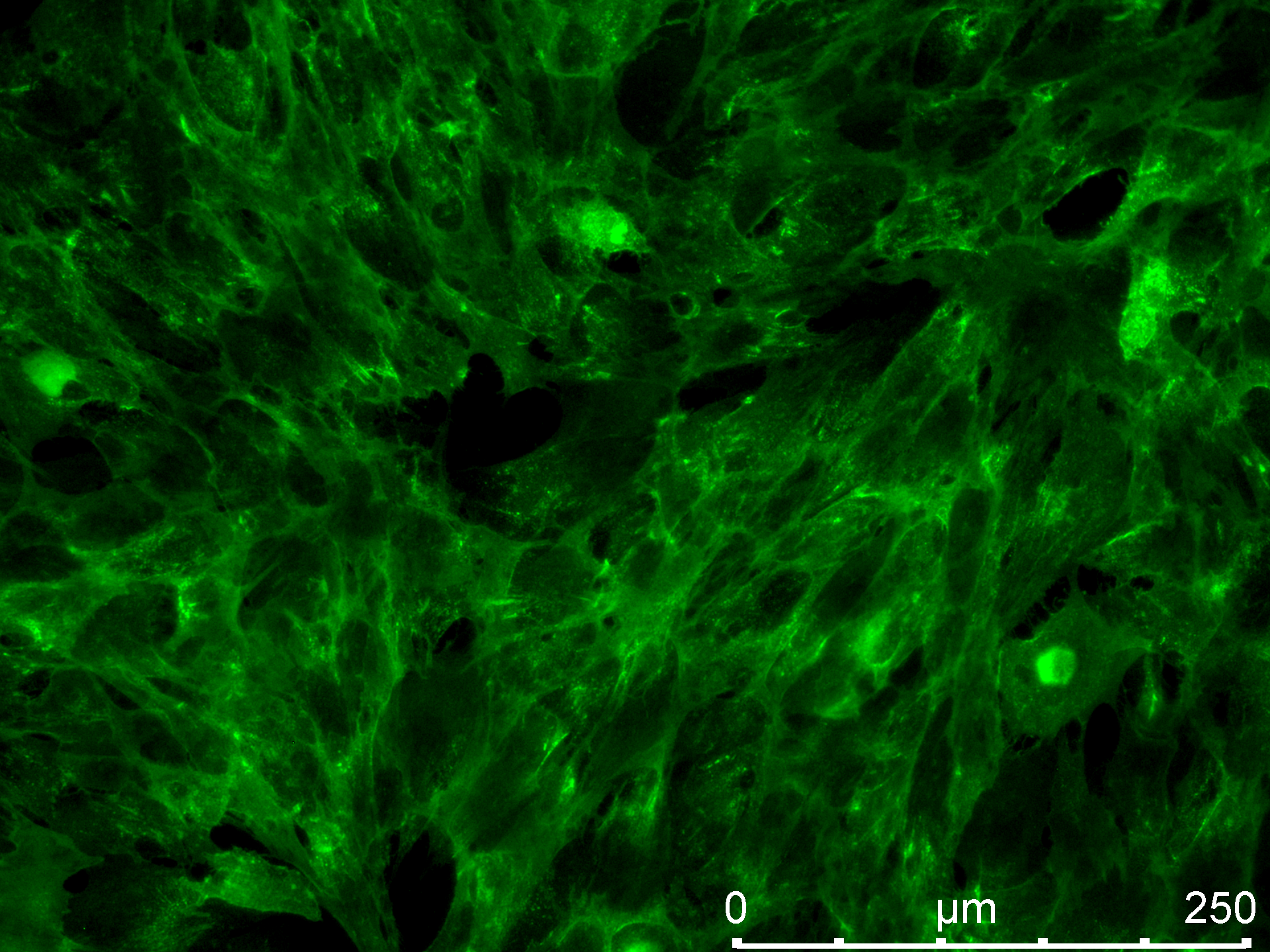

Supplement: Supplementary file 5 [file DataSheet_5.zip › Figure 5 raw datas/J/pc-SDC-1+TGFB1 1.tif]

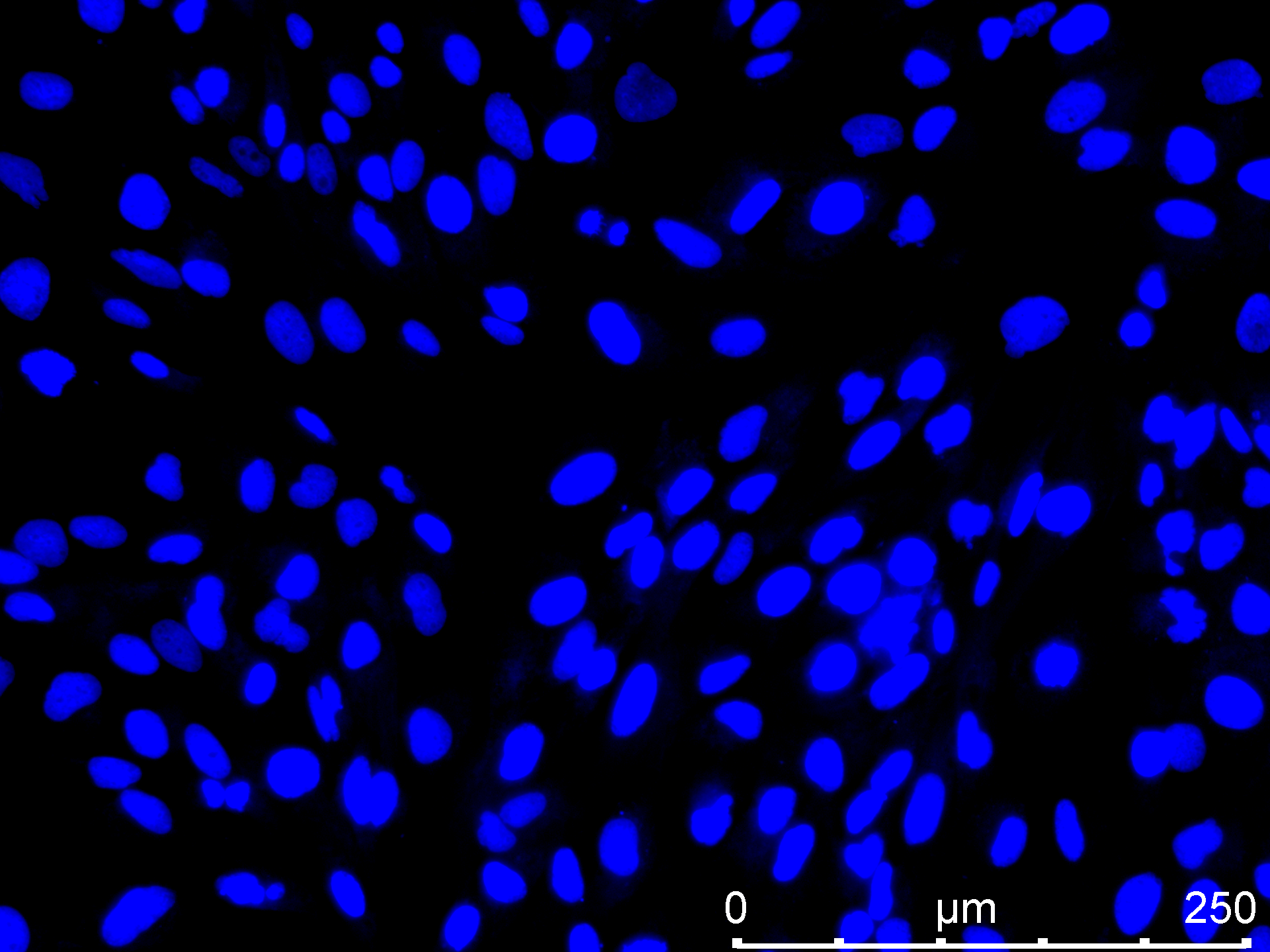

Supplement: Supplementary file 5 [file DataSheet_5.zip › Figure 5 raw datas/J/pc-SDC-1+TGFB1 2.tif]

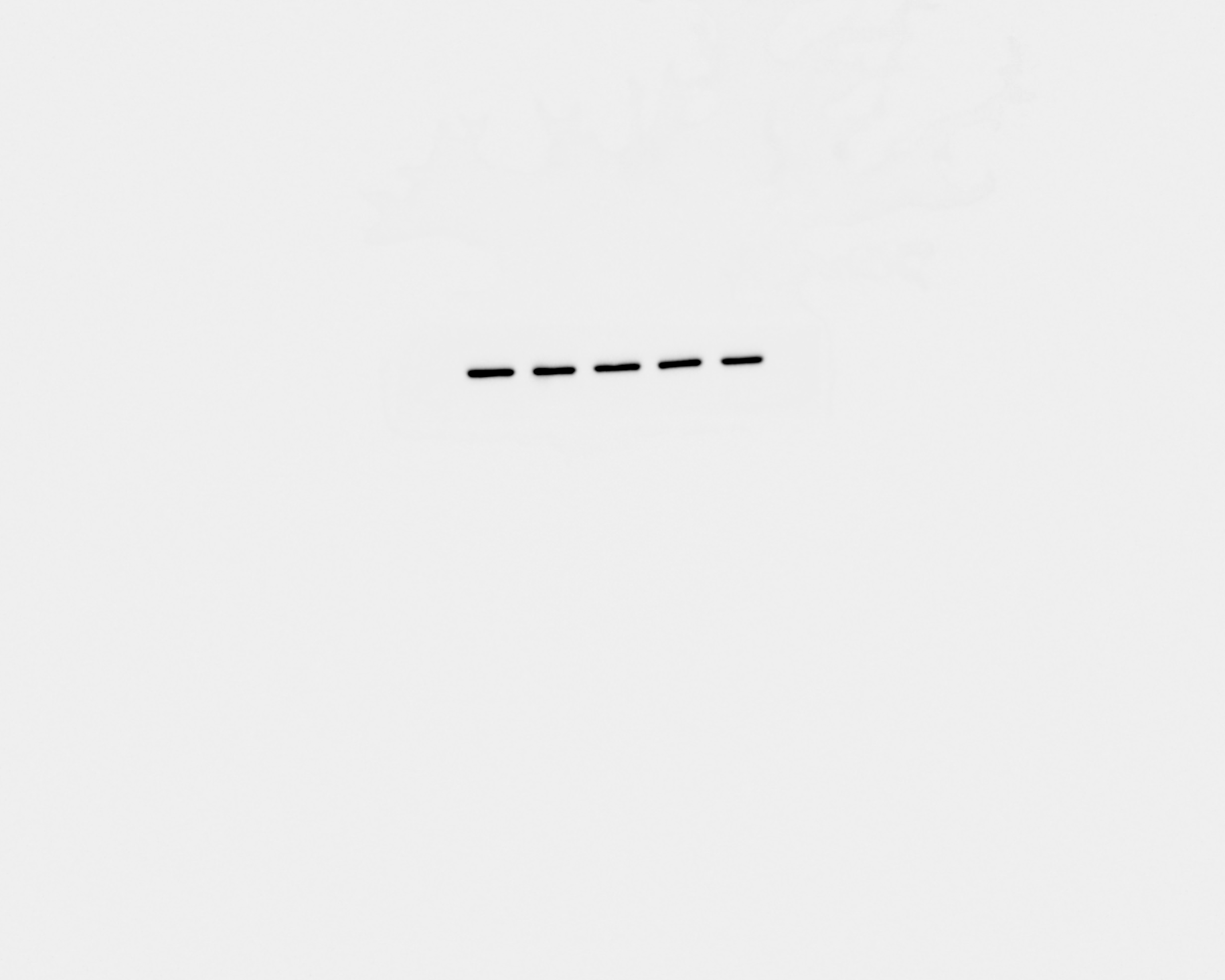

Supplement: Supplementary file 6 [file DataSheet_6.zip › Figure 6 raw datas/A B C/A. GAPDH.tif]

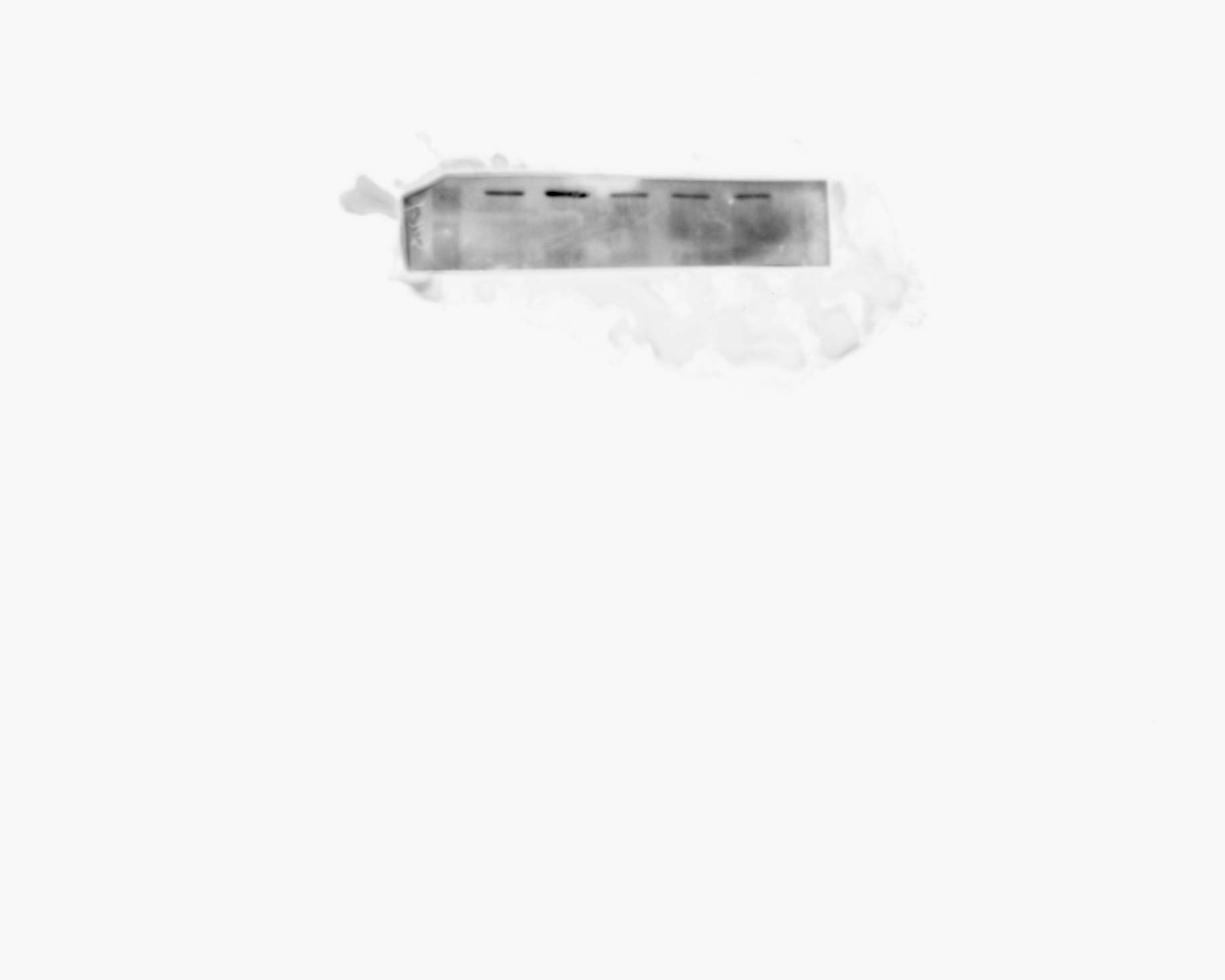

Supplement: Supplementary file 6 [file DataSheet_6.zip › Figure 6 raw datas/A B C/A. SDC-1.tif]

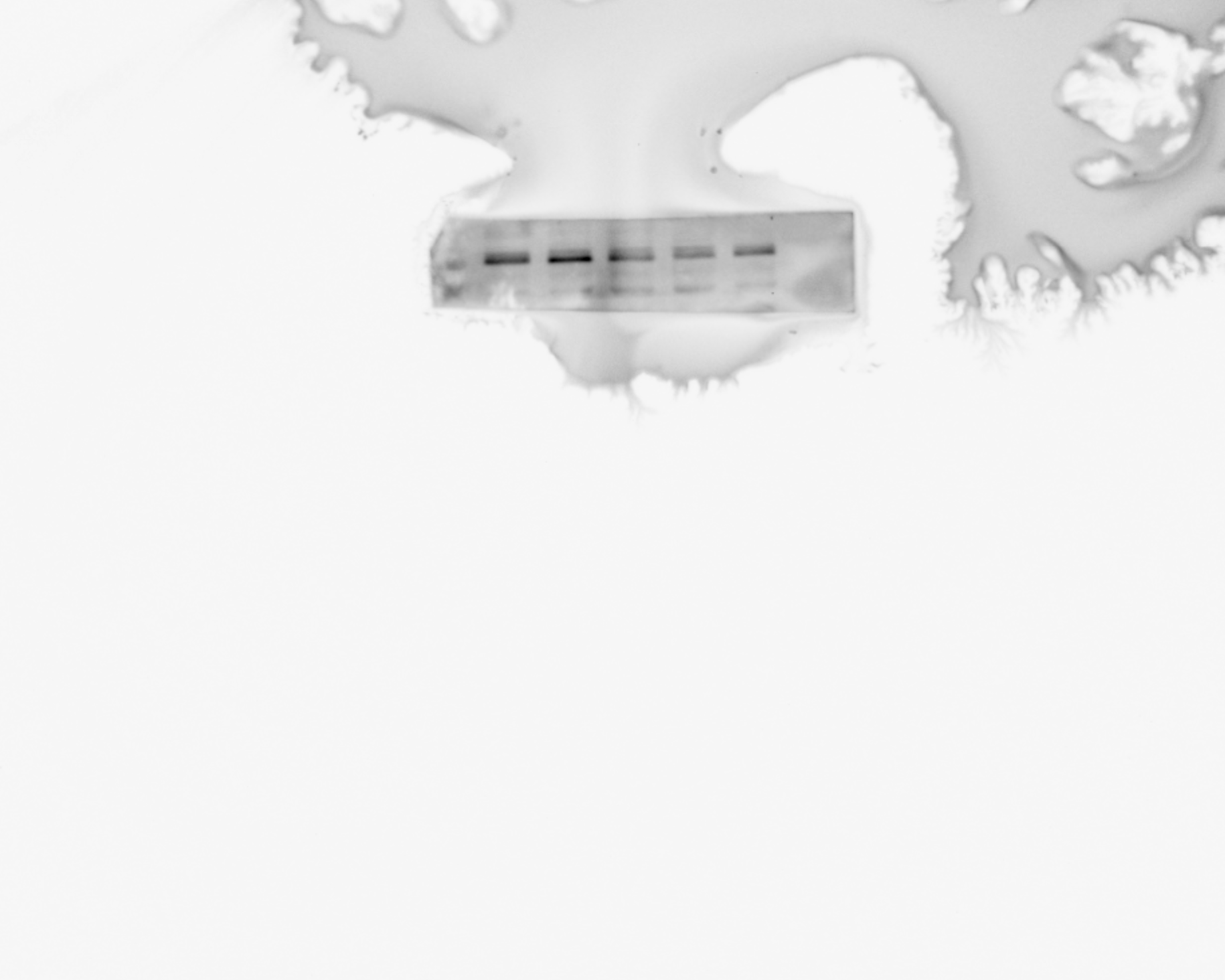

Supplement: Supplementary file 6 [file DataSheet_6.zip › Figure 6 raw datas/A B C/B. p-smad3.tif]

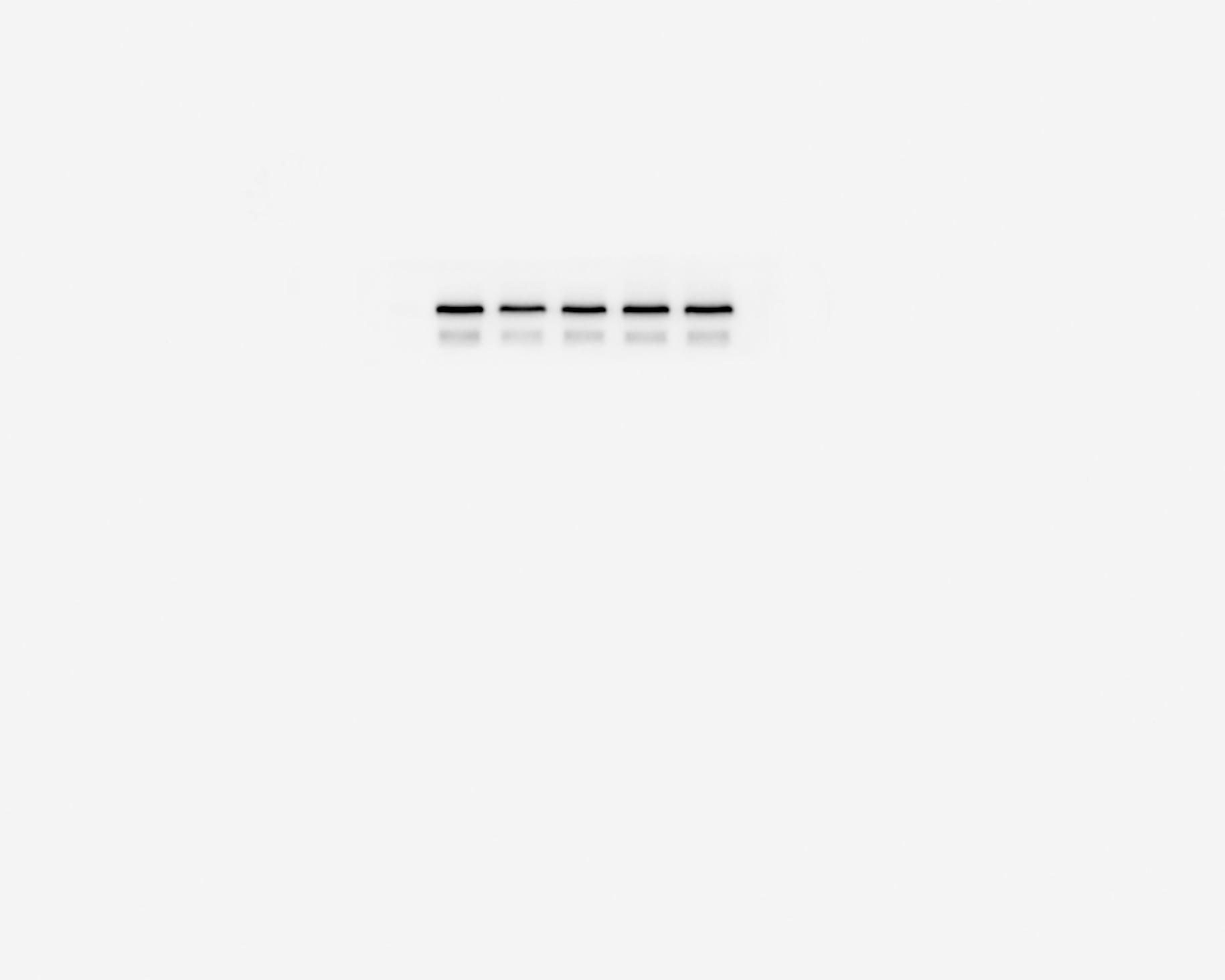

Supplement: Supplementary file 6 [file DataSheet_6.zip › Figure 6 raw datas/A B C/B. smad3.tif]

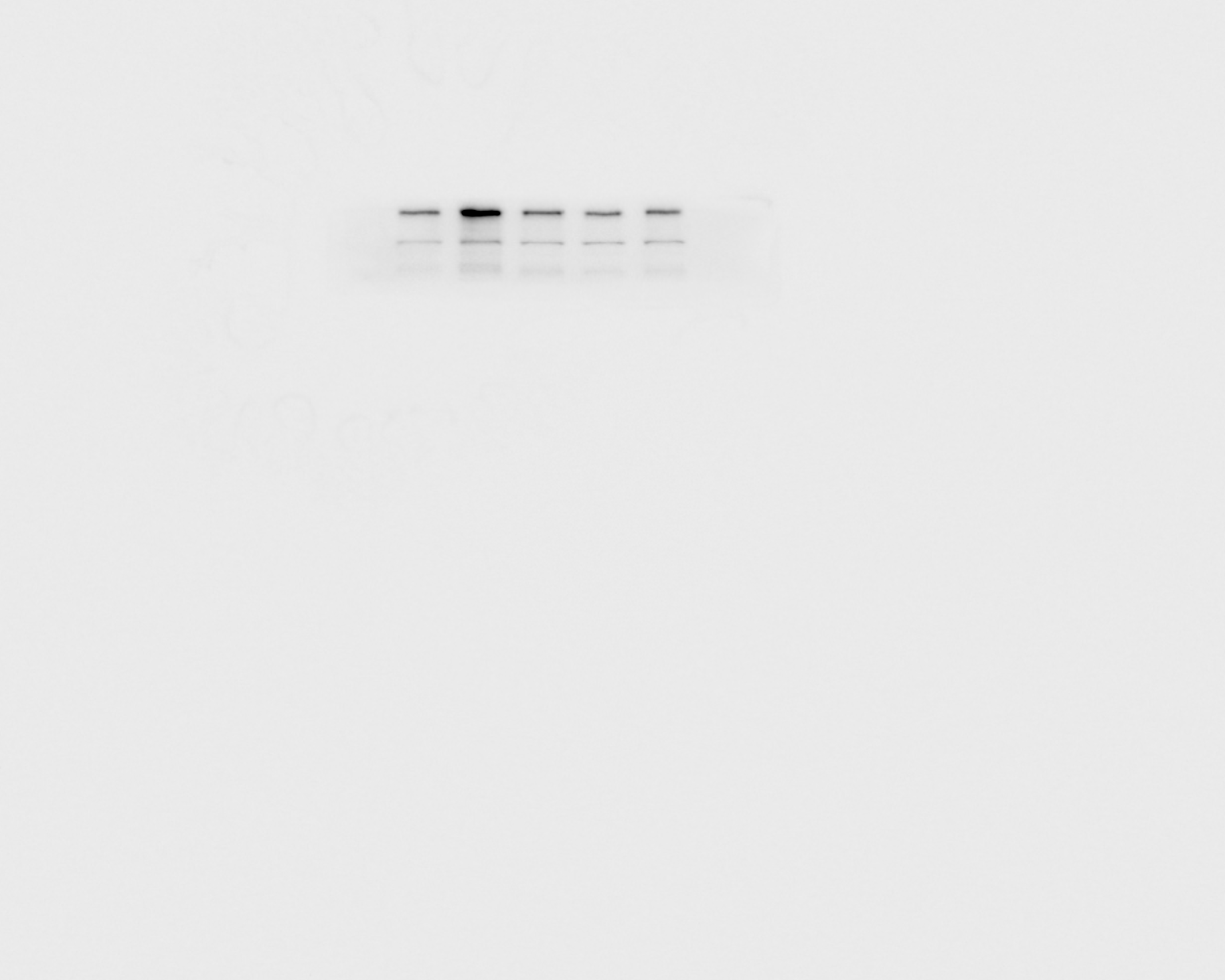

Supplement: Supplementary file 6 [file DataSheet_6.zip › Figure 6 raw datas/A B C/C. Collagen I.tif]

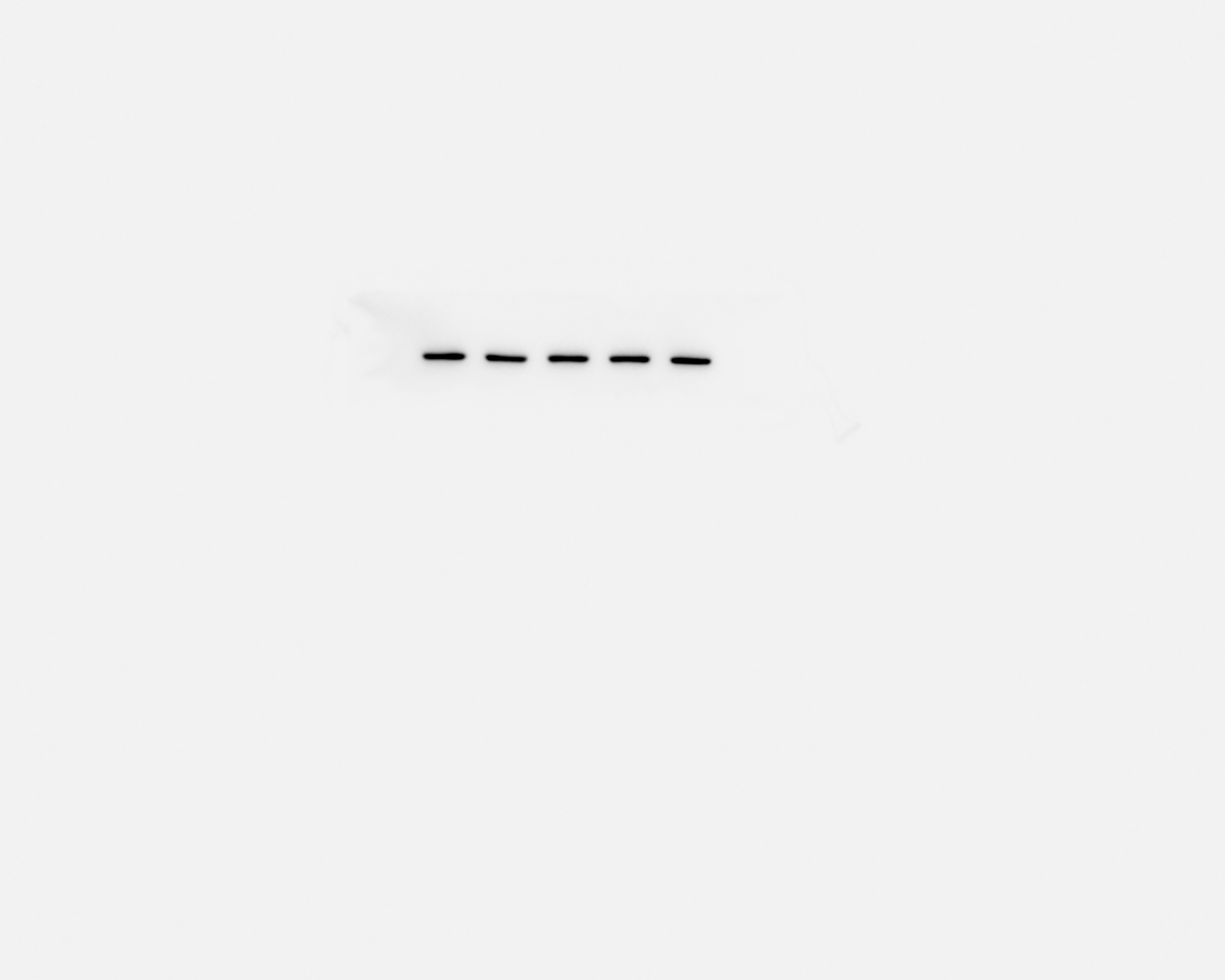

Supplement: Supplementary file 6 [file DataSheet_6.zip › Figure 6 raw datas/A B C/C. GAPDH.tif]

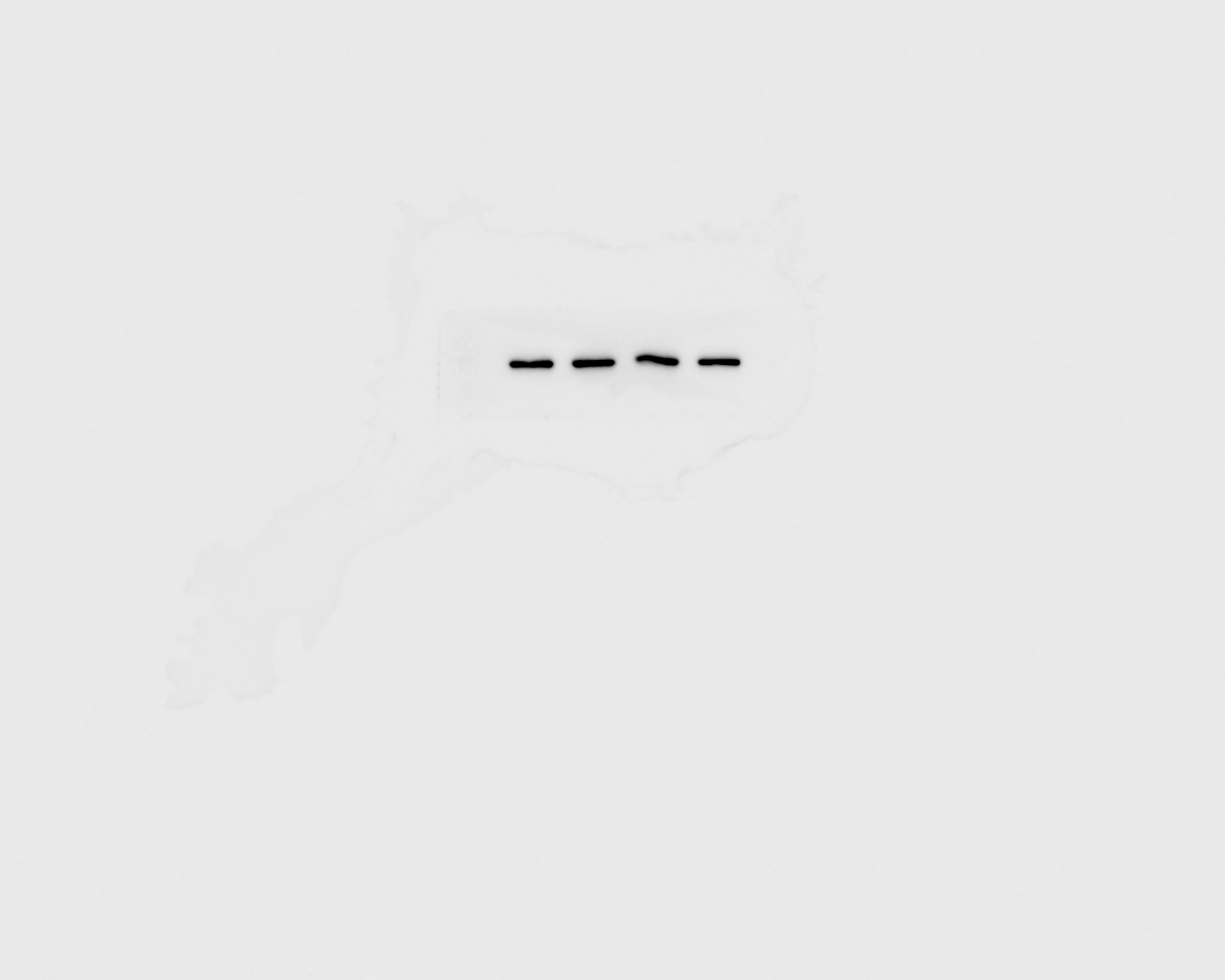

Supplement: Supplementary file 6 [file DataSheet_6.zip › Figure 6 raw datas/D E F/D. GAPDH.tif]

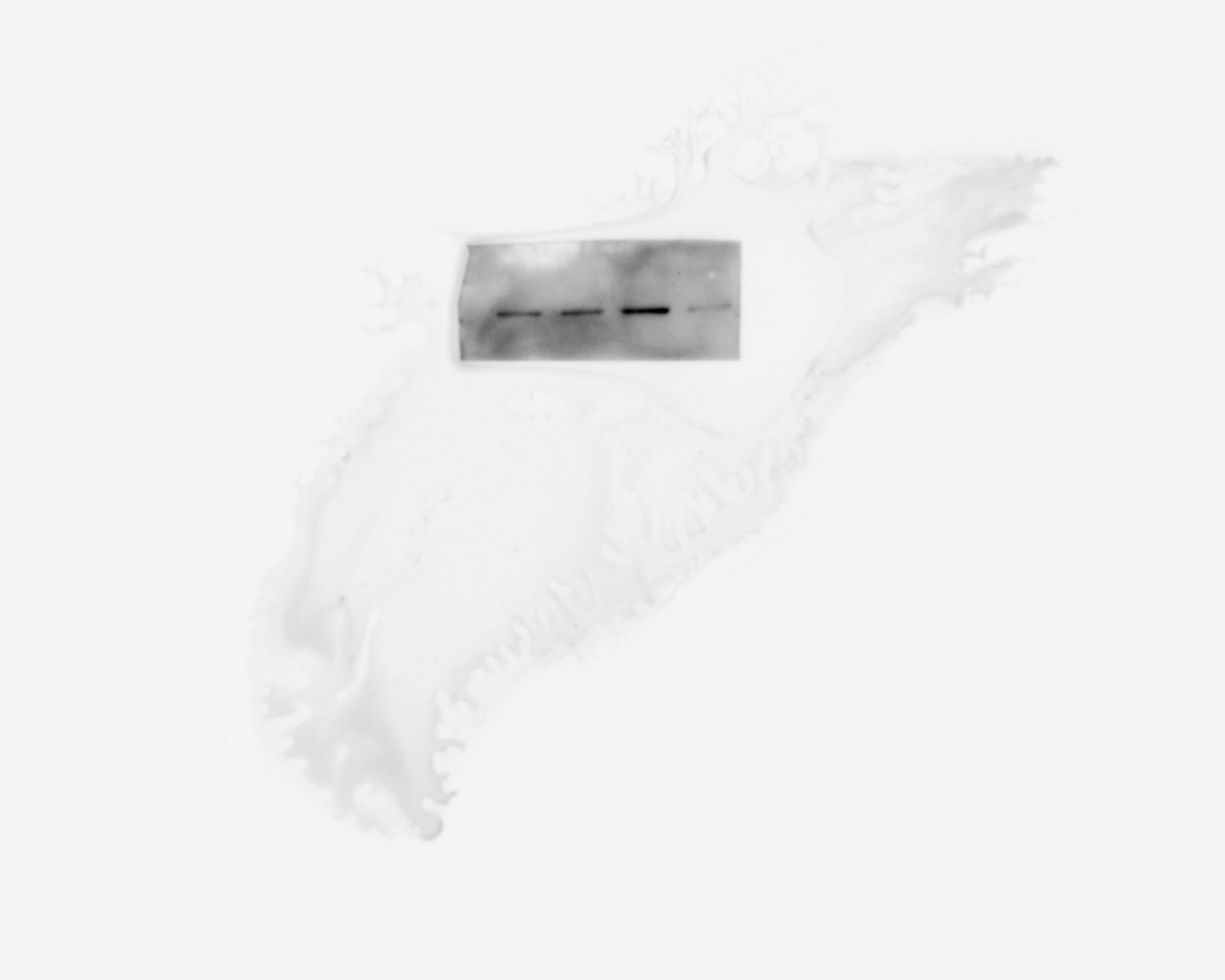

Supplement: Supplementary file 6 [file DataSheet_6.zip › Figure 6 raw datas/D E F/D. SDC-1.tif]

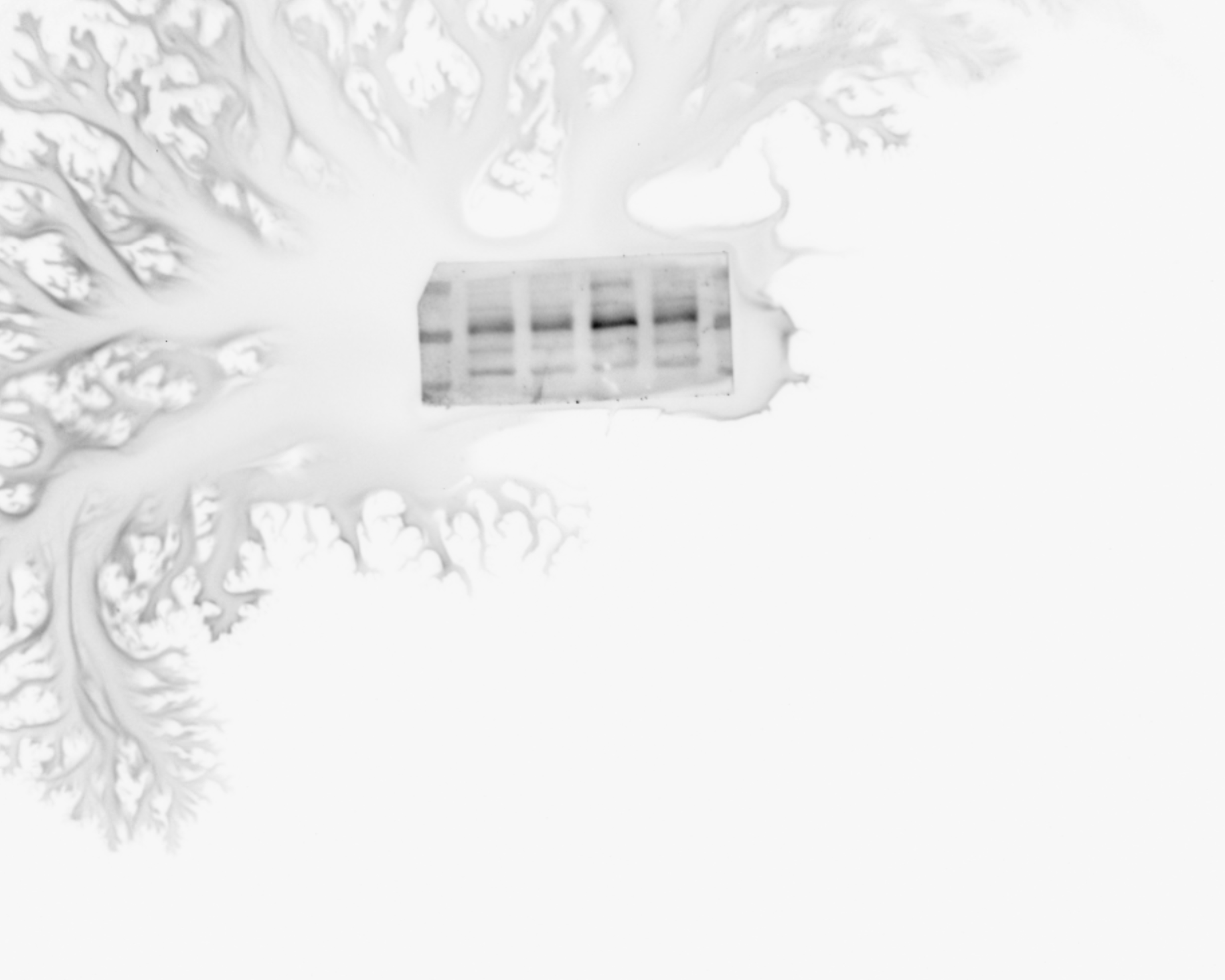

Supplement: Supplementary file 6 [file DataSheet_6.zip › Figure 6 raw datas/D E F/E. p-smad3.tif]

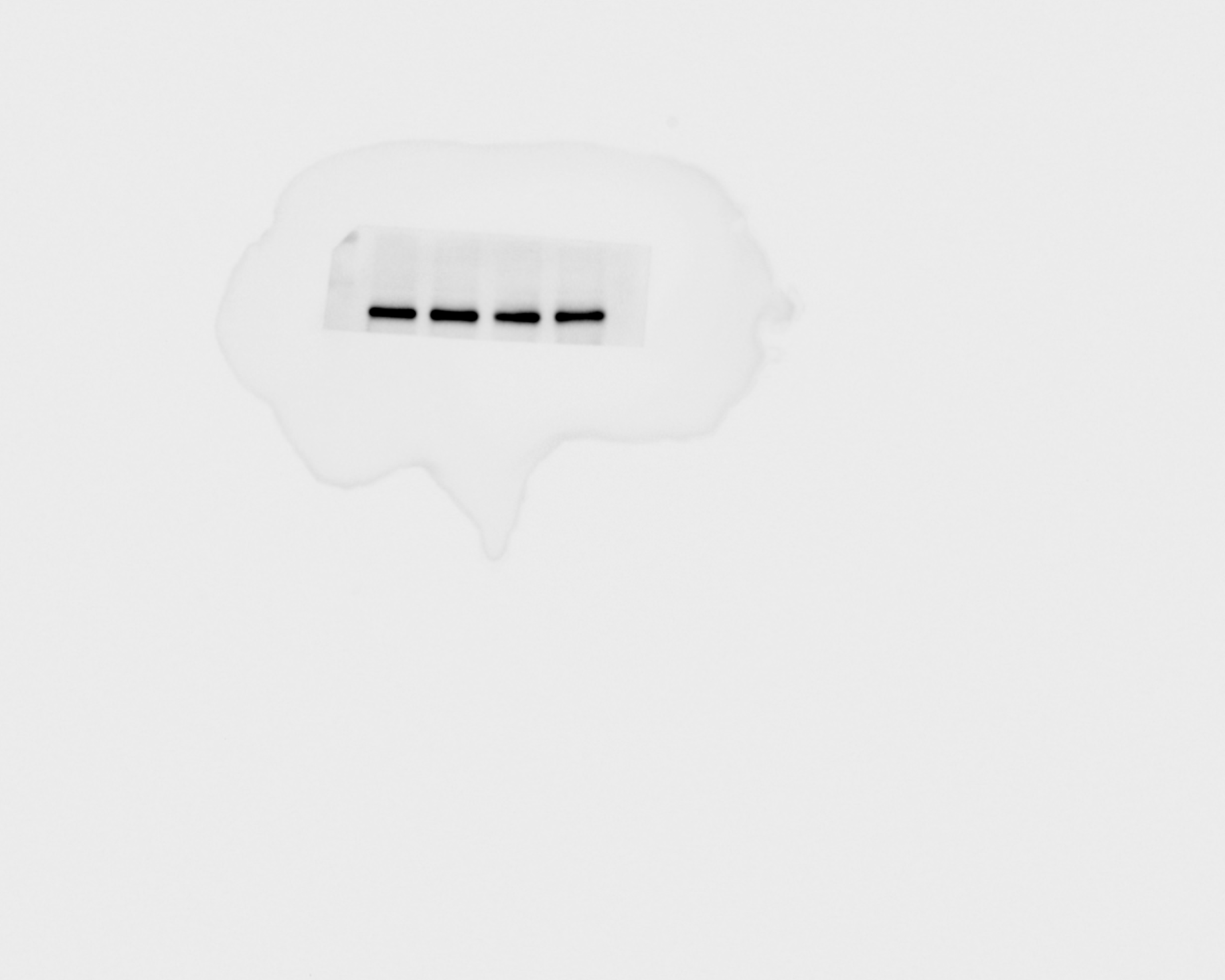

Supplement: Supplementary file 6 [file DataSheet_6.zip › Figure 6 raw datas/D E F/E. smad3.tif]

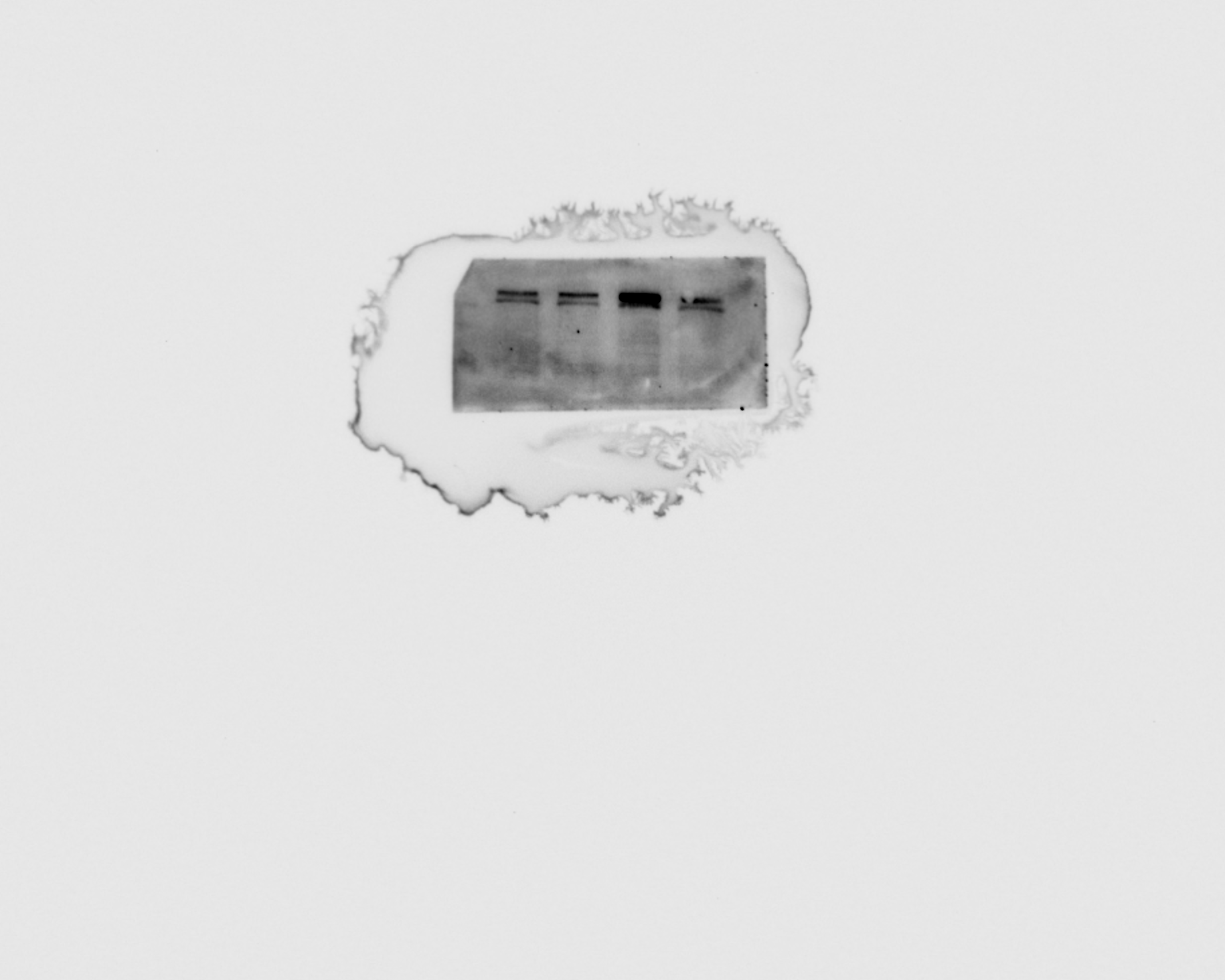

Supplement: Supplementary file 6 [file DataSheet_6.zip › Figure 6 raw datas/D E F/F. Collagen I.tif]

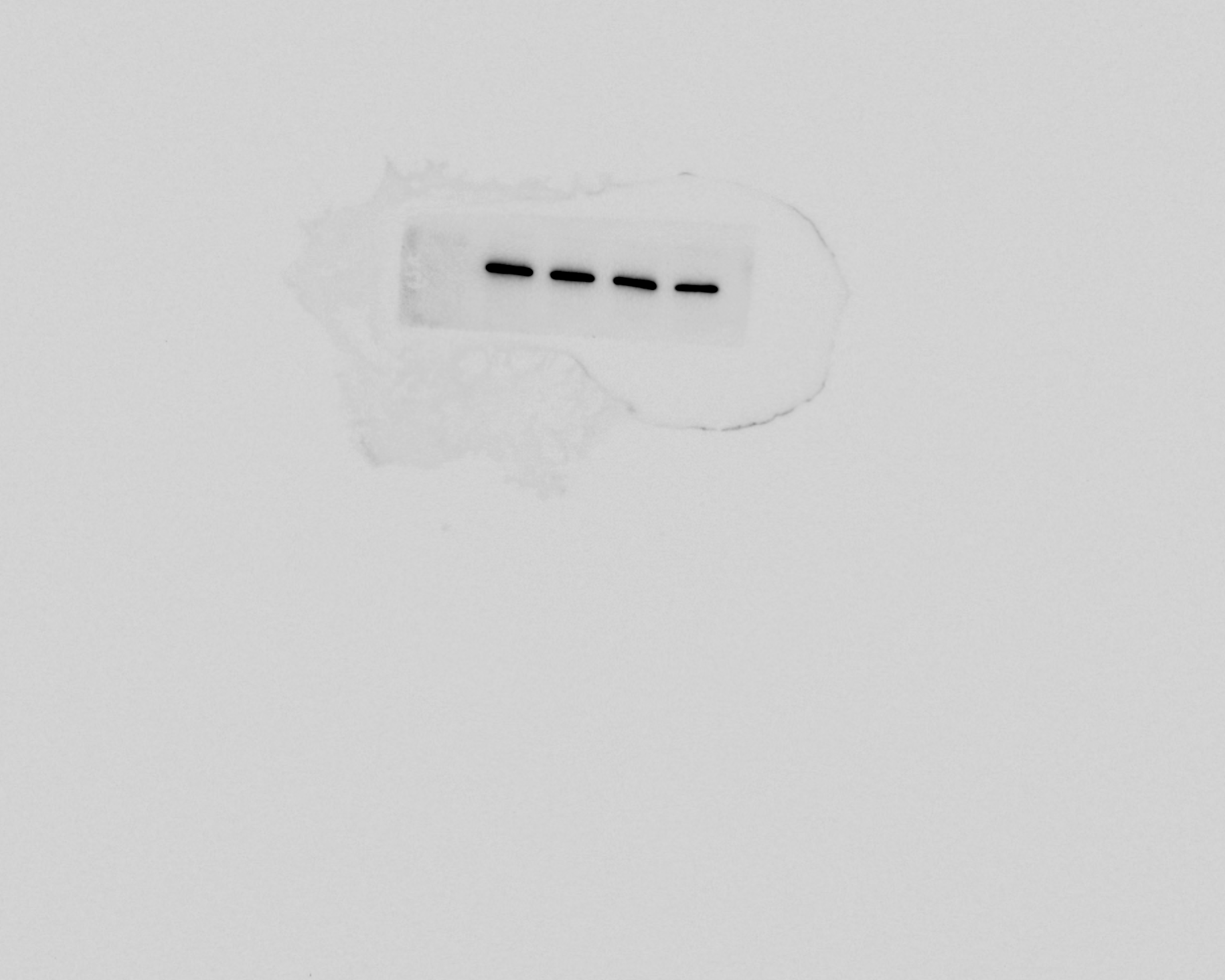

Supplement: Supplementary file 6 [file DataSheet_6.zip › Figure 6 raw datas/D E F/F. GAPDH.tif]

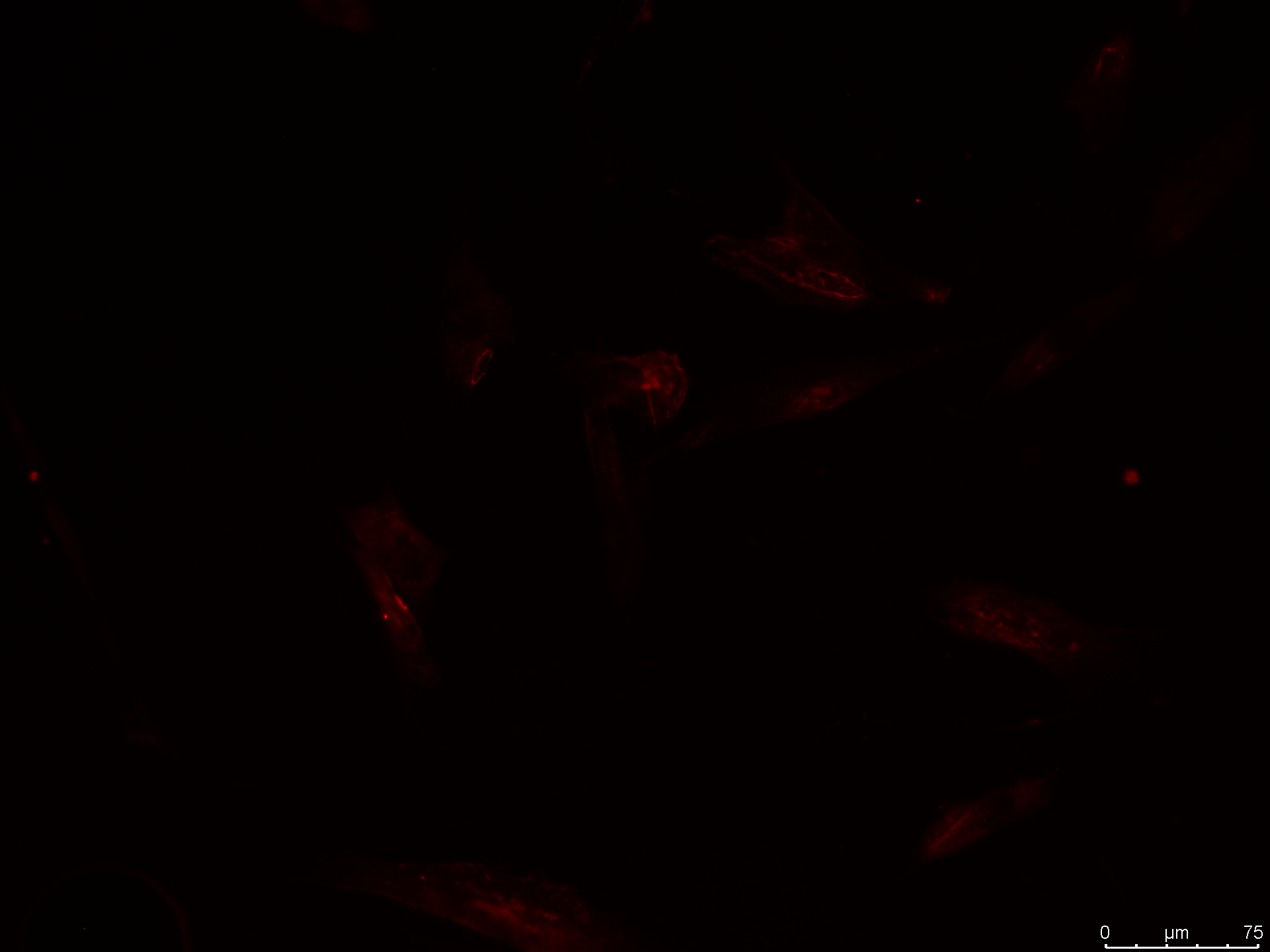

Supplement: Supplementary file 6 [file DataSheet_6.zip › Figure 6 raw datas/M/1.NC siRNA/NC siRNA 1.tif]

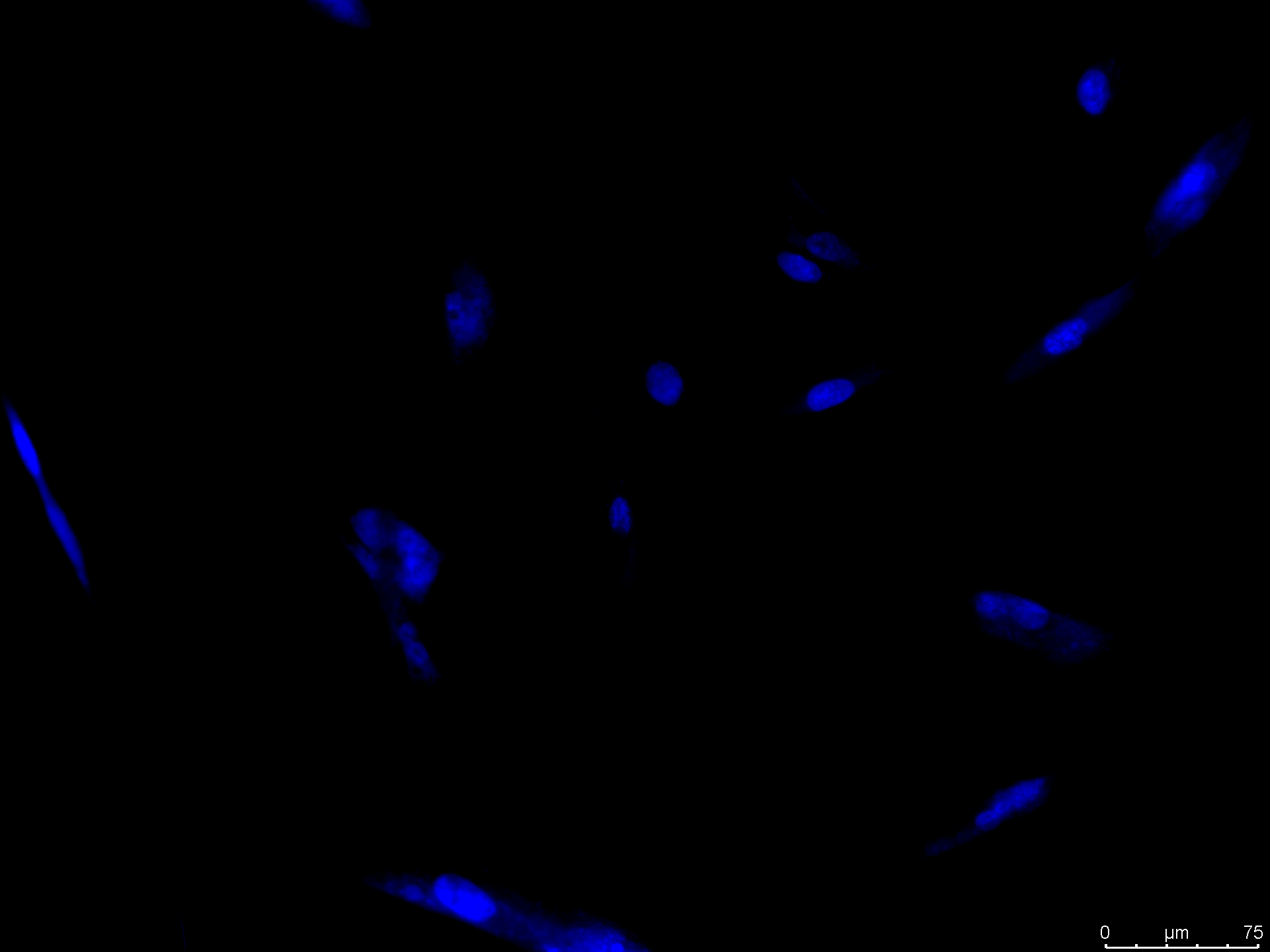

Supplement: Supplementary file 6 [file DataSheet_6.zip › Figure 6 raw datas/M/1.NC siRNA/NC siRNA 2.tif]

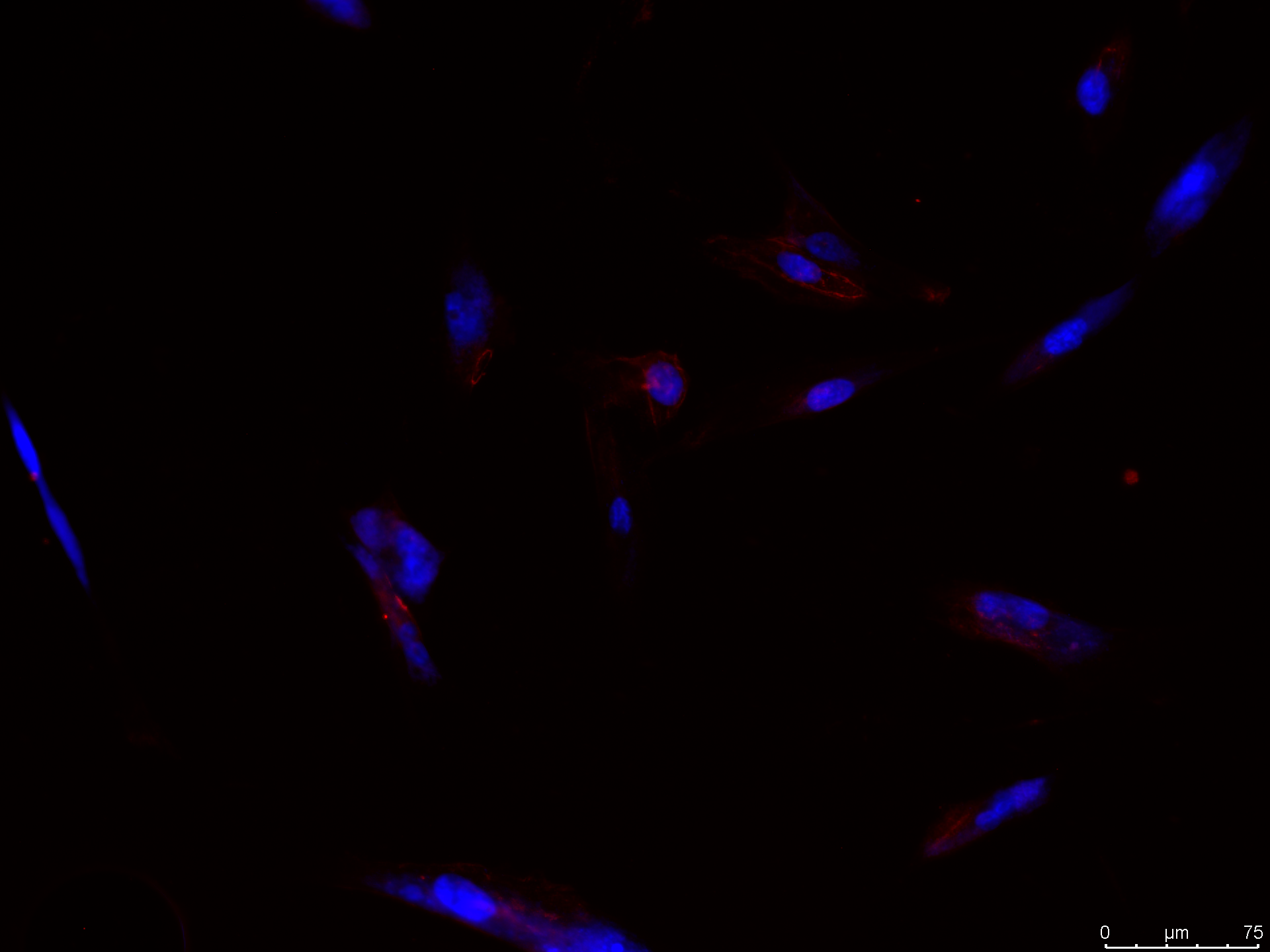

Supplement: Supplementary file 6 [file DataSheet_6.zip › Figure 6 raw datas/M/1.NC siRNA/NC siRNA 3.tif]

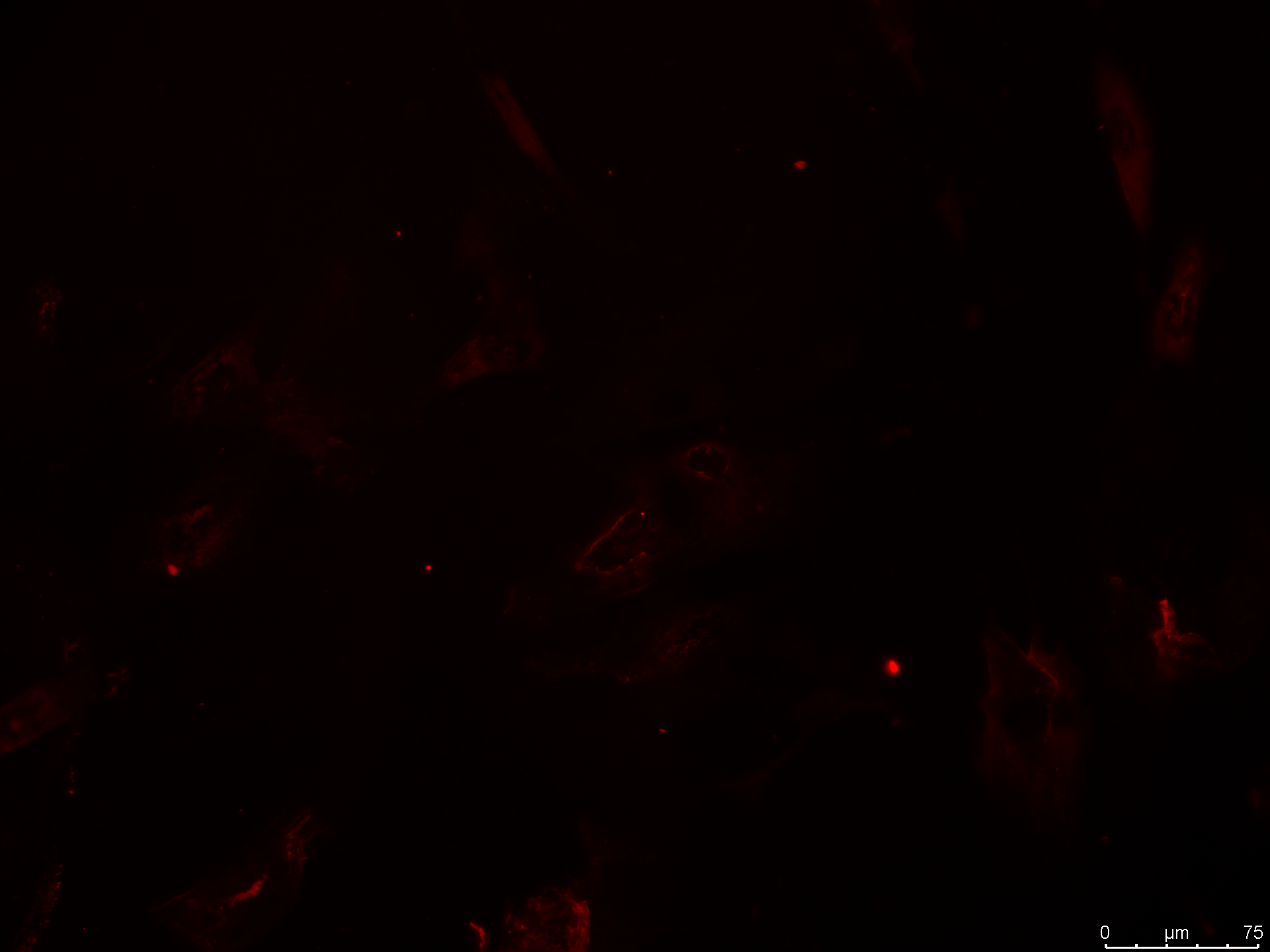

Supplement: Supplementary file 6 [file DataSheet_6.zip › Figure 6 raw datas/M/2.SDC-1 siRNA 1/SDC-1 siRNA 11.tif]

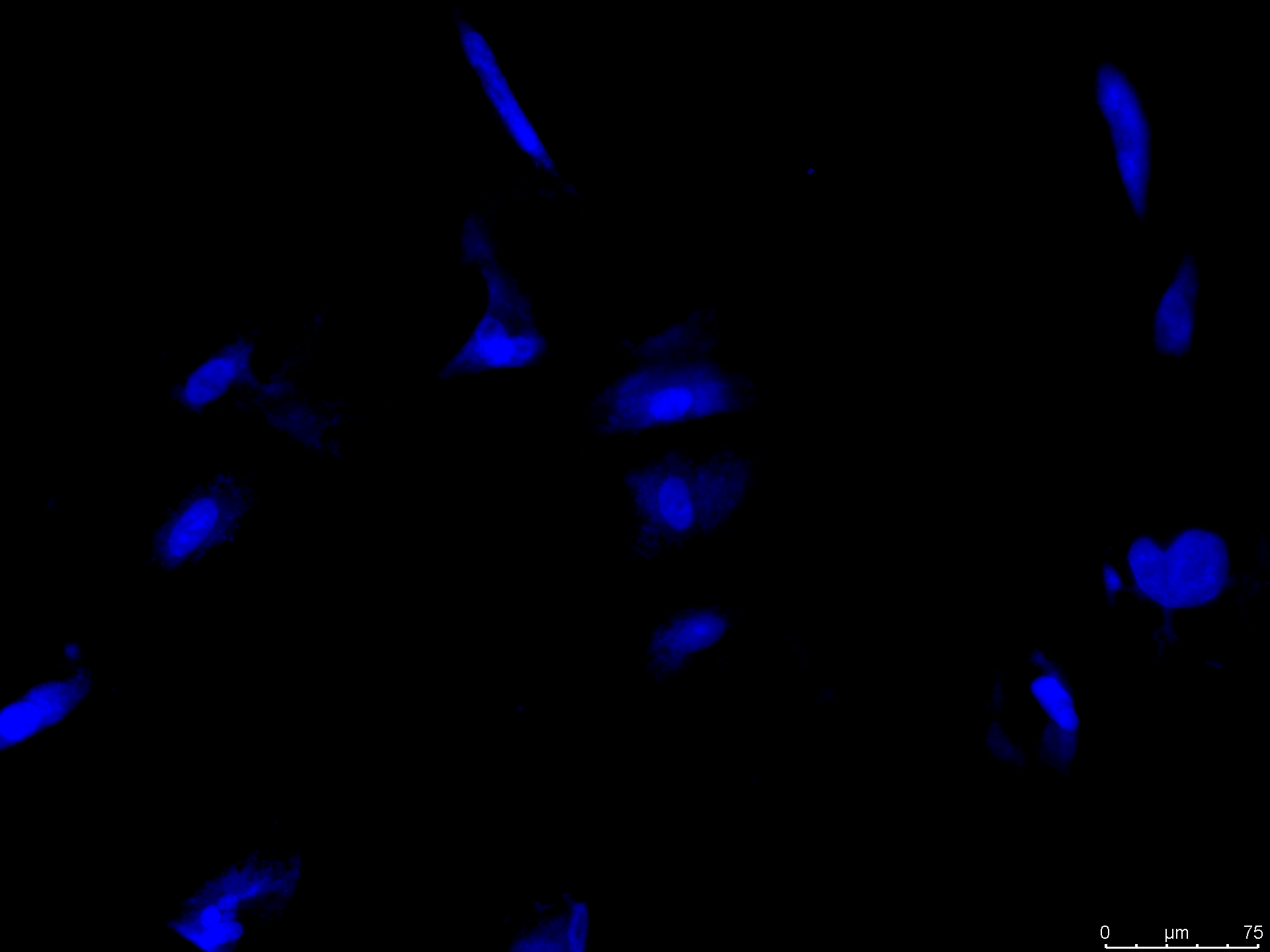

Supplement: Supplementary file 6 [file DataSheet_6.zip › Figure 6 raw datas/M/2.SDC-1 siRNA 1/SDC-1 siRNA 12.tif]

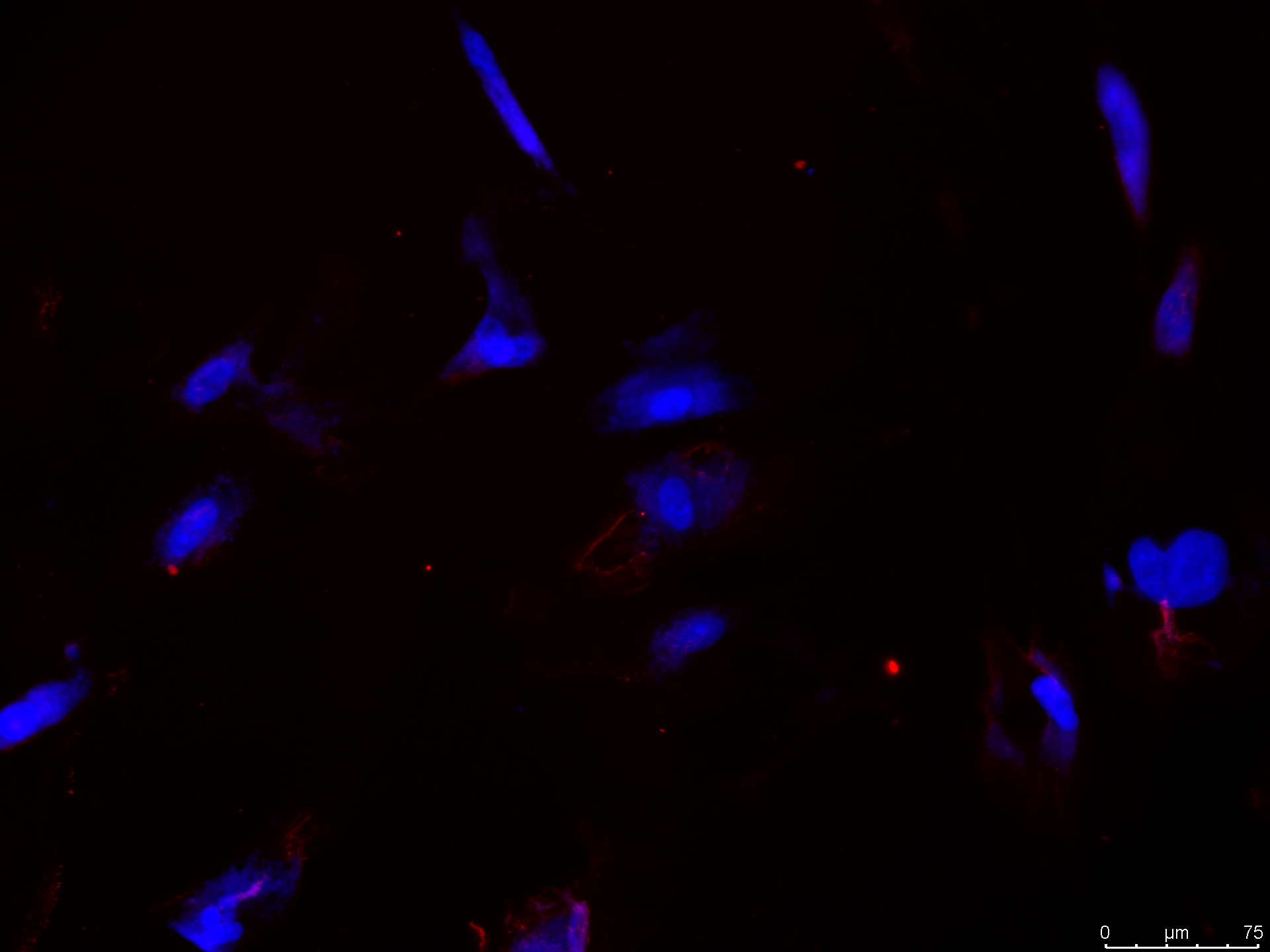

Supplement: Supplementary file 6 [file DataSheet_6.zip › Figure 6 raw datas/M/2.SDC-1 siRNA 1/SDC-1 siRNA 13.tif]

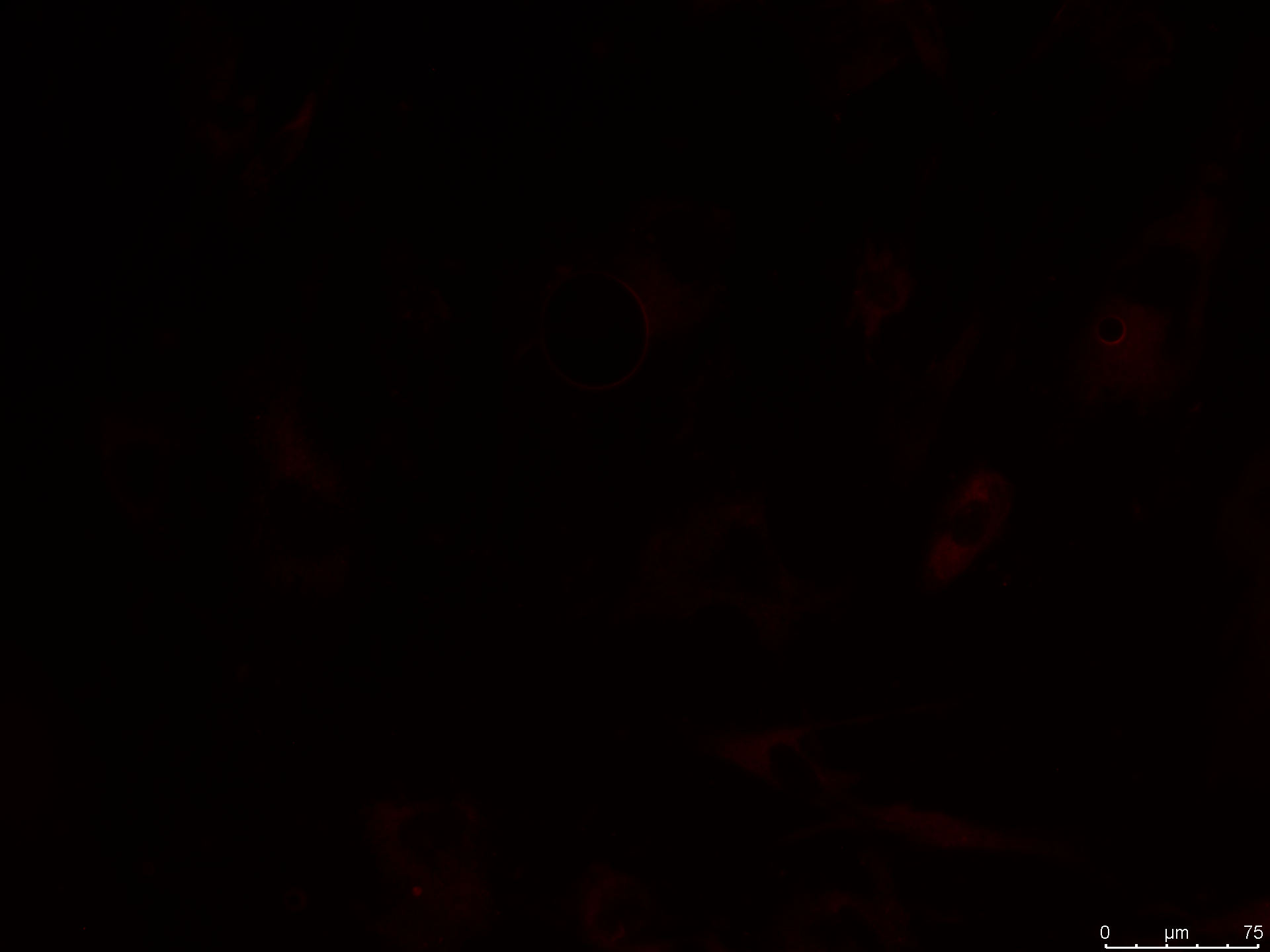

Supplement: Supplementary file 6 [file DataSheet_6.zip › Figure 6 raw datas/M/3.SDC-1 siRNA 2/SDC-1 siRNA 21.tif]

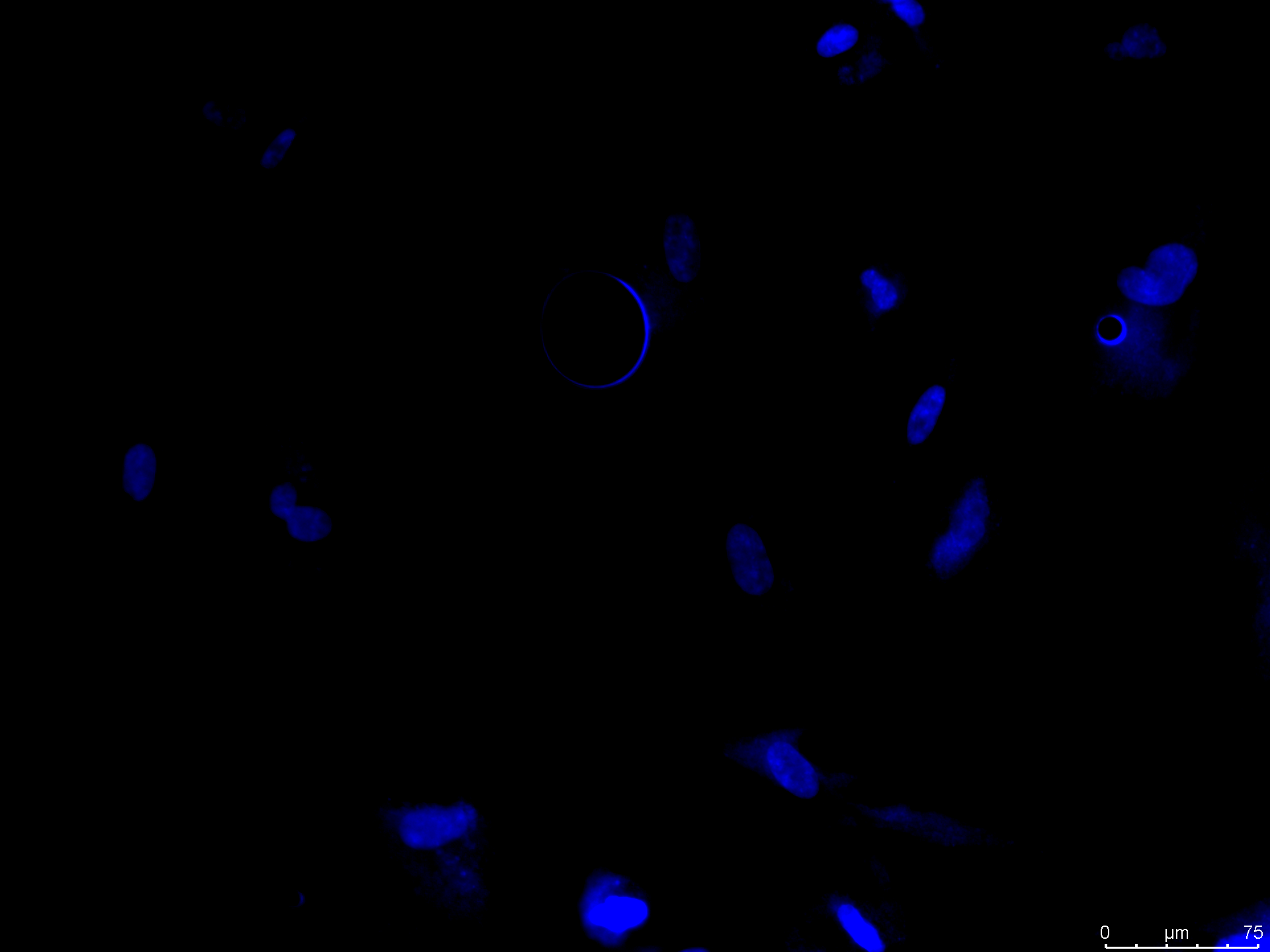

Supplement: Supplementary file 6 [file DataSheet_6.zip › Figure 6 raw datas/M/3.SDC-1 siRNA 2/SDC-1 siRNA 22.tif]

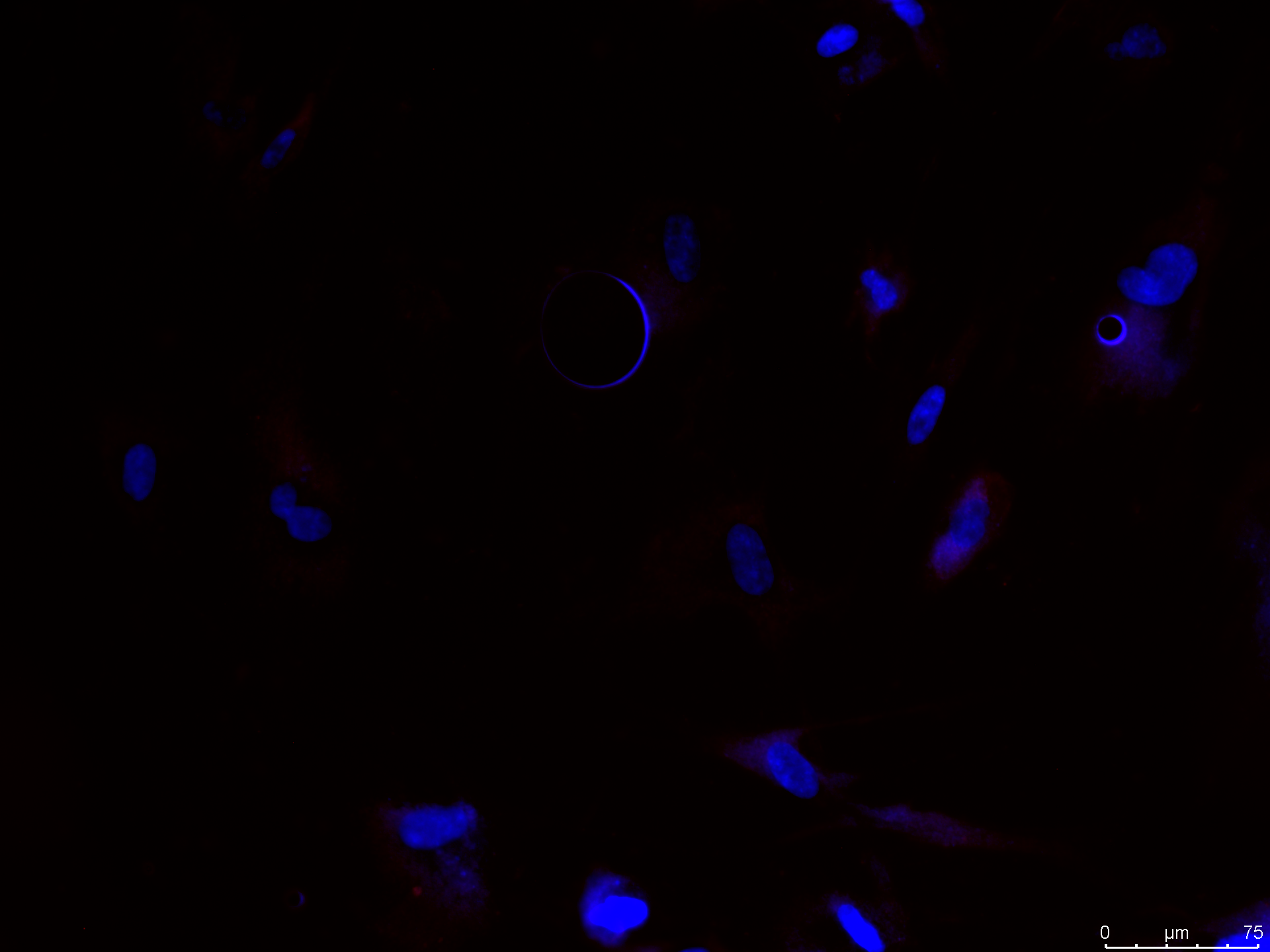

Supplement: Supplementary file 6 [file DataSheet_6.zip › Figure 6 raw datas/M/3.SDC-1 siRNA 2/SDC-1 siRNA 23.tif]

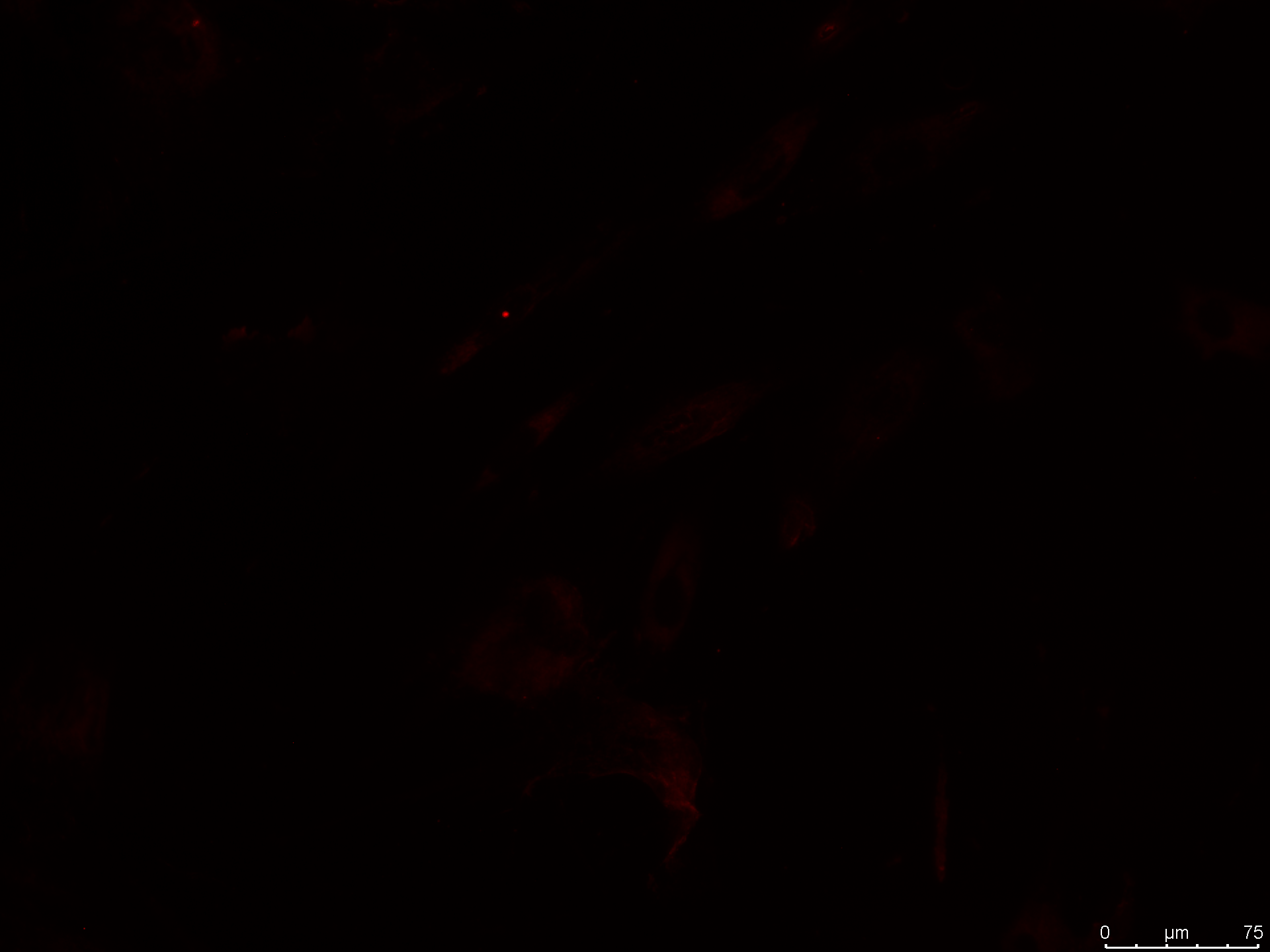

Supplement: Supplementary file 6 [file DataSheet_6.zip › Figure 6 raw datas/M/4.SDC-1 siRNA 3/SDC-1 siRNA 31.tif]

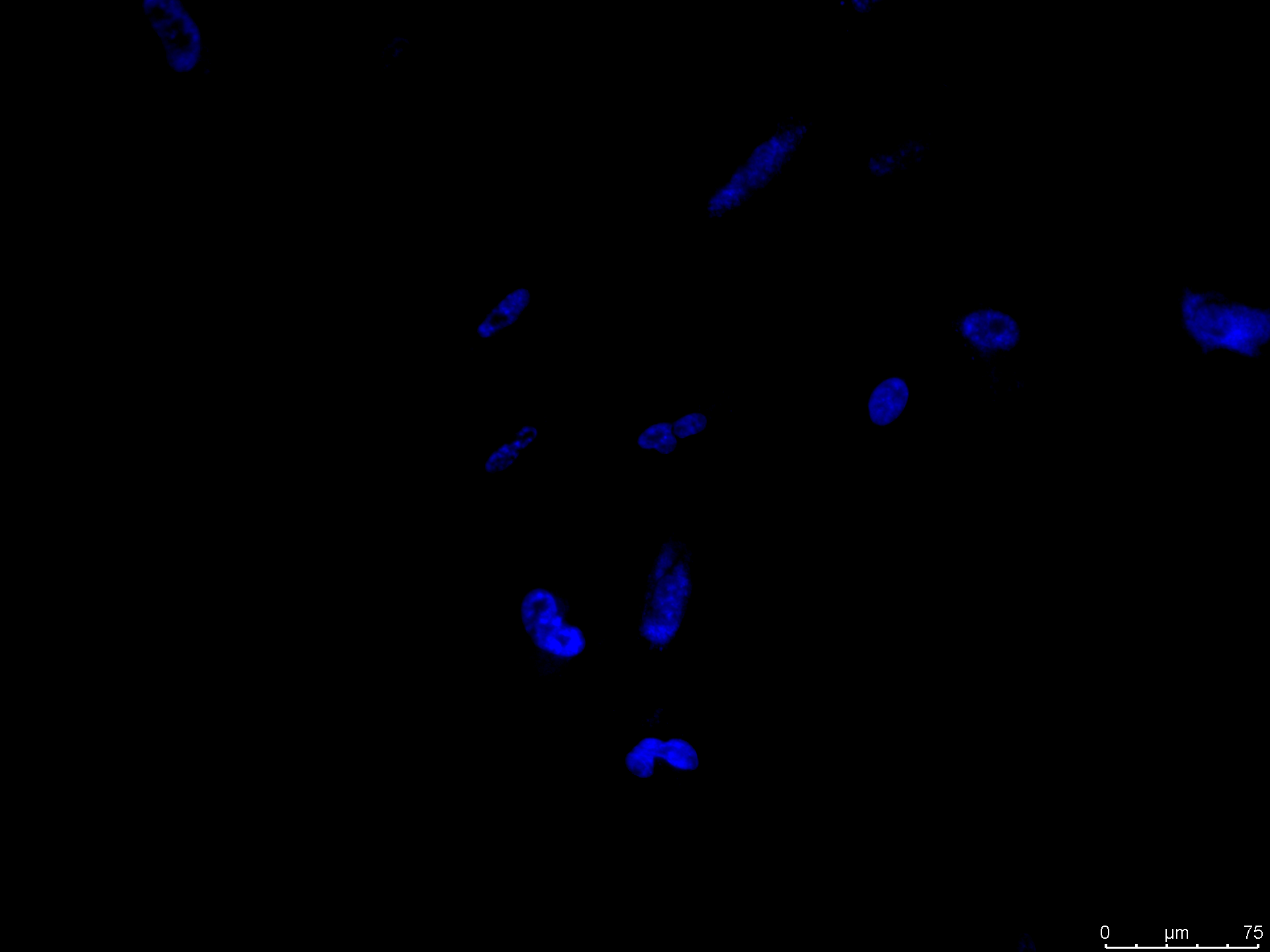

Supplement: Supplementary file 6 [file DataSheet_6.zip › Figure 6 raw datas/M/4.SDC-1 siRNA 3/SDC-1 siRNA 32.tif]

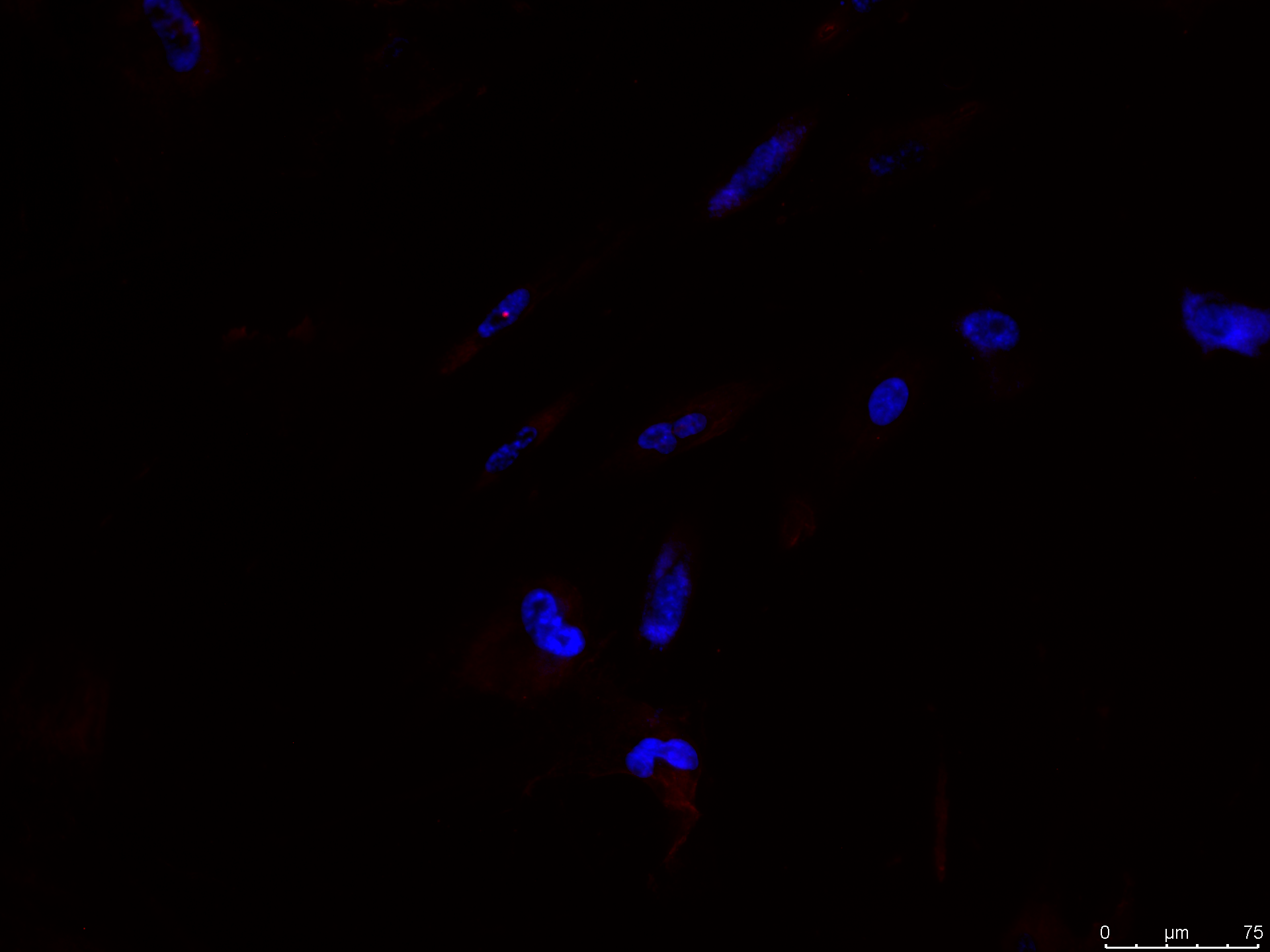

Supplement: Supplementary file 6 [file DataSheet_6.zip › Figure 6 raw datas/M/4.SDC-1 siRNA 3/SDC-1 siRNA 33.tif]

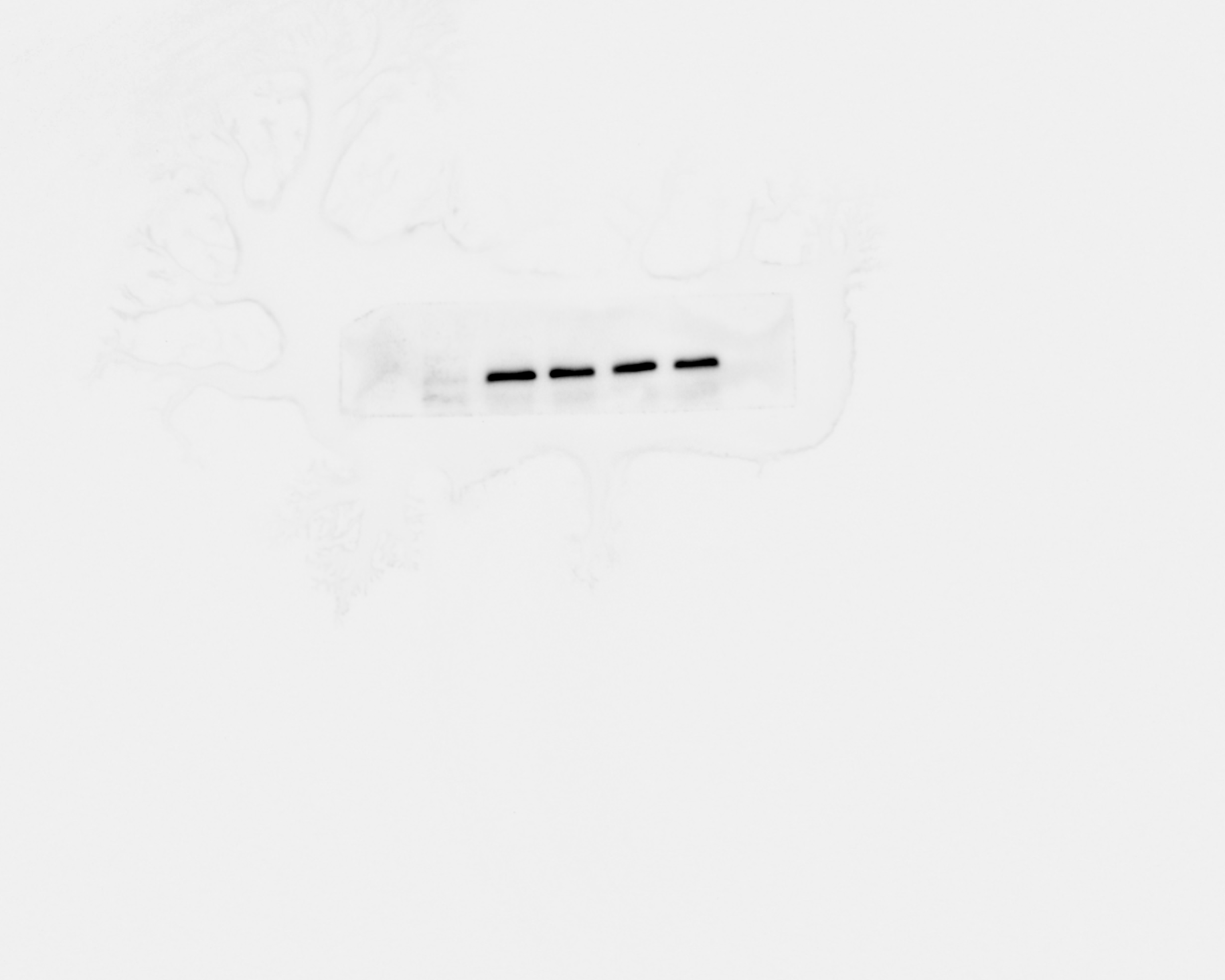

Supplement: Supplementary file 7 [file DataSheet_7.zip › Figure 7 raw datas/A B C D/A. p-smad3.tif]

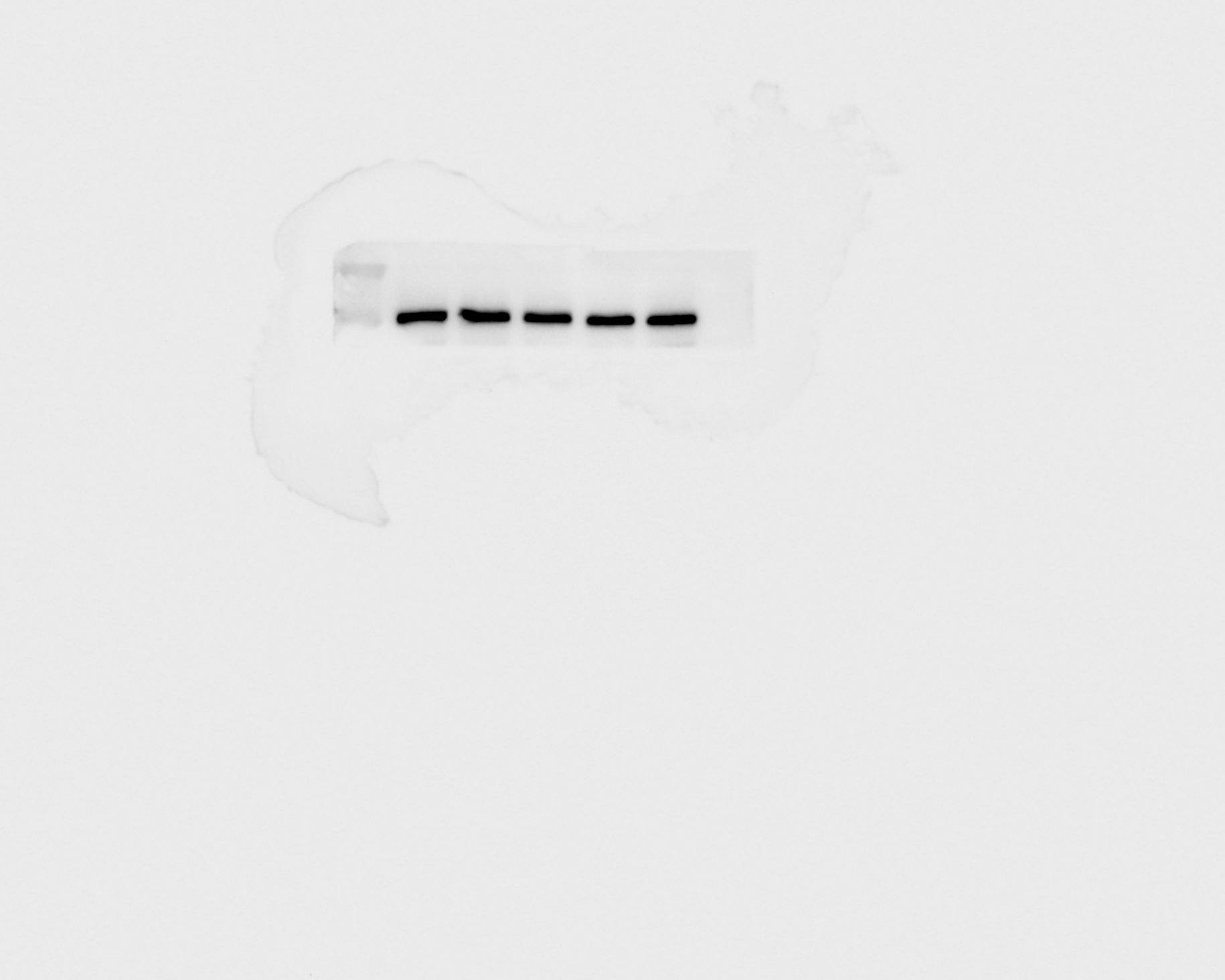

Supplement: Supplementary file 7 [file DataSheet_7.zip › Figure 7 raw datas/A B C D/A. smad3.tif]

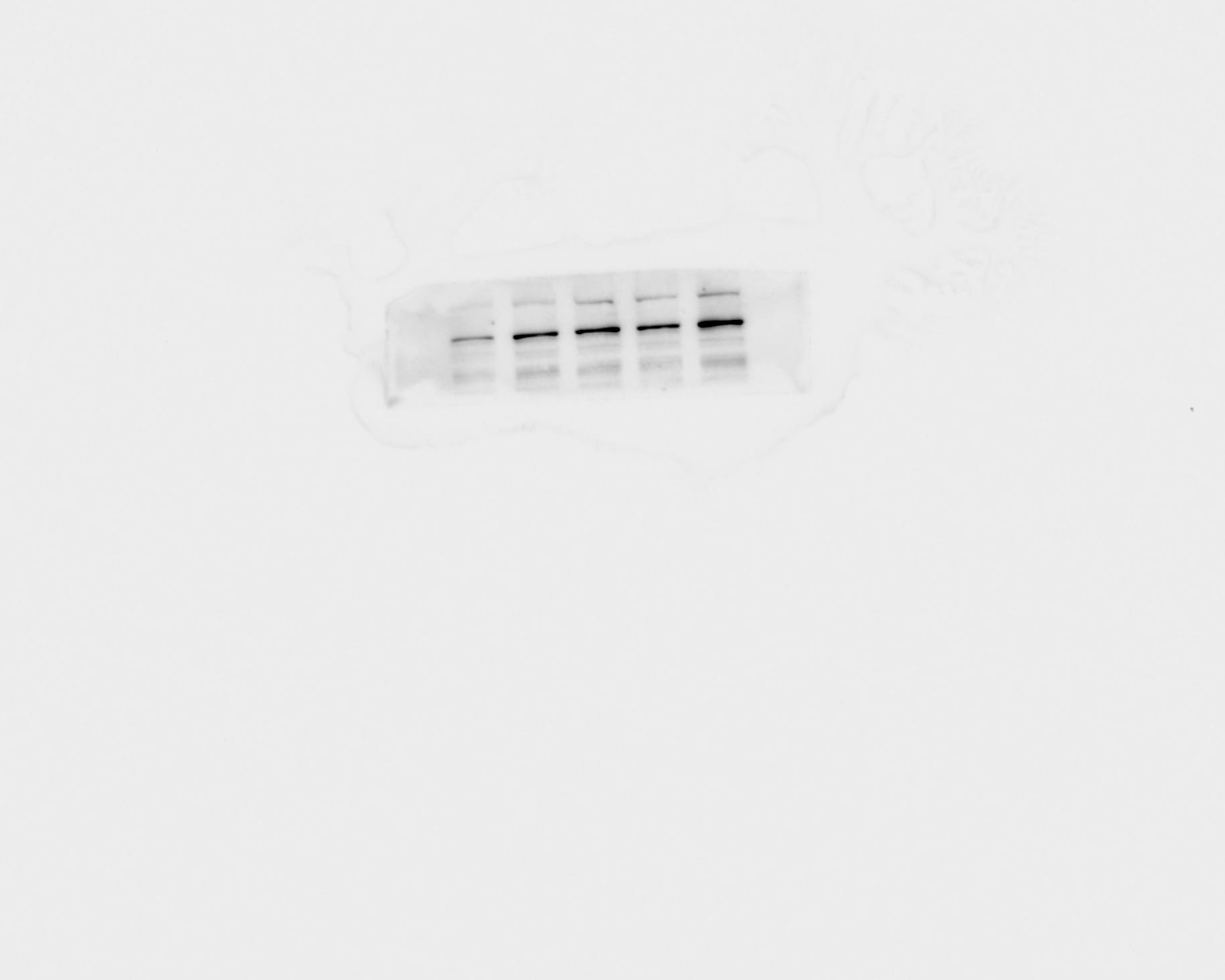

Supplement: Supplementary file 7 [file DataSheet_7.zip › Figure 7 raw datas/A B C D/B. Collagen I.tif]

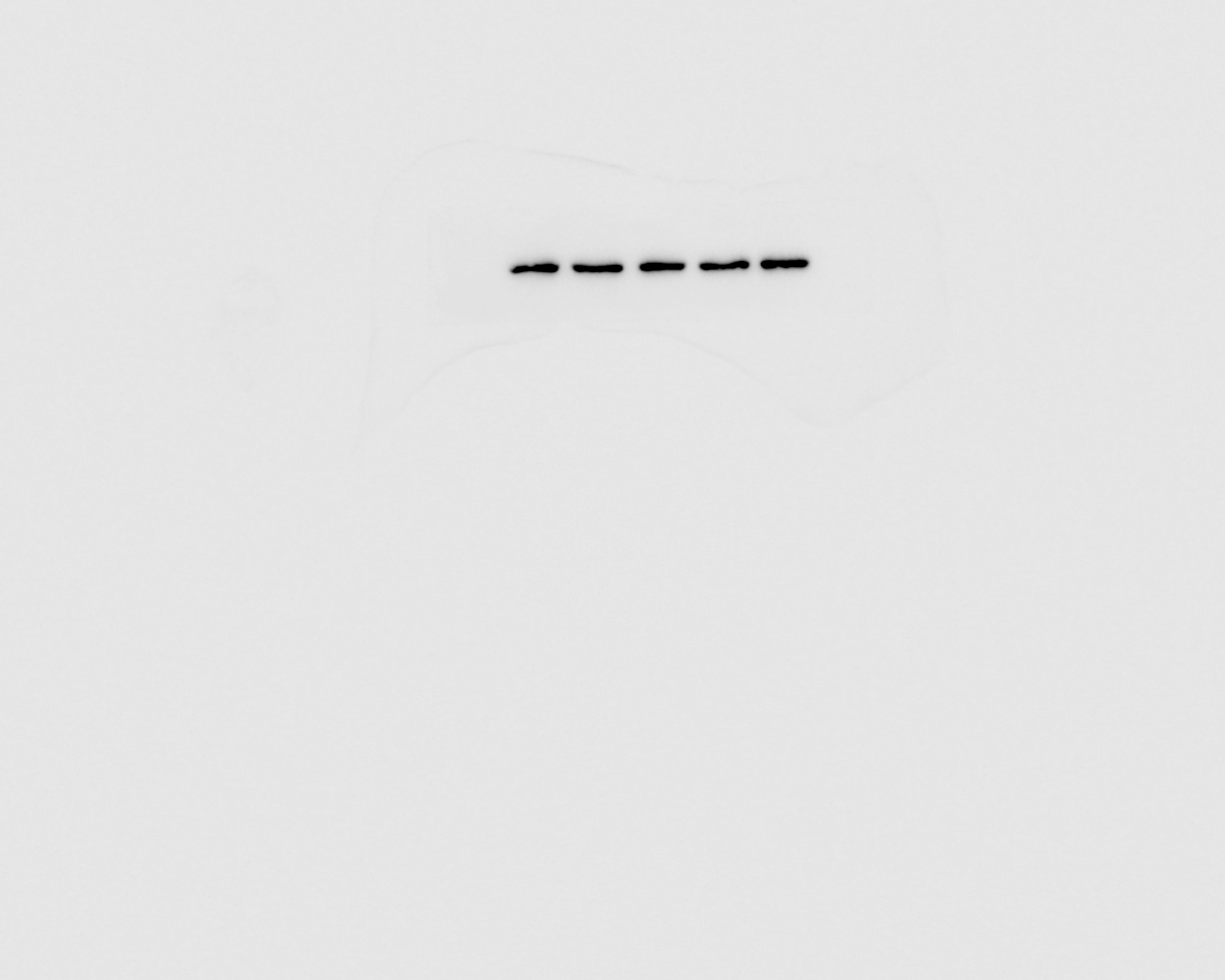

Supplement: Supplementary file 7 [file DataSheet_7.zip › Figure 7 raw datas/A B C D/B. GAPDH.tif]

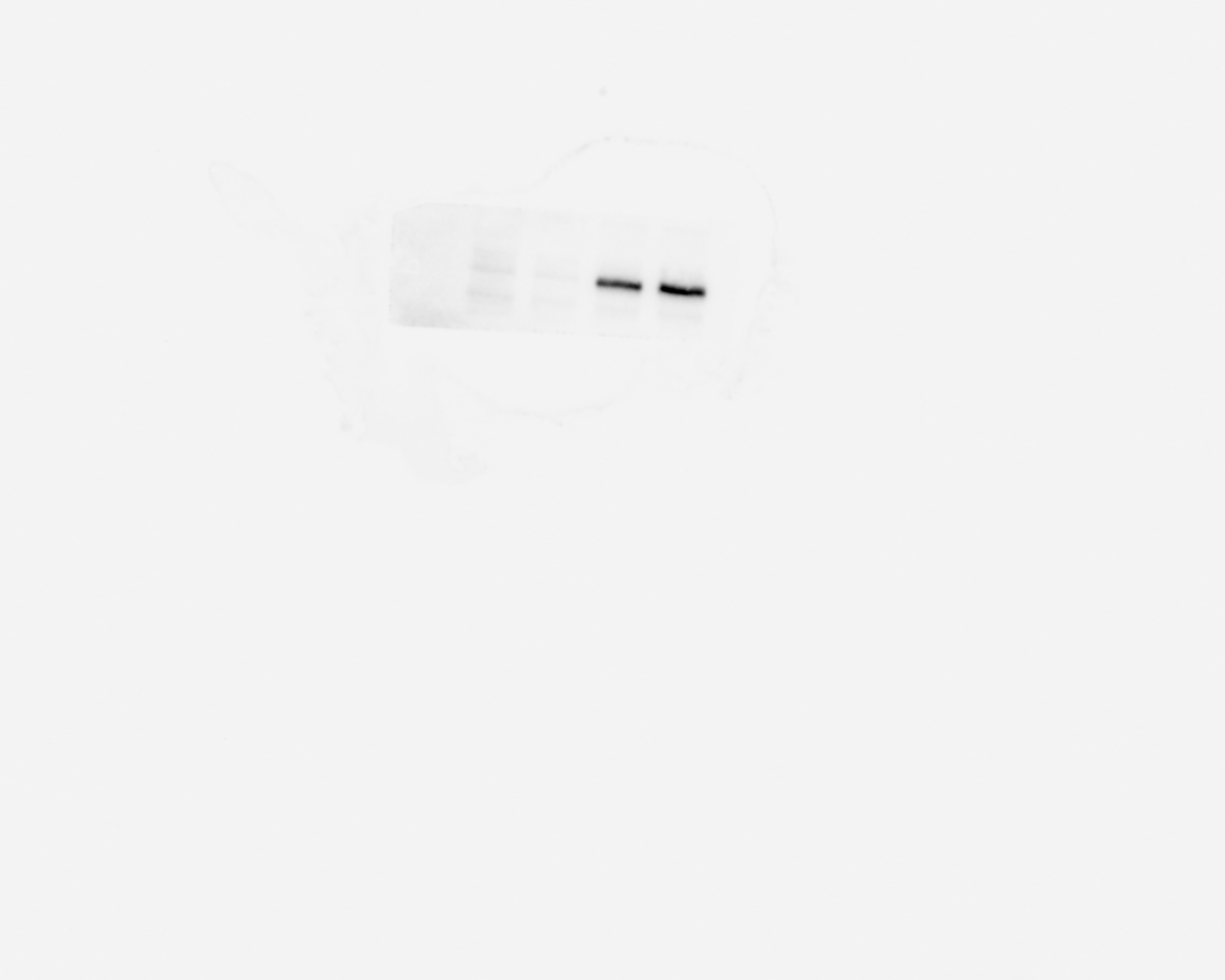

Supplement: Supplementary file 7 [file DataSheet_7.zip › Figure 7 raw datas/A B C D/C. p-smad3.tif]

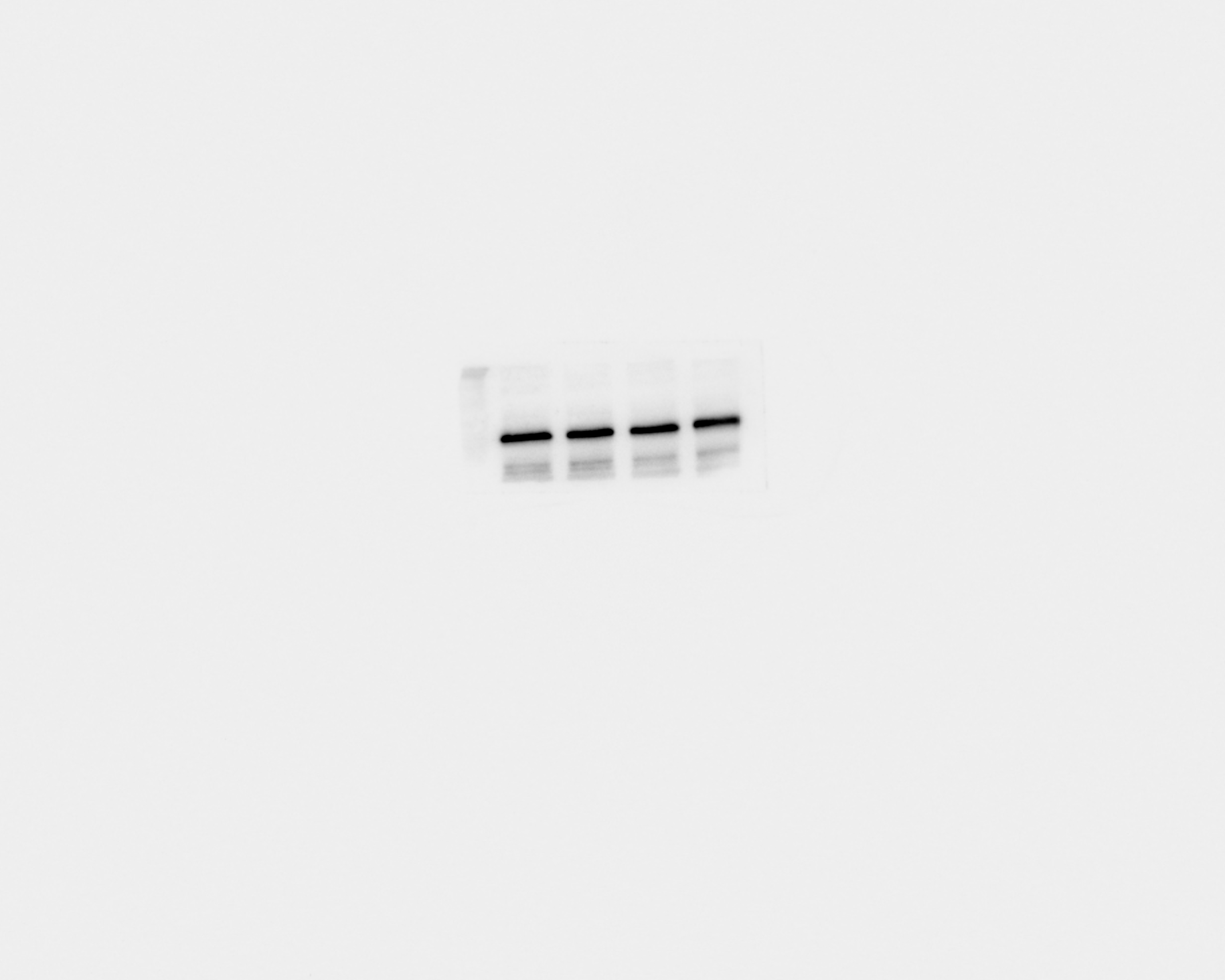

Supplement: Supplementary file 7 [file DataSheet_7.zip › Figure 7 raw datas/A B C D/C. smad3.tif]

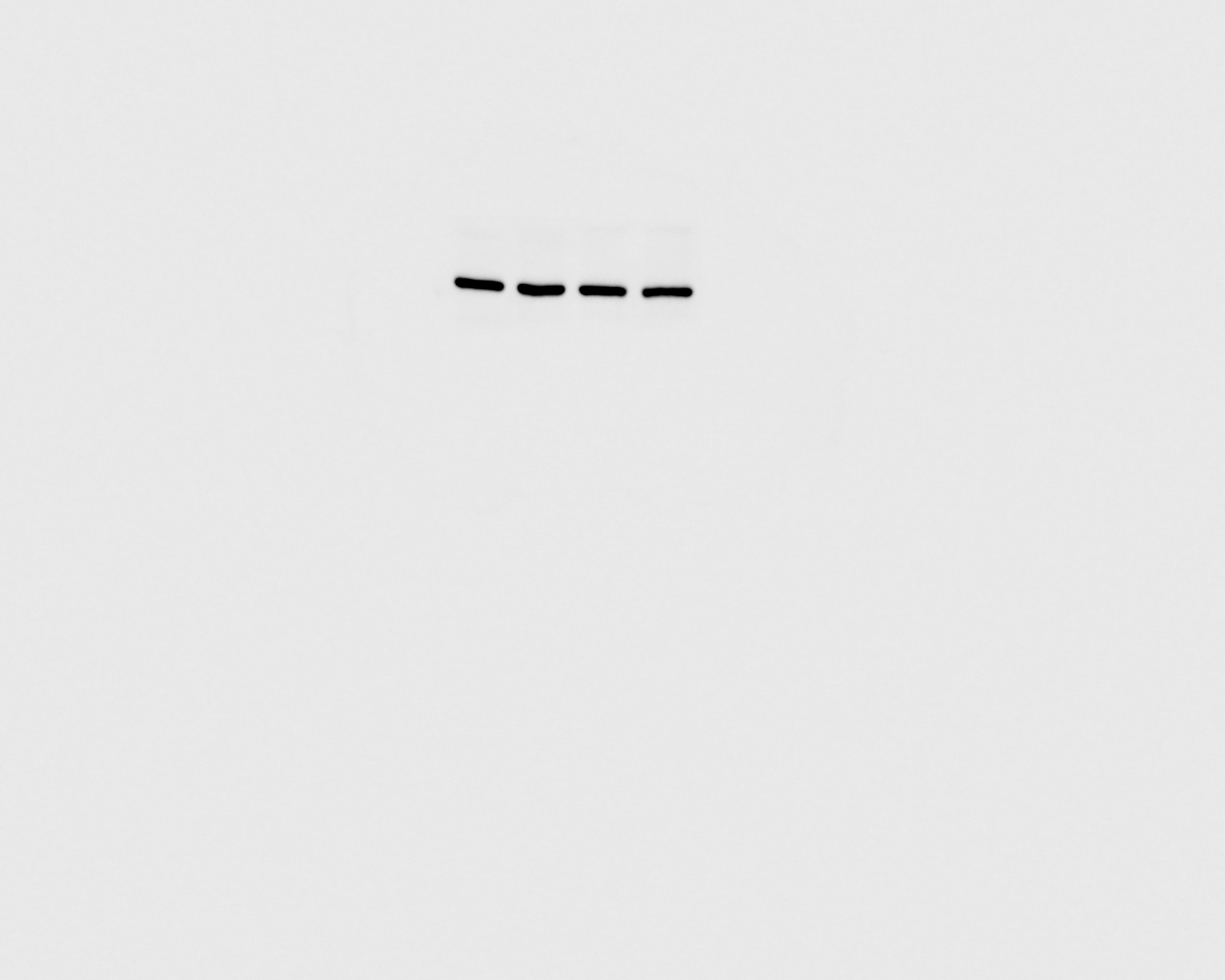

Supplement: Supplementary file 7 [file DataSheet_7.zip › Figure 7 raw datas/A B C D/D. GAPDH.tif]

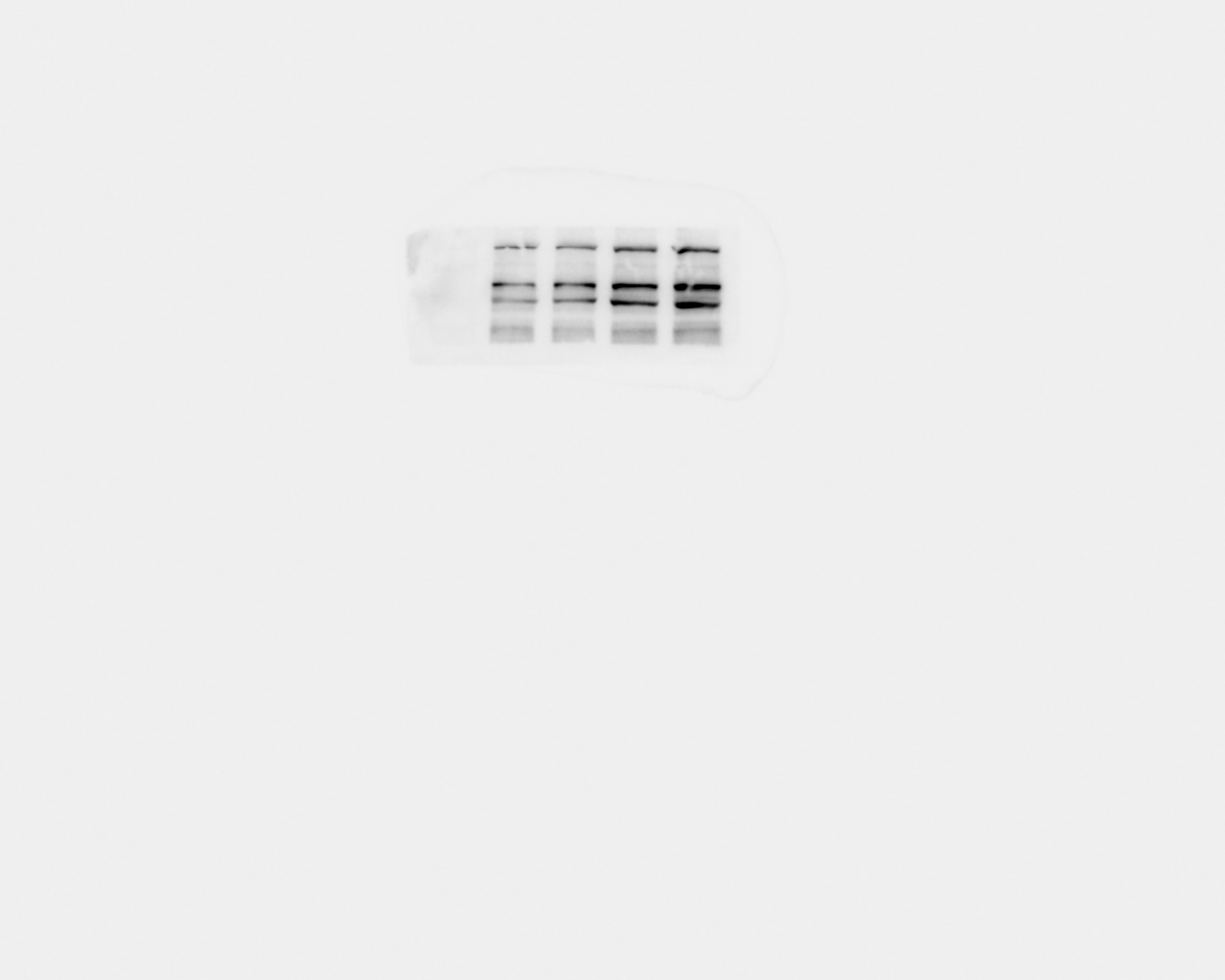

Supplement: Supplementary file 7 [file DataSheet_7.zip › Figure 7 raw datas/A B C D/D. collagen I.tif]

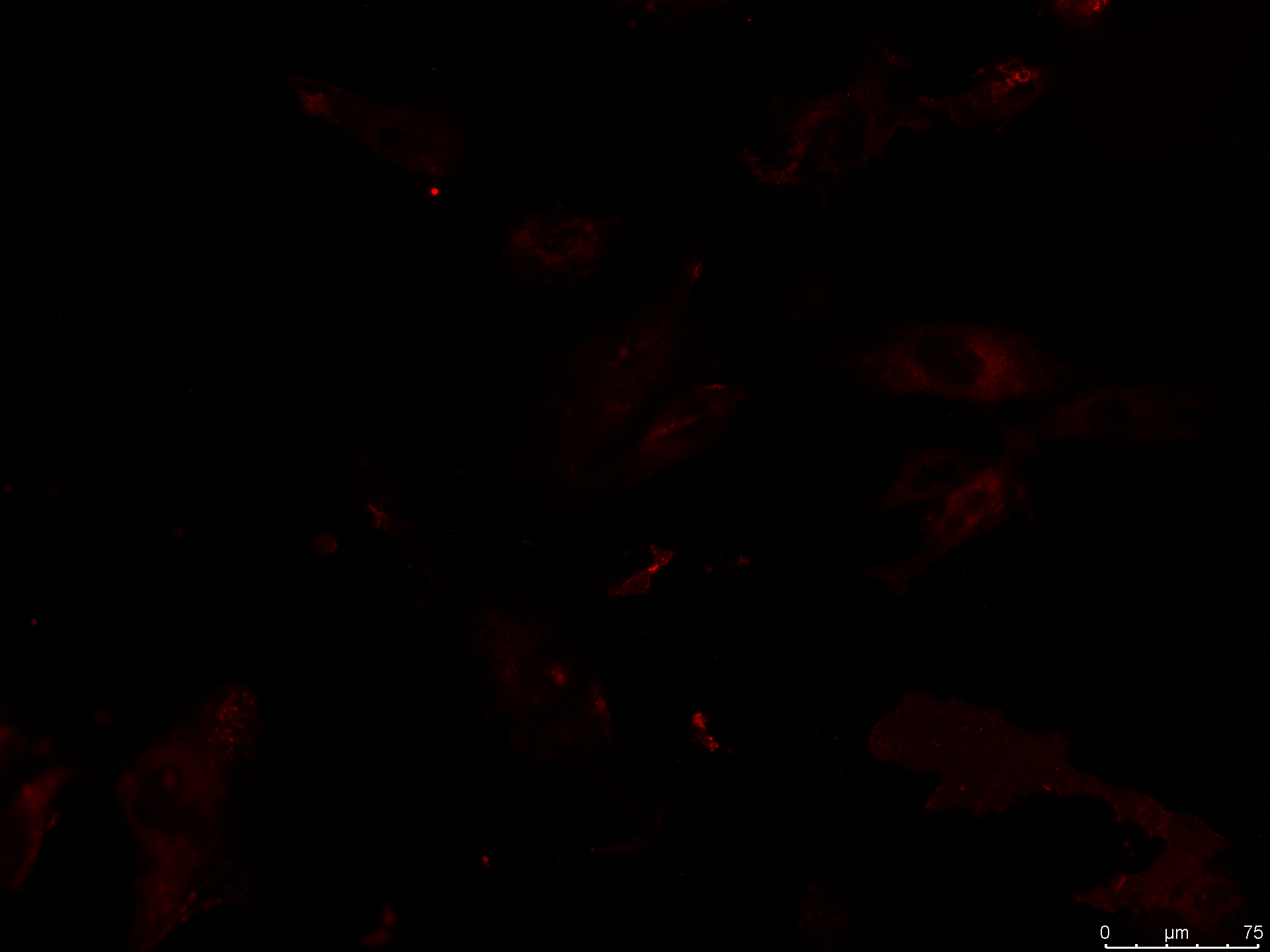

Supplement: Supplementary file 7 [file DataSheet_7.zip › Figure 7 raw datas/I/Control 1.tif]

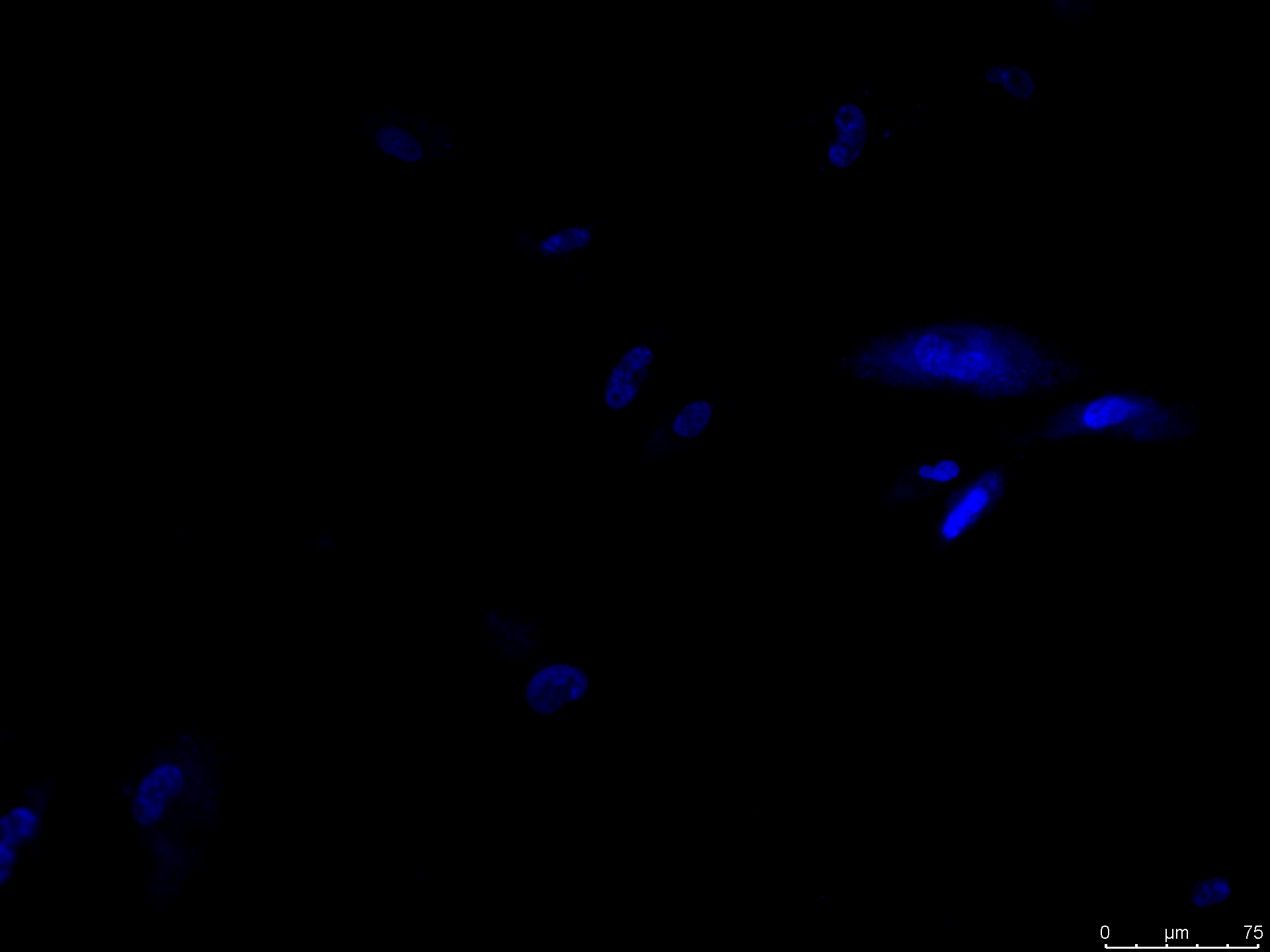

Supplement: Supplementary file 7 [file DataSheet_7.zip › Figure 7 raw datas/I/Control 2.tif]

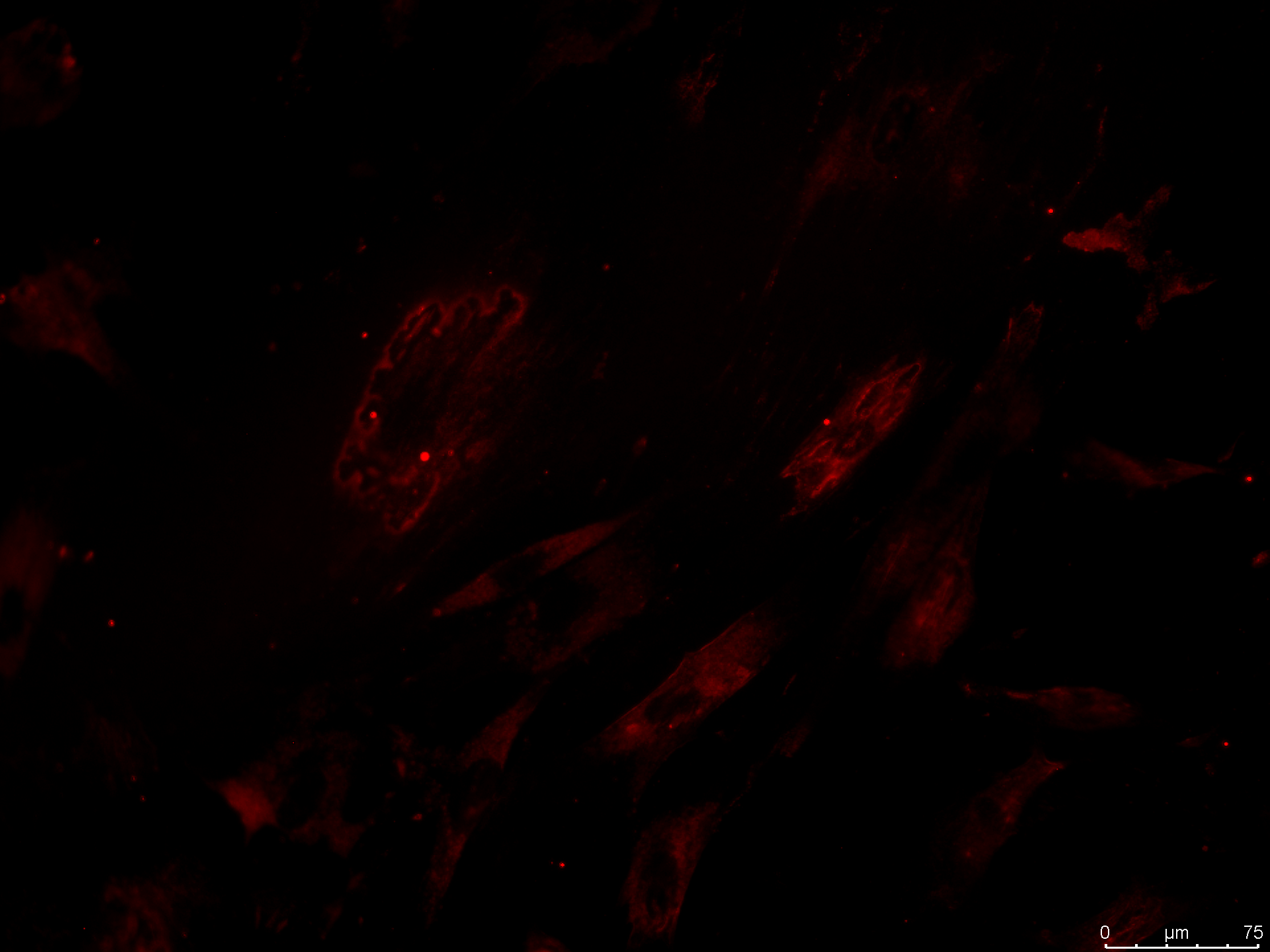

Supplement: Supplementary file 7 [file DataSheet_7.zip › Figure 7 raw datas/I/TGFB1 1.tif]

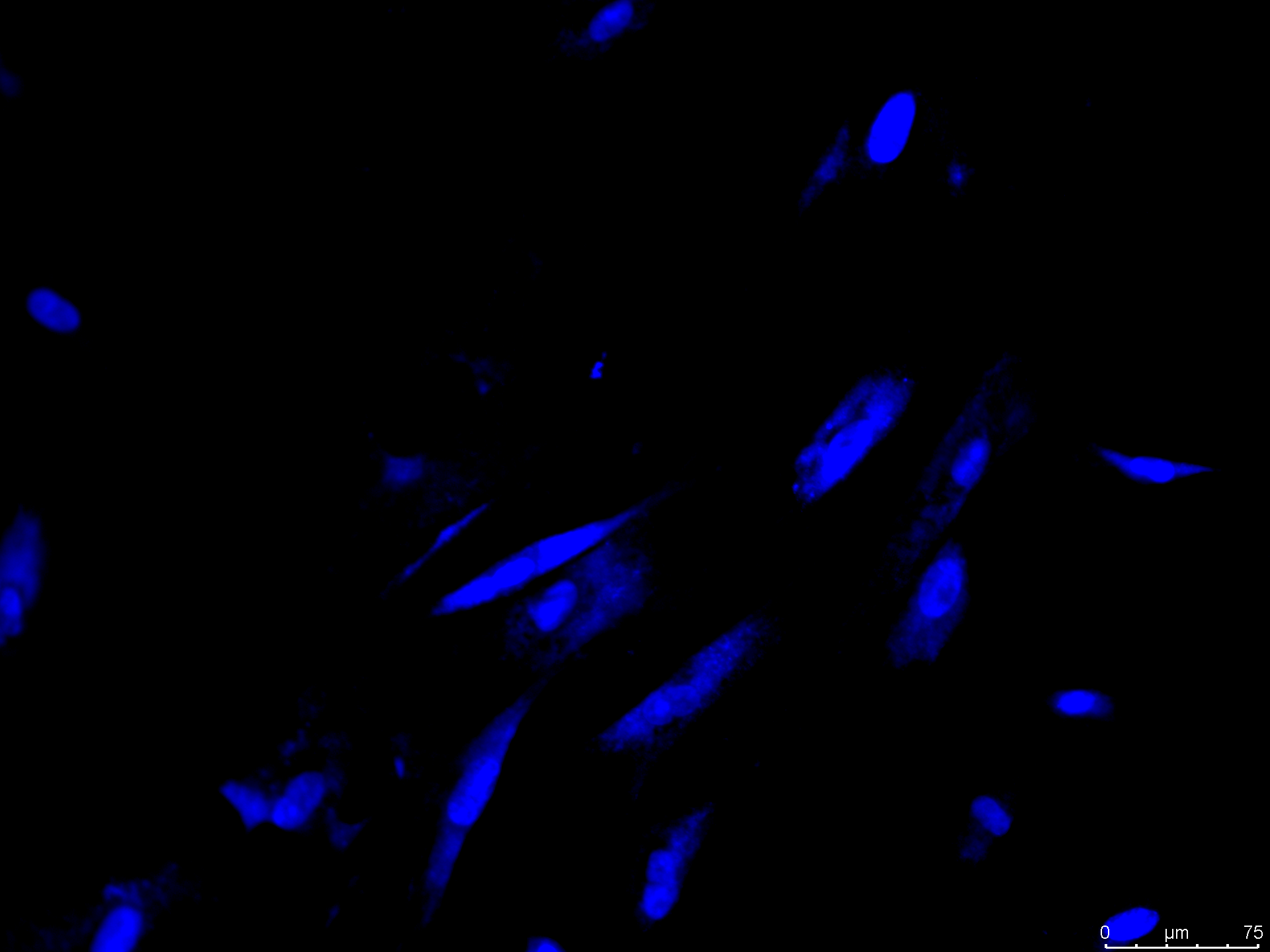

Supplement: Supplementary file 7 [file DataSheet_7.zip › Figure 7 raw datas/I/TGFB1 2.tif]

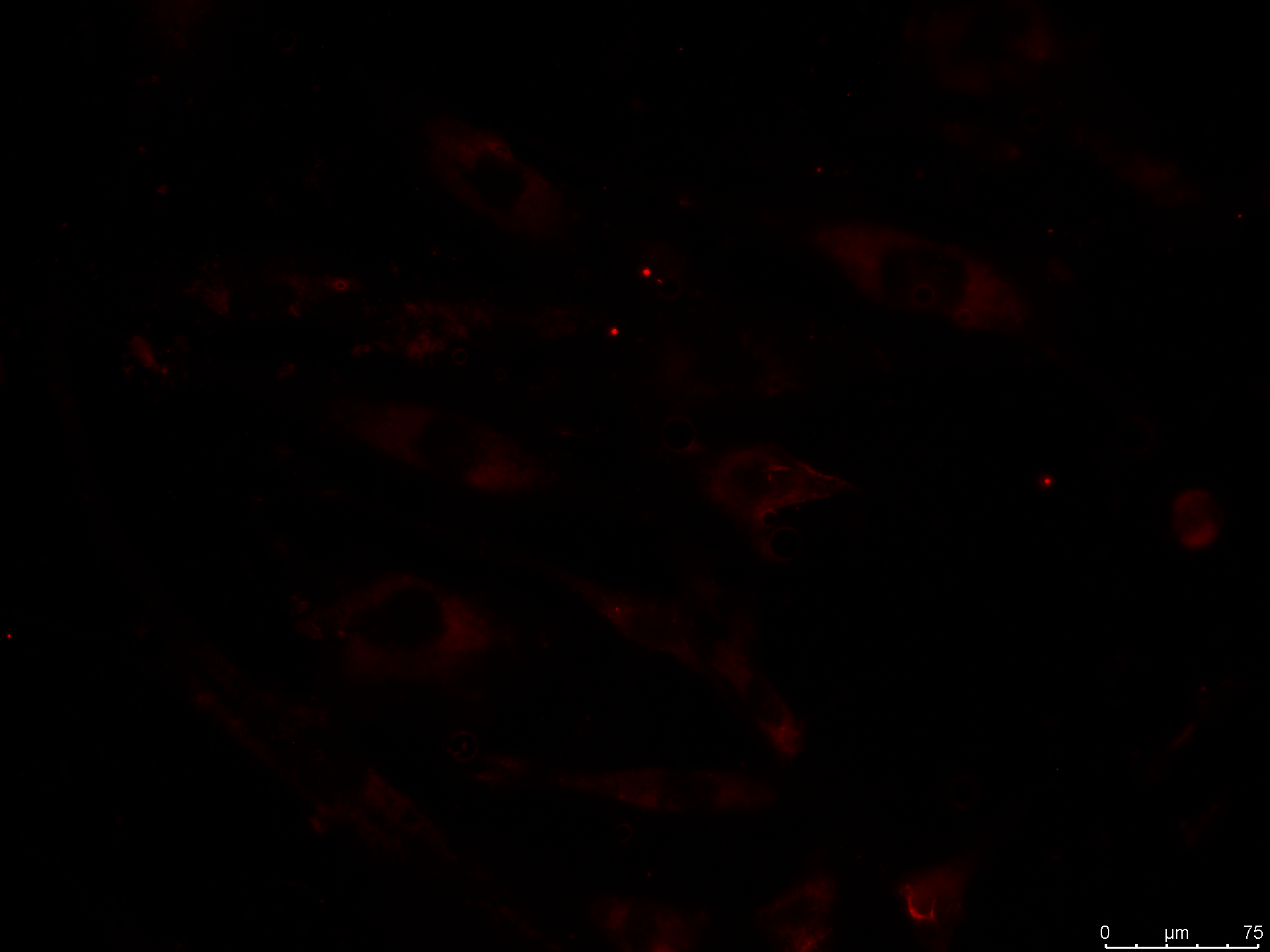

Supplement: Supplementary file 7 [file DataSheet_7.zip › Figure 7 raw datas/I/pcDNA3.1 1.tif]

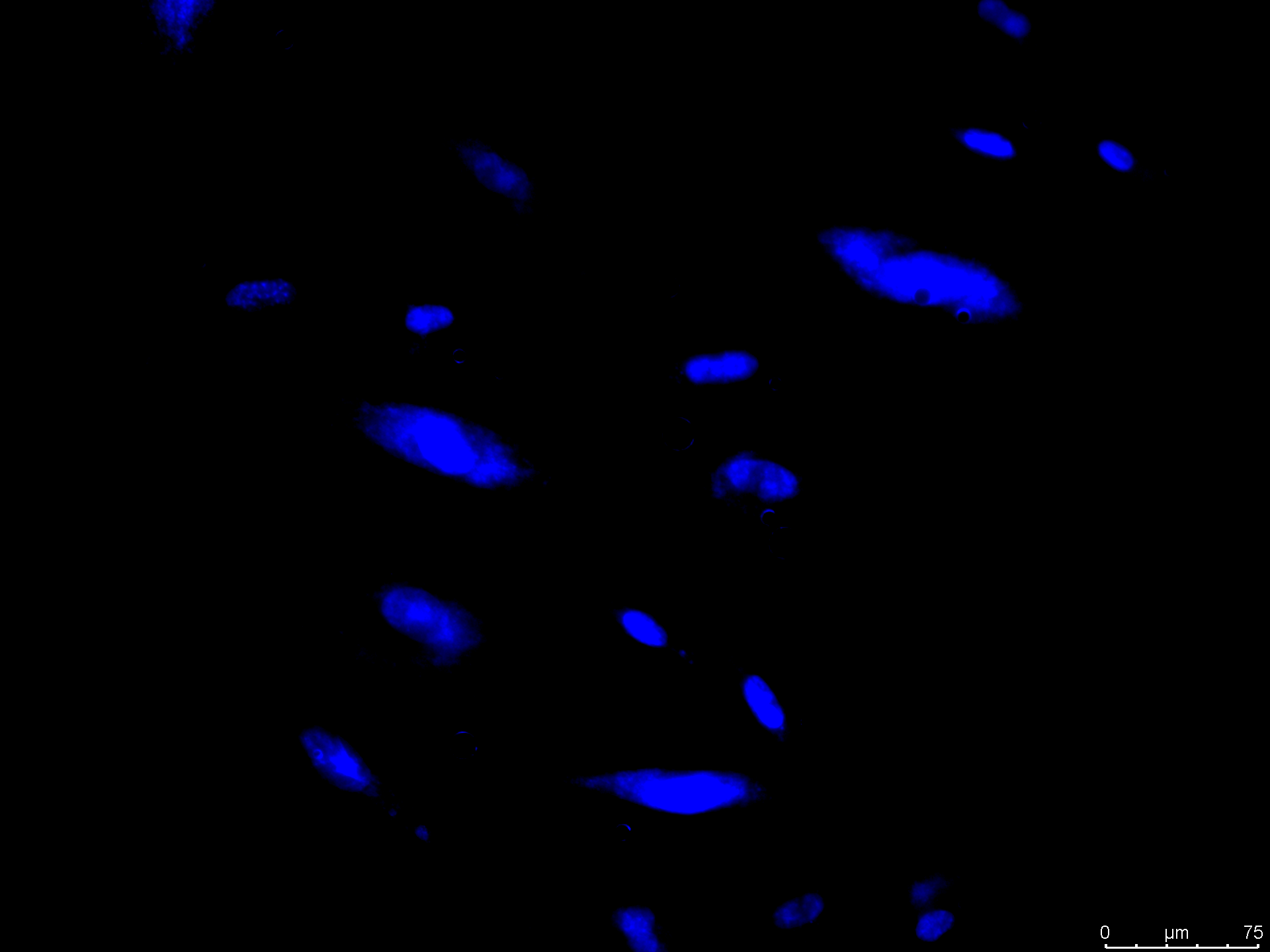

Supplement: Supplementary file 7 [file DataSheet_7.zip › Figure 7 raw datas/I/pcDNA3.1 2.tif]

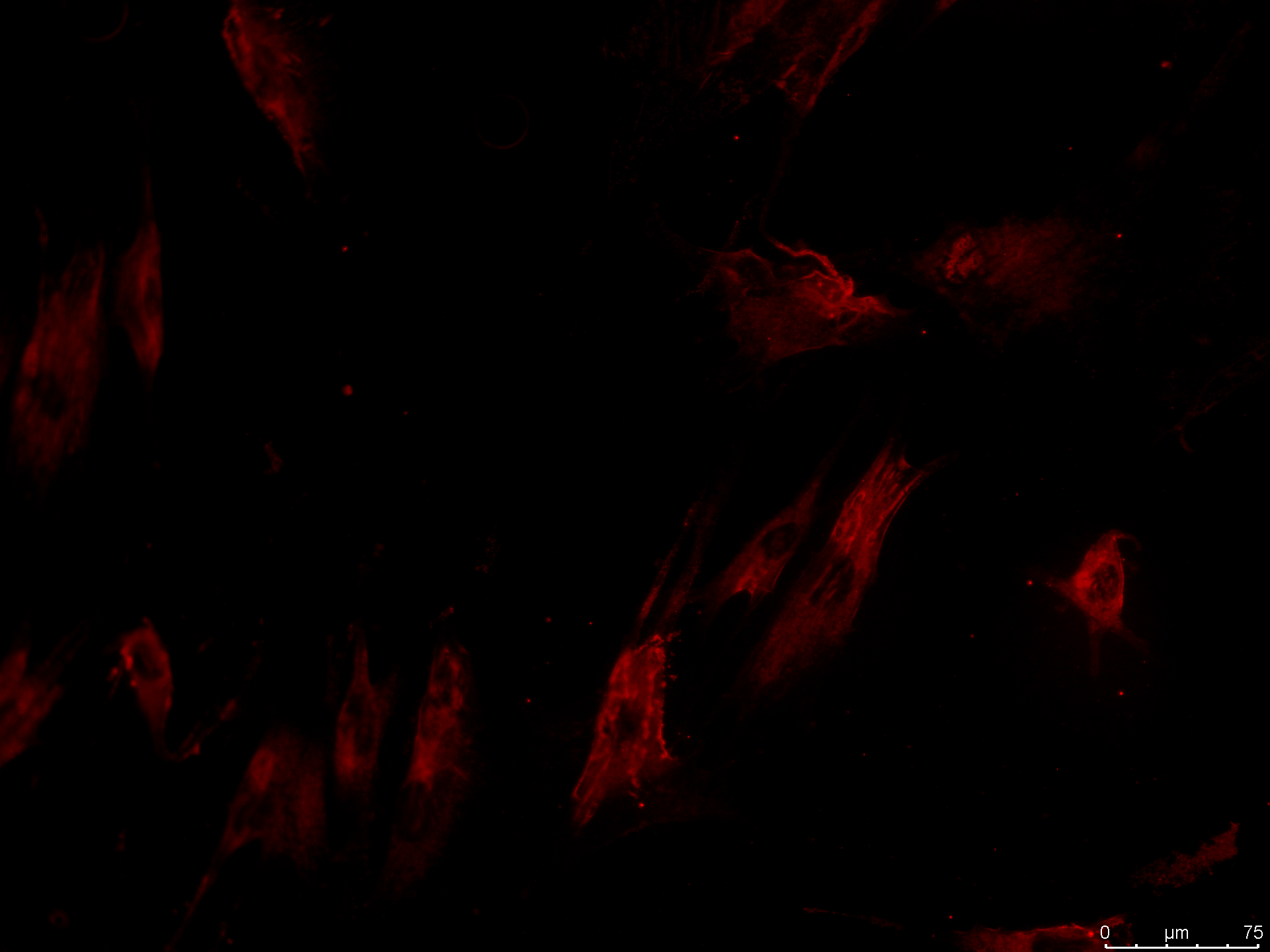

Supplement: Supplementary file 7 [file DataSheet_7.zip › Figure 7 raw datas/I/pcSDC-1+TGFB1 1.tif]

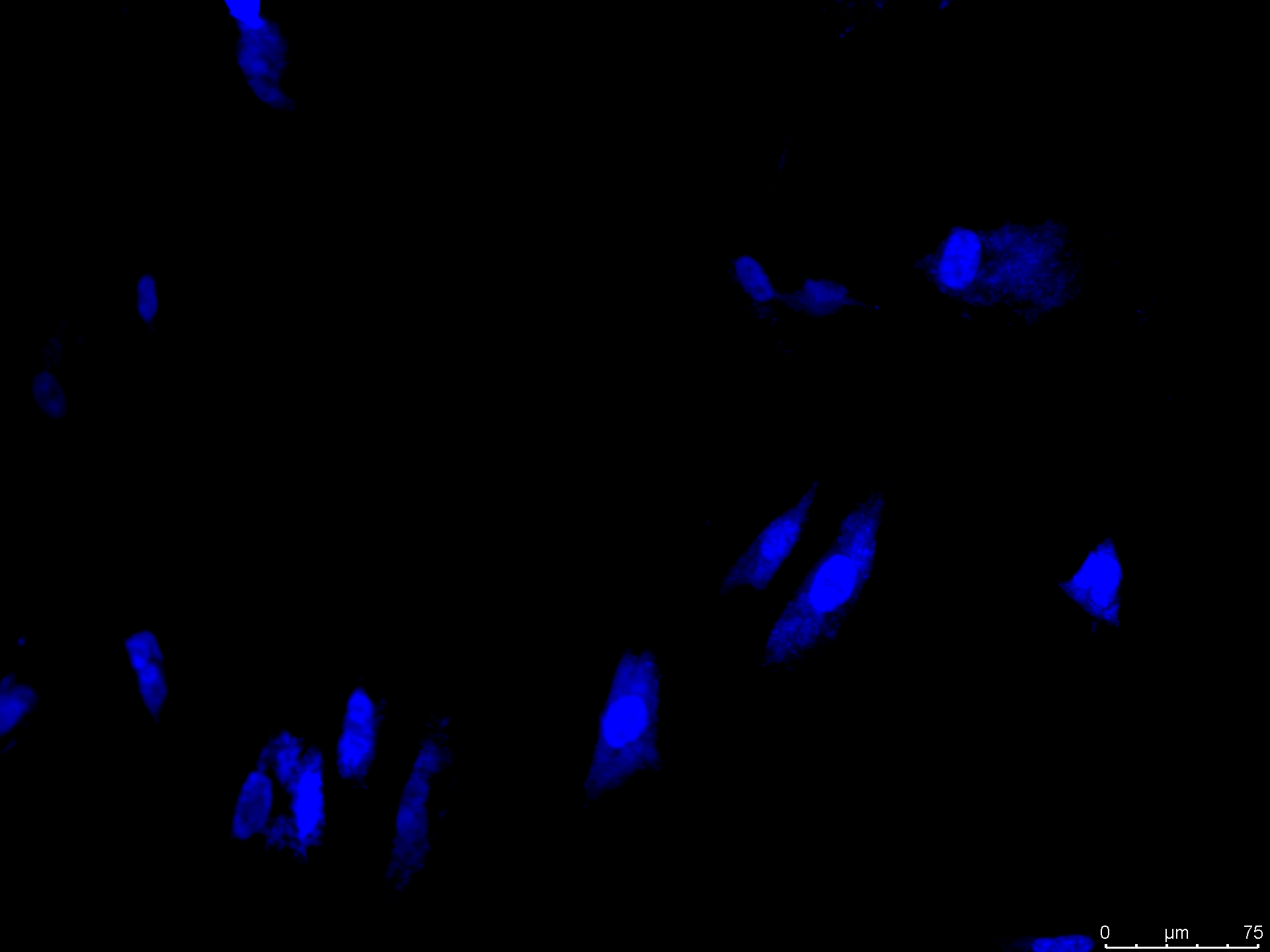

Supplement: Supplementary file 7 [file DataSheet_7.zip › Figure 7 raw datas/I/pcSDC-1+TGFB1 2.tif]

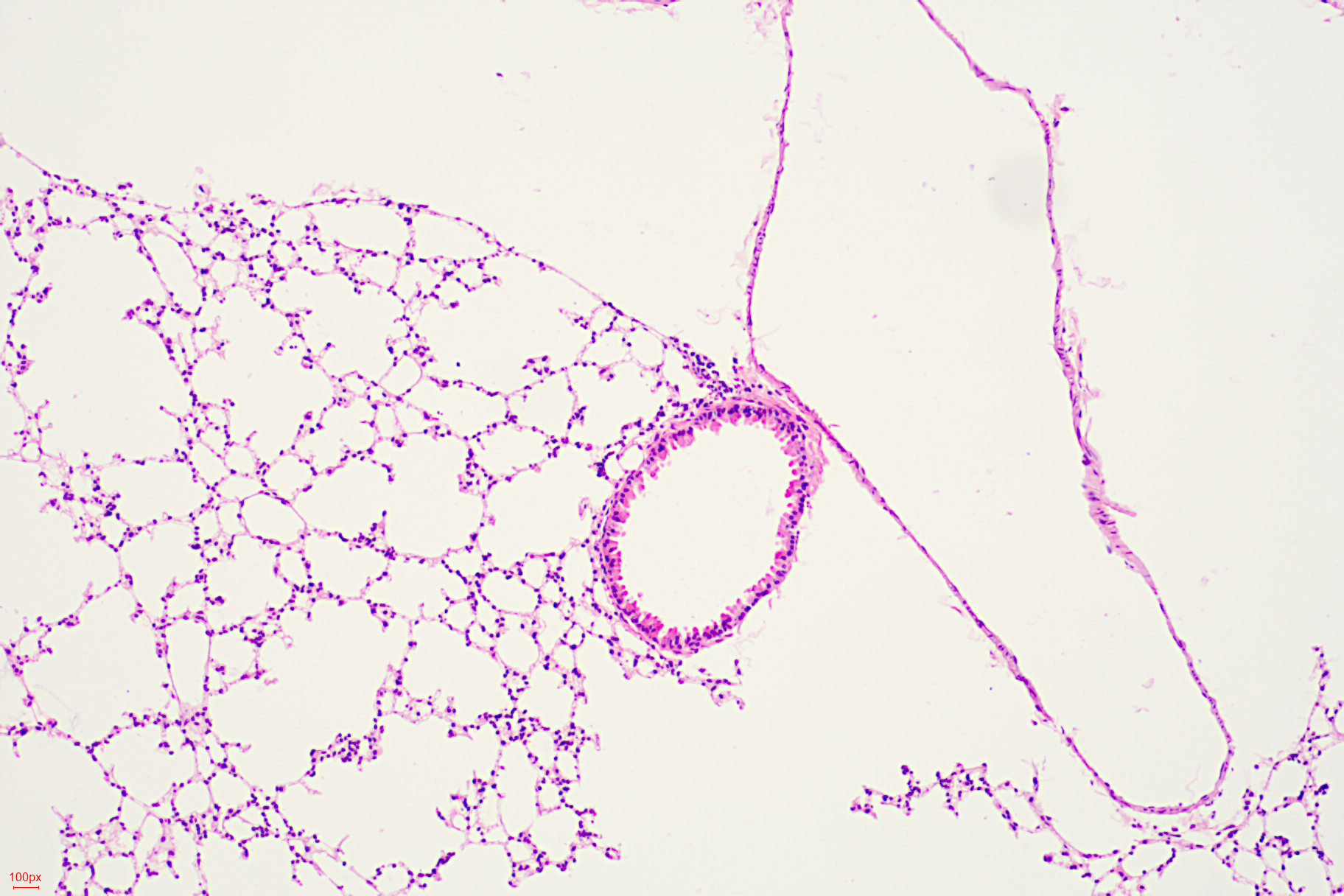

Supplement: Supplementary file 8 [file DataSheet_8.zip › Figure 8 raw datas/B. HE/Control.tif]

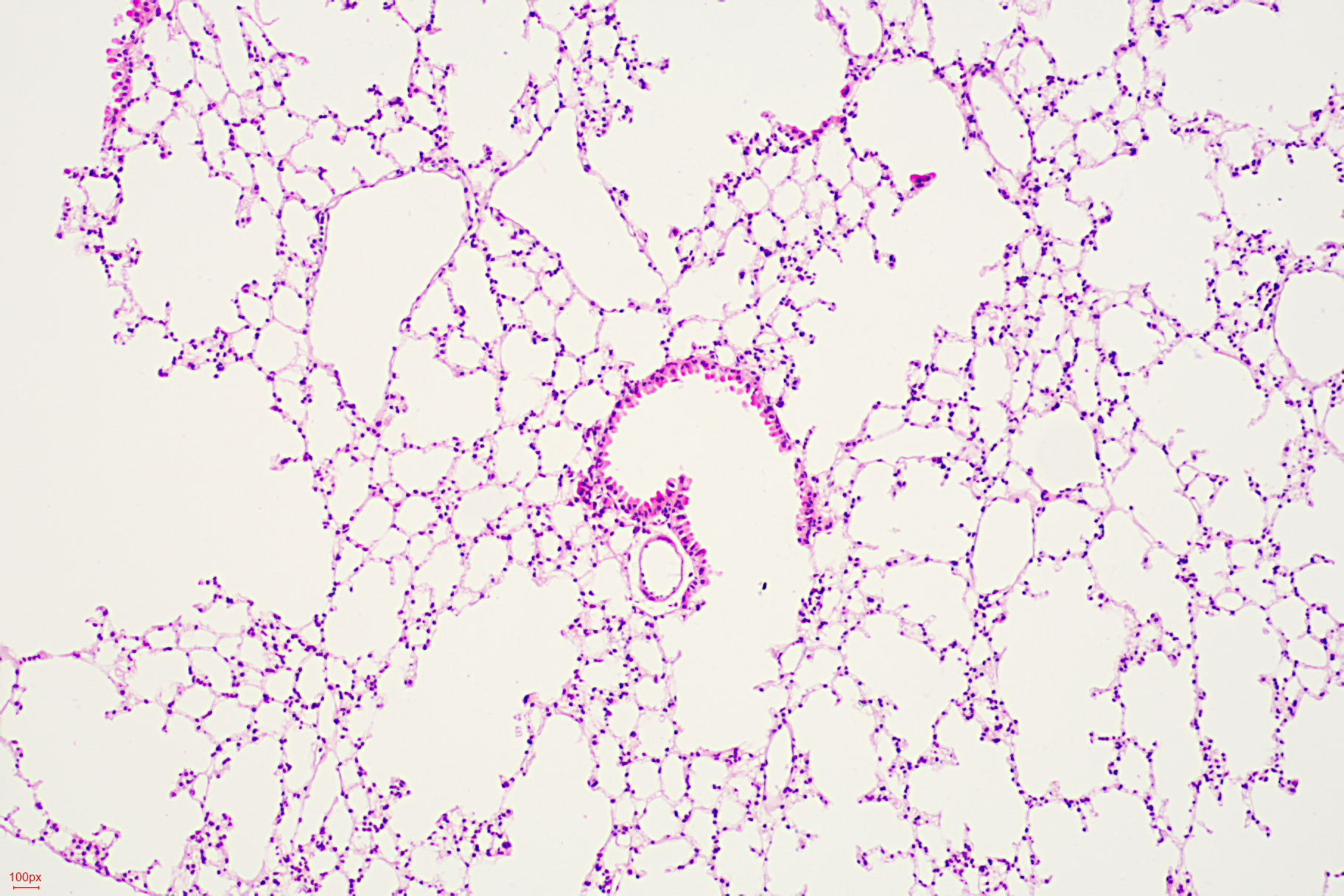

Supplement: Supplementary file 8 [file DataSheet_8.zip › Figure 8 raw datas/B. HE/LV2-NC.tif]

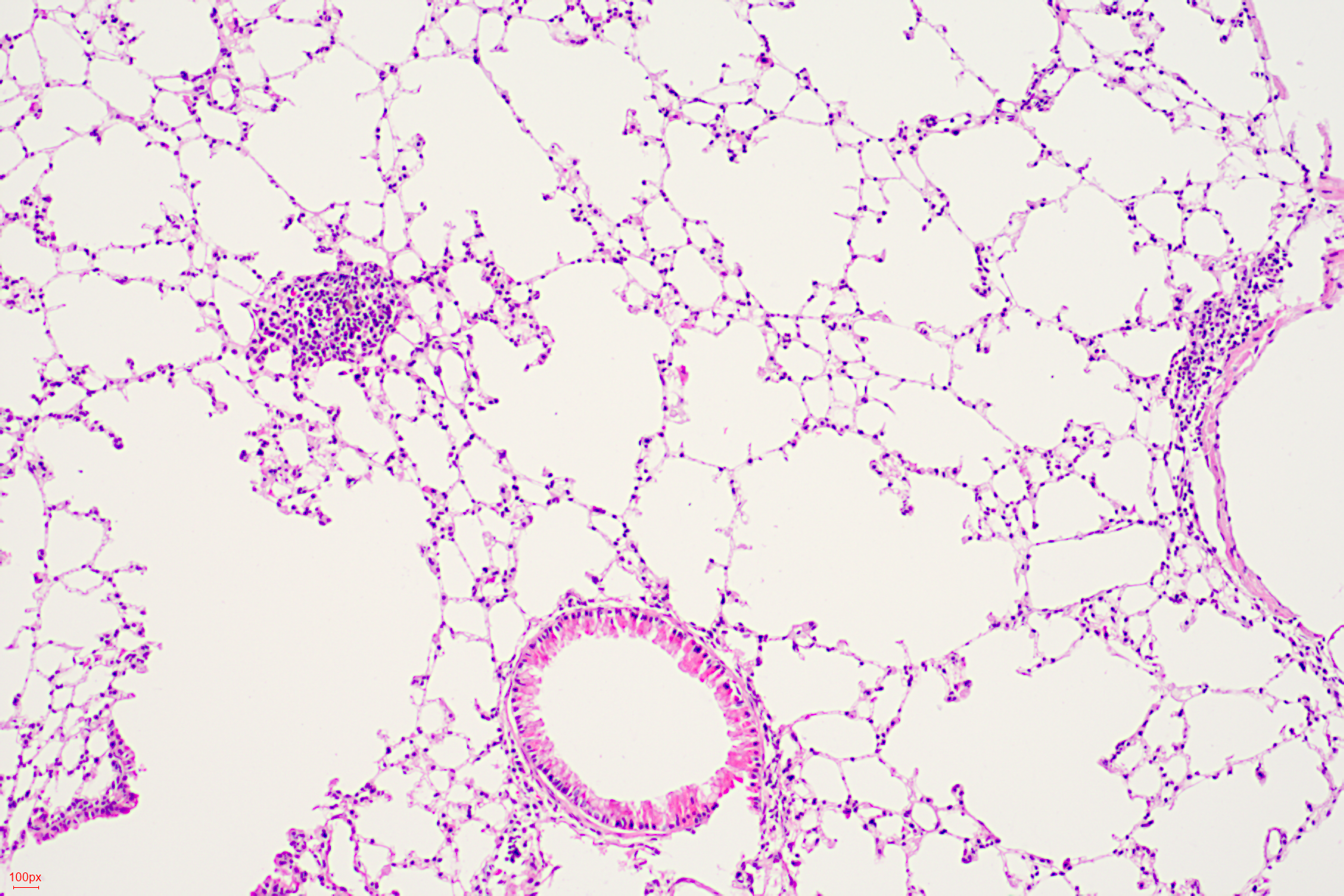

Supplement: Supplementary file 8 [file DataSheet_8.zip › Figure 8 raw datas/B. HE/OVA+SDC-1-shRNA.tif]

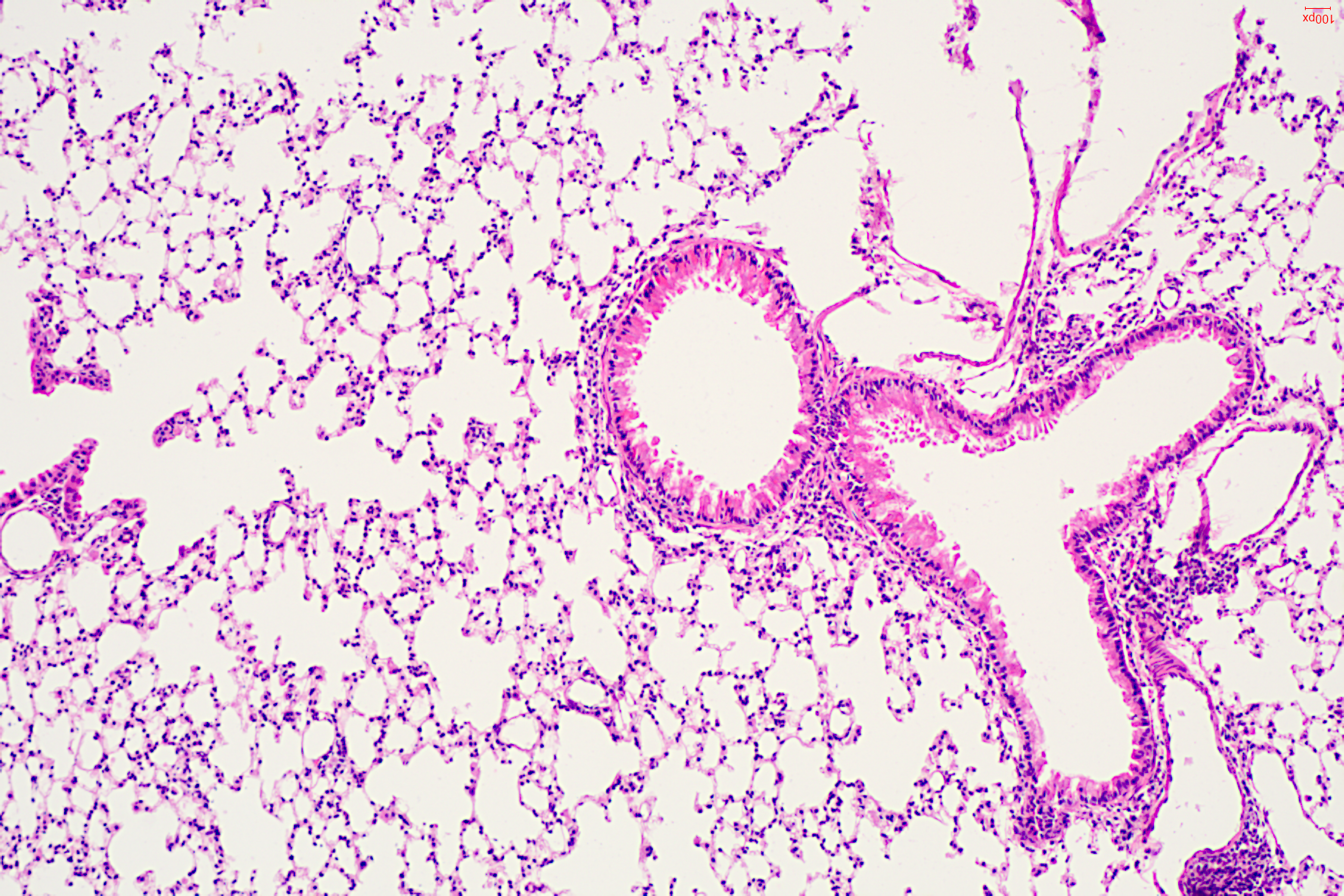

Supplement: Supplementary file 8 [file DataSheet_8.zip › Figure 8 raw datas/B. HE/OVA.tif]

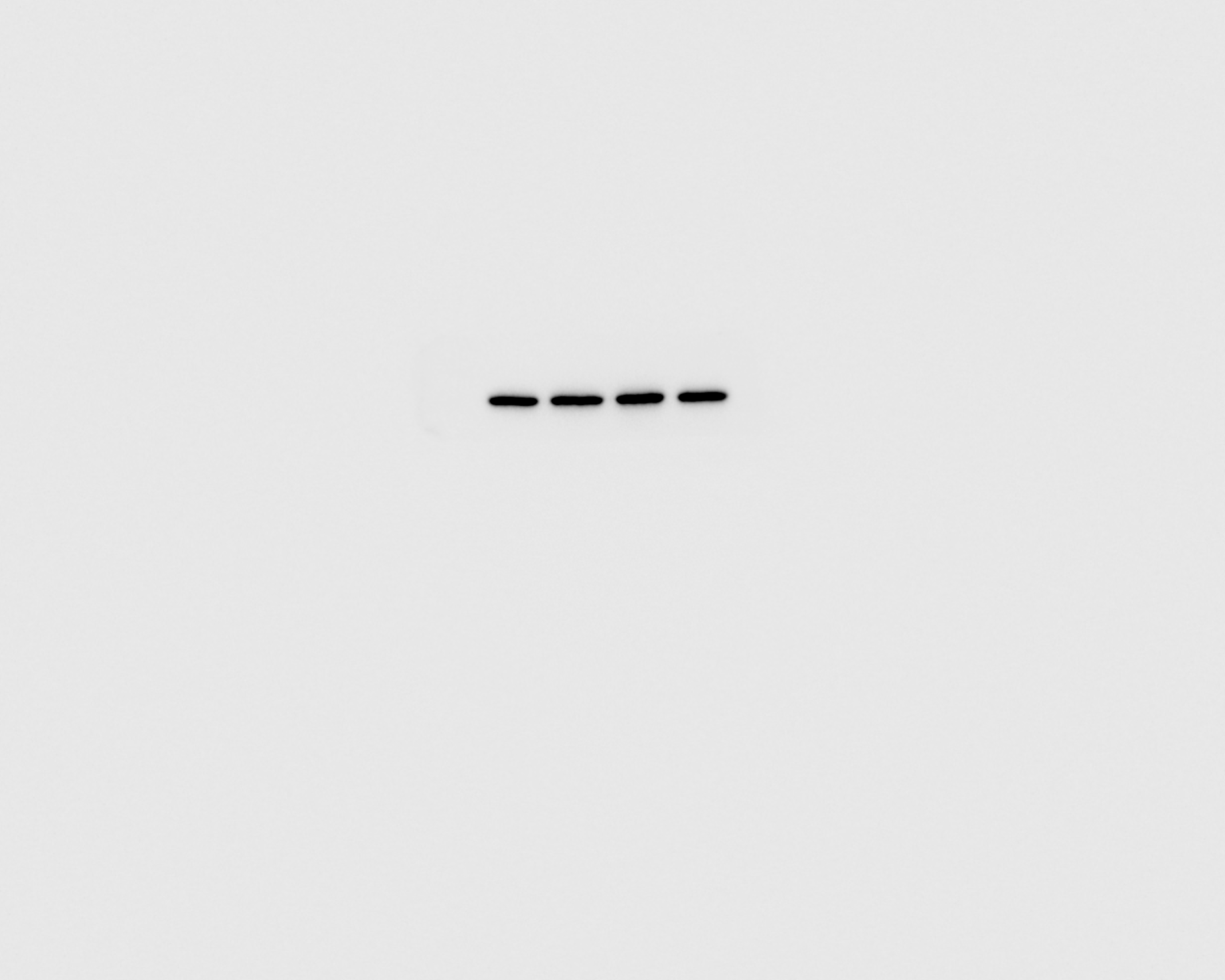

Supplement: Supplementary file 9 [file DataSheet_9.zip › Figure 9 raw datas/E. SDC-1 GAPDH/1.gapdh.tif]

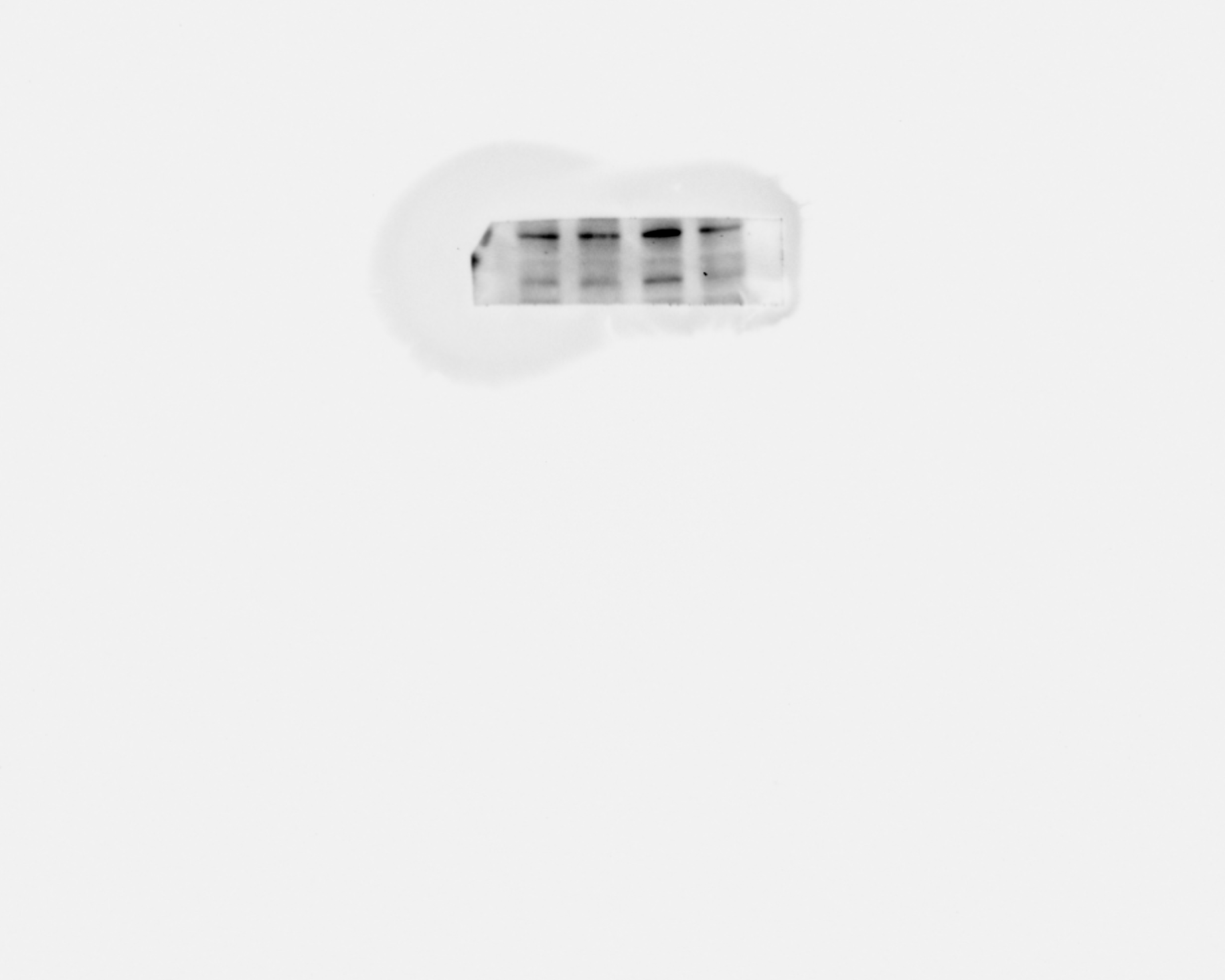

Supplement: Supplementary file 9 [file DataSheet_9.zip › Figure 9 raw datas/E. SDC-1 GAPDH/1.sdc.tif]

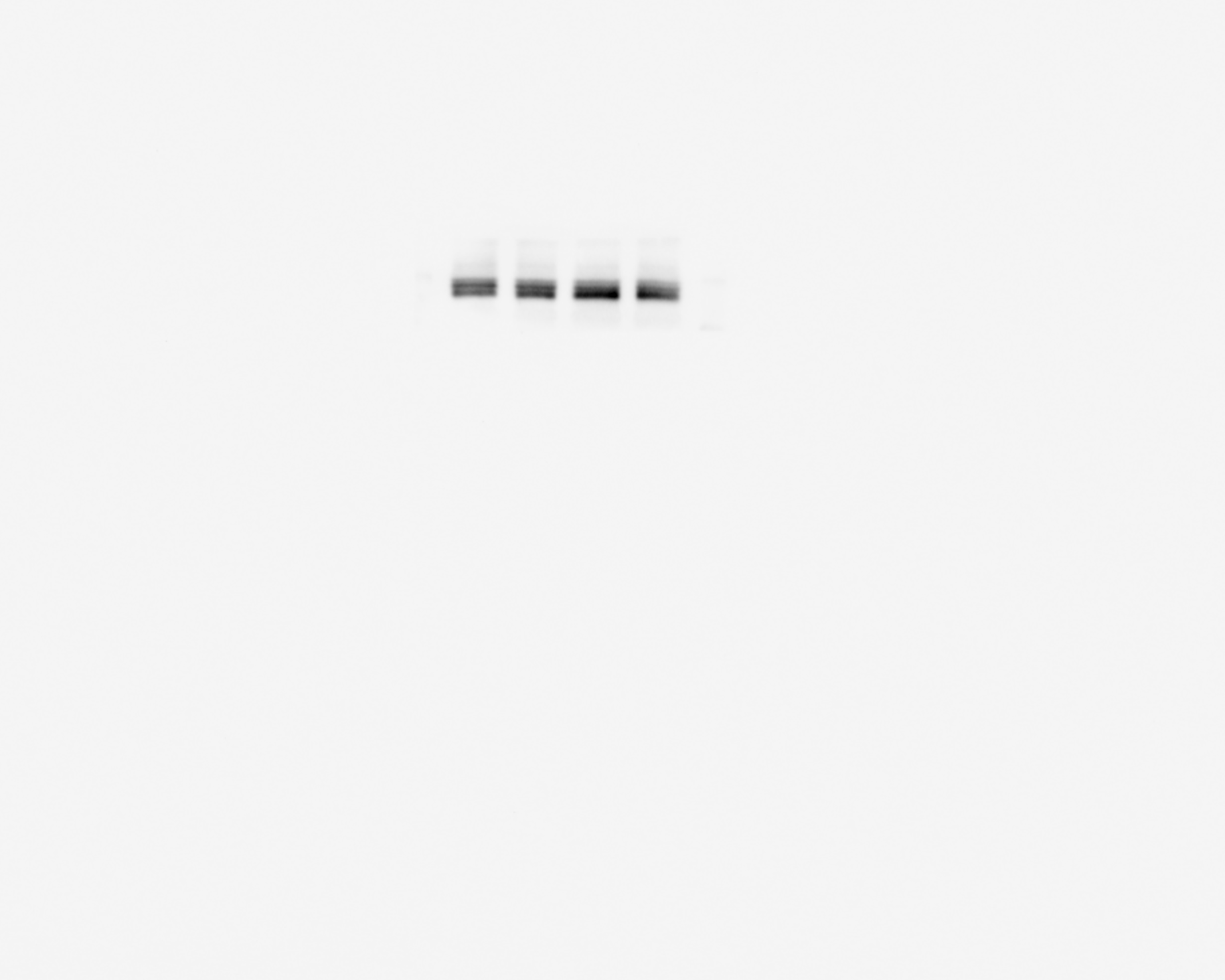

Supplement: Supplementary file 9 [file DataSheet_9.zip › Figure 9 raw datas/F. p-smad3 smad3/2.p3.tif]

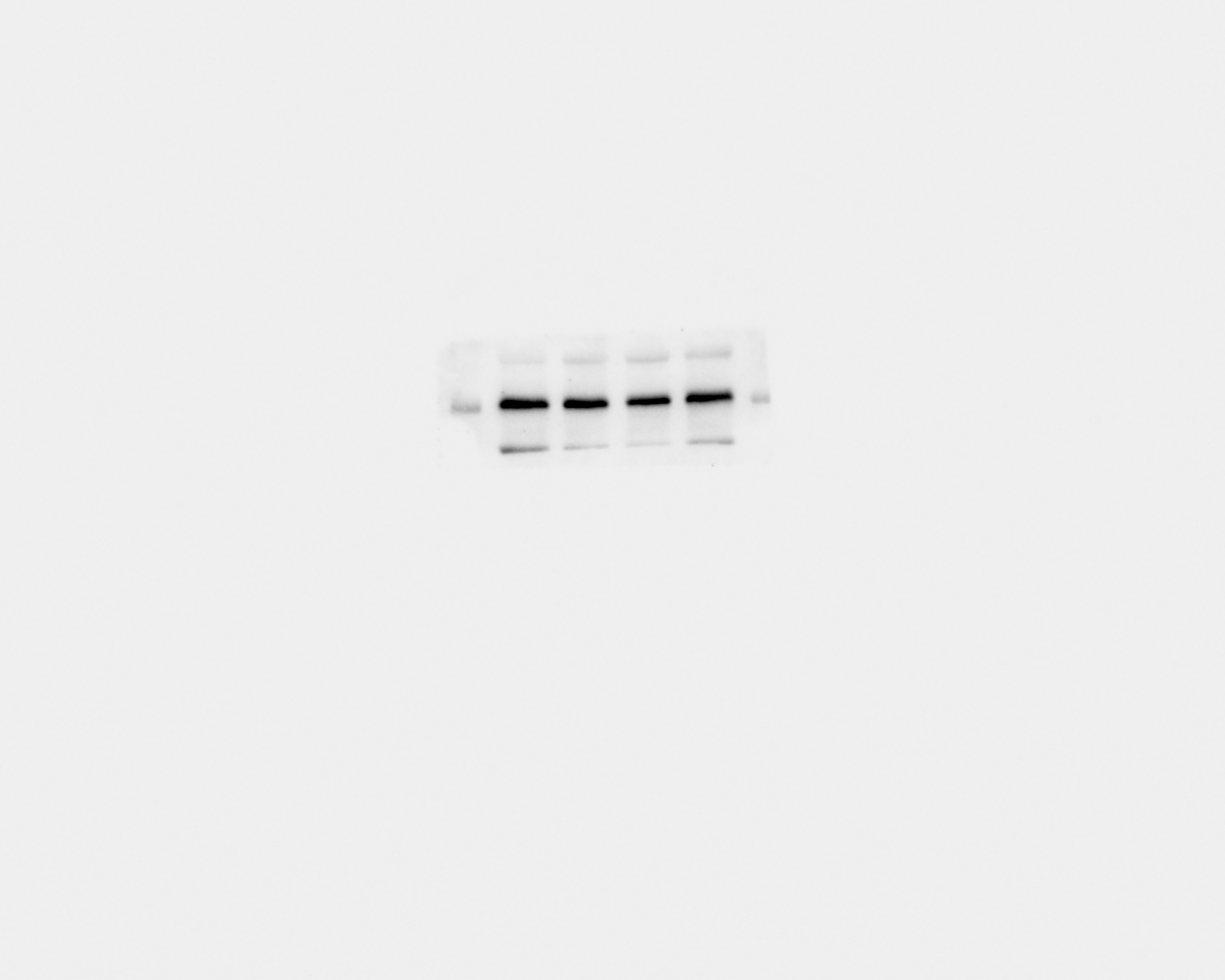

Supplement: Supplementary file 9 [file DataSheet_9.zip › Figure 9 raw datas/F. p-smad3 smad3/2.s3.tif]

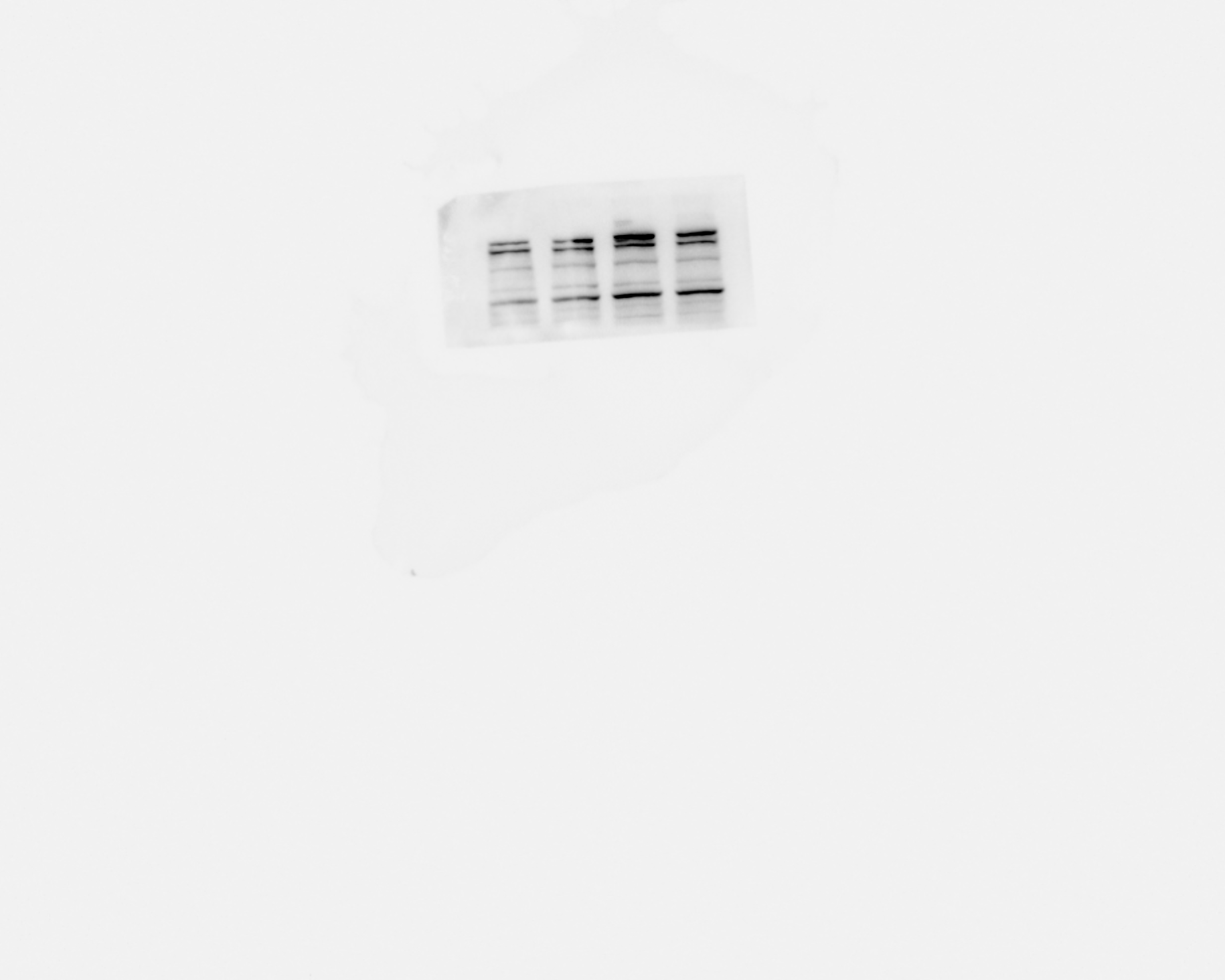

Supplement: Supplementary file 9 [file DataSheet_9.zip › Figure 9 raw datas/G. collagen I GAPDH/3.coll.tif]

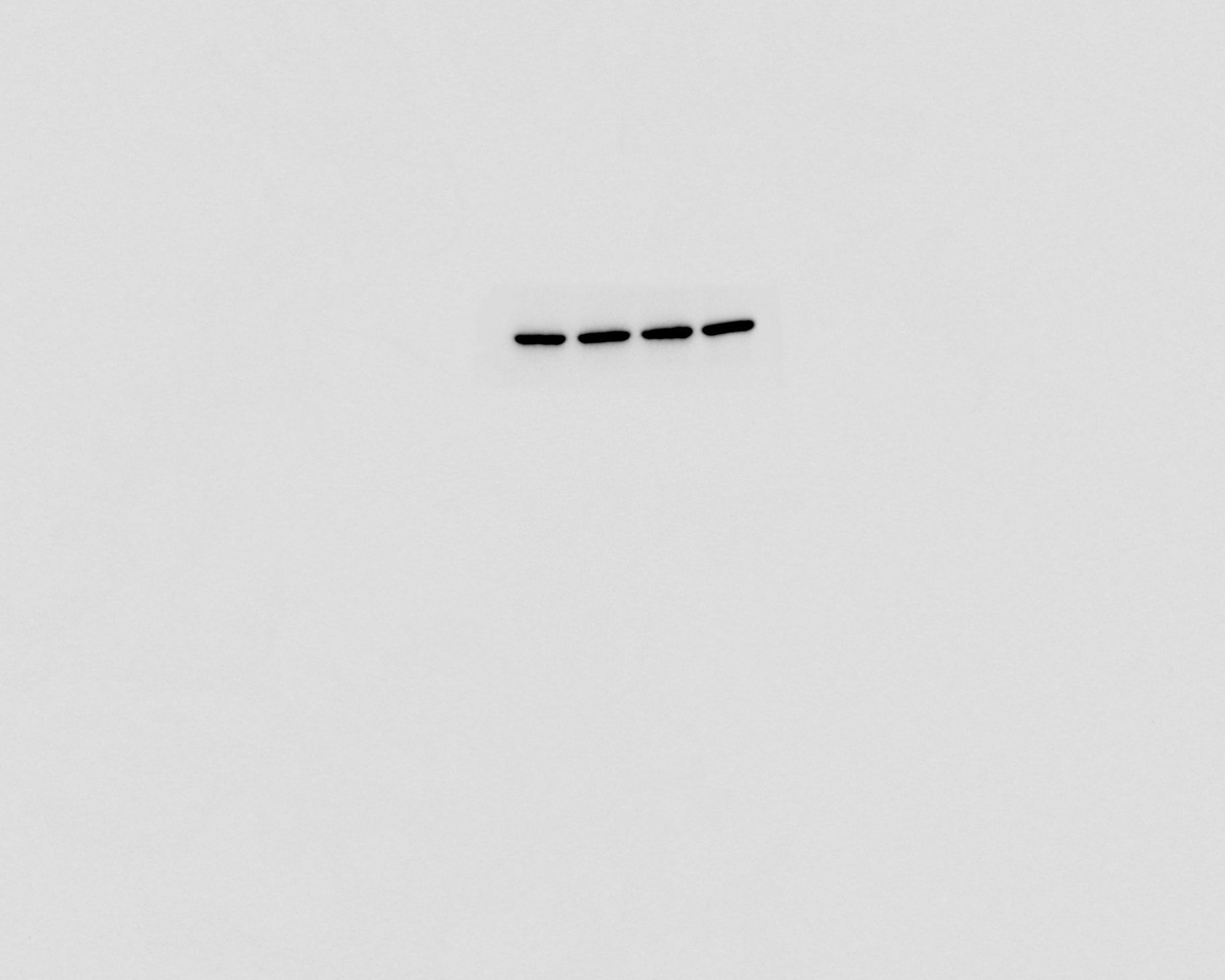

Supplement: Supplementary file 9 [file DataSheet_9.zip › Figure 9 raw datas/G. collagen I GAPDH/3.gapdh.tif]
