# Supplementary figures and images for: Syndecan-1 Amplifies Ovalbumin-Induced Airway Remodeling by Strengthening TGFβ1/Smad3 Action (part 2 of 2)
Source: Front Immunol. 2021 Oct 4;12:744477. doi: 10.3389/fimmu.2021.744477 (PMC8521046; doi:10.3389/fimmu.2021.744477)

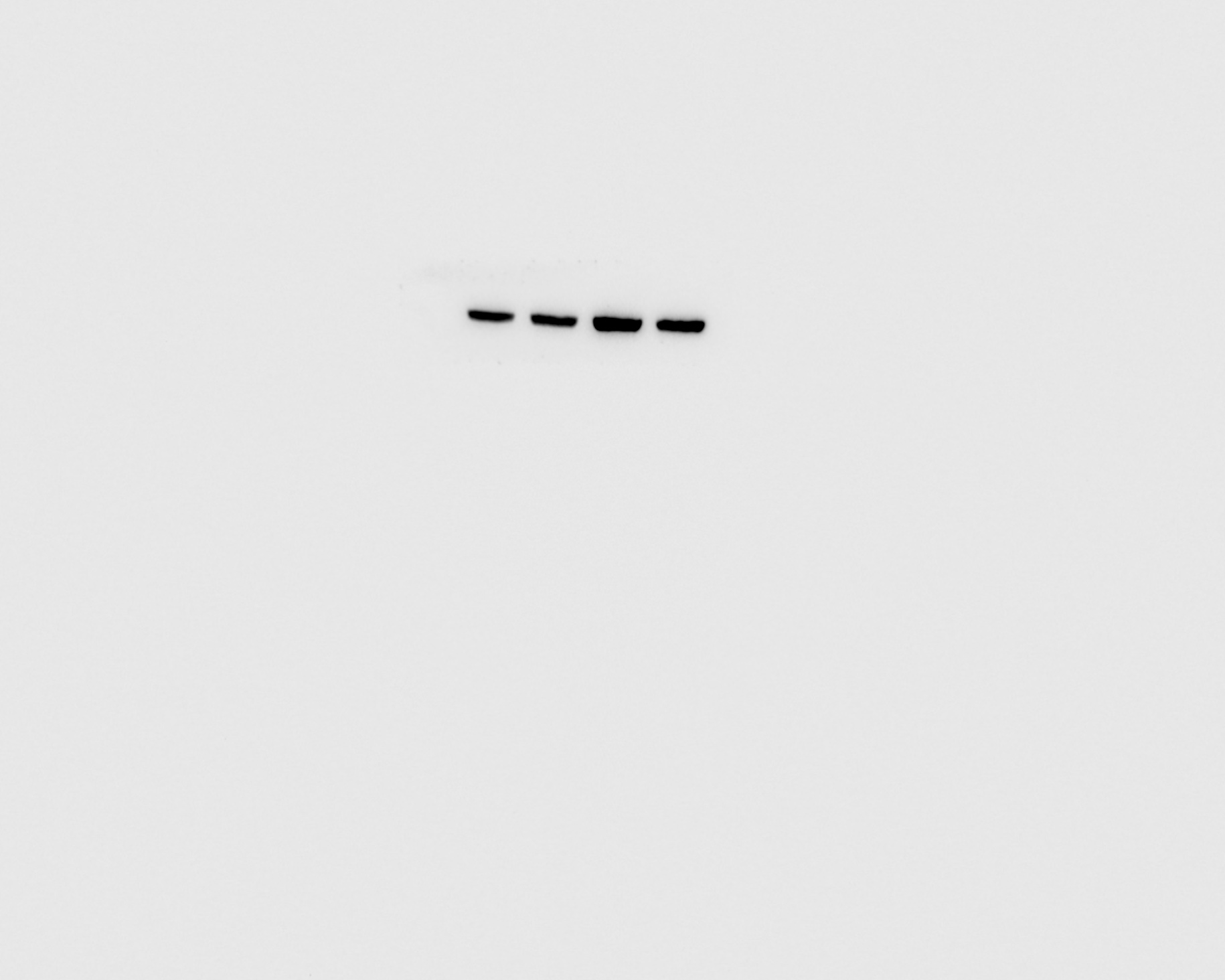

Supplement: Supplementary file 9 [file DataSheet_9.zip › Figure 9 raw datas/H. SMA GAPDH/4.SMA.tif]

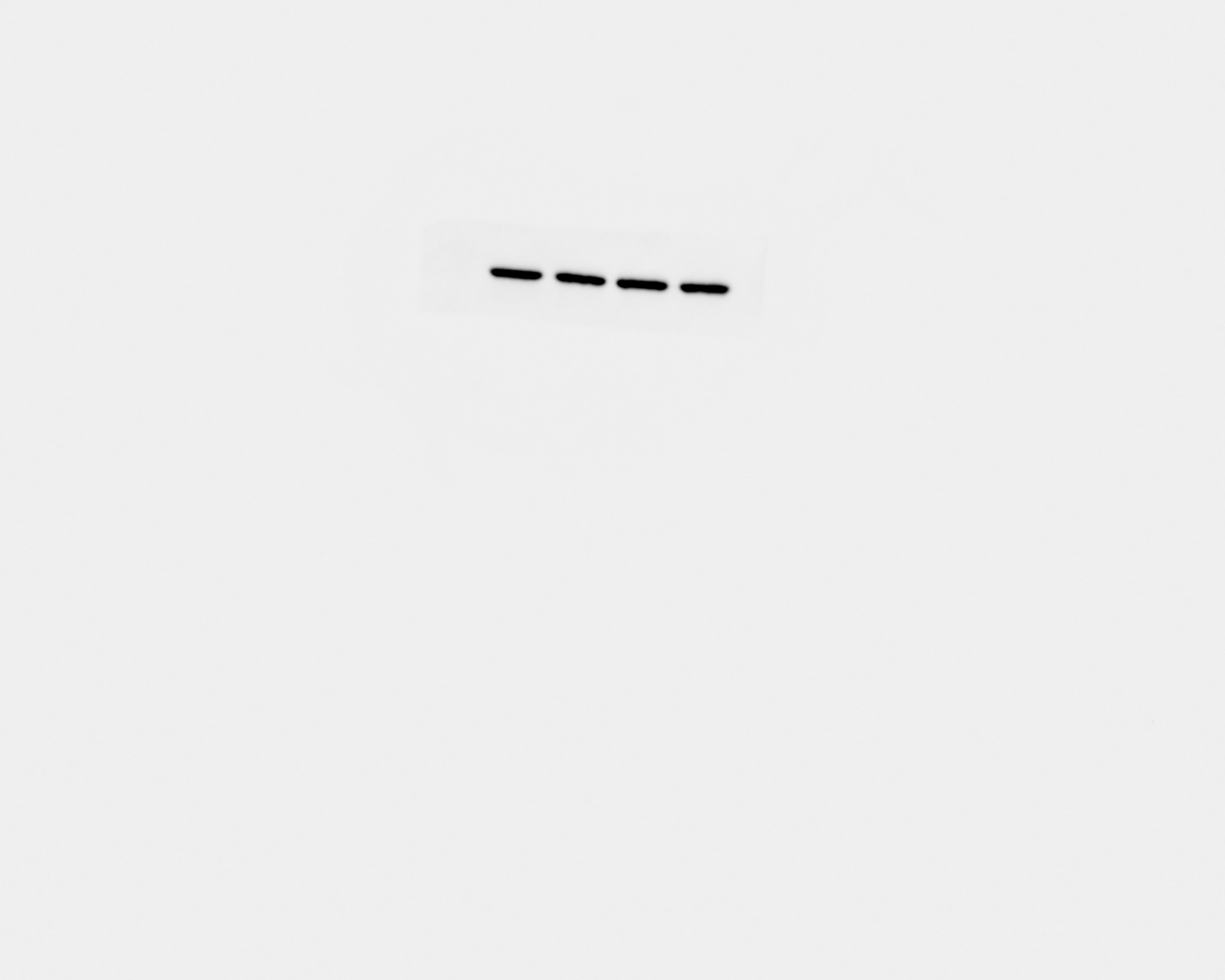

Supplement: Supplementary file 9 [file DataSheet_9.zip › Figure 9 raw datas/H. SMA GAPDH/4.gapdh.tif]

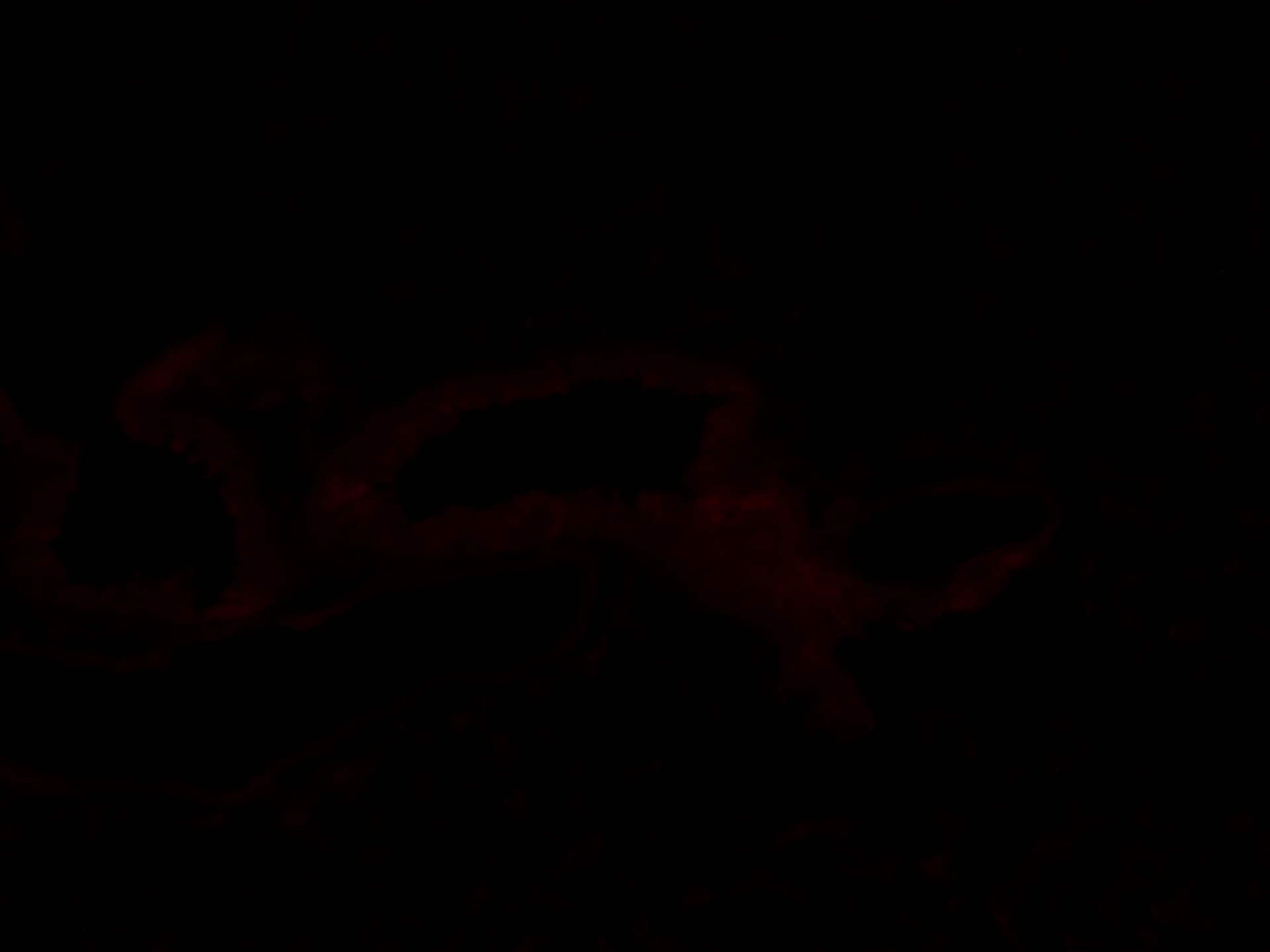

Supplement: Supplementary file 10 [file DataSheet_10.zip › Figure 10 raw datas/A. p-smad3/Control 1.tif]

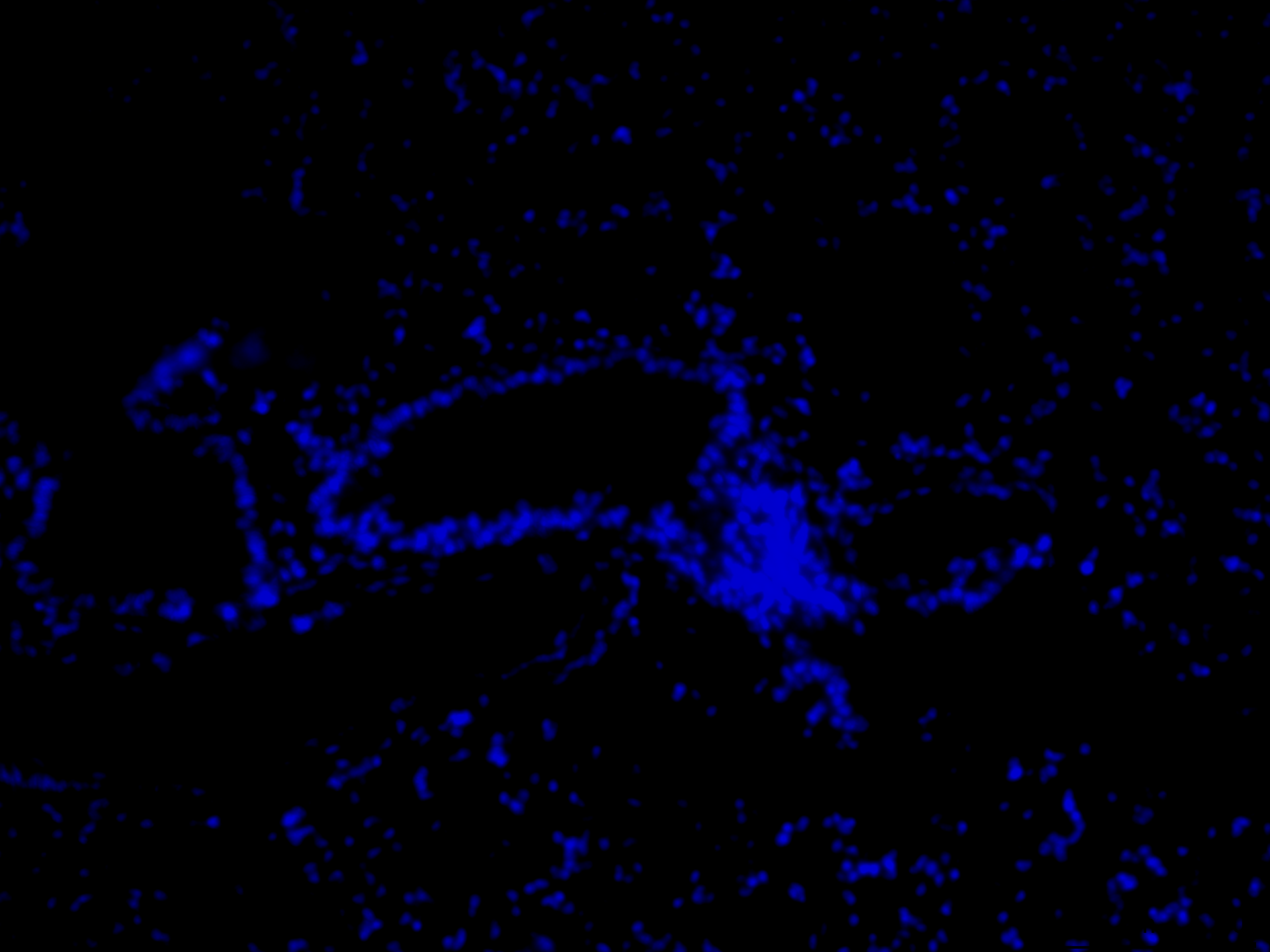

Supplement: Supplementary file 10 [file DataSheet_10.zip › Figure 10 raw datas/A. p-smad3/Control 2.tif]

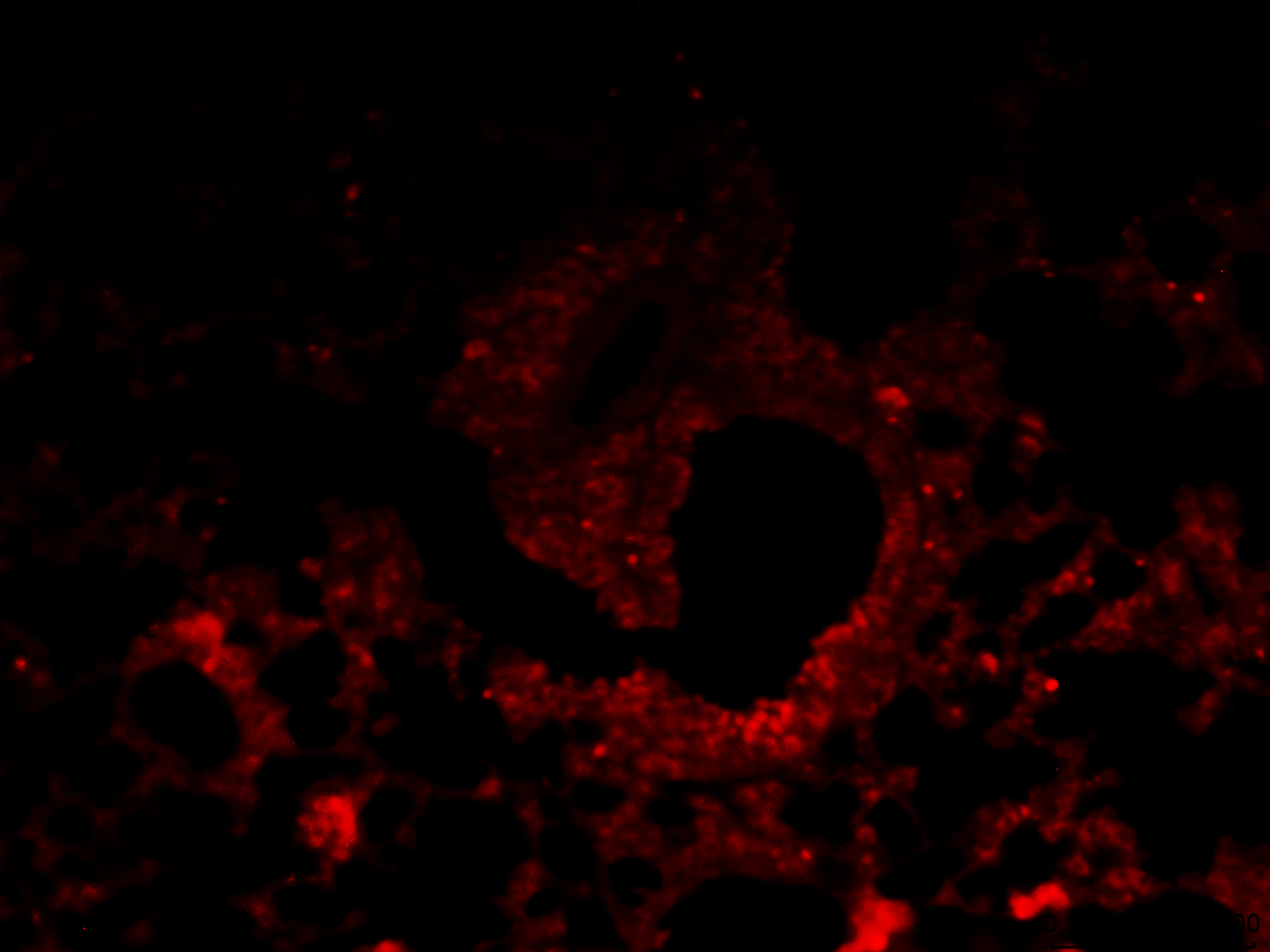

Supplement: Supplementary file 10 [file DataSheet_10.zip › Figure 10 raw datas/A. p-smad3/OVA 1.tif]

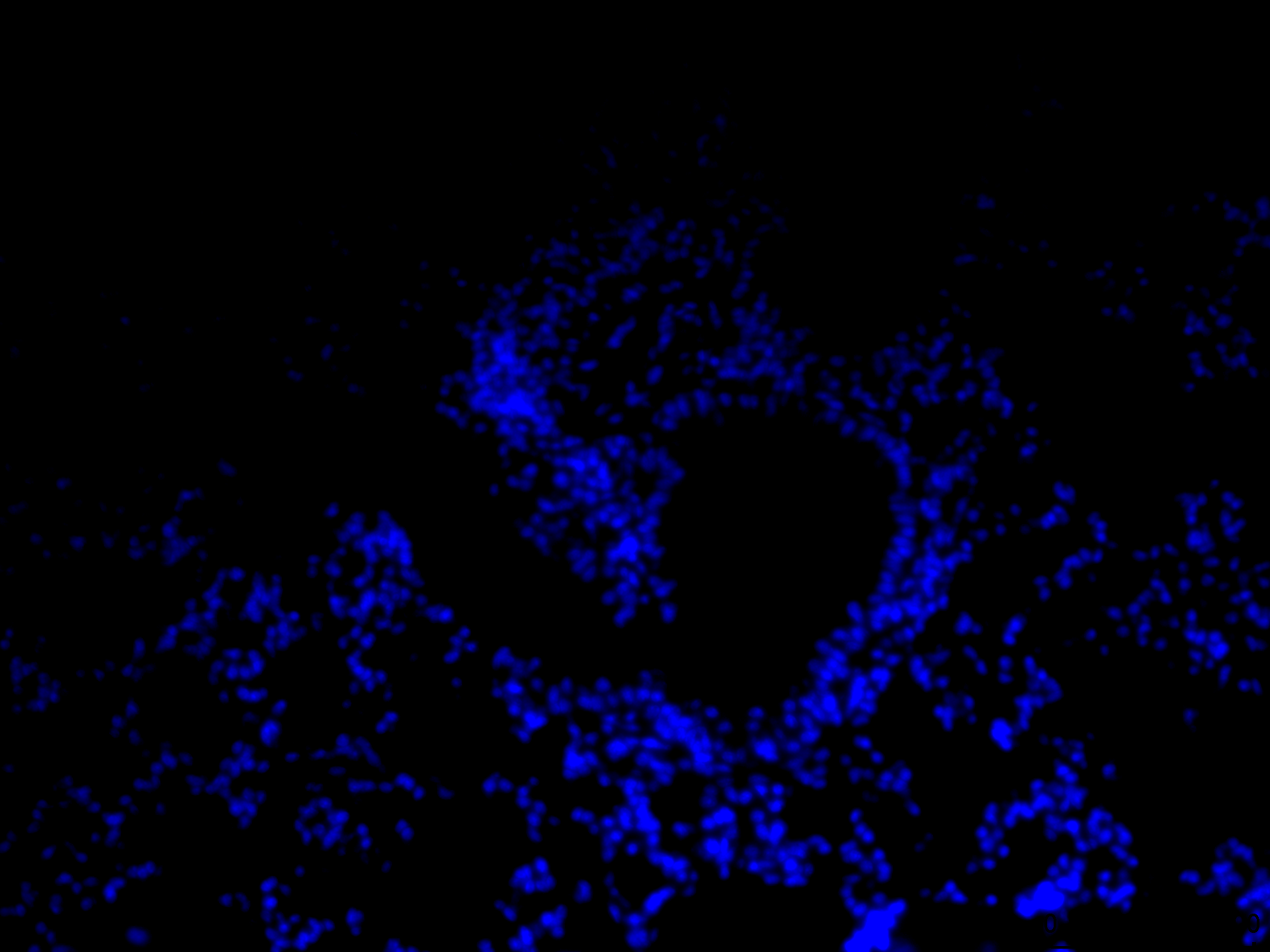

Supplement: Supplementary file 10 [file DataSheet_10.zip › Figure 10 raw datas/A. p-smad3/OVA 2.tif]

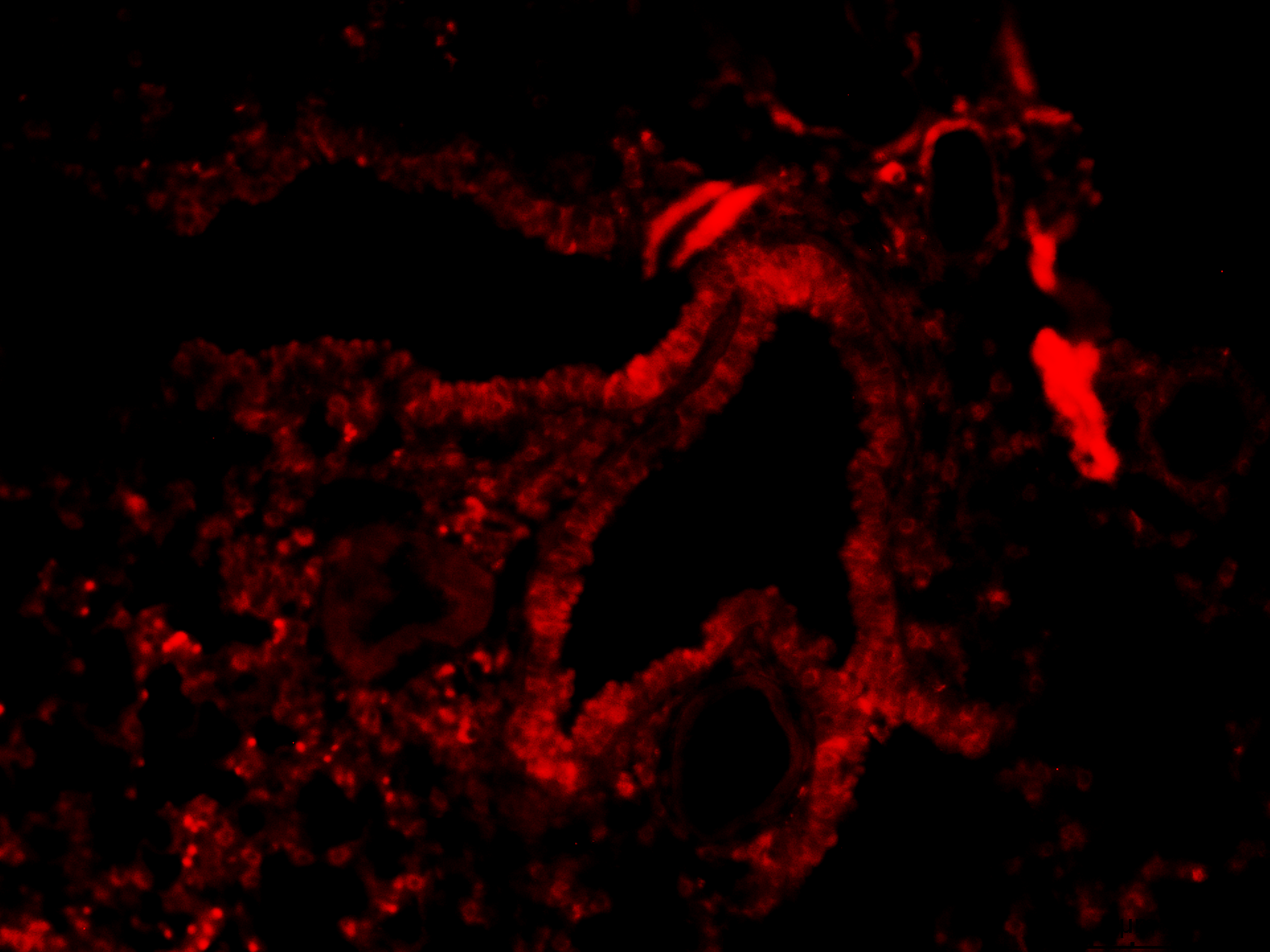

Supplement: Supplementary file 10 [file DataSheet_10.zip › Figure 10 raw datas/A. p-smad3/OVA+recombinant SDC1-1.tif]

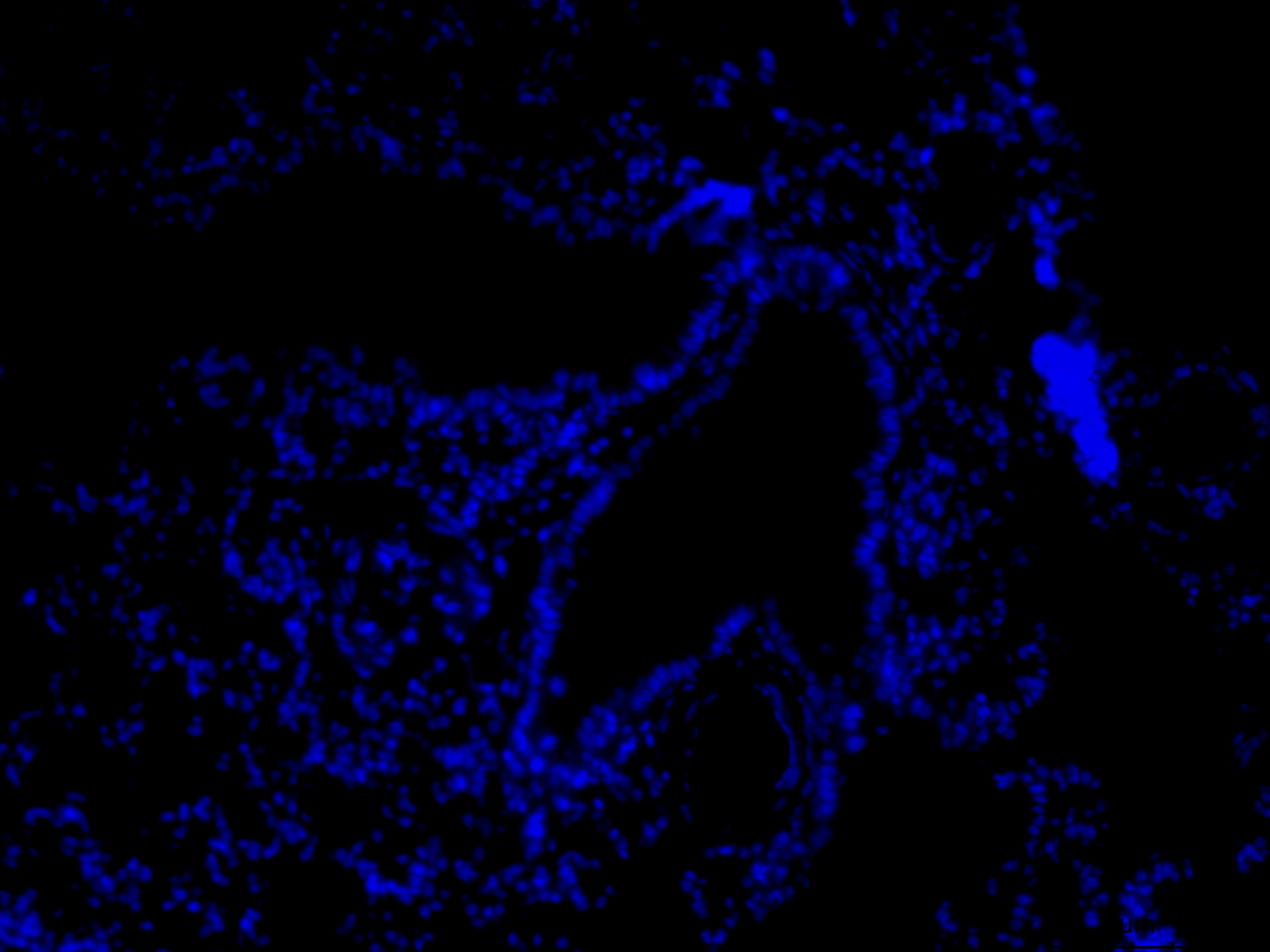

Supplement: Supplementary file 10 [file DataSheet_10.zip › Figure 10 raw datas/A. p-smad3/OVA+recombinant SDC1-2.tif]

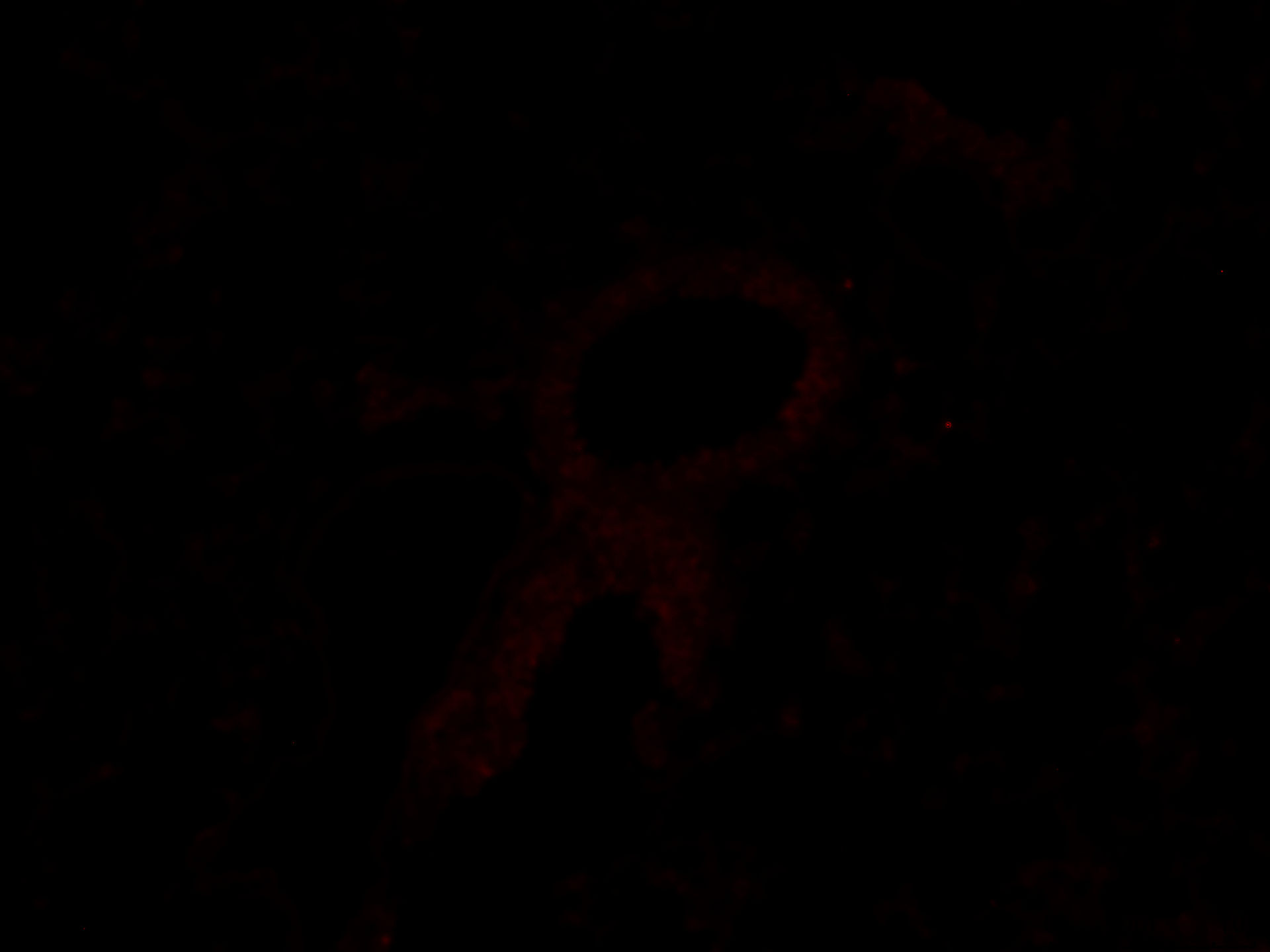

Supplement: Supplementary file 10 [file DataSheet_10.zip › Figure 10 raw datas/A. p-smad3/Recombinant SDC1-1.tif]

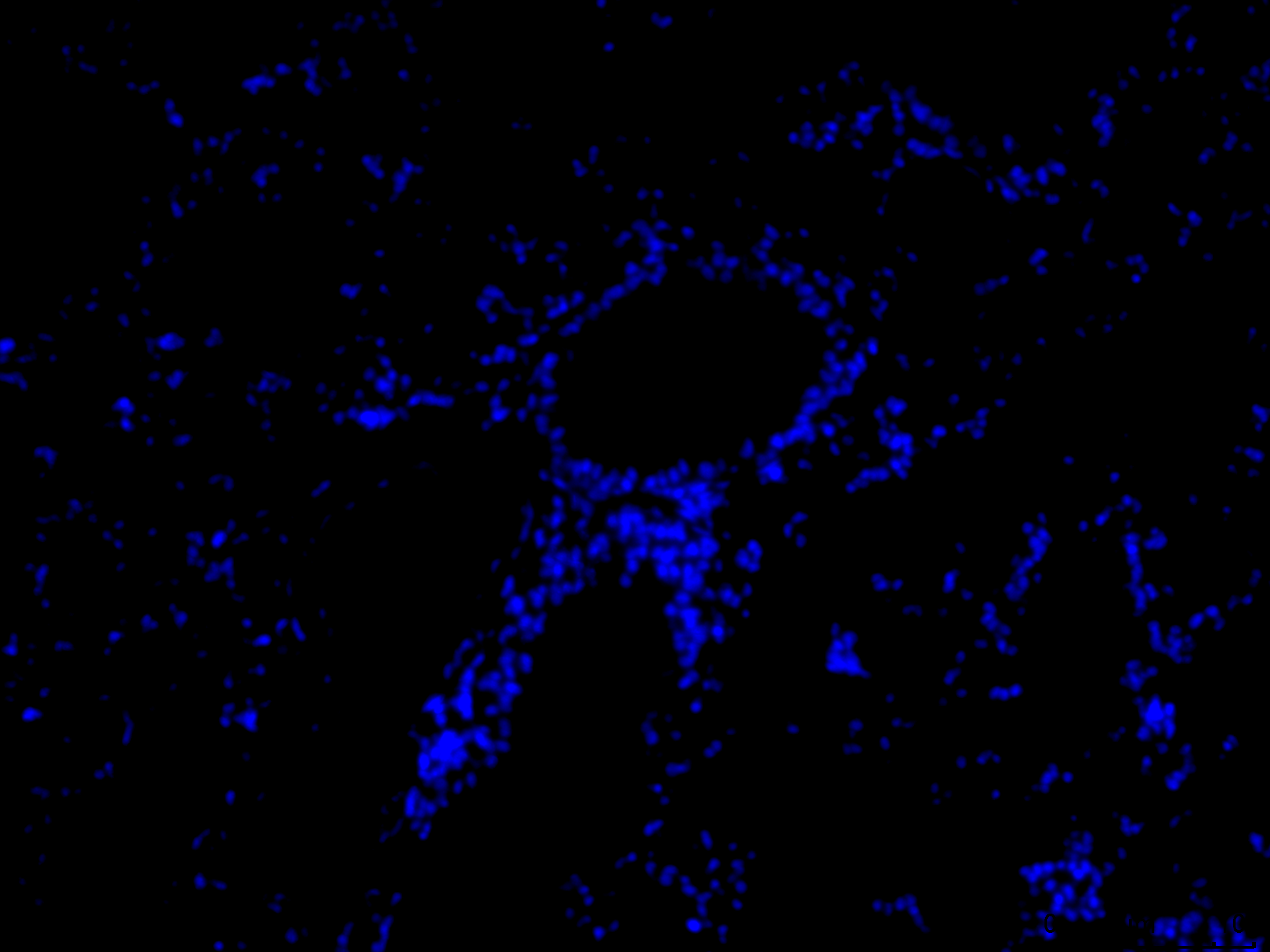

Supplement: Supplementary file 10 [file DataSheet_10.zip › Figure 10 raw datas/A. p-smad3/Recombinant SDC1-2.tif]

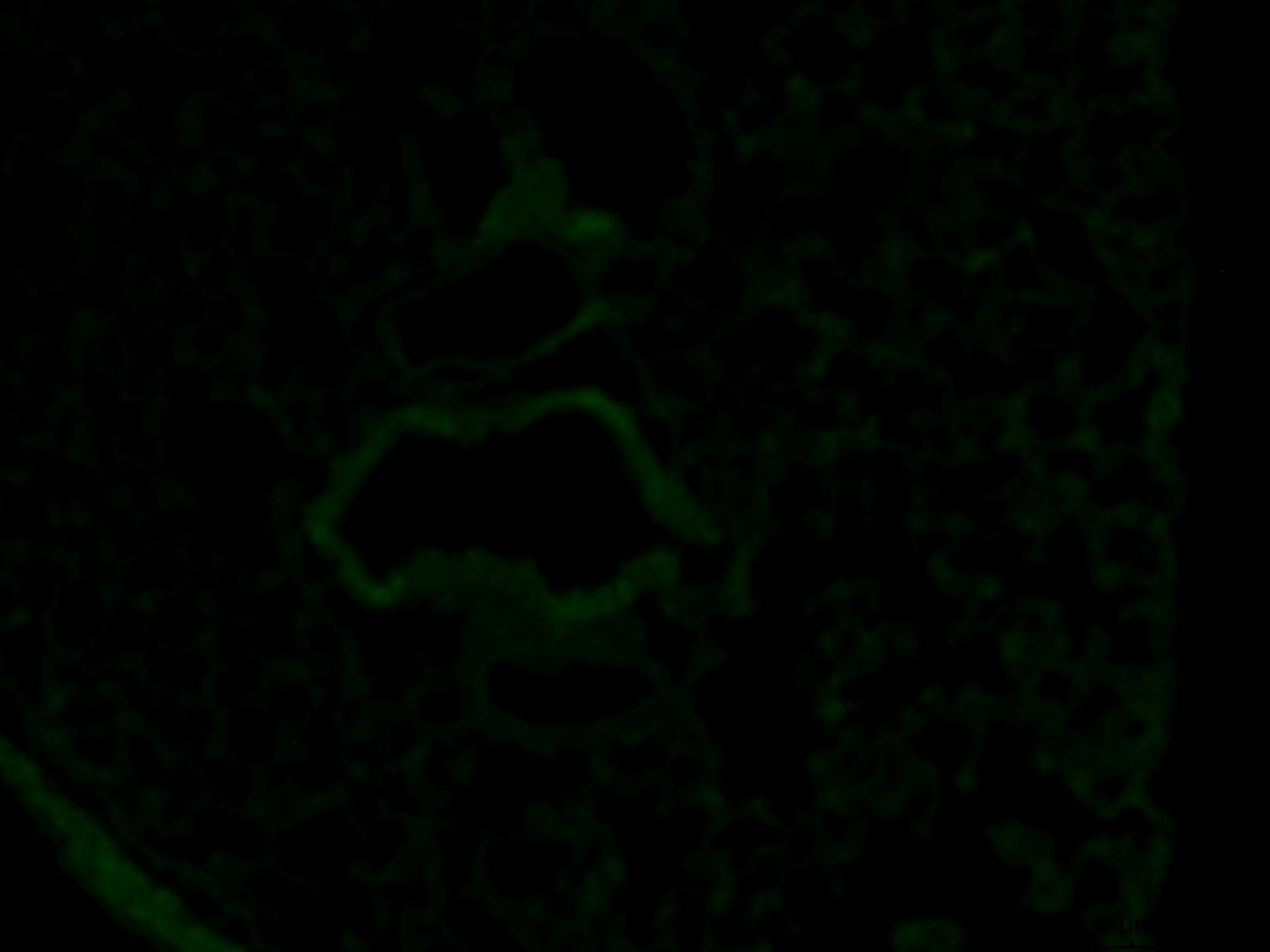

Supplement: Supplementary file 10 [file DataSheet_10.zip › Figure 10 raw datas/B. Collagen I/N11.tif]

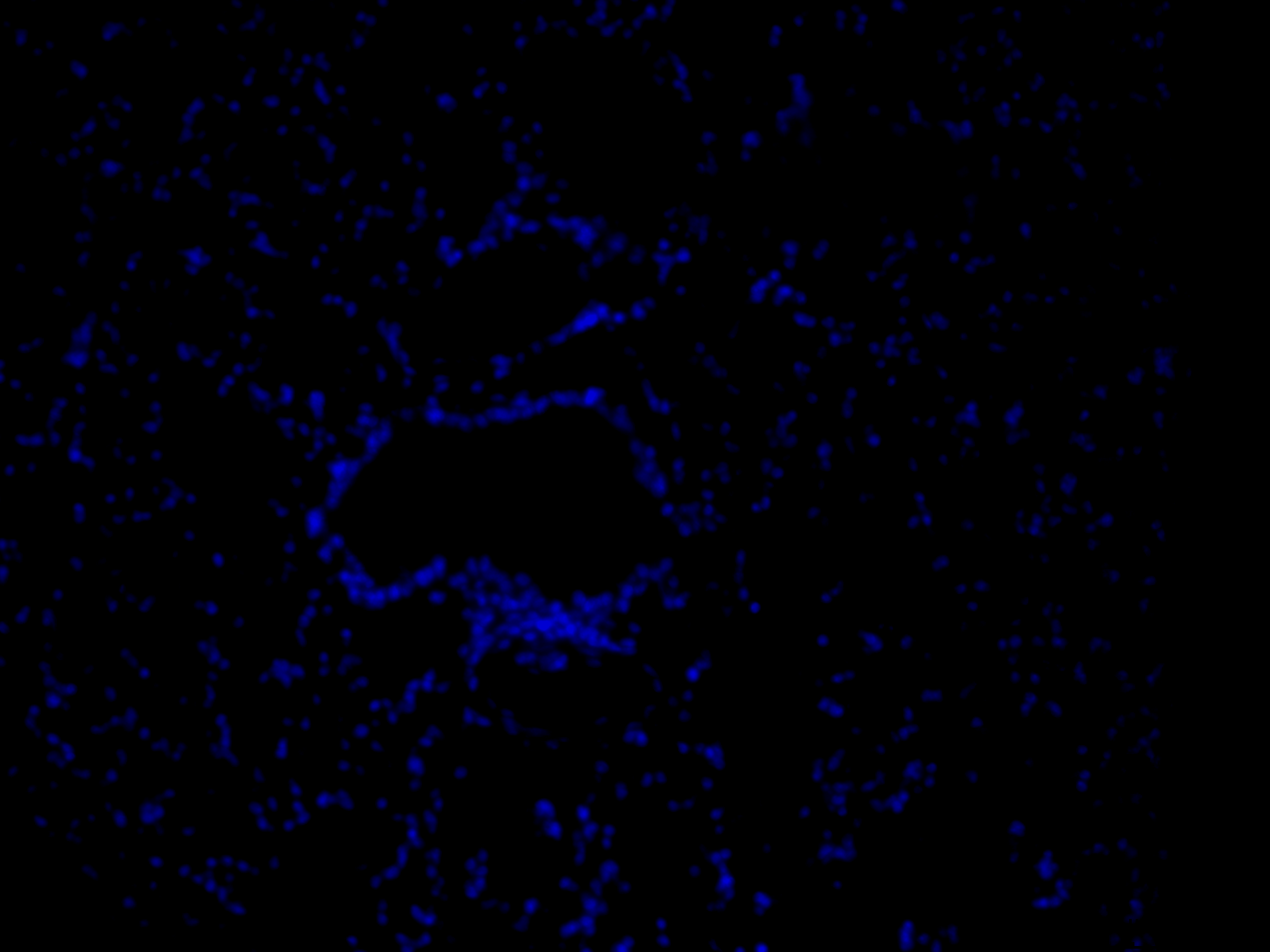

Supplement: Supplementary file 10 [file DataSheet_10.zip › Figure 10 raw datas/B. Collagen I/N12.tif]

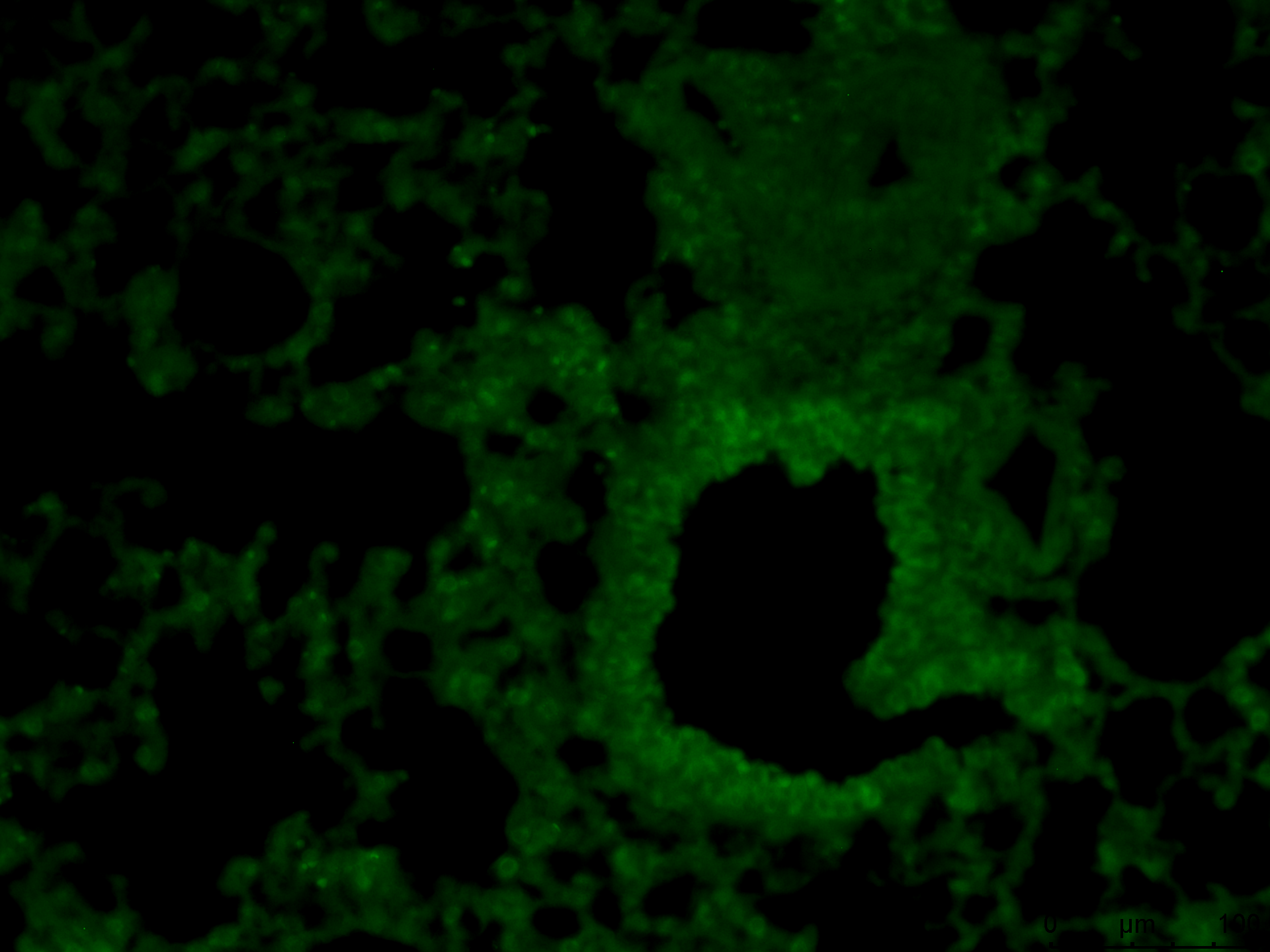

Supplement: Supplementary file 10 [file DataSheet_10.zip › Figure 10 raw datas/B. Collagen I/OVA-SC1-11.tif]

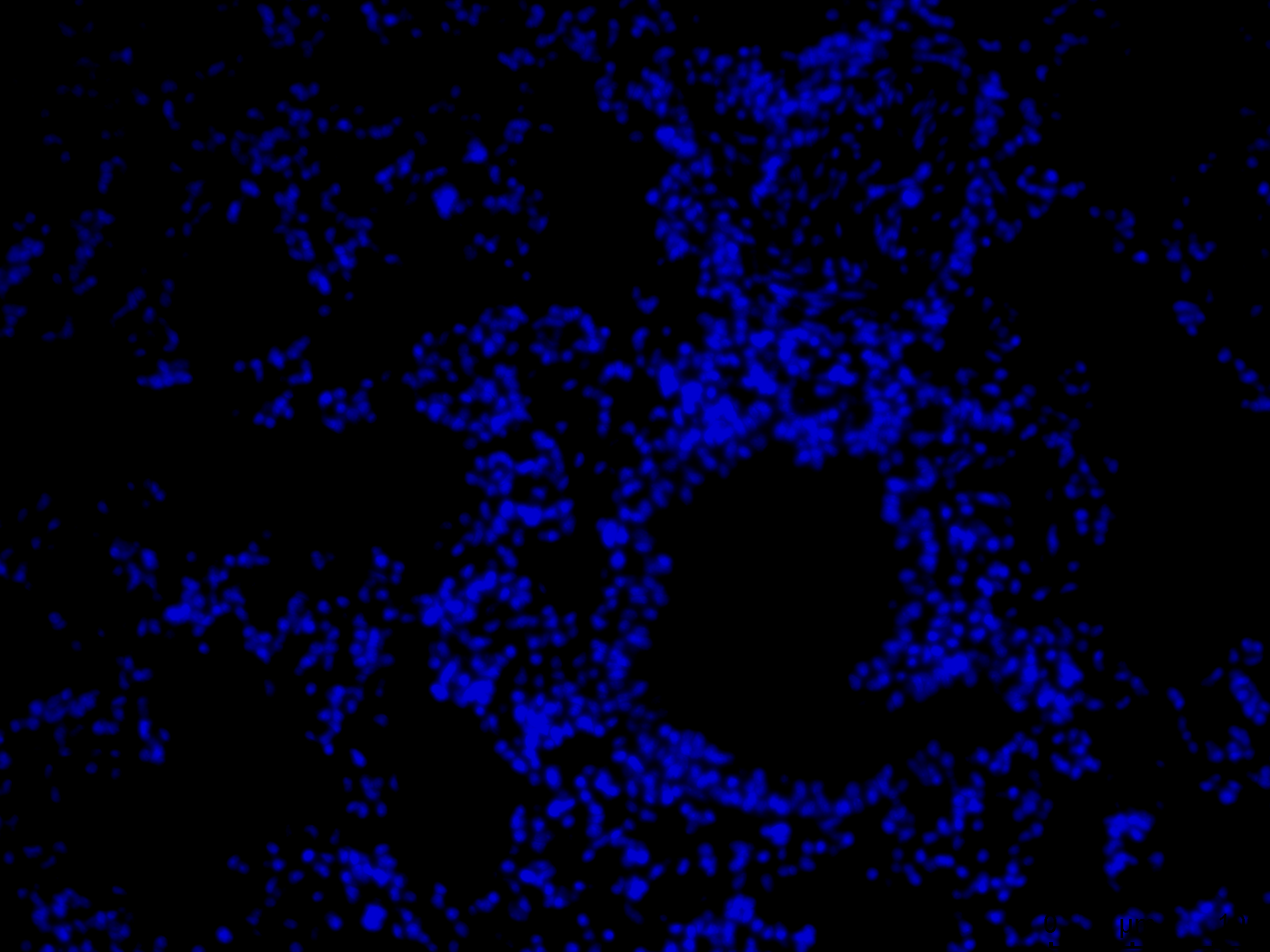

Supplement: Supplementary file 10 [file DataSheet_10.zip › Figure 10 raw datas/B. Collagen I/OVA-SC1-12.tif]

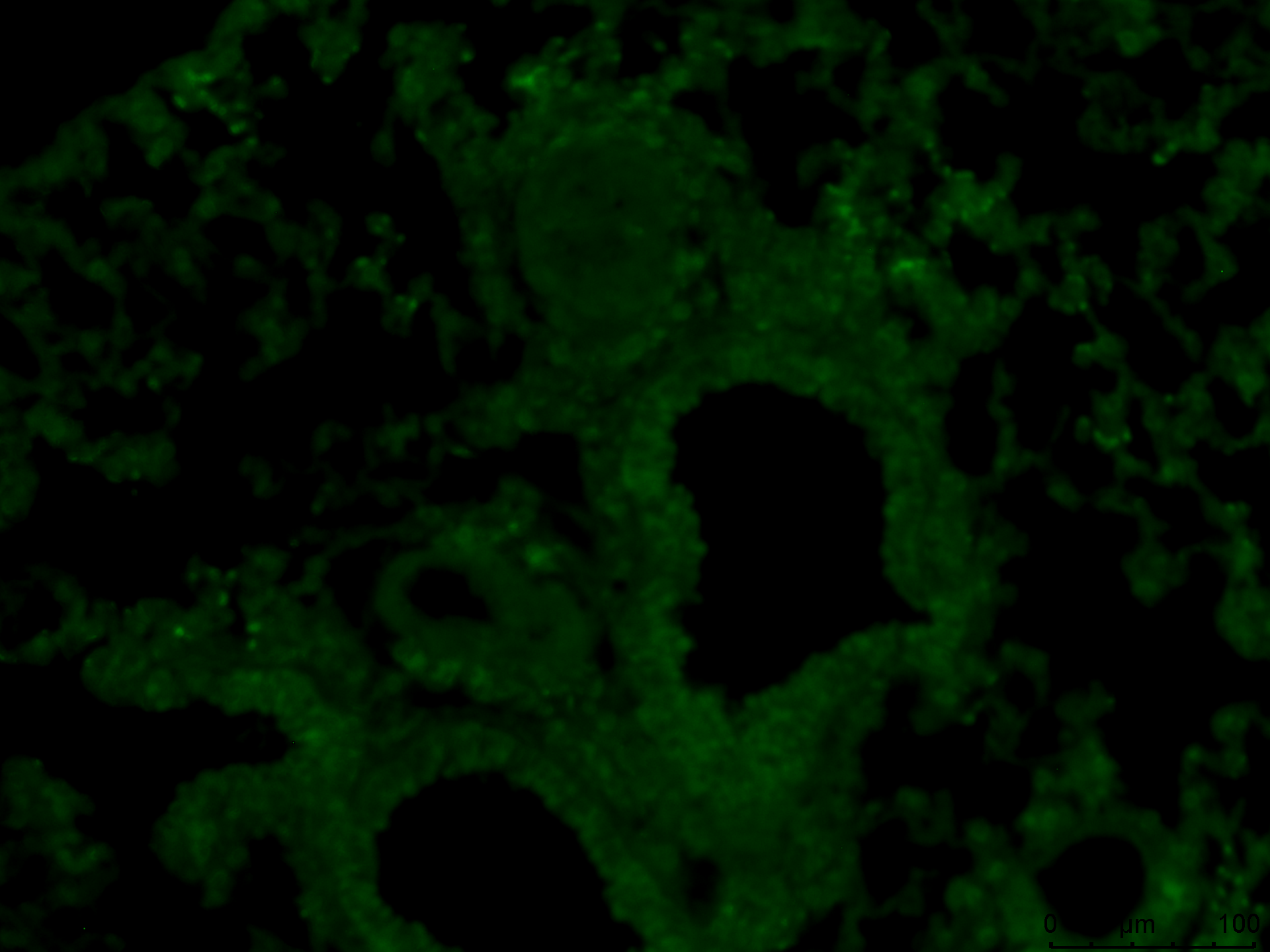

Supplement: Supplementary file 10 [file DataSheet_10.zip › Figure 10 raw datas/B. Collagen I/OVA1.tif]

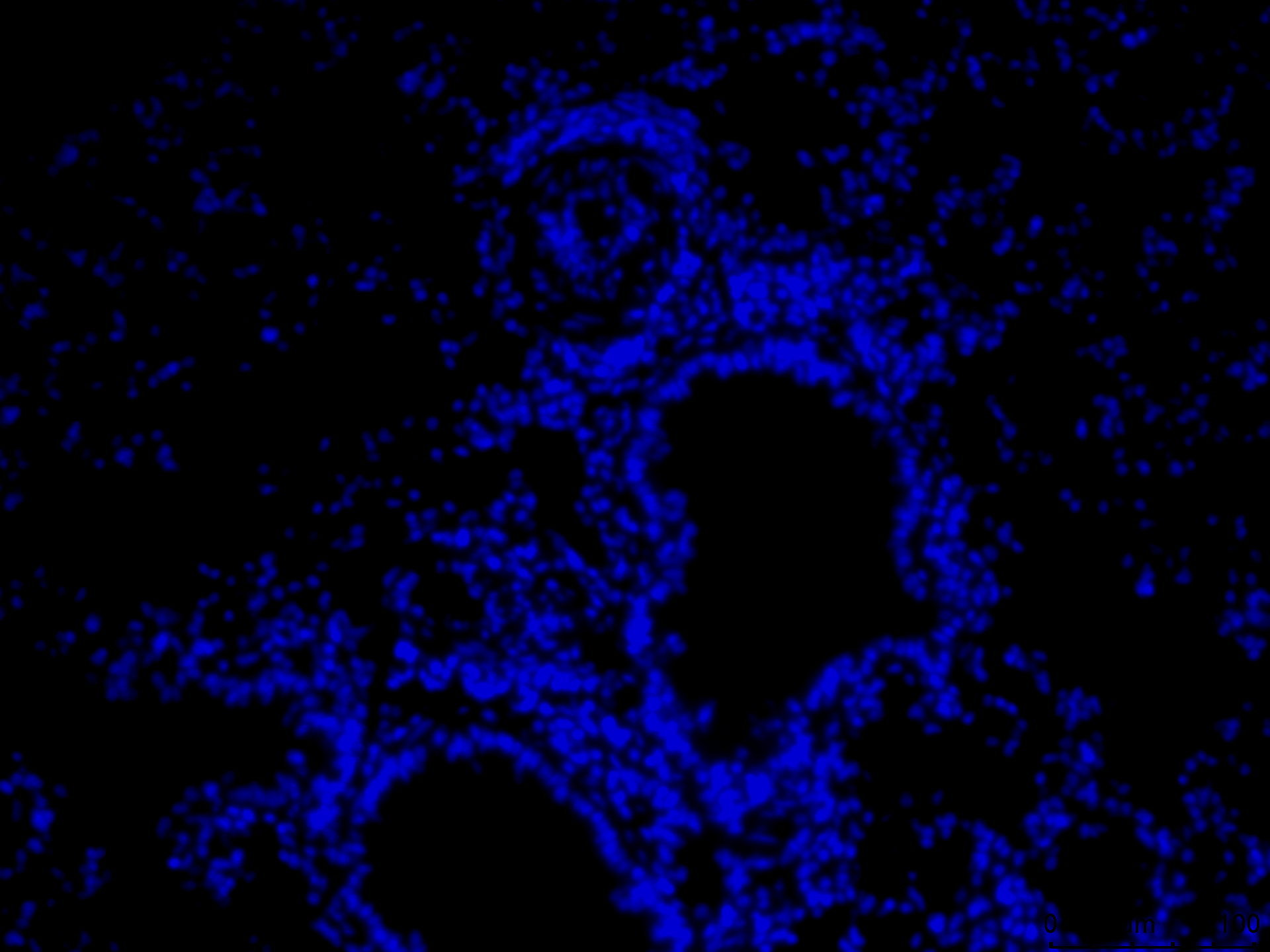

Supplement: Supplementary file 10 [file DataSheet_10.zip › Figure 10 raw datas/B. Collagen I/OVA2.tif]

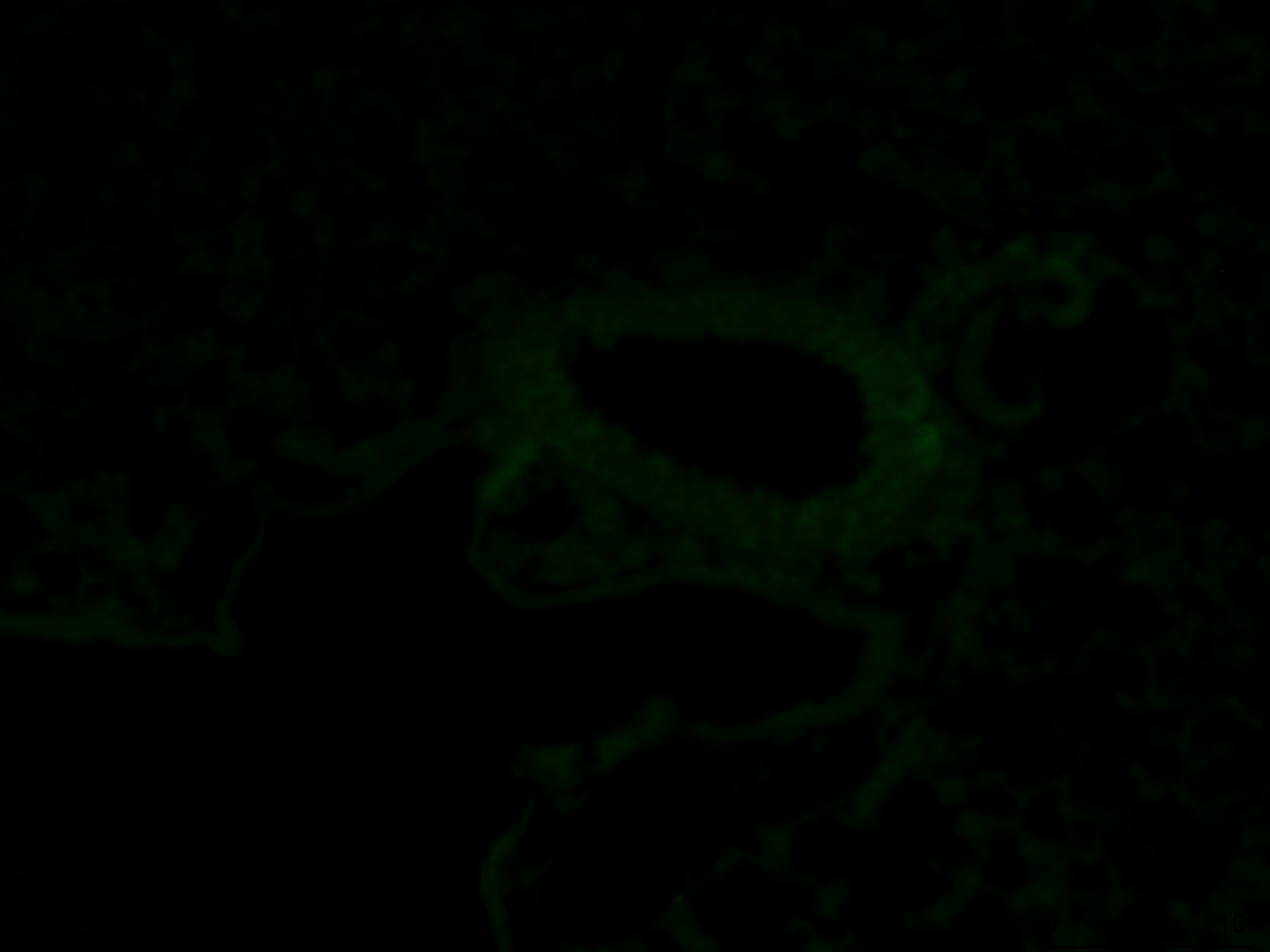

Supplement: Supplementary file 10 [file DataSheet_10.zip › Figure 10 raw datas/B. Collagen I/╢╘11.tif]

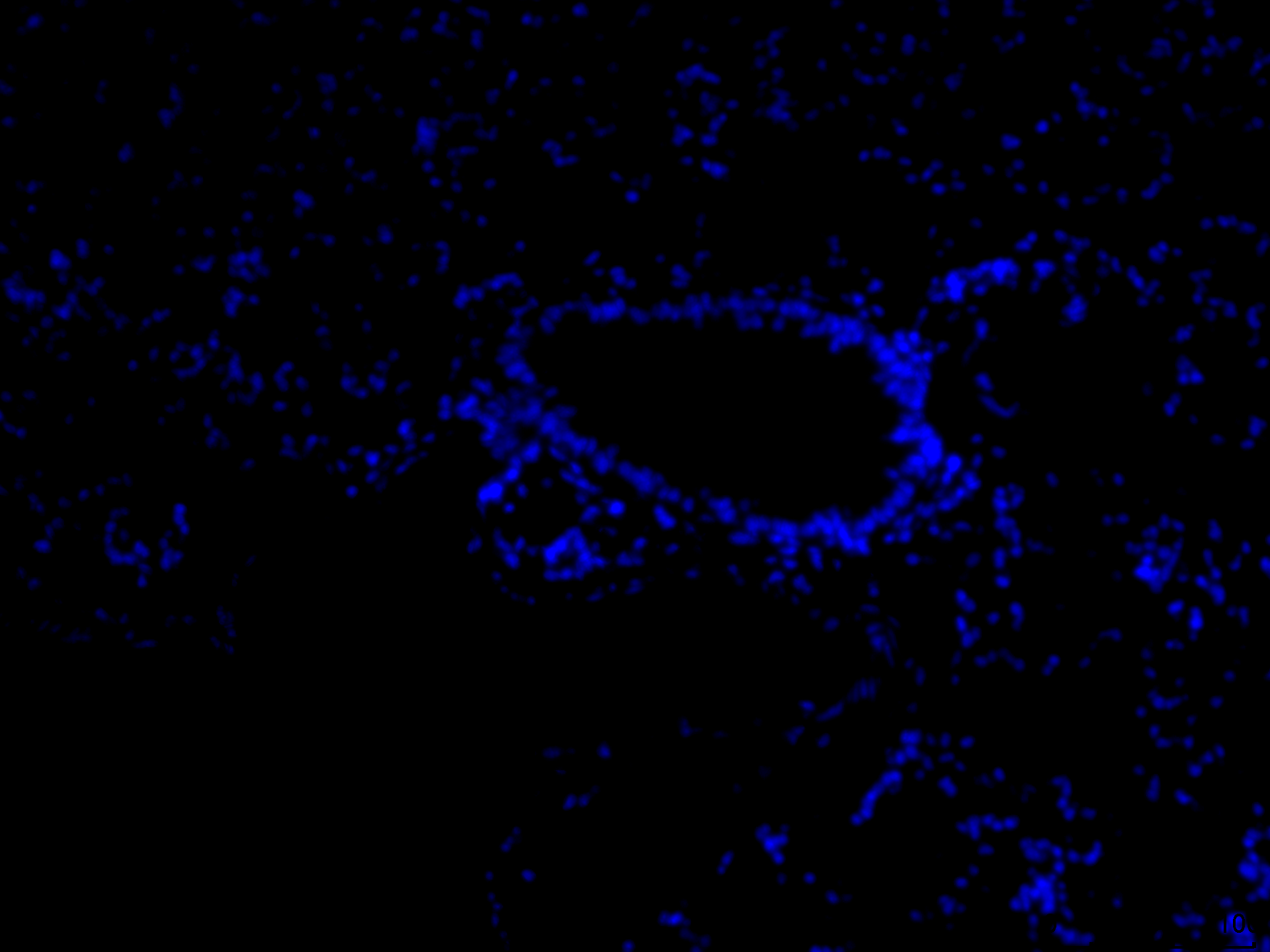

Supplement: Supplementary file 10 [file DataSheet_10.zip › Figure 10 raw datas/B. Collagen I/╢╘12.tif]

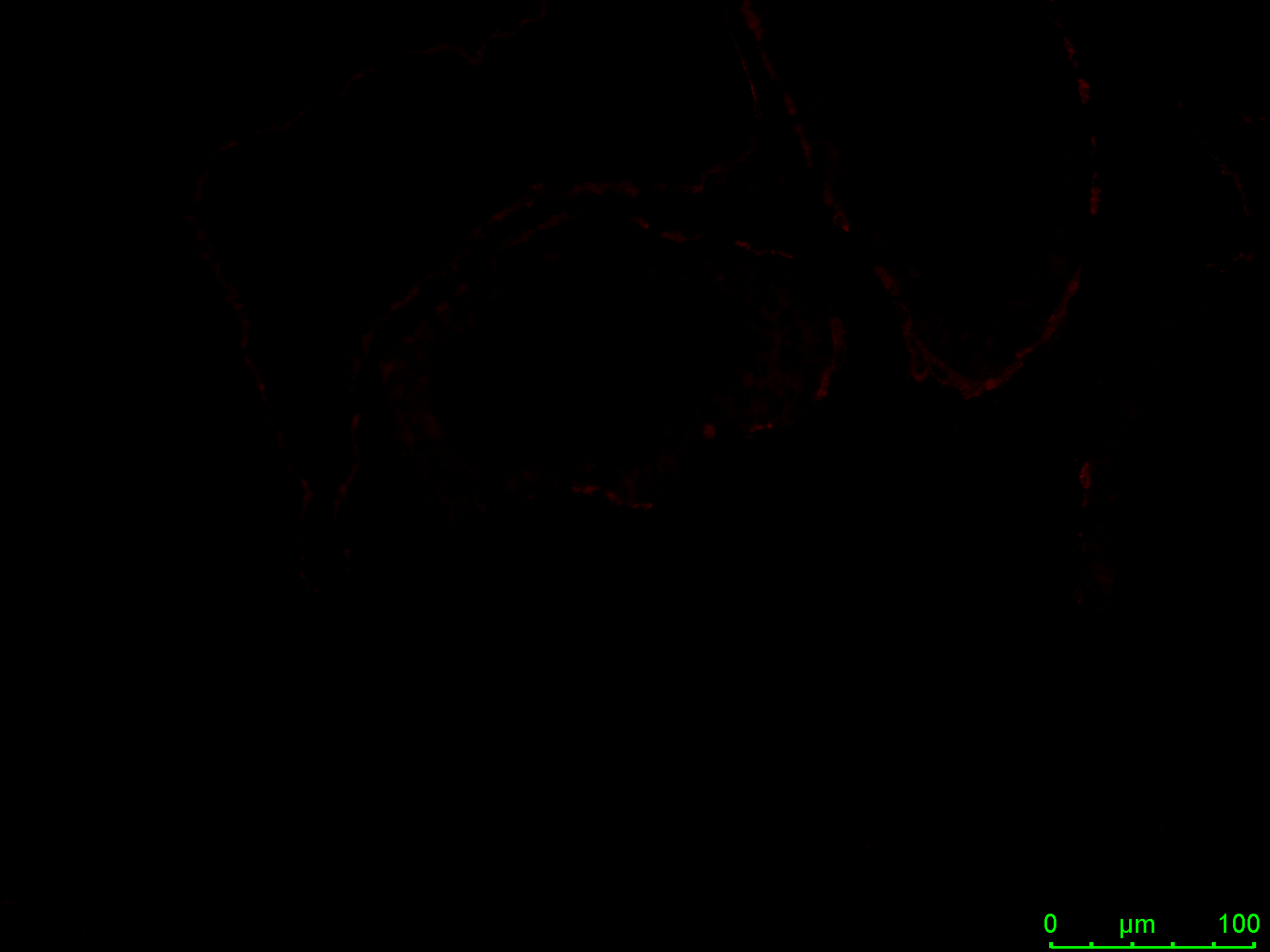

Supplement: Supplementary file 10 [file DataSheet_10.zip › Figure 10 raw datas/C. a-SMA/N11.tif]

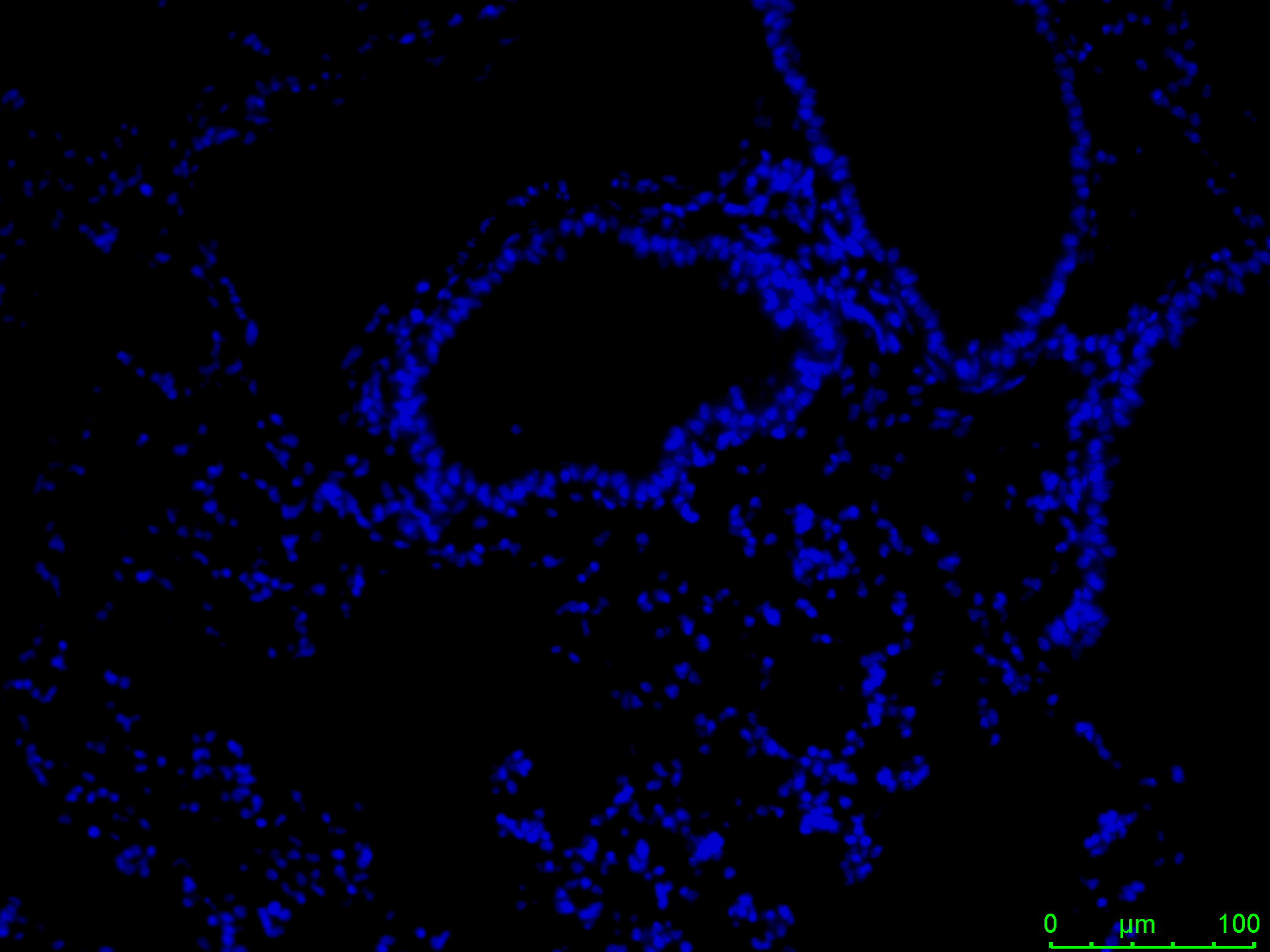

Supplement: Supplementary file 10 [file DataSheet_10.zip › Figure 10 raw datas/C. a-SMA/N12.tif]

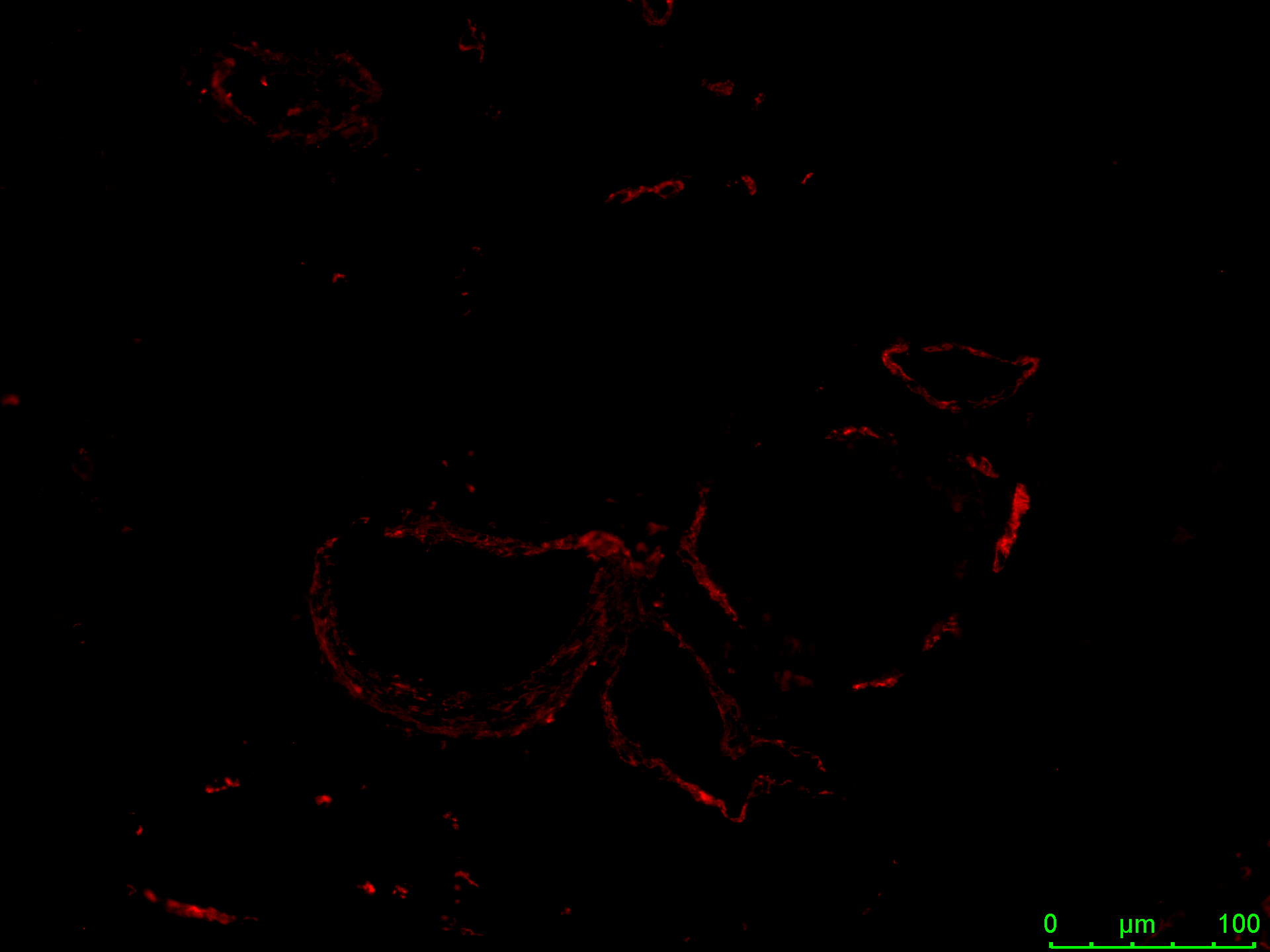

Supplement: Supplementary file 10 [file DataSheet_10.zip › Figure 10 raw datas/C. a-SMA/OVA+SDC-11.tif]

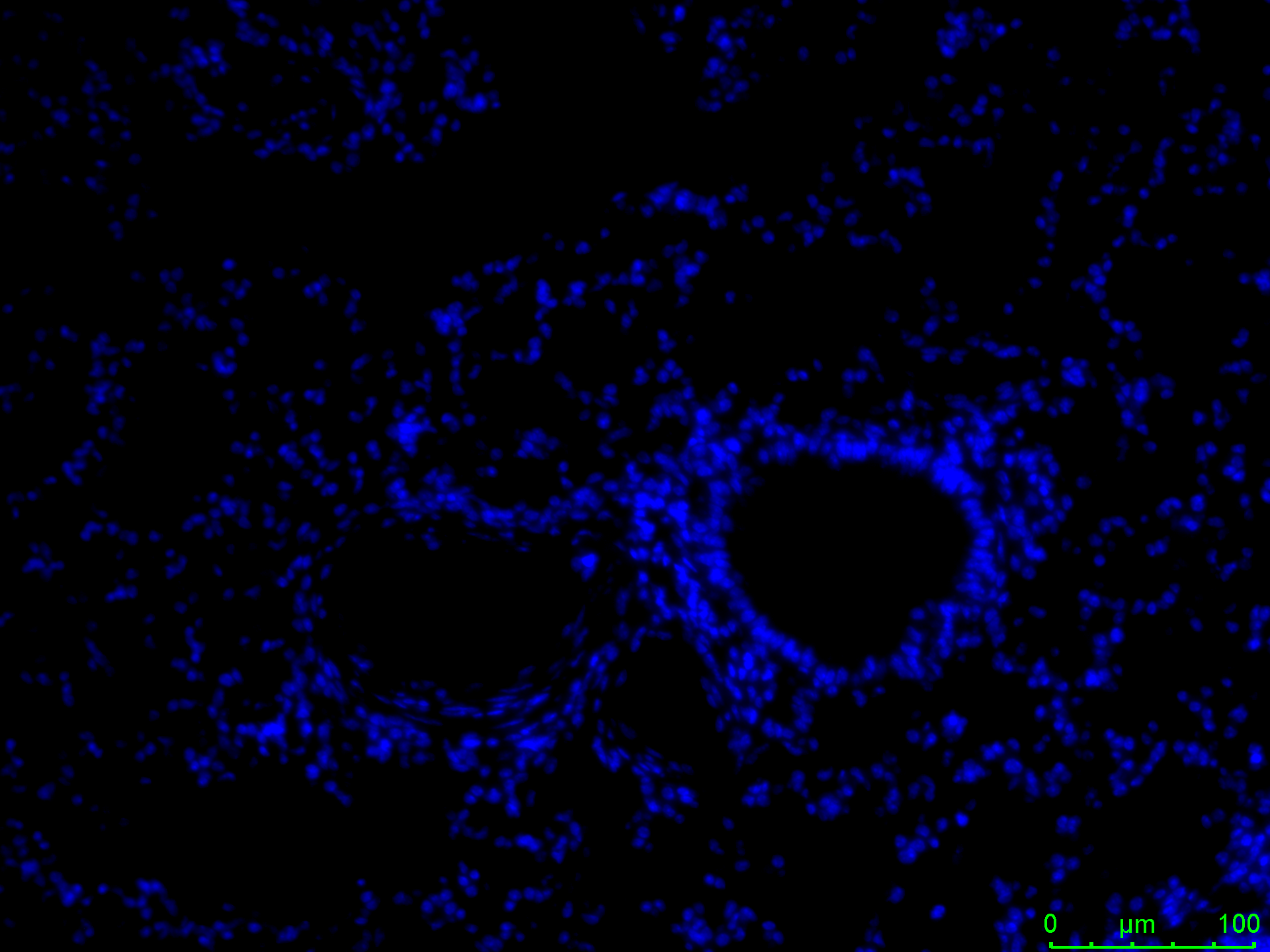

Supplement: Supplementary file 10 [file DataSheet_10.zip › Figure 10 raw datas/C. a-SMA/OVA+SDC-12.tif]

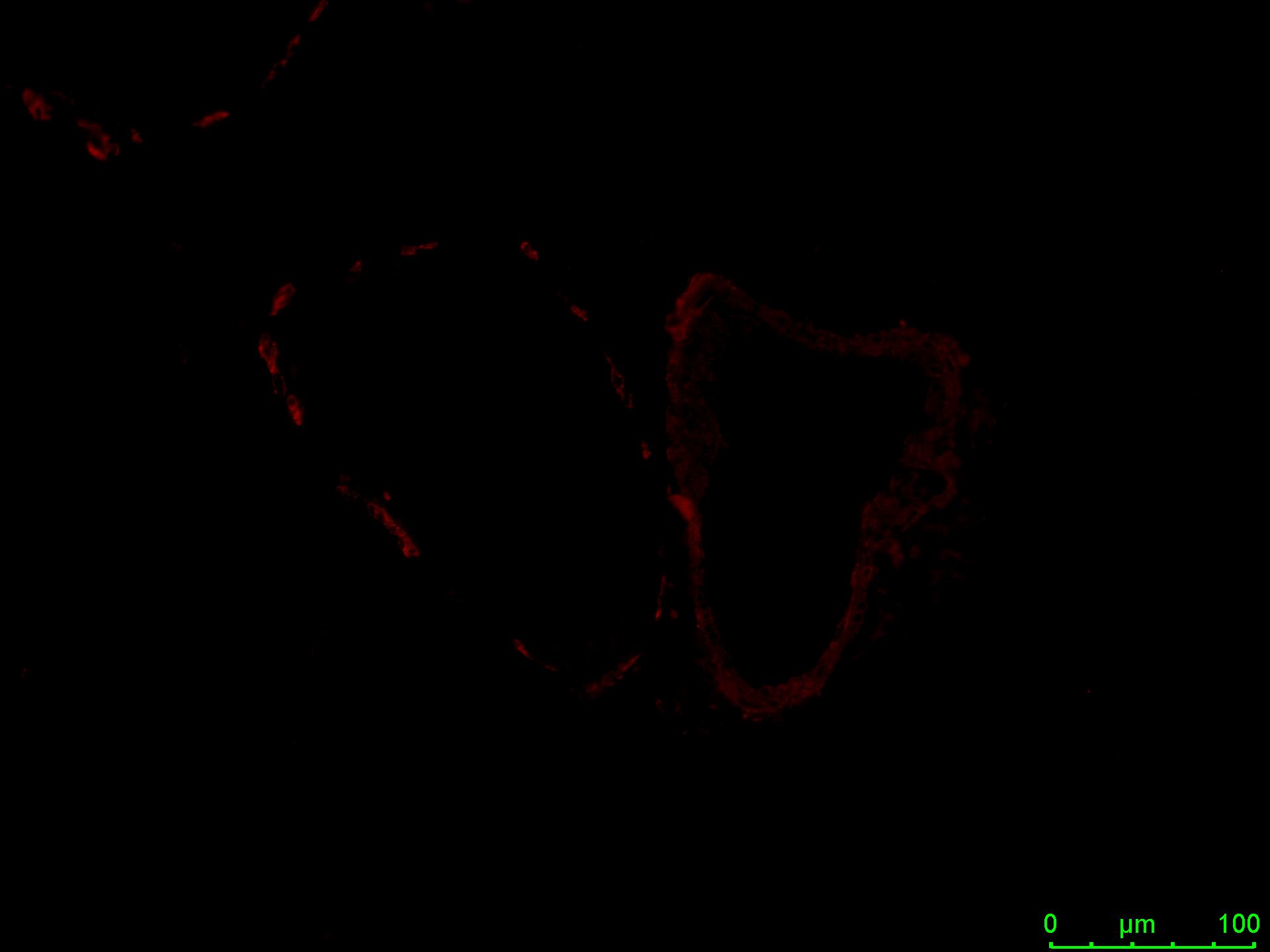

Supplement: Supplementary file 10 [file DataSheet_10.zip › Figure 10 raw datas/C. a-SMA/OVA11.tif]

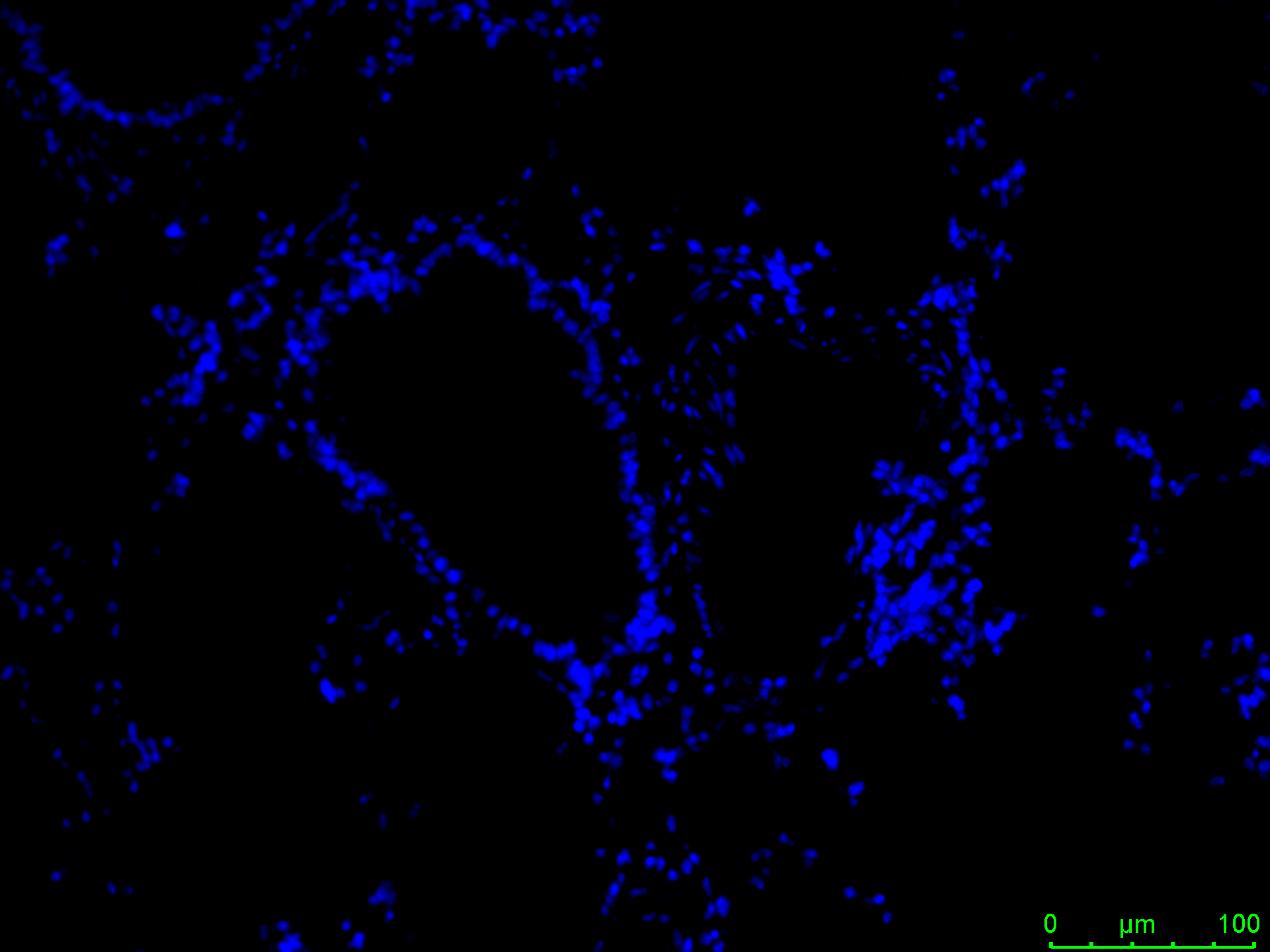

Supplement: Supplementary file 10 [file DataSheet_10.zip › Figure 10 raw datas/C. a-SMA/OVA12.tif]

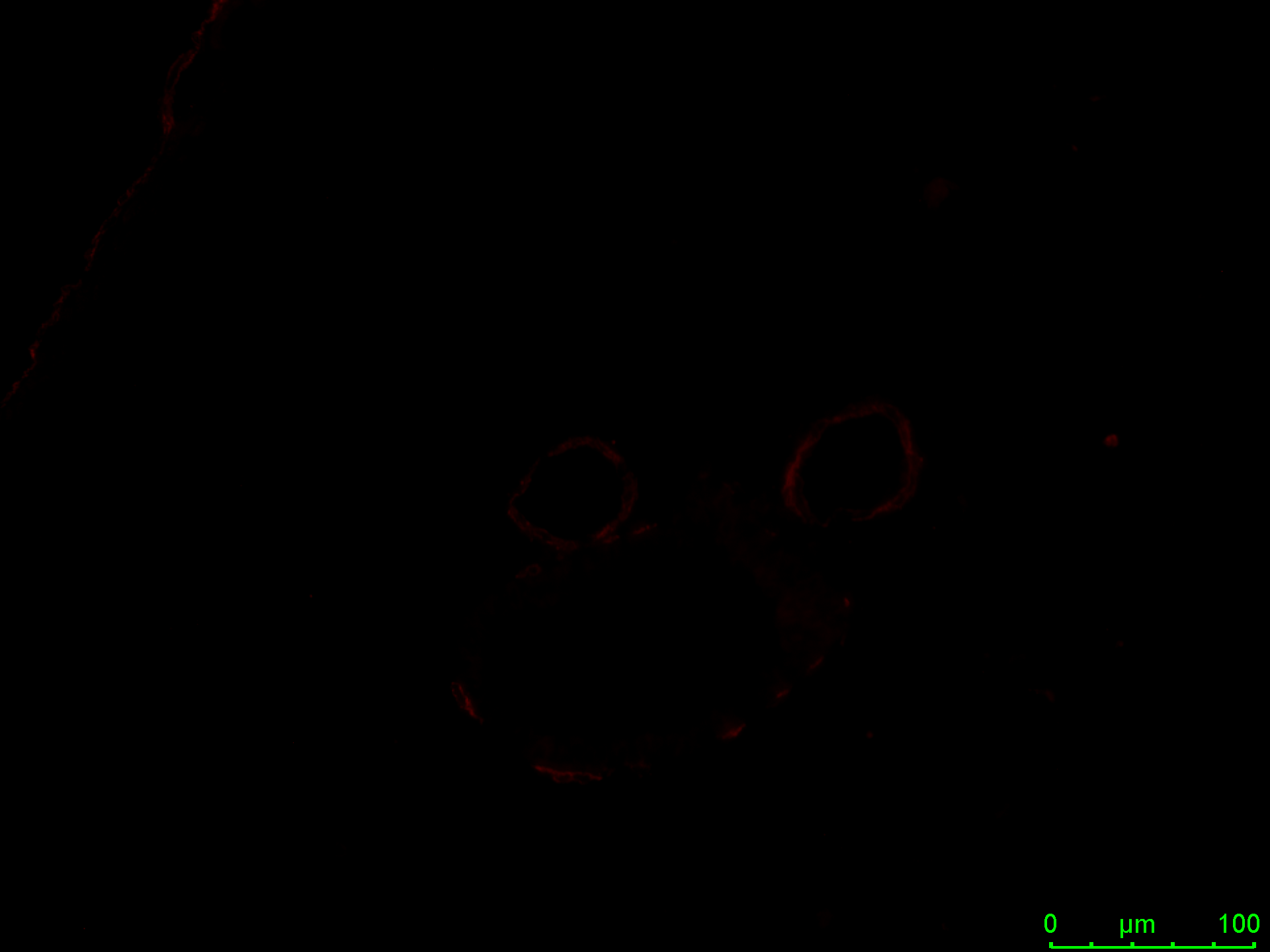

Supplement: Supplementary file 10 [file DataSheet_10.zip › Figure 10 raw datas/C. a-SMA/╢╘╒╒11.tif]

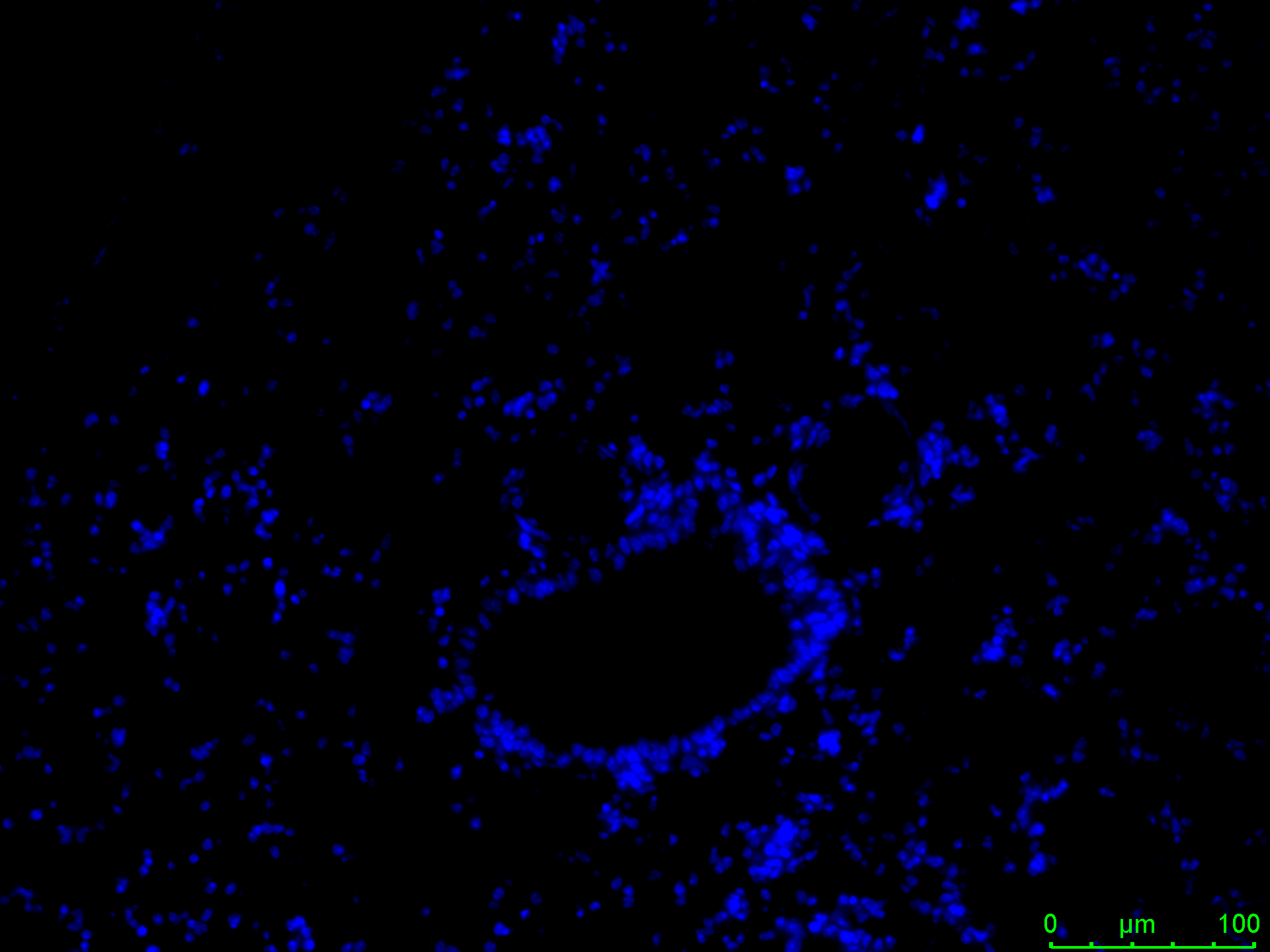

Supplement: Supplementary file 10 [file DataSheet_10.zip › Figure 10 raw datas/C. a-SMA/╢╘╒╒12.tif]

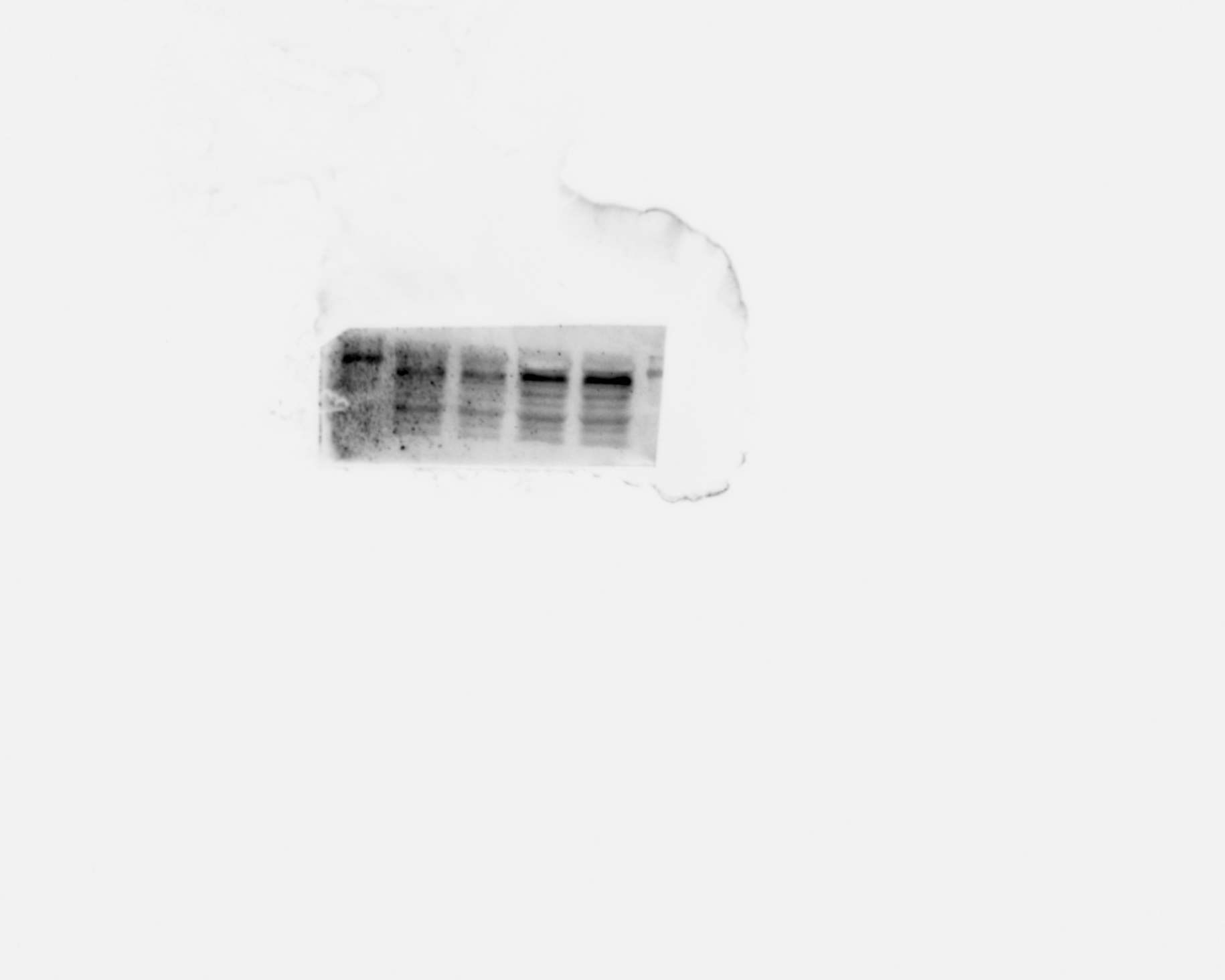

Supplement: Supplementary file 10 [file DataSheet_10.zip › Figure 10 raw datas/D. E. F WB/D. p-smad3.tif]

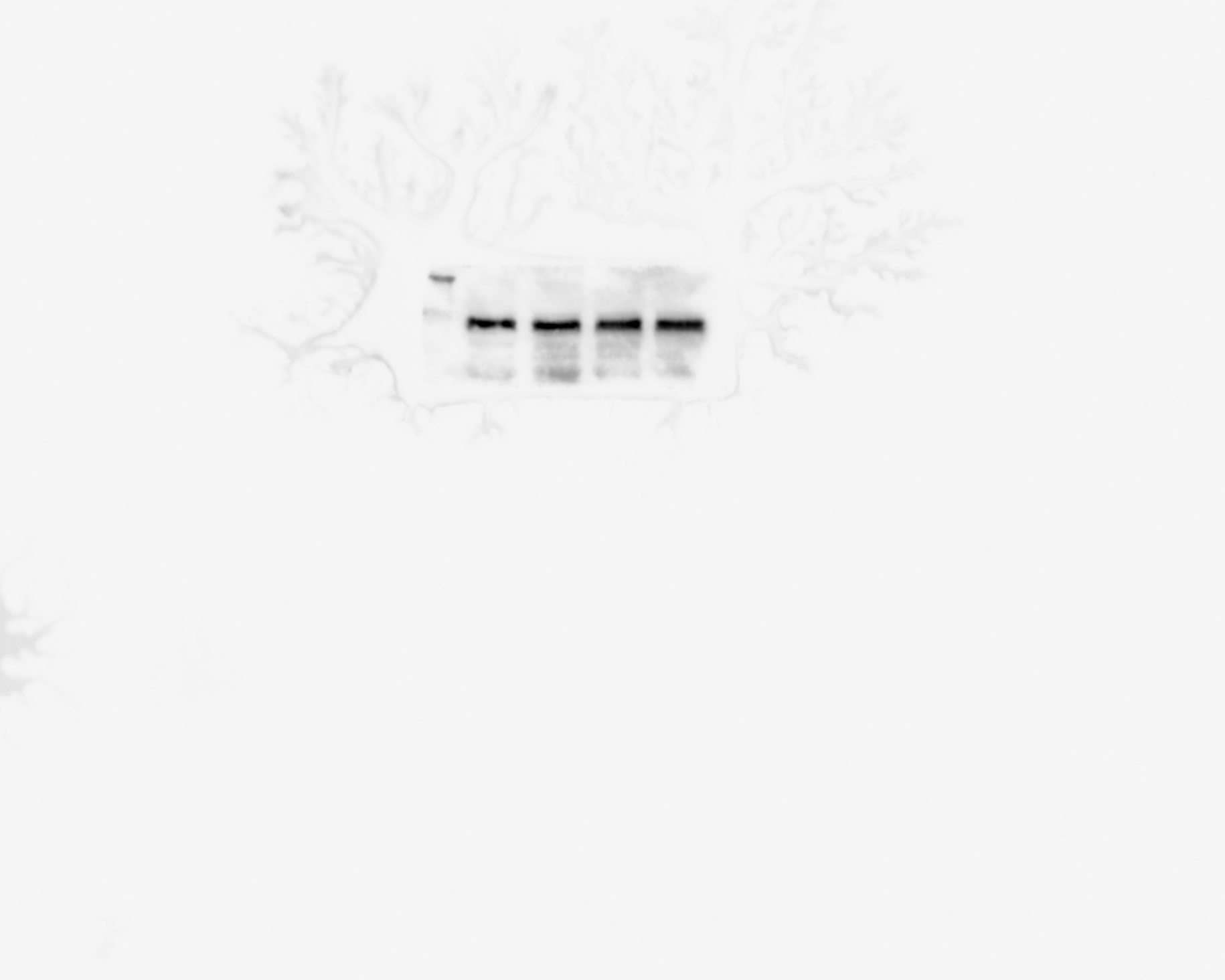

Supplement: Supplementary file 10 [file DataSheet_10.zip › Figure 10 raw datas/D. E. F WB/D. smad3.tif]

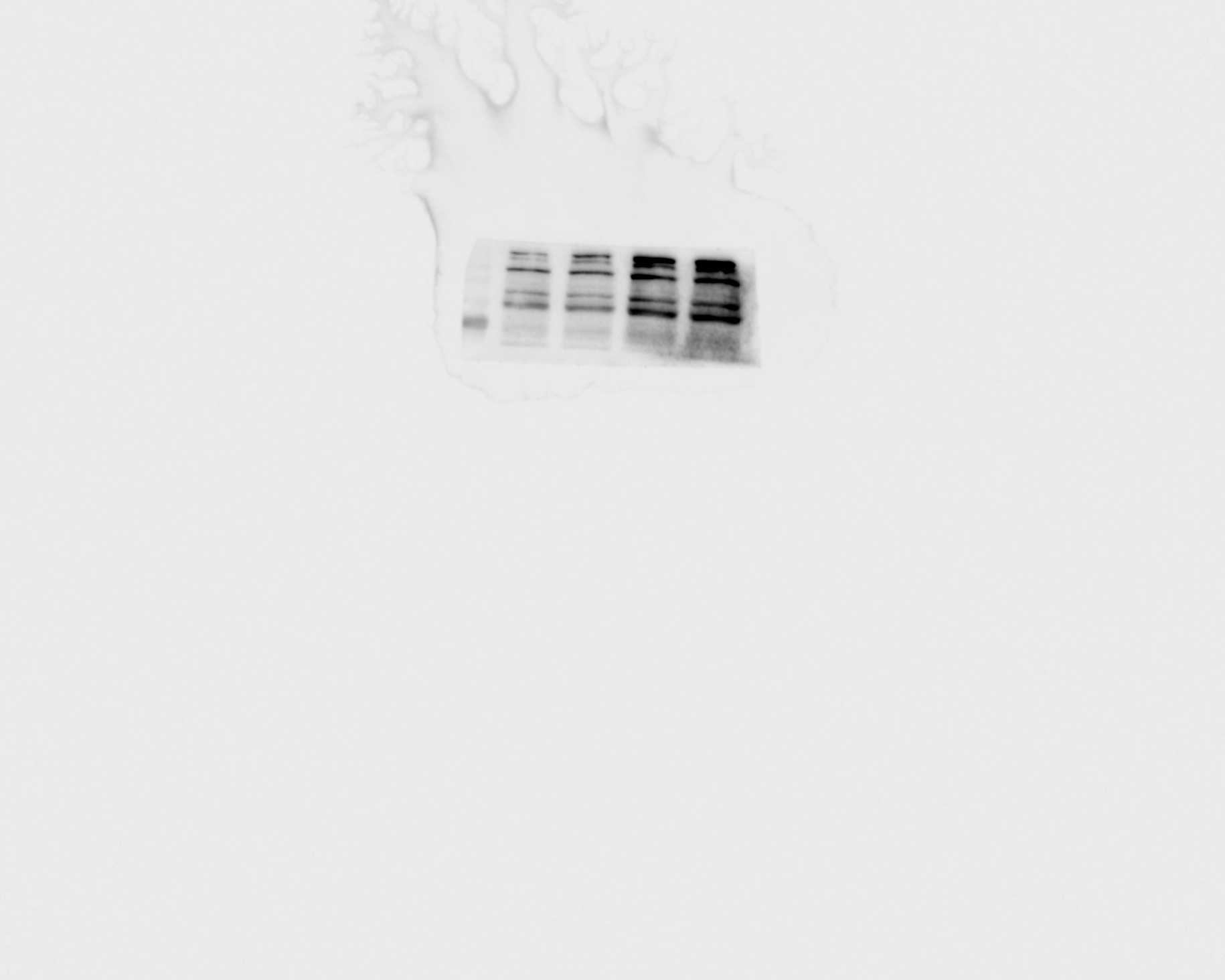

Supplement: Supplementary file 10 [file DataSheet_10.zip › Figure 10 raw datas/D. E. F WB/E. Collagen I.tif]

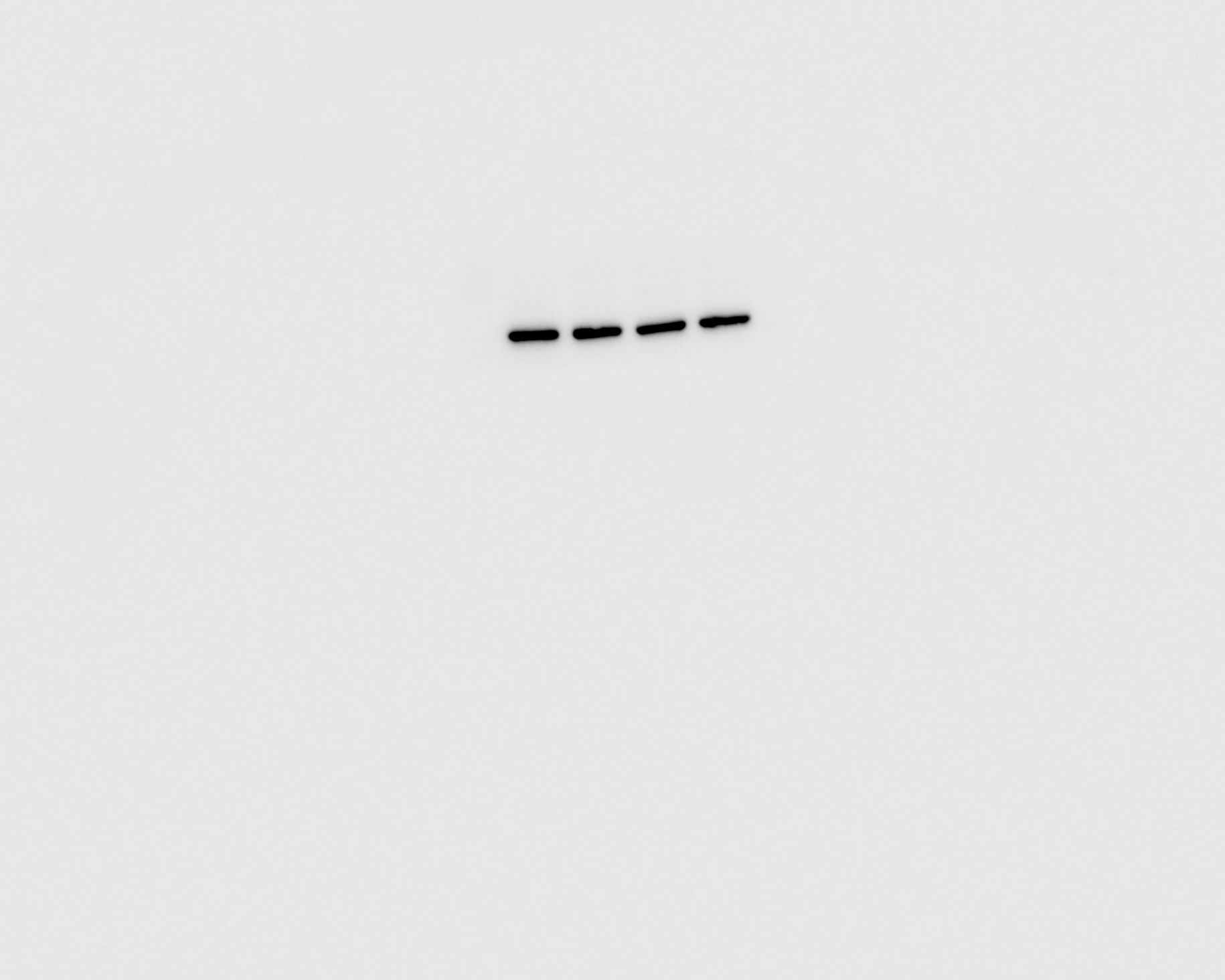

Supplement: Supplementary file 10 [file DataSheet_10.zip › Figure 10 raw datas/D. E. F WB/E. GAPDH.tif]

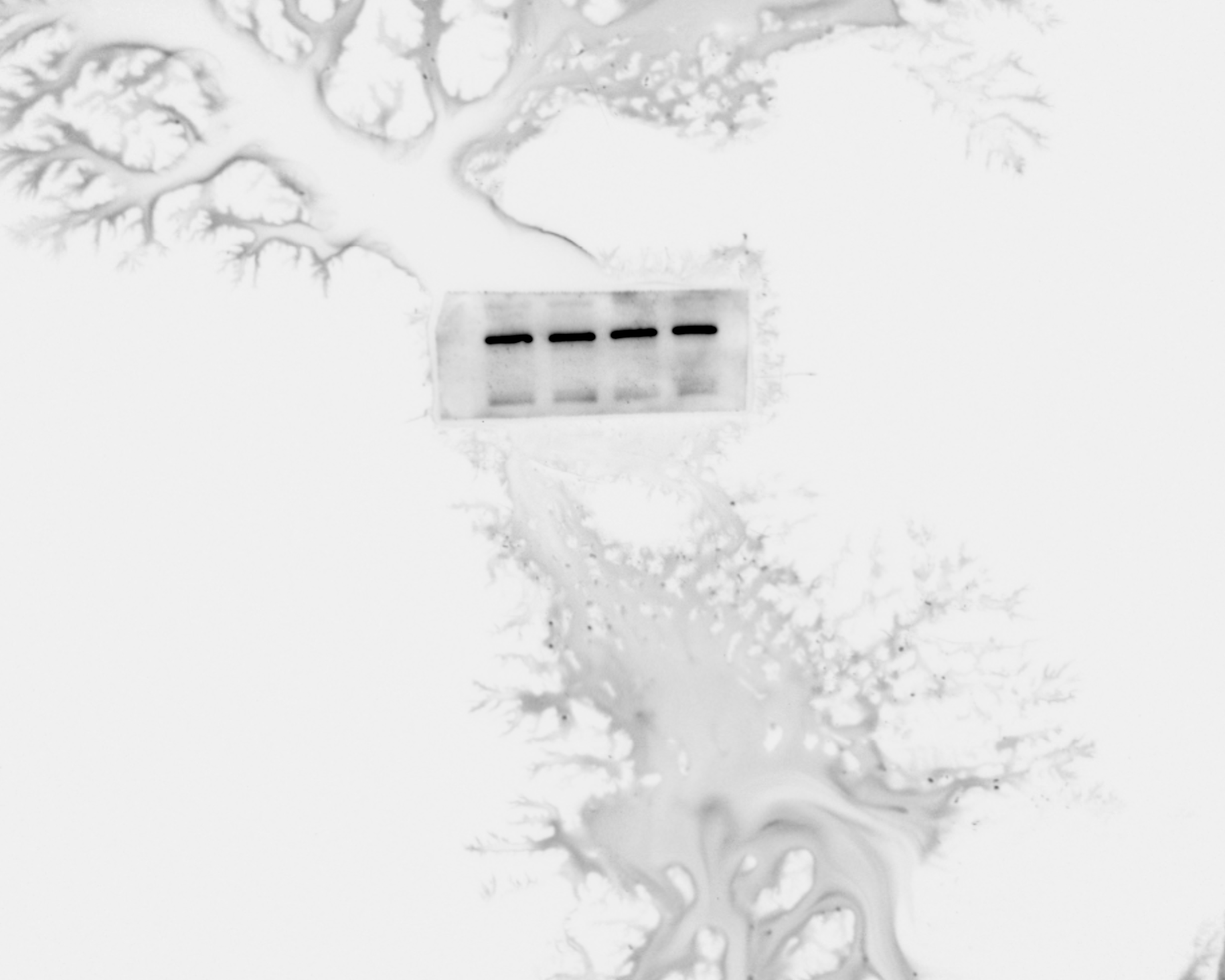

Supplement: Supplementary file 10 [file DataSheet_10.zip › Figure 10 raw datas/D. E. F WB/F. GAPDH.tif]

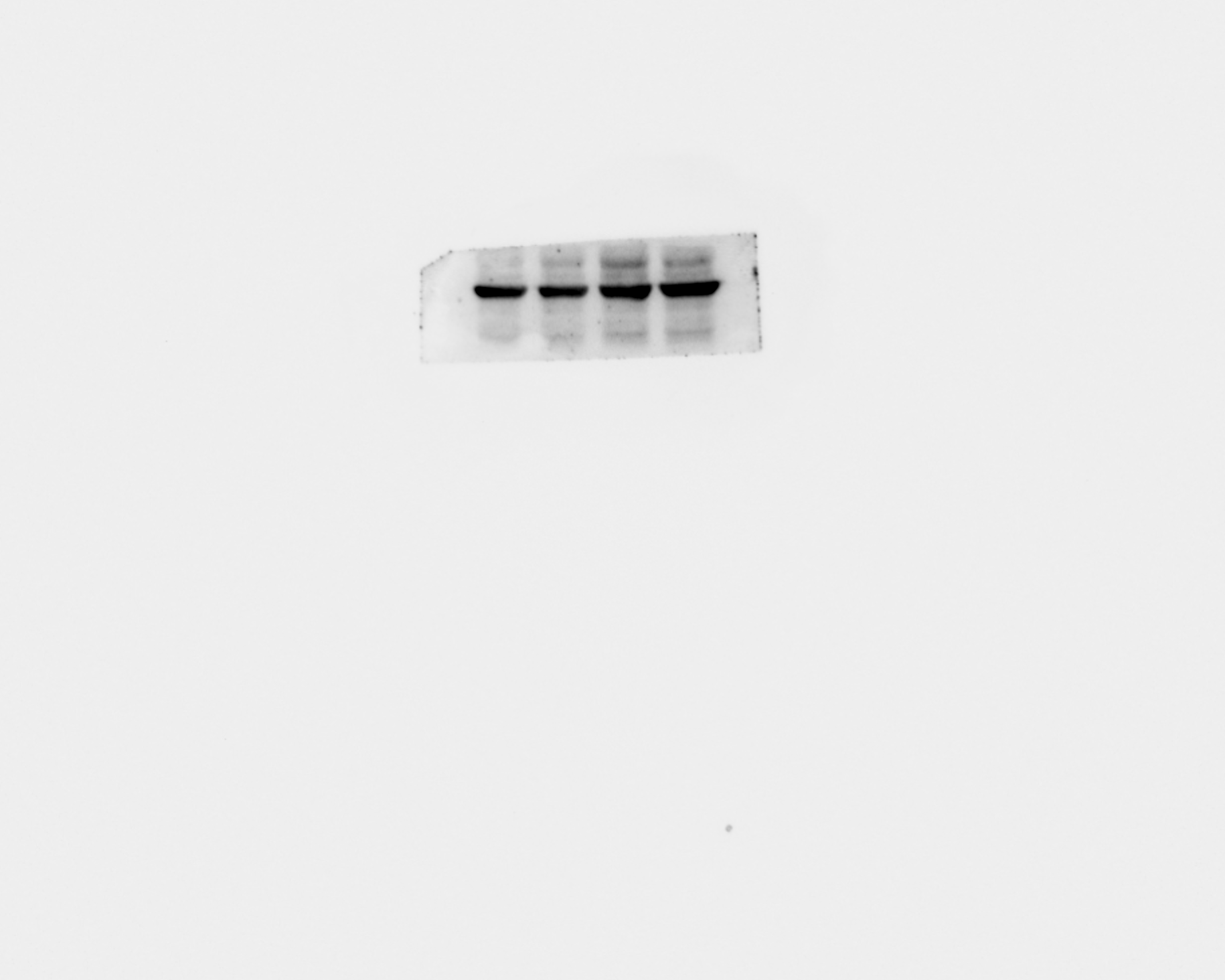

Supplement: Supplementary file 10 [file DataSheet_10.zip › Figure 10 raw datas/D. E. F WB/F. a-SMA.tif]
